# MicroRNA-Related Prognosis Biomarkers from High-Throughput Sequencing Data of Colorectal Cancer

**DOI:** 10.1155/2020/7905380

**Published:** 2020-09-09

**Authors:** Xiao-Liang Xing, Zhi-Yong Yao, Ti Zhang, Ning Zhu, Yuan-Wu Liu, Jing Peng

**Affiliations:** ^1^Xiangya Hospital, Central South University, Changsha, 410078 Hunan, China; ^2^Hunan Provincial Key Laboratory for Synthetic Biology of Traditional Chinese Medicine, School of Public Health and Laboratory Medicine, Hunan University of Medicine, Huaihua, 418000 Hunan, China; ^3^Beijing Advanced Innovation Center for Food Nutrition and Human Health, China Agricultural University, 100193 Beijing, China

## Abstract

**Background:**

Colorectal cancer (CRC) is the third most common cancer in the world, and most of them are adenocarcinomas. CRC could be classified as colon adenocarcinoma (COAD) and rectum adenocarcinoma (READ) according to the original tumorigenesis position. Increasing evidences indicated that microRNAs (miRNAs) play an important role in the occurrence of multiple tumors.

**Methods:**

In this study, we firstly downloaded miRNA (COAD, 8 controls vs. 455 tumors; READ, 3 controls vs. 161 tumors) and mRNA (COAD, 41 controls vs. 478 tumors; READ, 10 controls vs. 166 tumors) data from The Cancer Genome Atlas (TCGA) database and then used DESeq2, RegParallel, miRDB, TargetScanHuman 7.2, DAVID 6.8, STRING, and Cytoscape software to identify the potential prognosis biomarkers.

**Results:**

We identified 175 differential expression miRNAs (DEMs) and 3747 differential expression genes (DEGs) in COAD and 184 DEMs and 3928 DEGs in READ. And then, we obtained 21 (13 in COAD and 8 in READ) DEMs associated with the survival rates, which correlated with 440 (217 in COAD and 223 in READ) overlapping DEGs. Through survival analysis for those overlapping DEGs, we found 11 (8 in COAD and 3 in READ) overlapping DGEs associated with survival rates of patients, which were correlated with 9 (7 in COAD and 2 in READ) DEMs significantly.

**Conclusion:**

In this study, we found several candidate prognostic biomarkers which have been identified in various cancers and also found several new prognosis biomarkers of COAD and READ. In conclusion, this analysis based on theoretical knowledge and clinical outcomes we have done needs further confirmation by more researches.

## 1. Introduction

Colorectal cancer (CRC) is the third most prevalent cancer, accounting for 10.2% of all cancers, and the second leading cause of cancer mortality, making up 9.2% of total cancer deaths [1]. In 2018, there are approximately 1.8 million new cases around the world with 881,000 deaths [1]. Depending on the original tumorigenesis position, CRC could be classified as colon adenocarcinoma (COAD) and rectum adenocarcinoma (READ). Until now, the main efficiency therapy for CRC is limited to surgery. However, for those patients with CRC in an advanced phase, neoadjuvant therapy is indispensable to reduce the recurrence rate [2–5]. Despite the evolution of cancer pharmacological treatments providing advanced therapeutic strategies, new diagnostic and prognostic biomarkers are urgently needed [6–8].

With the development of The Cancer Genome Atlas (TCGA) database, which was based on the high-throughput sequencing, clinicians and researchers have increasingly understood the pathogenesis of various cancers. The data from TCGA is significantly important for guidance prevention, diagnosis, and treatment of various cancers. TCGA database characterized over 20,000 primary cancers and matched normal samples covering 33 cancer types, the information of which includes gene expression data, microRNA (miRNA) expression data, DNA methylation data, and standardized clinical data [9]. These are of great importance for clinicians and researchers to understand the mechanisms and find the potential prognostic biomarkers of related cancers.

miRNAs were first identified in *Caenorhabditis elegans* by Lee et al. in the 1990s [10]. They are a key component of the noncoding RNA family with 19-24 nucleotides in length and regulate the expression of target genes by partial complement binding and degradation. Increasing evidences indicate that miRNAs are involved in multiple cancers by regulating target gene expression [11–13]. In different cancers, miRNA expression patterns are different. Therefore, establishment of correlation between mRNA and miRNA expression in cancer patients is important for understanding cancer pathogenesis and discovering potential prognostic biomarkers.

In the present study, we downloaded TCGA-COAD (miRNA: 8 controls vs. 455 tumors; mRNA: 41 controls vs. 478 tumors) and TCGA-READ (miRNA: 3 controls vs. 161 tumors; mRNA: 10 controls vs. 166 tumors) project data from TCGA. Through a series of bioinformatics analyses, we identified 13 (9 downregulated and 4 upregulated) differential expression miRNAs (DEMs) and 8 (2 downregulated and 6 upregulated) DEMs related to the overall survival rate of patients with COAD and with READ, respectively. Furthermore, 8 differential expression genes (DEGs) (CACNA1D, DTNA, FZD3, PRR11, RPS6KA5, SERPINE1, TMEM178B, and TUBB6) correlated with 7 DEMs (has-miR-15b-3p, has-miR-144-5p, has-miR-130-3p, has-miR-500a-3p, has-miR-29c-5p, has-miR-486-5p, and has-miR-145-5p) were related to the survival rate of COAD, of which 6 genes (CACNA1D, DTNA, SERPINE1, TUBB6, FZD3, and RPS6KA5) were associated with pathologic TNM. And 3 DEGs (PLXNA1, PTPRU, and ZFPM2) correlated with 2 DEMs (has-miR-455-5p and has-miR-194-3p-3p) were associated with the survival rate of READ, of which 2 genes (PLXNA1 and ZFPM2) were associated with pathologic TN.

## 2. Materials and Methods

### 2.1. Data Downloading and Differential Expression Screening

All data used in the present study were downloaded from TCGA-COAD and TCGA-READ project (https://portal.gdc.cancer.gov/), including an open-access raw count table of miRNA-seq (isoform expression quantification) and RNA-seq (HTSeq-Counts) and the corresponding clinical information. The differential expression of mRNA and miRNA was analyzed under a standard workflow of the DESeq2 package installed in R (R version 3.6.2) [14]. Technical replication samples in COAD were collapsed by the collapseReplicates function of DESeq2. The significantly different expression of mRNAs and miRNAs was selected according to basemean ≥ 50, *p*adj < 0.05, and ∣logFC | ≥1.0 for COAD and READ. All normalized expression data were output for further survival analysis and pathologic TNM correlation analysis.

### 2.2. Survival Analysis for DEMs and DEGs

The miRNAs whose expression significantly changed in tumor samples were used for survival analysis by RegParallel and survival packages installed in R. Before analysis, all DEM data were transformed to a low-expression and a high-expression group according to the medium value of each normalized miRNA expression. Then, a Cox proportional hazards regression method was selected for the survival analysis combined with samples' overall survival data downloaded from TCGA. miRNAs which significantly correlated with the survival rate were selected according to *p* < 0.05 and used to predict downstream target genes. For those miRNA-predicted target genes, survival analyses were also performed by RegParallel and survival packages in the same way.

### 2.3. Target Gene Prediction of miRNAs

We utilized two different web-based tools miRDB and TargetScanHuman 7.2 for miRNA target gene prediction; the target genes only enriched in the two databases were selected as putative target genes [15, 16]. The Venn diagram was applied to visualize the overlapping DEGs of DEM target genes and DEGs in COAD and READ. To further understand the biological functions of the overlapping DEGs, we performed GO and KEGG pathway enrichment by DAVID 6.8 through input targeted gene official symbols [17].

### 2.4. Protein Interaction Network Construction and Topology Analysis

The official gene symbols were imported into the Search Tool for the Retrieval of Interacting Genes (STRING (version 11)) to assess the interactions [18]. A normal medium confidence interval of 0.4 was used as a threshold. Cytoscape software was utilized to identify and visualize central genes.

### 2.5. Pathologic TNM Correlation Analysis

Expression data of candidate DEGs were obtained from the normalized analysis of DESeq2 and normalized by the average of the control group. The pathologic TNM data were obtained from the clinical information. The classification of pathologic M is divided into two groups, M0 and M1, according to whether distant metastasis is transferred or not. The classification of pathologic N is divided into two groups, N0 and N1, according to whether lymph node metastasis is transferred or not. The classification of pathologic T is divided into two groups, T1+2 and T3+4, according to the tumor size and infiltrating range.

### 2.6. Statistical Analysis

IBM SPSS Statistics 22 was applied for the Kaplan-Meier analysis. A repeated measures ANOVA followed by unpaired two-tailed Student's *t*-test was used as indicated. All results are expressed as mean ± SEM. All figures were created using the Prism 6.01 and Cytoscape 3.7.2 software.

## 3. Results

### 3.1. Identification of DEMs and DEGs in COAD and READ

**Figure 1:**
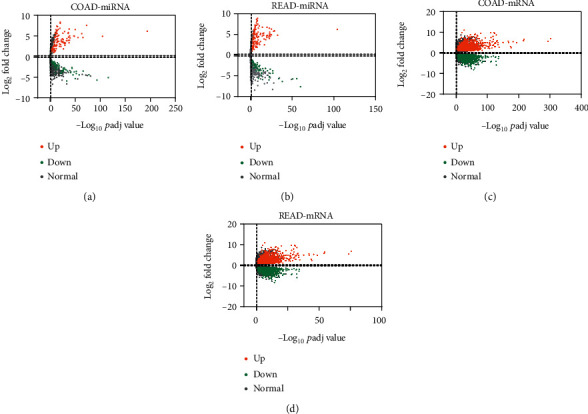
Volcano plot of differentially expressed miRNAs and mRNAs for COAD and READ. (a, b) Volcano plot of differentially expressed miRNAs for (a) COAD and (b) READ. (c, d) Volcano plot of differentially expressed mRNAs for (c) COAD and (d) READ. The threshold value was set as basemean ≥ 50, *p*adj < 0.05, and ∣logFC | ≥1.0.

We downloaded miRNA expression data from TCGA-COAD project which contains 463 samples (8 controls vs. 455 tumors). DEMs were picked up by relevant parameters (basemean ≥ 50, *p*adj < 0.05, and ∣logFC | ≥1.0). After screening, we identified 175 DEMs (67 downregulated and 108 upregulated) in COAD patients (Figure 1(a), supplementary table 1). Meanwhile, we downloaded 164 miRNA expression data of READ samples (3 controls vs. 161 tumors) through TCGA-READ project. And then, we identified 184 DEMs (59 downregulated and 125 upregulated) in READ patients (Figure 1(b), supplementary table 2).

For DEG analysis in COAD, we downloaded the gene expression data of 519 COAD samples (41 controls vs. 478 tumors) from TCGA-COAD project. By setting a series of thresholds (basemean ≥ 50, *p*adj < 0.05, and ∣logFC | ≥1.0), we identify 3747 DEGs, of which 1941 were upregulated and 1806 were downregulated (Figure 1(c), supplementary table 3). Likewise, we downloaded the gene expression data of 176 READ samples (10 controls vs. 166 tumors) and identified 3928 DEGs, of which 2078 were upregulated and 1850 were downregulated in READ (Figure 1(d), supplementary table 4).

### 3.2. Survival Analysis for DEMs in COAD and READ

Based on DEMs and relative clinical information, we used the Cox proportional hazards regression to assess their correlation with the survival status. 13 DEMs were confirmed to be significantly correlated with the survival status of patients with COAD, including 9 downregulated DEMs (hsa-miR-486-5p, hsa-miR-328-3p, hsa-miR-194-3p, hsa-miR-145-5p, hsa-miR-375-3p, hsa-miR-193b-3p, hsa-miR-501-3p, hsa-miR-29c-5p, and hsa-miR-500a-3p) and 4 upregulated DEMs (hsa-miR-130a-3p, hsa-miR-21-3p, hsa-miR-15b-3p, and hsa-miR-144-5p) (Table 1).

In the same way, we confirmed that 8 DEMs were significantly correlated with the survival status of patients with READ, including 2 downregulated DEMs (hsa-miR-194-3p and hsa-miR-29c-5p) and 6 upregulated DEMs (hsa-miR-15a-5p, hsa-miR-98-5p, hsa-miR-106b-5p, hsa-miR-455-5p, hsa-miR-21-5p, and hsa-miR-552-5p) (Table 2). In the intersection of the DEMs of COAD and READ, we identified two shared miRNAs: hsa-miR-194-3p and hsa-miR-29c-5p.

### 3.3. Prediction of DEM Target Genes and Functional Analysis

**Figure 2:**
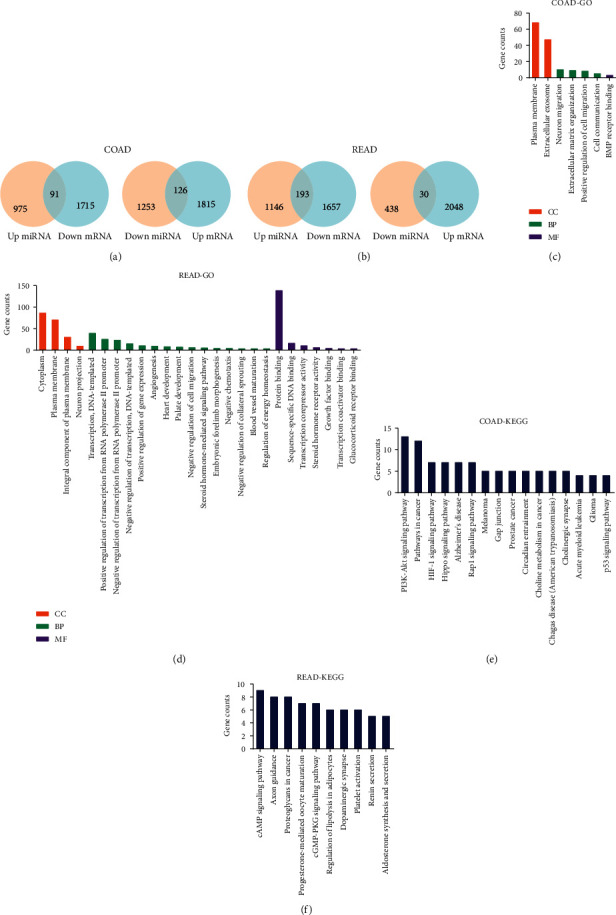
Prediction of target genes and functional analysis. (a, b) Overlapping DEGs and DEM target genes for (a) COAD and (b) READ. (c, d) The significantly enriched GO term (*p* value < 0.01) analyzed by using DAVID 6.8 for (c) COAD and (d) READ. (e, f) The significantly enriched KEGG pathway (*p* value < 0.05) analyzed by using DAVID 6.8 for (e) COAD and (f) READ. CC: cellular component (orange); BP: biological process (green); MF: molecular functions (purple).

Followed by DEM identification in COAD and READ, the target genes of those DEMs were predicted by using two different online tools, miRDB and TargetScanHuman 7.2. Combining those DEM-targeted genes with DEGs, we identified 217 overlapping DEGs, of which 91 overlapping DEGs were downregulated and 126 overlapping DEGs were upregulated in COAD (Figure 2(a)). And 223 overlapping DEGs were identified, of which 193 overlapping DEGs were downregulated and 30 overlapping DEGs were upregulated in READ (Figure 2(b)).

To investigate the biological effects of those overlapping DEGs, we performed GO and KEGG analyses by using DAVID 6.8. The results of GO analysis for COAD and READ are displayed in Figures 2(c) and 2(d) and supplementary tables 5 and 6. The results of KEGG analysis for COAD and READ are displayed in Figures 2(e) and 2(f) and supplementary tables 7 and 8.

### 3.4. PPI Network Analysis and Verification of Prognostic Genes

**Figure 3:**
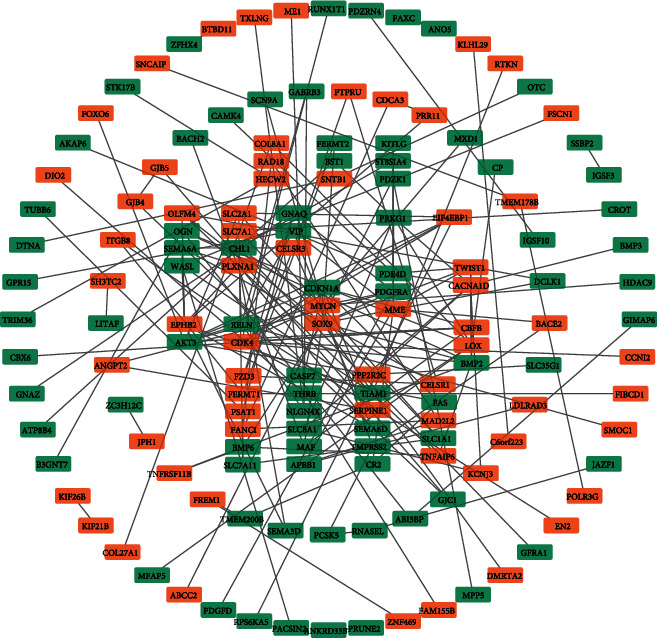
Constructed PPI networks of related genes in COAD. The PPI network was constructed by using the overlapping genes in COAD. Orange represents unregulated genes; green represents downregulated genes.

**Figure 4:**
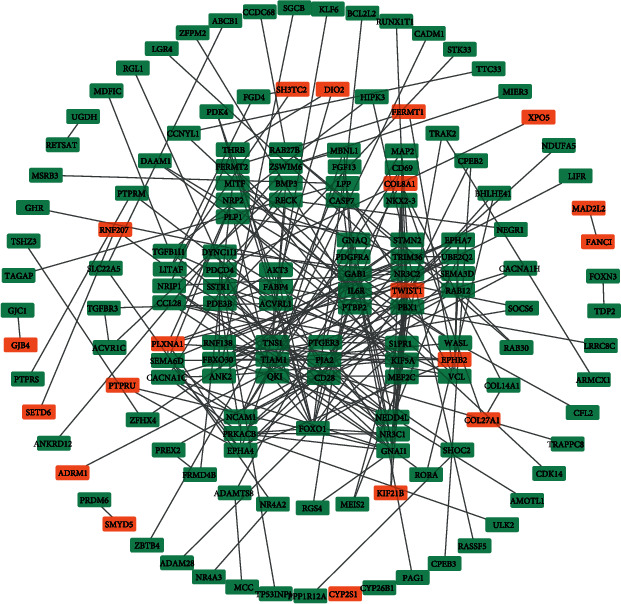
Constructed PPI networks of related genes in READ. The PPI network was constructed by using the overlapping genes in READ. Orange represents unregulated genes; green represents downregulated genes.

**Figure 5:**
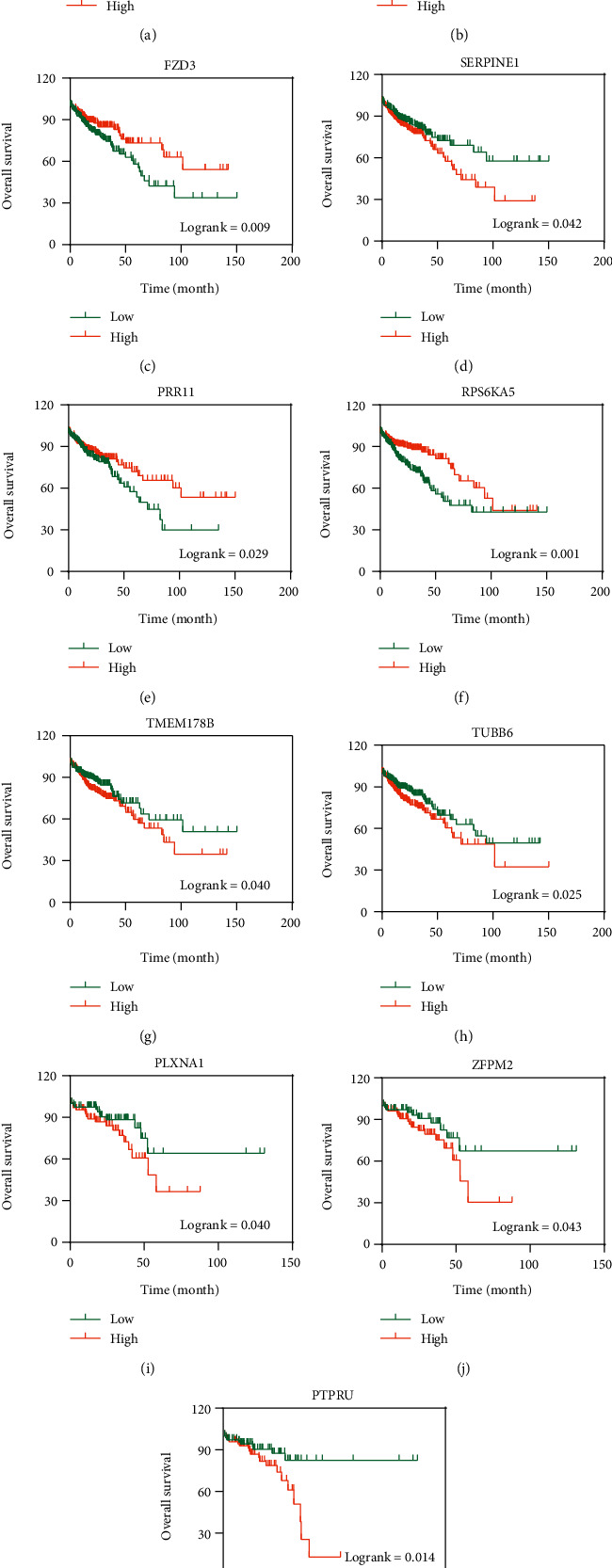
The survival curve of target genes for COAD and READ: (a–h) survival curves of target genes in COAD; (i–k) survival curves of target genes in READ.

We used the online tool STRING to assess the interactions of those overlapping DEGs and then Cytoscape 3.7.2 to visualize their interactions. The PPI network contained 217 nodes and 179 edges for COAD (Figure 3) and 223 nodes and 233 edges for READ (Figure 4). Once a gene connected with multiple genes, it is considered to have more important biological functions. So we chose those overlapping DEGs (including 145 overlapping DEGs in COAD and 153 overlapping DEGs in READ) which have one connection at least for subsequent analysis. By means of the Kaplan-Meier analysis, we found 8 overlapping DEGs (CACNA1D, DTNA, FZD3, PRR11, RPS6KA5, SERPINE1, TMEM178B, and TUBB6) in COAD (Figures 5(a)–5(h)) and 3 DEGs (PLXNA1, PTPRU, and ZFPM2) in READ which were correlated with the survival rate significantly (Figures 5(i)–5(k)).

**Figure 6:**
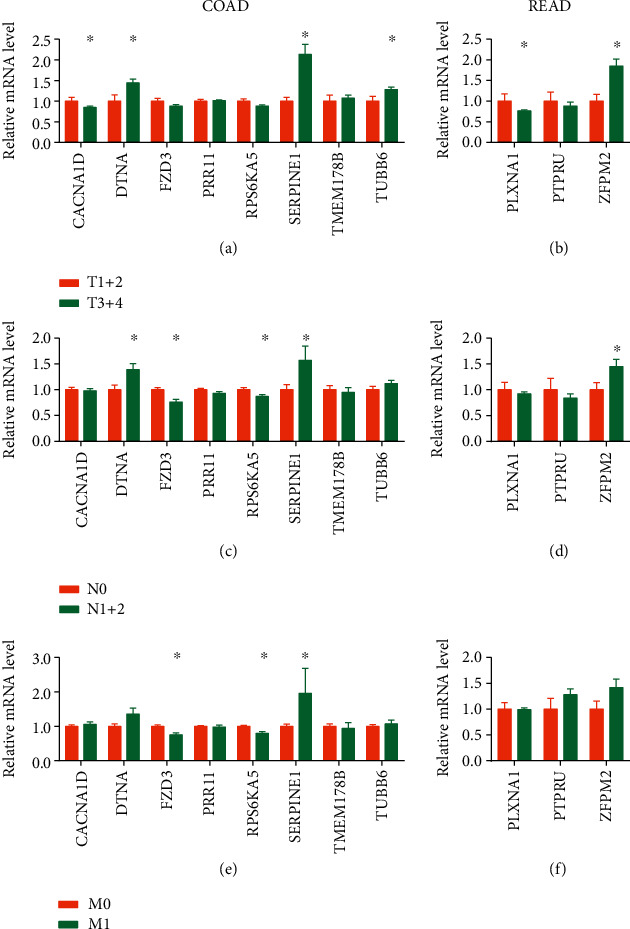
Associated analyses of target genes with pathologic TNM. (a, b) Associated analyses of target genes with a pathologic T stage for (a) COAD and (b) READ. (c, d) Associated analyses of target genes with a pathologic N stage for (c) COAD and (d) READ. (e, f) Associated analyses of target genes with a pathologic T stage for (e) COAD and (f) READ. A repeated measures ANOVA followed by unpaired two-tailed Student's *t*-test was used as indicated. All results are expressed as mean ± SEM.

Then, we evaluated the relationship between those 11 overlapping DEGs (8 in COAD and 3 in READ) related to the survival rate of patients and pathologic TNM. The results indicated that 6 overlapping DEGs (CACNA1D, DTNA, FZD3, RPS6KA5, SERPINE1, and TUBB6) were associated with pathologic TNM (Figures 6(a), 6(c), and 6(e)), of which 4 overlapping DEGs (CACNA1D, DTNA, SERPINE1, and TUBB6) were associated with pathologic T (Figure 6(a)), 4 overlapping DEGs (DTNA, FZD3, RPS6KA5, and SERPINE1) were associated with pathologic N (Figure 6(c)), and 3 overlapping DEGs (FZD3, RPS6KA5, and SERPINE1) were associated with pathologic M (Figure 6(e)) in COAD. The results also showed that 2 overlapping DEGs (PLXNA1 and ZFPM2) were associated with pathologic TN (Figures 6(b), 6(d), and 6(f)), of which 2 overlapping DEGs (PLXNA1 and ZFPM2) were associated with pathologic T (Figure 6(b)) and 1 overlapping DEG (ZFPM2) was associated with pathologic N (Figure 6(d)) in READ.

**Figure 7:**
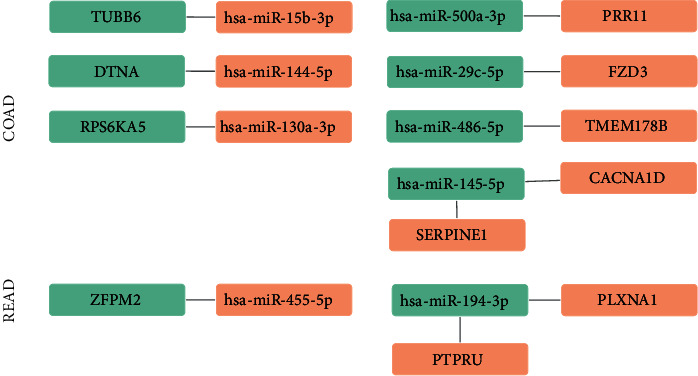
Network of COAD- and READ-related miRNAs and target genes. Orange represents unregulated genes; green represents downregulated genes.

By retrospective examination of the confirmed survival-associated overlapping DEGs with DEMs, we found that those downregulated overlapping DEGs TUBB5, DTNA, and RPS6KA5 were correlated with has-miR-15b-3p, has-miR-144-5p, and has-miR-130, respectively. And those upregulated overlapping DEGs PRR11, FZD3, TMTM178B, CACNA1D, and SERPINE1 were correlated with has-miR-500a-3p, has-miR-29c-5p, has-miR-486-5p, and has-miR-145-5p, respectively, in COAD as shown in Figure 7. The downregulated overlapping DEG ZFPM2 was correlated with has-miR-455-5p. Upregulated overlapping DEGs PLXNA1 and PTPRU were correlated with has-miR-194-3p in READ as shown in Figure 7.

## 4. Discussions

CRC is the third most prevalent cancer and the second leading cause of cancer mortality worldwide. Up to now, surgery is still the main treatment method in clinical practice [1]. In addition, for those patients in advanced cancer stages, the implementation of neoadjuvant therapy to reduce the recurrence rate and sphincter malfunction rate of colorectal cancer is indispensable [3]. Therefore, an accurate preoperative assessment of CRC is of great importance. In the present study, we analyzed the COAD and READ sequencing data from TCGA in order to reveal the key genes correlated with miRNAs and to evaluate their potential for diagnosis and treatment as biomarkers.

Increasing studies have shown that miRNAs play an important role in the diagnosis and treatment of cancers [19–22]. Through data mining of sequencing results deposited in TCGA, we identified 21 DEMs (13 DEMs in COAD and 8 DEMs in READ) associated with the survival rates and simultaneously negatively correlated with 440 (217 DEGs in COAD and 223 DEGs in READ) overlapping DEGs. For those 21 DEMs in COAD and READ, previous studies demonstrated that several miRNAs are indeed associated with survival in cancer patients, such as has-miR-328 in pancreatic cancer, has-miR-486 in non-small-cell lung cancer, and has-miR-145 in breast cancer and laryngeal cancer [23–26]. For those overlapping DEGs, we then analyze their correlation with the survival status. Finally, we found 8 and 3 DEGs which were correlated with 7 and 2 DEMs significantly associated with patients' overall survival in COAD and READ, respectively.

For those DEMs used in the miRNA-mRNA network, miR-144-5p, miR-145-5p, and miR-486-5p have been reported as biomarkers in bladder cancer, gastric cancer, and lung cancer, respectively [27–29]. Our results indicated that the three miRNAs have potential to be prognostic biomarkers in COAD as well. It is reported that other four miRNAs, miR-29c-5p, miR-15b-3p, miR-130a-3p, and miR-500a-3p, were associated with various cancers but not recommended as prognostic markers [30–33]. Our results suggest that those DEMs (miR-144-5p, miR-145-5p, miR-486-5p, miR-29c-5p, miR-15b-3p, miR-130a-3p, and miR-500a-3p) may serve as potential biomarkers, but their feasibility remains to be investigated. For those DEMs in READ, miR-455-5p was associated with oral squamous cancer [34]. However, few evidences indicate the association between miR-194-3p and cancers. Our results suggested that those DEMs (miR-455-5p and miR-194-3p) may also serve as potential biomarkers, but their feasibility remains to be further validated.

As for miRNA-targeted genes in COAD, TUBB6, FZD3, and SERPINE1 were reported to be prognostic biomarkers in colorectal cancer potentially [35–37]. According to the previous literature, one of the causes of sporadic colorectal cancers is RPS6KA5 frameshift mutation, which leads to the same consequence as being targeted by miRNAs [38]. At last, the four genes, DTNA, PRR11, TMEM178B, and CACNA1D, were mainly related to gastric cancer or prostate cancer [39–42]. In miRNA-targeted genes of READ, ZFPM2 was recommended as a diagnostic biomarker for malignant pleural mesothelioma and PLXNA1 and PTPRU were related to lung cancer or colon cancer [43–46]. Combining the expression levels of those target genes with TNM stage classification, SERPINE1 is the most important gene associated with tumor size and metastasis status in COAD. And miR-145-5p, targeting SERPINE1, could be another strong prognosis biomarker for COAD. Further research is urgently needed to verify the detailed regulation pattern between miR-145-5p and SERPINE1 in a COAD patient, and this might be a valuable target in COAD therapy since therapy using small RNA is safer than other methods in gene therapy [47]. For READ, no DEGs have been found through all TNM stages. It is most likely that the stringent criteria we set cannot screen out ideal DEMs and their target genes. There are many reports presenting potential prognostic miRNAs in colorectal cancer, but they just analyzed the microarray data without comparison with gene expression data or focused on hypoxia stress to cancer progression [48, 49]. Another research analyzed the differentially expressed miRNA and its potential target in COAD without considering the survival effect on the screened genes [50], which cannot be sufficiently used in clinical practice as prognostic biomarkers.

In the present study, we downloaded TCGA-COAD (miRNA: 8 controls vs. 455 tumors; mRNA: 41 controls vs. 478 tumors) and TCGA-READ (miRNA: 3 controls vs. 161 tumors; mRNA: 10 controls vs. 166 tumors) project data from TCGA. Through a series of bioinformatics analyses, we found that several potential DEMs could be used as prognostic biomarkers in COAD and READ. However, due to the small number of samples in the control group, false-positive or false-negative results might be inevitable in our results. In addition, by the own complexity of cancers, the combined analysis strategy may be more accurate than that using the individual miRNA or mRNA as the prognostic indicator [23]. Therefore, further analysis in READ and COAD is necessary to improve the accuracy of prediction results.

## 5. Conclusions

In conclusion, we identified that 7 DEMs and 8 DEGs were correlated with the survival of patients with COAD, which may be the potential biomarkers for the prognosis of patients with COAD, and 2 DEMs and 3 DEGs were correlated with the 232survival of patients with READ, which may be the potential biomarkers for the prognosis of patients with READ through bioinformatics analysis for COAD- and READ-related miRNAs and mRNAs systematically. Our research not only deepened the understanding of the pathogenesis but also contributed to the diagnosis and prognosis of patients with COAD and READ. However, it is worth emphasizing that the regulatory network of miRNA-mRNA is particularly complex and the number of case and control data used in the study was not sufficient. Therefore, more scientific researches are needed to confirm our findings while we just provide an analysis direction depending on theoretical knowledge and clinical outcomes.

## Figures and Tables

**Table 1:** Prognostic DEMs for COAD in Cox proportional hazards regression analysis.

miRNA	*p*	Logrank	HR	HRlower	HRupper
hsa-miR-486-5p	0.014	0.013	0.60	0.41	0.90
hsa-miR-328-3p	0.033	0.032	1.54	1.04	2.29
hsa-miR-194-3p	0.025	0.024	0.63	0.43	0.94
hsa-miR-145-5p	0.024	0.023	1.58	1.06	2.35
hsa-miR-375-3p	0.029	0.028	0.64	0.43	0.96
hsa-miR-193b-3p	0.047	0.045	1.50	1.01	2.23
hsa-miR-501-3p	0.042	0.041	0.66	0.45	0.99
hsa-miR-29c-5p	0.041	0.039	1.52	1.02	2.26
hsa-miR-500a-3p	0.010	0.009	0.59	0.40	0.88
hsa-miR-130a-3p	0.006	0.006	1.75	1.17	2.62
hsa-miR-21-3p	0.001	0.001	0.50	0.33	0.75
hsa-miR-15b-3p	0.044	0.043	0.66	0.44	0.99
hsa-miR-144-5p	0.048	0.046	0.67	0.45	1.00

**Table 2:** Prognostic DEMs for READ in Cox proportional hazards regression analysis.

miRNA	*p*	Logrank	HR	HRlower	HRupper
hsa-miR-194-3p	0.019	0.015	0.37	0.16	0.85
hsa-miR-29c-5p	0.030	0.025	2.41	1.09	5.33
hsa-miR-15a-5p	0.041	0.036	0.43	0.20	0.97
hsa-miR-98-5p	0.017	0.013	2.86	1.21	6.79
hsa-miR-106b-5p	0.036	0.030	2.32	1.06	5.07
hsa-miR-455-5p	0.013	0.009	0.33	0.14	0.79
hsa-miR-21-5p	0.035	0.030	0.41	0.18	0.94
hsa-miR-552-5p	0.009	0.006	0.33	0.14	0.76

## Data Availability

The data that support the findings of this study are openly available in TCGA at https://portal.gdc.cancer.gov/.

## Supplementary Materials

Supplementary materials Supplementary table 1: differentially expressed miRNAs for COAD. Supplementary table 2: differentially expressed miRNAs for READ. Supplementary table 3: differentially expressed mRNAs for COAD. Supplementary table 4: differentially expressed mRNAs for READ. Supplementary table 5: GO analysis of overlapping DEGs for COAD. Supplementary table 6: GO analysis of overlapping DEGs for READ. Supplementary table 7: KEGG analysis of overlapping DEGs for COAD. Supplementary table 8: KEGG analysis of overlapping DEGs for READ.
