# Supplementary material for: MicroRNA-Related Prognosis Biomarkers from High-Throughput Sequencing Data of Colorectal Cancer
Source: Biomed Res Int. 2020 Sep 9;2020:7905380. doi: 10.1155/2020/7905380 (PMC7501550; doi:10.1155/2020/7905380)
Supplement: Supplementary materials — Supplementary table 1: differentially expressed miRNAs for COAD. Supplementary table 2: differentially expressed miRNAs for READ. Supplementary table 3: differentially expressed mRNAs for COAD. Supplementary table 4: differentially expressed mRNAs for READ. Supplementary table 5: GO analysis of overlapping DEGs for COAD. Supplementary table 6: GO analysis of overlapping DEGs for READ. Supplementary table 7: KEGG analysis of overlapping DEGs for COAD. Supplementary table 8: KEGG analysis of overlapping DEGs for READ. [file 7905380.f1.docx]

**MicroRNA related prognosis biomarkers from high throughput sequencing data of colorectal cancer**

Xiao-liang Xing^1,2^, Zhi-Yong Yao^2^, Ti Zhang^2^, Ning Zhu^2^, Yuan-wu Liu^3*^, Jing Peng^1*^

^1^Xiangya Hospital, Central South University, Changsha, 410078, Hunan, P. R. China.

^2^Hunan Provincial Key Laboratory for Synthetic Biology of Traditional Chinese Medicine, School of public health and laboratory medicine, Hunan University of Medicine, Huaihua 418000, Hunan, P. R. China.

^3^Beijing Advanced Innovation Center for Food Nutrition and Human Health, China Agricultural University, 100193, Beijing, China.

*Correspondence: Yuan-wu Liu, yuanwu_liu@126.com; Jing Peng, [pengjing4346@163.com](mailto:pengjing4346@163.com)

**Supplementary information: 8 tables**

**Supplementary table 1 Differentially expressed miRNAs for COAD**

| Tag | baseMean | log2FoldChange | padj | Tag | baseMean | log2FoldChange | padj |
| --- | --- | --- | --- | --- | --- | --- | --- |
| MIMAT0000758 | 275.59 | 8.34 | 0.00 | MIMAT0000080 | 5451.01 | 1.88 | 0.00 |
| MIMAT0000073 | 178.44 | 8.07 | 0.00 | MIMAT0002809 | 1354.14 | 1.86 | 0.00 |
| MIMAT0004688 | 2738.82 | 7.57 | 0.00 | MIMAT0000071 | 1112.82 | 1.84 | 0.00 |
| MIMAT0003260 | 111.15 | 7.39 | 0.00 | MIMAT0000254 | 109711.01 | 1.83 | 0.00 |
| MIMAT0003242 | 327.44 | 7.28 | 0.00 | MIMAT0000281 | 233.12 | 1.81 | 0.00 |
| MIMAT0000433 | 256.19 | 6.87 | 0.00 | MIMAT0002821 | 55.15 | 1.69 | 0.00 |
| MIMAT0000074 | 655.31 | 6.68 | 0.00 | MIMAT0003249 | 935.62 | 1.68 | 0.00 |
| MIMAT0000090 | 81.98 | 6.59 | 0.00 | MIMAT0000691 | 63.20 | 1.67 | 0.00 |
| MIMAT0000434 | 7480.83 | 6.53 | 0.00 | MIMAT0000255 | 315.80 | 1.58 | 0.00 |
| MIMAT0004809 | 60.76 | 6.50 | 0.00 | MIMAT0000460 | 50883.47 | 1.55 | 0.00 |
| MIMAT0000765 | 54.46 | 6.35 | 0.00 | MIMAT0004946 | 63.65 | 1.55 | 0.00 |
| MIMAT0000072 | 85.57 | 6.29 | 0.00 | MIMAT0000757 | 6948.67 | 1.53 | 0.00 |
| MIMAT0000076 | 563990.02 | 6.13 | 0.00 | MIMAT0000449 | 651.49 | 1.48 | 0.00 |
| MIMAT0001341 | 397.38 | 6.05 | 0.00 | MIMAT0000077 | 130072.28 | 1.41 | 0.00 |
| MIMAT0003258 | 56.46 | 6.03 | 0.00 | MIMAT0000082 | 4629.00 | 1.36 | 0.00 |
| MIMAT0003389 | 436.07 | 5.82 | 0.00 | MIMAT0000441 | 1040.51 | 1.32 | 0.04 |
| MIMAT0004926 | 56.12 | 5.69 | 0.00 | MIMAT0000425 | 101.23 | 1.31 | 0.00 |
| MIMAT0000727 | 89.63 | 5.66 | 0.00 | MIMAT0004584 | 109.46 | 1.18 | 0.01 |
| MIMAT0019814 | 579.87 | 5.65 | 0.00 | MIMAT0000414 | 1622.17 | 1.10 | 0.00 |
| MIMAT0000444 | 461.70 | 5.62 | 0.00 | MIMAT0000085 | 304.49 | 1.02 | 0.00 |
| MIMAT0004493 | 68.51 | 5.61 | 0.00 | MIMAT0004808 | 1422.00 | -1.09 | 0.01 |
| MIMAT0000091 | 161.51 | 5.56 | 0.00 | MIMAT0002871 | 958.02 | -1.10 | 0.00 |
| MIMAT0000100 | 2320.25 | 5.54 | 0.00 | MIMAT0000088 | 3844.95 | -1.12 | 0.00 |
| MIMAT0026480 | 120.98 | 5.53 | 0.00 | MIMAT0004693 | 64.77 | -1.14 | 0.00 |
| MIMAT0026615 | 382.54 | 5.15 | 0.00 | MIMAT0004784 | 710.21 | -1.21 | 0.01 |
| MIMAT0004600 | 191.73 | 5.11 | 0.00 | MIMAT0000066 | 1608.65 | -1.21 | 0.00 |
| MIMAT0004797 | 1112.04 | 5.02 | 0.00 | MIMAT0000440 | 2017.03 | -1.22 | 0.00 |
| MIMAT0001536 | 1103.76 | 5.02 | 0.00 | MIMAT0022834 | 75.93 | -1.27 | 0.00 |
| MIMAT0000448 | 74.92 | 5.01 | 0.00 | MIMAT0000710 | 75.89 | -1.27 | 0.00 |
| MIMAT0000099 | 17955.38 | 4.94 | 0.00 | MIMAT0004673 | 50.36 | -1.29 | 0.00 |
| MIMAT0000432 | 3071.93 | 4.93 | 0.00 | MIMAT0000245 | 15095.96 | -1.29 | 0.00 |
| MIMAT0004927 | 103.84 | 4.91 | 0.00 | MIMAT0004502 | 14189.30 | -1.50 | 0.00 |
| MIMAT0004481 | 78.69 | 4.91 | 0.00 | MIMAT0004680 | 58.54 | -1.51 | 0.00 |
| MIMAT0000075 | 1465.21 | 4.71 | 0.00 | MIMAT0000257 | 518.83 | -1.56 | 0.00 |
| MIMAT0000416 | 149.29 | 4.67 | 0.00 | MIMAT0000062 | 56299.39 | -1.57 | 0.00 |
| MIMAT0000095 | 55.44 | 4.65 | 0.00 | MIMAT0001639 | 62.09 | -1.63 | 0.00 |
| MIMAT0000680 | 809.38 | 4.63 | 0.00 | MIMAT0004571 | 249.23 | -1.72 | 0.00 |
| MIMAT0004598 | 2039.35 | 4.62 | 0.00 | MIMAT0004672 | 848.48 | -1.73 | 0.00 |
| MIMAT0000259 | 46825.46 | 4.51 | 0.00 | MIMAT0000510 | 1264.59 | -1.79 | 0.00 |
| MIMAT0004703 | 571.33 | 4.37 | 0.00 | MIMAT0002876 | 132.49 | -1.82 | 0.00 |
| MIMAT0016895 | 87.93 | 4.34 | 0.00 | MIMAT0000753 | 203.88 | -1.91 | 0.00 |
| MIMAT0001635 | 304.68 | 4.32 | 0.00 | MIMAT0004774 | 157.65 | -2.03 | 0.00 |
| MIMAT0022727 | 1613.98 | 4.28 | 0.00 | MIMAT0000760 | 62.50 | -2.08 | 0.00 |
| MIMAT0000264 | 60763.53 | 4.13 | 0.00 | MIMAT0000446 | 1726.15 | -2.08 | 0.00 |
| MIMAT0000272 | 2781.23 | 4.13 | 0.00 | MIMAT0000617 | 35055.19 | -2.08 | 0.00 |
| MIMAT0000096 | 140.85 | 4.00 | 0.00 | MIMAT0003322 | 81.32 | -2.21 | 0.00 |
| MIMAT0000692 | 14911.96 | 3.97 | 0.00 | MIMAT0004748 | 131.43 | -2.26 | 0.00 |
| MIMAT0003215 | 178.10 | 3.97 | 0.00 | MIMAT0005899 | 134.36 | -2.27 | 0.00 |
| MIMAT0000733 | 2238.23 | 3.92 | 0.00 | MIMAT0000426 | 239.44 | -2.28 | 0.00 |
| MIMAT0000069 | 1925.53 | 3.83 | 0.00 | MIMAT0004597 | 1858.77 | -2.29 | 0.00 |
| MIMAT0004553 | 89.66 | 3.77 | 0.00 | MIMAT0004585 | 177.66 | -2.31 | 0.00 |
| MIMAT0003338 | 137.23 | 3.72 | 0.00 | MIMAT0000092 | 57002.41 | -2.37 | 0.00 |
| MIMAT0004497 | 74.86 | 3.71 | 0.00 | MIMAT0000764 | 97.27 | -2.43 | 0.00 |
| MIMAT0004955 | 195.31 | 3.65 | 0.00 | MIMAT0004799 | 154.37 | -2.64 | 0.00 |
| MIMAT0000263 | 282.03 | 3.62 | 0.00 | MIMAT0004678 | 92.56 | -2.72 | 0.00 |
| MIMAT0004586 | 102.71 | 3.58 | 0.00 | MIMAT0004482 | 65.85 | -2.76 | 0.00 |
| MIMAT0004604 | 670.10 | 3.58 | 0.00 | MIMAT0000064 | 1514.53 | -2.78 | 0.00 |
| MIMAT0000103 | 131.35 | 3.52 | 0.00 | MIMAT0000732 | 3892.83 | -2.79 | 0.00 |
| MIMAT0000274 | 107.87 | 3.45 | 0.00 | MIMAT0000417 | 542.00 | -2.82 | 0.00 |
| MIMAT0000070 | 2881.67 | 3.44 | 0.00 | MIMAT0002174 | 152.68 | -2.85 | 0.00 |
| MIMAT0000682 | 1722.82 | 3.39 | 0.00 | MIMAT0004911 | 57.04 | -2.87 | 0.00 |
| MIMAT0000243 | 191334.35 | 3.37 | 0.00 | MIMAT0000423 | 787.61 | -2.95 | 0.00 |
| MIMAT0000067 | 22634.28 | 3.37 | 0.00 | MIMAT0004682 | 270.01 | -3.12 | 0.00 |
| MIMAT0000445 | 5614.86 | 3.34 | 0.00 | MIMAT0002819 | 97.23 | -3.15 | 0.00 |
| MIMAT0000438 | 558.48 | 3.26 | 0.00 | MIMAT0000689 | 45295.03 | -3.23 | 0.00 |
| MIMAT0000068 | 347.08 | 3.12 | 0.00 | MIMAT0000728 | 89862.99 | -3.24 | 0.00 |
| MIMAT0000431 | 101.85 | 3.07 | 0.00 | MIMAT0003218 | 207.48 | -3.42 | 0.00 |
| MIMAT0000461 | 79.15 | 2.94 | 0.00 | MIMAT0005951 | 3437.03 | -3.42 | 0.00 |
| MIMAT0000420 | 938.45 | 2.93 | 0.00 | MIMAT0001340 | 286.03 | -3.52 | 0.00 |
| MIMAT0000253 | 218156.91 | 2.81 | 0.00 | MIMAT0004780 | 147.99 | -3.53 | 0.00 |
| MIMAT0000435 | 469585.72 | 2.74 | 0.00 | MIMAT0004614 | 446.23 | -3.55 | 0.00 |
| MIMAT0000083 | 1667.49 | 2.74 | 0.00 | MIMAT0000063 | 31622.97 | -3.57 | 0.00 |
| MIMAT0004563 | 7827.26 | 2.73 | 0.00 | MIMAT0004766 | 186.88 | -3.61 | 0.00 |
| MIMAT0000232 | 7838.46 | 2.73 | 0.00 | MIMAT0000256 | 2397.49 | -3.71 | 0.00 |
| MIMAT0000222 | 209935.89 | 2.66 | 0.00 | MIMAT0004945 | 57.70 | -3.72 | 0.00 |
| MIMAT0000226 | 786.01 | 2.65 | 0.00 | MIMAT0003239 | 191.83 | -3.86 | 0.00 |
| MIMAT0000681 | 2888.96 | 2.63 | 0.00 | MIMAT0000451 | 1511.92 | -3.94 | 0.00 |
| MIMAT0000754 | 68.71 | 2.61 | 0.00 | MIMAT0000437 | 9373.17 | -4.06 | 0.00 |
| MIMAT0000455 | 142.93 | 2.49 | 0.00 | MIMAT0000731 | 424.37 | -4.12 | 0.00 |
| MIMAT0000261 | 24293.85 | 2.47 | 0.00 | MIMAT0000427 | 94.63 | -4.12 | 0.00 |
| MIMAT0004495 | 64.54 | 2.47 | 0.00 | MIMAT0004484 | 1420.49 | -4.39 | 0.00 |
| MIMAT0000280 | 1033.23 | 2.44 | 0.00 | MIMAT0004671 | 874.31 | -4.42 | 0.00 |
| MIMAT0004494 | 9277.00 | 2.43 | 0.00 | MIMAT0000443 | 914.17 | -4.42 | 0.00 |
| MIMAT0000763 | 1418.36 | 2.42 | 0.00 | MIMAT0000227 | 1064.78 | -5.10 | 0.00 |
| MIMAT0001631 | 841.87 | 2.39 | 0.00 | MIMAT0000250 | 110.14 | -5.32 | 0.00 |
| MIMAT0001080 | 10099.89 | 2.13 | 0.00 | MIMAT0000752 | 77.24 | -5.69 | 0.00 |
| MIMAT0004501 | 83.03 | 1.92 | 0.00 | MIMAT0002177 | 423.61 | -5.93 | 0.00 |
| MIMAT0000084 | 4439.64 | 1.92 | 0.00 |  |  |  |  |

**Supplementary table 2 Differentially expressed miRNAs for READ**

| Tag | baseMean | log2FoldChange | padj | Tag | baseMean | log2FoldChange | padj |
| --- | --- | --- | --- | --- | --- | --- | --- |
| MIMAT0000758 | 415.91 | 8.91 | 0.00 | MIMAT0000754 | 81.35 | 2.73 | 0.00 |
| MIMAT0003242 | 354.12 | 8.67 | 0.00 | MIMAT0000420 | 1136.76 | 2.73 | 0.00 |
| MIMAT0026615 | 549.94 | 8.38 | 0.00 | MIMAT0000255 | 337.72 | 2.72 | 0.00 |
| MIMAT0000073 | 254.80 | 8.20 | 0.00 | MIMAT0000084 | 4519.64 | 2.69 | 0.00 |
| MIMAT0000091 | 195.70 | 7.82 | 0.00 | MIMAT0000071 | 1135.02 | 2.67 | 0.00 |
| MIMAT0001341 | 541.96 | 7.54 | 0.00 | MIMAT0000757 | 7721.72 | 2.55 | 0.00 |
| MIMAT0003260 | 132.77 | 7.26 | 0.00 | MIMAT0004563 | 8403.68 | 2.55 | 0.00 |
| MIMAT0000433 | 251.81 | 7.24 | 0.00 | MIMAT0000232 | 8417.68 | 2.55 | 0.00 |
| MIMAT0000072 | 121.22 | 7.13 | 0.00 | MIMAT0000080 | 6599.47 | 2.51 | 0.00 |
| MIMAT0000272 | 2921.55 | 7.07 | 0.00 | MIMAT0000763 | 1597.85 | 2.31 | 0.00 |
| MIMAT0003215 | 203.35 | 6.94 | 0.00 | MIMAT0000435 | 447628.80 | 2.23 | 0.00 |
| MIMAT0000074 | 823.80 | 6.77 | 0.00 | MIMAT0003393 | 1673.80 | 2.21 | 0.00 |
| MIMAT0001536 | 1318.24 | 6.74 | 0.00 | MIMAT0002821 | 74.54 | 2.20 | 0.01 |
| MIMAT0004493 | 86.23 | 6.64 | 0.00 | MIMAT0000414 | 2422.91 | 2.19 | 0.00 |
| MIMAT0001635 | 431.53 | 6.58 | 0.00 | MIMAT0004657 | 173.22 | 2.17 | 0.00 |
| MIMAT0000434 | 8553.69 | 6.55 | 0.00 | MIMAT0000318 | 7115.00 | 2.16 | 0.00 |
| MIMAT0000095 | 68.58 | 6.32 | 0.00 | MIMAT0000425 | 127.28 | 2.11 | 0.00 |
| MIMAT0004688 | 2966.56 | 6.30 | 0.00 | MIMAT0004549 | 210.71 | 2.03 | 0.00 |
| MIMAT0003258 | 67.53 | 6.27 | 0.00 | MIMAT0000077 | 138184.10 | 1.94 | 0.00 |
| MIMAT0004600 | 313.87 | 6.25 | 0.00 | MIMAT0000082 | 5555.61 | 1.91 | 0.00 |
| MIMAT0000076 | 652875.11 | 6.22 | 0.00 | MIMAT0000079 | 50.06 | 1.89 | 0.01 |
| MIMAT0004809 | 63.04 | 6.17 | 0.00 | MIMAT0000456 | 1038.17 | 1.87 | 0.00 |
| MIMAT0017990 | 60.96 | 6.12 | 0.00 | MIMAT0000646 | 944.76 | 1.86 | 0.00 |
| MIMAT0003150 | 57.51 | 6.05 | 0.00 | MIMAT0004494 | 8836.87 | 1.82 | 0.00 |
| MIMAT0000090 | 102.89 | 5.91 | 0.00 | MIMAT0000104 | 162.78 | 1.69 | 0.00 |
| MIMAT0000432 | 3921.53 | 5.89 | 0.00 | MIMAT0003249 | 917.37 | 1.63 | 0.03 |
| MIMAT0004921 | 50.82 | 5.87 | 0.00 | MIMAT0000736 | 130.13 | 1.60 | 0.00 |
| MIMAT0000100 | 2952.60 | 5.79 | 0.00 | MIMAT0000270 | 306.64 | 1.43 | 0.01 |
| MIMAT0004497 | 88.59 | 5.72 | 0.00 | MIMAT0000085 | 351.68 | 1.38 | 0.00 |
| MIMAT0000075 | 2130.61 | 5.59 | 0.00 | MIMAT0001620 | 5335.14 | 1.37 | 0.01 |
| MIMAT0019814 | 721.79 | 5.57 | 0.00 | MIMAT0000101 | 42155.85 | 1.34 | 0.01 |
| MIMAT0004926 | 74.62 | 5.46 | 0.00 | MIMAT0004543 | 432.68 | 1.28 | 0.01 |
| MIMAT0000765 | 72.18 | 5.44 | 0.00 | MIMAT0000086 | 24079.75 | 1.02 | 0.01 |
| MIMAT0004703 | 763.43 | 5.35 | 0.00 | MIMAT0001639 | 72.16 | -1.17 | 0.01 |
| MIMAT0000727 | 119.84 | 5.30 | 0.00 | MIMAT0000761 | 56.96 | -1.28 | 0.00 |
| MIMAT0003389 | 453.71 | 5.27 | 0.00 | MIMAT0000245 | 18265.04 | -1.29 | 0.00 |
| MIMAT0022727 | 1647.38 | 5.23 | 0.00 | MIMAT0004784 | 889.07 | -1.54 | 0.02 |
| MIMAT0000264 | 76371.26 | 5.18 | 0.00 | MIMAT0004558 | 1701.79 | -1.57 | 0.00 |
| MIMAT0000069 | 2354.21 | 5.13 | 0.00 | MIMAT0004774 | 188.27 | -1.57 | 0.01 |
| MIMAT0000067 | 38949.51 | 5.09 | 0.00 | MIMAT0000088 | 3317.37 | -1.59 | 0.00 |
| MIMAT0000252 | 57.96 | 5.09 | 0.00 | MIMAT0000426 | 246.64 | -1.68 | 0.00 |
| MIMAT0000680 | 935.44 | 5.08 | 0.00 | MIMAT0000753 | 219.54 | -1.76 | 0.00 |
| MIMAT0000448 | 97.57 | 5.05 | 0.00 | MIMAT0004748 | 155.15 | -1.77 | 0.00 |
| MIMAT0000444 | 557.42 | 4.98 | 0.00 | MIMAT0004673 | 61.41 | -1.82 | 0.00 |
| MIMAT0000431 | 127.96 | 4.97 | 0.00 | MIMAT0004502 | 15291.70 | -1.86 | 0.00 |
| MIMAT0004553 | 92.09 | 4.97 | 0.00 | MIMAT0004597 | 1869.78 | -2.15 | 0.00 |
| MIMAT0000416 | 236.77 | 4.96 | 0.00 | MIMAT0022834 | 85.22 | -2.18 | 0.00 |
| MIMAT0000222 | 251710.04 | 4.93 | 0.00 | MIMAT0000710 | 85.37 | -2.18 | 0.00 |
| MIMAT0000445 | 7348.61 | 4.83 | 0.00 | MIMAT0004672 | 959.91 | -2.19 | 0.00 |
| MIMAT0000259 | 53003.36 | 4.81 | 0.00 | MIMAT0000417 | 595.49 | -2.20 | 0.00 |
| MIMAT0000096 | 186.24 | 4.79 | 0.00 | MIMAT0000446 | 1736.90 | -2.24 | 0.00 |
| MIMAT0000243 | 241020.78 | 4.78 | 0.00 | MIMAT0004585 | 173.69 | -2.25 | 0.00 |
| MIMAT0004598 | 2022.48 | 4.72 | 0.00 | MIMAT0002876 | 155.81 | -2.28 | 0.00 |
| MIMAT0000682 | 2168.63 | 4.67 | 0.00 | MIMAT0000760 | 77.41 | -2.48 | 0.00 |
| MIMAT0000068 | 418.23 | 4.61 | 0.00 | MIMAT0005899 | 229.29 | -2.49 | 0.02 |
| MIMAT0000099 | 20007.33 | 4.57 | 0.00 | MIMAT0002174 | 184.77 | -2.51 | 0.00 |
| MIMAT0000226 | 1024.09 | 4.51 | 0.00 | MIMAT0000092 | 74775.34 | -2.52 | 0.00 |
| MIMAT0004955 | 245.02 | 4.49 | 0.00 | MIMAT0000764 | 112.38 | -2.54 | 0.00 |
| MIMAT0003338 | 166.08 | 4.48 | 0.00 | MIMAT0000732 | 4197.86 | -2.70 | 0.00 |
| MIMAT0004495 | 64.70 | 4.45 | 0.00 | MIMAT0004682 | 286.92 | -2.82 | 0.00 |
| MIMAT0000733 | 2137.84 | 4.41 | 0.00 | MIMAT0005951 | 3603.44 | -2.85 | 0.00 |
| MIMAT0004481 | 84.35 | 4.35 | 0.00 | MIMAT0004911 | 59.92 | -2.95 | 0.00 |
| MIMAT0000070 | 4143.80 | 4.30 | 0.00 | MIMAT0000064 | 2105.23 | -3.10 | 0.00 |
| MIMAT0000281 | 366.00 | 4.29 | 0.00 | MIMAT0000256 | 2986.56 | -3.12 | 0.00 |
| MIMAT0016895 | 108.12 | 4.28 | 0.00 | MIMAT0000728 | 88181.03 | -3.15 | 0.00 |
| MIMAT0000691 | 78.44 | 4.20 | 0.00 | MIMAT0004799 | 160.55 | -3.18 | 0.00 |
| MIMAT0026480 | 131.06 | 4.19 | 0.00 | MIMAT0004780 | 164.88 | -3.26 | 0.00 |
| MIMAT0000460 | 61468.41 | 4.05 | 0.00 | MIMAT0004766 | 170.98 | -3.29 | 0.00 |
| MIMAT0001080 | 12004.51 | 4.01 | 0.00 | MIMAT0001340 | 399.27 | -3.39 | 0.00 |
| MIMAT0000094 | 60.81 | 3.98 | 0.00 | MIMAT0004671 | 758.51 | -3.44 | 0.00 |
| MIMAT0000692 | 17304.55 | 3.90 | 0.00 | MIMAT0000762 | 51.99 | -3.68 | 0.00 |
| MIMAT0000274 | 129.71 | 3.80 | 0.00 | MIMAT0004678 | 111.95 | -3.70 | 0.00 |
| MIMAT0000083 | 2236.29 | 3.75 | 0.00 | MIMAT0004945 | 65.64 | -3.73 | 0.00 |
| MIMAT0000253 | 230994.40 | 3.69 | 0.00 | MIMAT0002819 | 154.75 | -3.74 | 0.00 |
| MIMAT0000275 | 53.09 | 3.41 | 0.00 | MIMAT0000063 | 36407.67 | -3.76 | 0.00 |
| MIMAT0000280 | 841.19 | 3.37 | 0.00 | MIMAT0000423 | 1046.47 | -3.81 | 0.00 |
| MIMAT0004586 | 109.79 | 3.32 | 0.00 | MIMAT0004482 | 80.03 | -3.81 | 0.00 |
| MIMAT0000261 | 25626.51 | 3.30 | 0.00 | MIMAT0003218 | 291.30 | -4.32 | 0.00 |
| MIMAT0002809 | 1458.56 | 3.29 | 0.00 | MIMAT0000689 | 56236.72 | -4.36 | 0.00 |
| MIMAT0004797 | 968.28 | 3.24 | 0.00 | MIMAT0000731 | 481.81 | -4.43 | 0.00 |
| MIMAT0000461 | 85.58 | 3.18 | 0.00 | MIMAT0000451 | 1547.69 | -4.48 | 0.00 |
| MIMAT0000263 | 347.02 | 3.12 | 0.00 | MIMAT0000437 | 14743.43 | -5.05 | 0.00 |
| MIMAT0001631 | 1192.25 | 3.04 | 0.00 | MIMAT0000443 | 1477.38 | -5.21 | 0.00 |
| MIMAT0000438 | 629.25 | 2.95 | 0.00 | MIMAT0004614 | 610.59 | -5.28 | 0.00 |
| MIMAT0000681 | 2415.87 | 2.90 | 0.00 | MIMAT0000427 | 156.54 | -5.62 | 0.00 |
| MIMAT0004927 | 107.75 | 2.90 | 0.00 | MIMAT0003239 | 315.05 | -5.68 | 0.00 |
| MIMAT0000254 | 71814.55 | 2.87 | 0.00 | MIMAT0004484 | 2150.24 | -5.69 | 0.00 |
| MIMAT0000103 | 210.06 | 2.85 | 0.03 | MIMAT0000227 | 1576.47 | -5.75 | 0.00 |
| MIMAT0017985 | 475.80 | 2.81 | 0.00 | MIMAT0002177 | 511.08 | -5.91 | 0.00 |
| MIMAT0004604 | 728.26 | 2.79 | 0.00 | MIMAT0000450 | 77.99 | -5.99 | 0.00 |
| MIMAT0000449 | 757.14 | 2.77 | 0.00 | MIMAT0000250 | 192.09 | -6.40 | 0.00 |
| MIMAT0000455 | 163.30 | 2.74 | 0.00 | MIMAT0000752 | 230.73 | -7.64 | 0.00 |

**Supplementary table 3 Differentially expressed mRNAs for COAD**

| Tag | baseMean | log2FoldChange | padj | Tag | baseMean | log2FoldChange | padj |
| --- | --- | --- | --- | --- | --- | --- | --- |
| ENSG00000221867 | 99.95 | 10.79 | 0.00 | ENSG00000170632 | 1582.44 | 1.03 | 0.00 |
| ENSG00000197172 | 83.41 | 9.63 | 0.00 | ENSG00000123374 | 1395.69 | 1.03 | 0.00 |
| ENSG00000167755 | 908.82 | 9.50 | 0.00 | ENSG00000115268 | 18636.47 | 1.02 | 0.00 |
| ENSG00000185269 | 2634.17 | 9.48 | 0.00 | ENSG00000213462 | 333.55 | 1.02 | 0.00 |
| ENSG00000230316 | 159.56 | 9.24 | 0.00 | ENSG00000111344 | 545.98 | 1.02 | 0.00 |
| ENSG00000214039 | 603.73 | 9.16 | 0.00 | ENSG00000101442 | 588.83 | 1.02 | 0.00 |
| ENSG00000129455 | 99.21 | 8.91 | 0.00 | ENSG00000081181 | 166.47 | 1.02 | 0.00 |
| ENSG00000128610 | 83.39 | 8.59 | 0.00 | ENSG00000118939 | 598.93 | 1.02 | 0.00 |
| ENSG00000139800 | 98.82 | 8.55 | 0.00 | ENSG00000147383 | 1514.51 | 1.02 | 0.00 |
| ENSG00000276122 | 60.15 | 8.54 | 0.00 | ENSG00000130299 | 817.68 | 1.02 | 0.00 |
| ENSG00000170373 | 977.72 | 8.48 | 0.00 | ENSG00000001497 | 2129.03 | 1.02 | 0.00 |
| ENSG00000169035 | 200.02 | 8.37 | 0.00 | ENSG00000040275 | 489.05 | 1.02 | 0.00 |
| ENSG00000135374 | 111.15 | 8.05 | 0.00 | ENSG00000135476 | 990.24 | 1.02 | 0.00 |
| ENSG00000196260 | 111.89 | 8.00 | 0.00 | ENSG00000260005 | 52.14 | 1.02 | 0.00 |
| ENSG00000123500 | 858.56 | 7.85 | 0.00 | ENSG00000165752 | 423.96 | 1.02 | 0.00 |
| ENSG00000249550 | 140.55 | 7.74 | 0.00 | ENSG00000147874 | 820.09 | 1.02 | 0.00 |
| ENSG00000224099 | 120.30 | 7.69 | 0.00 | ENSG00000171316 | 2096.92 | 1.02 | 0.00 |
| ENSG00000198842 | 1555.99 | 7.59 | 0.00 | ENSG00000249353 | 130.35 | 1.02 | 0.00 |
| ENSG00000104371 | 91.30 | 7.54 | 0.00 | ENSG00000100401 | 5094.79 | 1.02 | 0.00 |
| ENSG00000168875 | 57.15 | 7.53 | 0.00 | ENSG00000123219 | 415.13 | 1.02 | 0.00 |
| ENSG00000204542 | 86.53 | 7.34 | 0.00 | ENSG00000103037 | 541.73 | 1.02 | 0.00 |
| ENSG00000170454 | 59.09 | 7.31 | 0.00 | ENSG00000173207 | 1037.30 | 1.02 | 0.00 |
| ENSG00000134827 | 839.24 | 7.27 | 0.00 | ENSG00000145293 | 1960.82 | 1.02 | 0.00 |
| ENSG00000250920 | 50.35 | 7.27 | 0.00 | ENSG00000164038 | 277.73 | 1.02 | 0.00 |
| ENSG00000137673 | 2455.79 | 7.18 | 0.00 | ENSG00000105655 | 704.27 | 1.02 | 0.00 |
| ENSG00000108244 | 2221.60 | 7.16 | 0.00 | ENSG00000148773 | 6159.39 | 1.01 | 0.00 |
| ENSG00000233532 | 65.08 | 7.12 | 0.00 | ENSG00000186638 | 295.19 | 1.01 | 0.00 |
| ENSG00000251164 | 159.60 | 7.02 | 0.00 | ENSG00000165730 | 197.27 | 1.01 | 0.00 |
| ENSG00000249306 | 63.56 | 6.88 | 0.00 | ENSG00000007376 | 1565.11 | 1.01 | 0.00 |
| ENSG00000167767 | 1413.10 | 6.79 | 0.00 | ENSG00000148840 | 2241.60 | 1.01 | 0.00 |
| ENSG00000253929 | 63.79 | 6.73 | 0.00 | ENSG00000151849 | 574.55 | 1.01 | 0.00 |
| ENSG00000228742 | 56.96 | 6.71 | 0.00 | ENSG00000103222 | 2973.04 | 1.01 | 0.00 |
| ENSG00000205922 | 125.00 | 6.71 | 0.00 | ENSG00000142207 | 1843.39 | 1.01 | 0.00 |
| ENSG00000251095 | 77.65 | 6.53 | 0.00 | ENSG00000148362 | 1378.50 | 1.01 | 0.00 |
| ENSG00000060718 | 1354.68 | 6.48 | 0.00 | ENSG00000169783 | 204.86 | 1.01 | 0.00 |
| ENSG00000111700 | 143.24 | 6.43 | 0.00 | ENSG00000122547 | 1274.02 | 1.01 | 0.00 |
| ENSG00000185247 | 56.71 | 6.35 | 0.00 | ENSG00000053372 | 2146.77 | 1.01 | 0.00 |
| ENSG00000170369 | 61.40 | 6.34 | 0.00 | ENSG00000236699 | 386.62 | 1.01 | 0.00 |
| ENSG00000148702 | 72.87 | 6.33 | 0.00 | ENSG00000177464 | 194.07 | 1.01 | 0.00 |
| ENSG00000164778 | 68.53 | 6.29 | 0.00 | ENSG00000182010 | 290.51 | 1.01 | 0.00 |
| ENSG00000162344 | 92.63 | 6.29 | 0.00 | ENSG00000203880 | 2226.38 | 1.01 | 0.00 |
| ENSG00000164379 | 1464.88 | 6.28 | 0.00 | ENSG00000173113 | 3924.12 | 1.01 | 0.00 |
| ENSG00000163736 | 309.00 | 6.23 | 0.00 | ENSG00000130511 | 2002.10 | 1.01 | 0.00 |
| ENSG00000229404 | 52.73 | 6.15 | 0.00 | ENSG00000244398 | 440.96 | 1.01 | 0.00 |
| ENSG00000186007 | 137.59 | 6.13 | 0.00 | ENSG00000136891 | 1187.84 | 1.01 | 0.00 |
| ENSG00000066405 | 423.30 | 6.11 | 0.00 | ENSG00000221968 | 514.81 | 1.01 | 0.00 |
| ENSG00000043355 | 232.41 | 6.07 | 0.00 | ENSG00000053918 | 5342.09 | 1.01 | 0.00 |
| ENSG00000105664 | 752.13 | 6.03 | 0.00 | ENSG00000109501 | 2903.08 | 1.01 | 0.00 |
| ENSG00000062038 | 3353.95 | 5.99 | 0.00 | ENSG00000261068 | 83.09 | 1.01 | 0.00 |
| ENSG00000170835 | 1387.61 | 5.95 | 0.00 | ENSG00000085840 | 553.00 | 1.01 | 0.00 |
| ENSG00000272620 | 430.77 | 5.90 | 0.00 | ENSG00000102393 | 1210.60 | 1.01 | 0.00 |
| ENSG00000185479 | 466.06 | 5.85 | 0.00 | ENSG00000154429 | 417.40 | 1.01 | 0.00 |
| ENSG00000275216 | 293.30 | 5.80 | 0.00 | ENSG00000027869 | 281.38 | 1.01 | 0.00 |
| ENSG00000115507 | 104.92 | 5.77 | 0.00 | ENSG00000234420 | 420.84 | 1.01 | 0.00 |
| ENSG00000164283 | 237.34 | 5.74 | 0.00 | ENSG00000188211 | 563.47 | 1.01 | 0.00 |
| ENSG00000250641 | 314.45 | 5.74 | 0.00 | ENSG00000130589 | 3437.71 | 1.01 | 0.00 |
| ENSG00000178773 | 1094.57 | 5.72 | 0.00 | ENSG00000120158 | 525.54 | 1.00 | 0.00 |
| ENSG00000188064 | 51.28 | 5.71 | 0.00 | ENSG00000184967 | 1258.89 | 1.00 | 0.00 |
| ENSG00000213468 | 55.21 | 5.71 | 0.00 | ENSG00000135127 | 549.02 | 1.00 | 0.00 |
| ENSG00000137868 | 435.81 | 5.71 | 0.00 | ENSG00000156795 | 425.07 | 1.00 | 0.00 |
| ENSG00000204869 | 81.09 | 5.70 | 0.00 | ENSG00000180921 | 7041.15 | 1.00 | 0.00 |
| ENSG00000107159 | 1817.07 | 5.67 | 0.00 | ENSG00000106009 | 2757.53 | 1.00 | 0.00 |
| ENSG00000259485 | 79.42 | 5.62 | 0.00 | ENSG00000171049 | 65.76 | 1.00 | 0.00 |
| ENSG00000187258 | 193.31 | 5.59 | 0.00 | ENSG00000156787 | 389.78 | 1.00 | 0.00 |
| ENSG00000182379 | 245.68 | 5.53 | 0.00 | ENSG00000158859 | 734.81 | 1.00 | 0.00 |
| ENSG00000244355 | 315.04 | 5.53 | 0.00 | ENSG00000130208 | 675.26 | 1.00 | 0.00 |
| ENSG00000128683 | 127.05 | 5.52 | 0.00 | ENSG00000187678 | 1863.32 | 1.00 | 0.00 |
| ENSG00000171759 | 128.37 | 5.50 | 0.00 | ENSG00000187720 | 759.29 | -1.00 | 0.00 |
| ENSG00000177238 | 175.88 | 5.48 | 0.00 | ENSG00000156345 | 218.11 | -1.00 | 0.00 |
| ENSG00000105989 | 305.00 | 5.48 | 0.00 | ENSG00000156052 | 2415.20 | -1.00 | 0.00 |
| ENSG00000205420 | 141.21 | 5.42 | 0.00 | ENSG00000233006 | 77.61 | -1.00 | 0.00 |
| ENSG00000165376 | 5984.27 | 5.42 | 0.00 | ENSG00000074527 | 596.32 | -1.00 | 0.00 |
| ENSG00000142700 | 62.50 | 5.41 | 0.00 | ENSG00000276566 | 65.34 | -1.00 | 0.03 |
| ENSG00000161798 | 117.63 | 5.41 | 0.00 | ENSG00000160471 | 88.28 | -1.00 | 0.00 |
| ENSG00000137745 | 70.34 | 5.41 | 0.00 | ENSG00000211662 | 1987.99 | -1.00 | 0.01 |
| ENSG00000226532 | 52.35 | 5.37 | 0.00 | ENSG00000137171 | 2421.78 | -1.00 | 0.00 |
| ENSG00000182798 | 83.56 | 5.35 | 0.00 | ENSG00000172270 | 30688.54 | -1.00 | 0.00 |
| ENSG00000175832 | 3941.71 | 5.35 | 0.00 | ENSG00000149577 | 1590.31 | -1.00 | 0.00 |
| ENSG00000281406 | 187.01 | 5.33 | 0.00 | ENSG00000101916 | 86.93 | -1.00 | 0.00 |
| ENSG00000122641 | 1269.50 | 5.30 | 0.00 | ENSG00000186188 | 716.73 | -1.00 | 0.00 |
| ENSG00000234985 | 85.28 | 5.27 | 0.00 | ENSG00000171608 | 372.40 | -1.00 | 0.00 |
| ENSG00000015413 | 9045.95 | 5.24 | 0.00 | ENSG00000185567 | 1278.20 | -1.00 | 0.00 |
| ENSG00000103888 | 4966.20 | 5.24 | 0.00 | ENSG00000100221 | 3018.64 | -1.01 | 0.00 |
| ENSG00000134757 | 421.92 | 5.23 | 0.00 | ENSG00000126785 | 239.42 | -1.01 | 0.00 |
| ENSG00000140835 | 126.93 | 5.20 | 0.00 | ENSG00000162852 | 870.01 | -1.01 | 0.00 |
| ENSG00000275896 | 585.10 | 5.20 | 0.00 | ENSG00000135002 | 1950.44 | -1.01 | 0.00 |
| ENSG00000129451 | 1874.15 | 5.16 | 0.00 | ENSG00000186496 | 59.25 | -1.01 | 0.00 |
| ENSG00000184956 | 236.73 | 5.14 | 0.00 | ENSG00000187942 | 50.98 | -1.01 | 0.00 |
| ENSG00000163735 | 870.71 | 5.09 | 0.00 | ENSG00000082146 | 640.29 | -1.01 | 0.00 |
| ENSG00000125207 | 286.85 | 5.04 | 0.00 | ENSG00000223547 | 366.65 | -1.01 | 0.00 |
| ENSG00000236081 | 295.43 | 5.01 | 0.00 | ENSG00000187601 | 253.92 | -1.01 | 0.00 |
| ENSG00000128422 | 908.89 | 4.98 | 0.00 | ENSG00000143409 | 897.86 | -1.01 | 0.00 |
| ENSG00000123388 | 58.79 | 4.94 | 0.00 | ENSG00000162144 | 1361.70 | -1.01 | 0.00 |
| ENSG00000105219 | 198.87 | 4.93 | 0.00 | ENSG00000129493 | 801.50 | -1.01 | 0.00 |
| ENSG00000186832 | 60.23 | 4.90 | 0.00 | ENSG00000138764 | 1230.95 | -1.01 | 0.00 |
| ENSG00000129991 | 59.26 | 4.89 | 0.00 | ENSG00000141441 | 566.28 | -1.01 | 0.00 |
| ENSG00000105464 | 1388.44 | 4.88 | 0.00 | ENSG00000170677 | 1185.15 | -1.01 | 0.00 |
| ENSG00000172016 | 3310.34 | 4.87 | 0.00 | ENSG00000138029 | 4015.35 | -1.01 | 0.00 |
| ENSG00000181577 | 1293.06 | 4.86 | 0.00 | ENSG00000179909 | 57.95 | -1.01 | 0.00 |
| ENSG00000184292 | 1451.69 | 4.84 | 0.00 | ENSG00000012223 | 202.29 | -1.01 | 0.00 |
| ENSG00000120149 | 510.85 | 4.84 | 0.00 | ENSG00000166206 | 73.44 | -1.01 | 0.02 |
| ENSG00000254560 | 90.61 | 4.82 | 0.00 | ENSG00000155744 | 669.63 | -1.01 | 0.00 |
| ENSG00000225616 | 117.59 | 4.81 | 0.00 | ENSG00000170647 | 152.63 | -1.01 | 0.00 |
| ENSG00000163347 | 3266.96 | 4.81 | 0.00 | ENSG00000141506 | 199.65 | -1.02 | 0.00 |
| ENSG00000184937 | 68.17 | 4.79 | 0.00 | ENSG00000182022 | 588.47 | -1.02 | 0.00 |
| ENSG00000101115 | 124.65 | 4.79 | 0.00 | ENSG00000116667 | 1878.36 | -1.02 | 0.00 |
| ENSG00000245694 | 142.19 | 4.76 | 0.00 | ENSG00000181007 | 53.58 | -1.02 | 0.00 |
| ENSG00000139515 | 639.73 | 4.76 | 0.00 | ENSG00000074706 | 59.24 | -1.02 | 0.00 |
| ENSG00000215182 | 1605.63 | 4.75 | 0.00 | ENSG00000163362 | 2582.30 | -1.02 | 0.00 |
| ENSG00000167244 | 26824.84 | 4.73 | 0.00 | ENSG00000137449 | 578.57 | -1.02 | 0.00 |
| ENSG00000182968 | 69.39 | 4.72 | 0.00 | ENSG00000095321 | 2473.22 | -1.02 | 0.00 |
| ENSG00000136574 | 72.94 | 4.71 | 0.00 | ENSG00000113532 | 245.87 | -1.02 | 0.00 |
| ENSG00000149968 | 1574.47 | 4.70 | 0.00 | ENSG00000105251 | 184.04 | -1.02 | 0.00 |
| ENSG00000181418 | 116.24 | 4.68 | 0.00 | ENSG00000076513 | 1463.40 | -1.02 | 0.00 |
| ENSG00000280109 | 92.76 | 4.66 | 0.00 | ENSG00000139044 | 2703.56 | -1.02 | 0.00 |
| ENSG00000273132 | 56.40 | 4.66 | 0.00 | ENSG00000152527 | 133.41 | -1.02 | 0.00 |
| ENSG00000088992 | 2017.75 | 4.65 | 0.00 | ENSG00000106829 | 472.73 | -1.02 | 0.00 |
| ENSG00000237686 | 63.70 | 4.62 | 0.00 | ENSG00000171596 | 52.65 | -1.02 | 0.00 |
| ENSG00000247844 | 382.48 | 4.62 | 0.00 | ENSG00000119541 | 2222.28 | -1.02 | 0.00 |
| ENSG00000266402 | 217.54 | 4.59 | 0.00 | ENSG00000131069 | 5805.27 | -1.03 | 0.00 |
| ENSG00000248810 | 67.65 | 4.59 | 0.00 | ENSG00000175164 | 2704.15 | -1.03 | 0.00 |
| ENSG00000172031 | 261.68 | 4.58 | 0.00 | ENSG00000163171 | 919.92 | -1.03 | 0.00 |
| ENSG00000168065 | 97.16 | 4.55 | 0.00 | ENSG00000197471 | 315.22 | -1.03 | 0.00 |
| ENSG00000189280 | 159.66 | 4.54 | 0.00 | ENSG00000102882 | 5228.58 | -1.03 | 0.00 |
| ENSG00000213293 | 74.43 | 4.54 | 0.00 | ENSG00000248771 | 587.10 | -1.03 | 0.00 |
| ENSG00000212993 | 270.21 | 4.54 | 0.00 | ENSG00000117114 | 371.45 | -1.03 | 0.00 |
| ENSG00000165905 | 1435.66 | 4.51 | 0.00 | ENSG00000082074 | 399.14 | -1.03 | 0.00 |
| ENSG00000095752 | 156.38 | 4.51 | 0.00 | ENSG00000112245 | 7488.15 | -1.03 | 0.00 |
| ENSG00000238178 | 118.34 | 4.47 | 0.00 | ENSG00000211973 | 222.92 | -1.03 | 0.01 |
| ENSG00000159217 | 132.78 | 4.45 | 0.00 | ENSG00000224520 | 108.54 | -1.03 | 0.00 |
| ENSG00000170231 | 355.96 | 4.44 | 0.00 | ENSG00000111859 | 2402.99 | -1.03 | 0.00 |
| ENSG00000159263 | 506.38 | 4.44 | 0.00 | ENSG00000172086 | 1455.48 | -1.03 | 0.00 |
| ENSG00000237643 | 433.34 | 4.44 | 0.00 | ENSG00000266412 | 6283.97 | -1.03 | 0.00 |
| ENSG00000179546 | 325.94 | 4.42 | 0.00 | ENSG00000010610 | 1256.46 | -1.03 | 0.00 |
| ENSG00000196611 | 3909.17 | 4.41 | 0.00 | ENSG00000177707 | 886.22 | -1.03 | 0.00 |
| ENSG00000137699 | 3148.15 | 4.41 | 0.00 | ENSG00000186517 | 567.96 | -1.04 | 0.00 |
| ENSG00000103355 | 248.77 | 4.39 | 0.00 | ENSG00000031003 | 636.93 | -1.04 | 0.00 |
| ENSG00000110195 | 206.46 | 4.38 | 0.00 | ENSG00000174125 | 124.47 | -1.04 | 0.00 |
| ENSG00000099953 | 4125.67 | 4.37 | 0.00 | ENSG00000117758 | 1638.23 | -1.04 | 0.00 |
| ENSG00000100078 | 86.16 | 4.37 | 0.00 | ENSG00000167644 | 1658.51 | -1.04 | 0.00 |
| ENSG00000181085 | 233.05 | 4.36 | 0.00 | ENSG00000184371 | 870.71 | -1.04 | 0.00 |
| ENSG00000182747 | 225.26 | 4.35 | 0.00 | ENSG00000108352 | 3395.37 | -1.04 | 0.00 |
| ENSG00000105048 | 156.96 | 4.34 | 0.00 | ENSG00000008516 | 130.60 | -1.04 | 0.00 |
| ENSG00000131015 | 75.57 | 4.34 | 0.00 | ENSG00000174080 | 587.95 | -1.04 | 0.00 |
| ENSG00000198535 | 931.05 | 4.33 | 0.00 | ENSG00000072415 | 1193.73 | -1.04 | 0.00 |
| ENSG00000142619 | 79.86 | 4.32 | 0.00 | ENSG00000102984 | 83.01 | -1.04 | 0.00 |
| ENSG00000145506 | 859.27 | 4.31 | 0.00 | ENSG00000009790 | 141.37 | -1.04 | 0.00 |
| ENSG00000164398 | 787.27 | 4.28 | 0.00 | ENSG00000123094 | 245.35 | -1.04 | 0.00 |
| ENSG00000042832 | 357.58 | 4.26 | 0.00 | ENSG00000108984 | 810.10 | -1.04 | 0.00 |
| ENSG00000231764 | 52.68 | 4.26 | 0.00 | ENSG00000159164 | 107.83 | -1.04 | 0.00 |
| ENSG00000104899 | 142.95 | 4.24 | 0.00 | ENSG00000120129 | 4020.65 | -1.04 | 0.00 |
| ENSG00000162009 | 63.46 | 4.23 | 0.00 | ENSG00000112033 | 2225.41 | -1.04 | 0.00 |
| ENSG00000158296 | 302.78 | 4.23 | 0.00 | ENSG00000120137 | 2835.54 | -1.04 | 0.00 |
| ENSG00000073067 | 1959.47 | 4.21 | 0.00 | ENSG00000186350 | 2928.29 | -1.04 | 0.00 |
| ENSG00000134762 | 470.00 | 4.19 | 0.00 | ENSG00000118257 | 1220.50 | -1.05 | 0.00 |
| ENSG00000196754 | 327.65 | 4.16 | 0.00 | ENSG00000100242 | 3957.08 | -1.05 | 0.00 |
| ENSG00000131142 | 54.17 | 4.16 | 0.00 | ENSG00000138771 | 4747.42 | -1.05 | 0.00 |
| ENSG00000112761 | 81.55 | 4.15 | 0.00 | ENSG00000015285 | 261.11 | -1.05 | 0.00 |
| ENSG00000140807 | 3186.10 | 4.14 | 0.00 | ENSG00000167748 | 1846.24 | -1.05 | 0.00 |
| ENSG00000232237 | 56.73 | 4.13 | 0.00 | ENSG00000072694 | 136.27 | -1.05 | 0.00 |
| ENSG00000174171 | 77.14 | 4.12 | 0.00 | ENSG00000196569 | 491.17 | -1.05 | 0.00 |
| ENSG00000148848 | 772.70 | 4.11 | 0.00 | ENSG00000163154 | 92.78 | -1.05 | 0.00 |
| ENSG00000240204 | 61.39 | 4.11 | 0.00 | ENSG00000140479 | 779.81 | -1.05 | 0.00 |
| ENSG00000005001 | 864.87 | 4.10 | 0.00 | ENSG00000230487 | 142.21 | -1.05 | 0.00 |
| ENSG00000088836 | 306.56 | 4.09 | 0.00 | ENSG00000140497 | 5933.46 | -1.05 | 0.00 |
| ENSG00000189433 | 145.55 | 4.06 | 0.00 | ENSG00000204314 | 83.37 | -1.05 | 0.00 |
| ENSG00000183248 | 293.02 | 4.04 | 0.00 | ENSG00000213762 | 242.38 | -1.05 | 0.00 |
| ENSG00000144452 | 77.40 | 4.02 | 0.00 | ENSG00000117020 | 434.20 | -1.05 | 0.00 |
| ENSG00000165816 | 823.00 | 4.01 | 0.00 | ENSG00000123095 | 696.21 | -1.05 | 0.00 |
| ENSG00000182352 | 88.59 | 4.00 | 0.00 | ENSG00000169508 | 251.94 | -1.05 | 0.00 |
| ENSG00000149243 | 257.73 | 4.00 | 0.00 | ENSG00000188060 | 71.74 | -1.06 | 0.00 |
| ENSG00000136231 | 141.60 | 4.00 | 0.00 | ENSG00000198851 | 414.70 | -1.06 | 0.00 |
| ENSG00000214049 | 940.78 | 4.00 | 0.00 | ENSG00000116209 | 10525.78 | -1.06 | 0.00 |
| ENSG00000100867 | 103.79 | 3.97 | 0.00 | ENSG00000144045 | 406.44 | -1.06 | 0.00 |
| ENSG00000015520 | 214.76 | 3.96 | 0.00 | ENSG00000171291 | 120.11 | -1.06 | 0.00 |
| ENSG00000107807 | 130.19 | 3.96 | 0.00 | ENSG00000100092 | 1855.96 | -1.06 | 0.00 |
| ENSG00000118785 | 5502.88 | 3.95 | 0.00 | ENSG00000176903 | 862.87 | -1.06 | 0.00 |
| ENSG00000112494 | 159.76 | 3.95 | 0.00 | ENSG00000224272 | 256.32 | -1.06 | 0.00 |
| ENSG00000081277 | 433.72 | 3.93 | 0.00 | ENSG00000130821 | 4832.39 | -1.06 | 0.00 |
| ENSG00000183734 | 3944.93 | 3.93 | 0.00 | ENSG00000113924 | 380.30 | -1.06 | 0.00 |
| ENSG00000187556 | 70.99 | 3.90 | 0.00 | ENSG00000160325 | 1748.22 | -1.06 | 0.00 |
| ENSG00000251493 | 89.72 | 3.89 | 0.00 | ENSG00000166710 | 90366.55 | -1.06 | 0.00 |
| ENSG00000226476 | 68.67 | 3.89 | 0.00 | ENSG00000166689 | 1408.14 | -1.06 | 0.00 |
| ENSG00000242814 | 92.22 | 3.88 | 0.00 | ENSG00000138593 | 1038.55 | -1.06 | 0.00 |
| ENSG00000274979 | 91.46 | 3.88 | 0.00 | ENSG00000142949 | 15883.09 | -1.06 | 0.00 |
| ENSG00000214612 | 68.61 | 3.88 | 0.00 | ENSG00000127334 | 2501.63 | -1.06 | 0.00 |
| ENSG00000240668 | 431.75 | 3.87 | 0.00 | ENSG00000138641 | 815.40 | -1.06 | 0.00 |
| ENSG00000101255 | 2885.22 | 3.86 | 0.00 | ENSG00000079263 | 106.96 | -1.06 | 0.00 |
| ENSG00000164932 | 1040.71 | 3.82 | 0.00 | ENSG00000112320 | 162.59 | -1.06 | 0.00 |
| ENSG00000115363 | 325.29 | 3.82 | 0.00 | ENSG00000223572 | 537.52 | -1.06 | 0.00 |
| ENSG00000213613 | 50.70 | 3.81 | 0.00 | ENSG00000035403 | 6359.15 | -1.07 | 0.00 |
| ENSG00000158055 | 175.94 | 3.78 | 0.00 | ENSG00000272644 | 59.33 | -1.07 | 0.00 |
| ENSG00000196335 | 117.61 | 3.77 | 0.00 | ENSG00000114023 | 2623.30 | -1.07 | 0.00 |
| ENSG00000206069 | 134.13 | 3.74 | 0.00 | ENSG00000131171 | 2534.83 | -1.07 | 0.00 |
| ENSG00000204866 | 111.39 | 3.74 | 0.00 | ENSG00000101082 | 75.03 | -1.07 | 0.00 |
| ENSG00000210144 | 545.46 | 3.73 | 0.00 | ENSG00000095917 | 52.75 | -1.07 | 0.01 |
| ENSG00000231826 | 304.41 | 3.72 | 0.00 | ENSG00000105650 | 466.95 | -1.07 | 0.00 |
| ENSG00000134812 | 80.02 | 3.70 | 0.00 | ENSG00000146192 | 226.92 | -1.07 | 0.00 |
| ENSG00000204335 | 497.90 | 3.70 | 0.00 | ENSG00000070961 | 2123.68 | -1.07 | 0.00 |
| ENSG00000125657 | 347.69 | 3.70 | 0.00 | ENSG00000086015 | 3269.44 | -1.07 | 0.00 |
| ENSG00000240950 | 111.57 | 3.70 | 0.00 | ENSG00000075240 | 2288.56 | -1.07 | 0.00 |
| ENSG00000255026 | 216.13 | 3.69 | 0.00 | ENSG00000172197 | 563.11 | -1.07 | 0.00 |
| ENSG00000232803 | 364.79 | 3.69 | 0.00 | ENSG00000172296 | 221.49 | -1.07 | 0.00 |
| ENSG00000130600 | 1567.02 | 3.67 | 0.00 | ENSG00000144935 | 68.47 | -1.07 | 0.00 |
| ENSG00000273604 | 493.18 | 3.67 | 0.00 | ENSG00000164035 | 179.66 | -1.07 | 0.00 |
| ENSG00000238133 | 76.94 | 3.65 | 0.00 | ENSG00000100351 | 97.84 | -1.07 | 0.00 |
| ENSG00000196711 | 61.16 | 3.65 | 0.00 | ENSG00000044524 | 125.23 | -1.07 | 0.00 |
| ENSG00000112559 | 513.96 | 3.64 | 0.00 | ENSG00000137070 | 227.01 | -1.07 | 0.00 |
| ENSG00000196917 | 78.66 | 3.64 | 0.00 | ENSG00000188227 | 75.86 | -1.08 | 0.00 |
| ENSG00000162004 | 125.39 | 3.64 | 0.00 | ENSG00000127920 | 422.72 | -1.08 | 0.00 |
| ENSG00000262188 | 100.43 | 3.63 | 0.00 | ENSG00000154889 | 448.41 | -1.08 | 0.00 |
| ENSG00000099937 | 50.11 | 3.62 | 0.00 | ENSG00000050628 | 140.87 | -1.08 | 0.00 |
| ENSG00000126583 | 81.48 | 3.62 | 0.00 | ENSG00000183049 | 1441.36 | -1.08 | 0.00 |
| ENSG00000103253 | 424.21 | 3.62 | 0.00 | ENSG00000163346 | 3012.10 | -1.08 | 0.00 |
| ENSG00000173894 | 552.14 | 3.61 | 0.00 | ENSG00000187546 | 81.28 | -1.08 | 0.00 |
| ENSG00000206195 | 56.97 | 3.61 | 0.00 | ENSG00000005882 | 1072.12 | -1.08 | 0.00 |
| ENSG00000113739 | 908.11 | 3.60 | 0.00 | ENSG00000171016 | 72.51 | -1.08 | 0.00 |
| ENSG00000237721 | 86.46 | 3.60 | 0.00 | ENSG00000241106 | 64.22 | -1.08 | 0.00 |
| ENSG00000132746 | 86.65 | 3.59 | 0.00 | ENSG00000103449 | 91.57 | -1.08 | 0.00 |
| ENSG00000206075 | 2456.81 | 3.59 | 0.00 | ENSG00000130529 | 4592.32 | -1.08 | 0.00 |
| ENSG00000100344 | 51.12 | 3.59 | 0.00 | ENSG00000114541 | 588.65 | -1.08 | 0.00 |
| ENSG00000173391 | 186.15 | 3.58 | 0.00 | ENSG00000253570 | 57.73 | -1.08 | 0.00 |
| ENSG00000131188 | 391.93 | 3.58 | 0.00 | ENSG00000162616 | 359.42 | -1.08 | 0.00 |
| ENSG00000202198 | 564.09 | 3.57 | 0.00 | ENSG00000153563 | 288.28 | -1.08 | 0.00 |
| ENSG00000100593 | 87.03 | 3.55 | 0.00 | ENSG00000178078 | 3637.49 | -1.08 | 0.00 |
| ENSG00000169429 | 3886.67 | 3.55 | 0.00 | ENSG00000075568 | 2868.41 | -1.08 | 0.00 |
| ENSG00000171004 | 561.09 | 3.54 | 0.00 | ENSG00000152952 | 2577.69 | -1.08 | 0.00 |
| ENSG00000180537 | 53.22 | 3.54 | 0.00 | ENSG00000132854 | 52.10 | -1.08 | 0.00 |
| ENSG00000104327 | 125.19 | 3.54 | 0.00 | ENSG00000258311 | 56.62 | -1.08 | 0.00 |
| ENSG00000120875 | 1847.83 | 3.53 | 0.00 | ENSG00000174348 | 663.83 | -1.08 | 0.00 |
| ENSG00000253414 | 106.06 | 3.52 | 0.00 | ENSG00000018408 | 997.12 | -1.09 | 0.00 |
| ENSG00000138028 | 937.88 | 3.51 | 0.00 | ENSG00000160654 | 109.30 | -1.09 | 0.00 |
| ENSG00000151650 | 146.08 | 3.50 | 0.00 | ENSG00000115993 | 1725.16 | -1.09 | 0.00 |
| ENSG00000075461 | 154.22 | 3.50 | 0.00 | ENSG00000257151 | 50.62 | -1.09 | 0.00 |
| ENSG00000114656 | 160.32 | 3.50 | 0.00 | ENSG00000261534 | 78.29 | -1.09 | 0.00 |
| ENSG00000181652 | 199.41 | 3.49 | 0.00 | ENSG00000151240 | 611.82 | -1.09 | 0.00 |
| ENSG00000101187 | 2618.08 | 3.49 | 0.00 | ENSG00000198879 | 169.96 | -1.09 | 0.00 |
| ENSG00000137203 | 247.36 | 3.49 | 0.00 | ENSG00000129538 | 4483.21 | -1.09 | 0.00 |
| ENSG00000173557 | 183.42 | 3.46 | 0.00 | ENSG00000107537 | 1464.54 | -1.09 | 0.00 |
| ENSG00000210140 | 361.68 | 3.46 | 0.00 | ENSG00000240583 | 2240.10 | -1.09 | 0.00 |
| ENSG00000023839 | 245.90 | 3.45 | 0.00 | ENSG00000198478 | 4341.47 | -1.09 | 0.00 |
| ENSG00000166670 | 135.11 | 3.45 | 0.00 | ENSG00000204128 | 994.54 | -1.09 | 0.00 |
| ENSG00000187583 | 152.47 | 3.44 | 0.00 | ENSG00000144824 | 426.49 | -1.09 | 0.00 |
| ENSG00000131746 | 4734.17 | 3.44 | 0.00 | ENSG00000122420 | 62.81 | -1.09 | 0.00 |
| ENSG00000198759 | 120.73 | 3.43 | 0.00 | ENSG00000101331 | 54.68 | -1.09 | 0.00 |
| ENSG00000225972 | 309.30 | 3.42 | 0.00 | ENSG00000182195 | 114.09 | -1.09 | 0.00 |
| ENSG00000106038 | 235.61 | 3.42 | 0.00 | ENSG00000117791 | 423.52 | -1.09 | 0.00 |
| ENSG00000258227 | 103.40 | 3.42 | 0.00 | ENSG00000106789 | 2469.41 | -1.09 | 0.00 |
| ENSG00000131730 | 547.80 | 3.42 | 0.00 | ENSG00000099338 | 50.68 | -1.09 | 0.00 |
| ENSG00000163132 | 246.16 | 3.40 | 0.00 | ENSG00000112773 | 1077.04 | -1.09 | 0.00 |
| ENSG00000148734 | 129.25 | 3.40 | 0.00 | ENSG00000167601 | 1041.45 | -1.09 | 0.00 |
| ENSG00000151388 | 734.85 | 3.40 | 0.00 | ENSG00000204186 | 95.77 | -1.09 | 0.00 |
| ENSG00000213315 | 165.72 | 3.39 | 0.00 | ENSG00000100647 | 2525.76 | -1.09 | 0.00 |
| ENSG00000233695 | 531.91 | 3.38 | 0.00 | ENSG00000008382 | 1000.85 | -1.10 | 0.00 |
| ENSG00000169247 | 378.40 | 3.38 | 0.00 | ENSG00000118094 | 69.80 | -1.10 | 0.00 |
| ENSG00000057593 | 67.73 | 3.38 | 0.00 | ENSG00000204370 | 2815.86 | -1.10 | 0.00 |
| ENSG00000178752 | 92.28 | 3.35 | 0.00 | ENSG00000214189 | 56.26 | -1.10 | 0.00 |
| ENSG00000135480 | 366.54 | 3.35 | 0.00 | ENSG00000117528 | 2555.88 | -1.10 | 0.00 |
| ENSG00000167757 | 426.65 | 3.35 | 0.00 | ENSG00000151715 | 5753.67 | -1.10 | 0.00 |
| ENSG00000158106 | 999.44 | 3.34 | 0.00 | ENSG00000122121 | 585.76 | -1.10 | 0.00 |
| ENSG00000279806 | 244.86 | 3.30 | 0.00 | ENSG00000117643 | 264.14 | -1.10 | 0.00 |
| ENSG00000124882 | 1872.95 | 3.30 | 0.00 | ENSG00000095539 | 2267.46 | -1.10 | 0.00 |
| ENSG00000145244 | 79.82 | 3.29 | 0.00 | ENSG00000121406 | 98.58 | -1.10 | 0.00 |
| ENSG00000137747 | 368.32 | 3.29 | 0.00 | ENSG00000143515 | 546.99 | -1.10 | 0.00 |
| ENSG00000088002 | 900.84 | 3.28 | 0.00 | ENSG00000187017 | 1314.21 | -1.10 | 0.00 |
| ENSG00000165188 | 185.36 | 3.28 | 0.00 | ENSG00000182534 | 1519.89 | -1.10 | 0.00 |
| ENSG00000273079 | 232.91 | 3.27 | 0.00 | ENSG00000136068 | 16811.44 | -1.10 | 0.00 |
| ENSG00000159708 | 121.91 | 3.27 | 0.00 | ENSG00000151914 | 3838.63 | -1.10 | 0.00 |
| ENSG00000186474 | 219.42 | 3.27 | 0.00 | ENSG00000173376 | 206.98 | -1.10 | 0.00 |
| ENSG00000231369 | 54.12 | 3.27 | 0.00 | ENSG00000163884 | 60.19 | -1.10 | 0.00 |
| ENSG00000101144 | 1378.24 | 3.27 | 0.00 | ENSG00000167323 | 1971.62 | -1.10 | 0.00 |
| ENSG00000120708 | 32612.74 | 3.25 | 0.00 | ENSG00000065618 | 5499.33 | -1.10 | 0.00 |
| ENSG00000166682 | 67.91 | 3.25 | 0.00 | ENSG00000130449 | 660.28 | -1.10 | 0.00 |
| ENSG00000163817 | 1024.59 | 3.23 | 0.00 | ENSG00000120063 | 2459.59 | -1.10 | 0.00 |
| ENSG00000130513 | 6885.37 | 3.21 | 0.00 | ENSG00000185133 | 622.36 | -1.10 | 0.00 |
| ENSG00000078098 | 433.10 | 3.20 | 0.00 | ENSG00000168421 | 165.49 | -1.10 | 0.00 |
| ENSG00000240652 | 82.29 | 3.20 | 0.00 | ENSG00000127954 | 194.58 | -1.10 | 0.00 |
| ENSG00000230061 | 153.66 | 3.20 | 0.00 | ENSG00000020577 | 518.32 | -1.10 | 0.00 |
| ENSG00000106536 | 67.61 | 3.19 | 0.00 | ENSG00000116774 | 757.79 | -1.10 | 0.00 |
| ENSG00000227619 | 93.62 | 3.19 | 0.00 | ENSG00000161405 | 367.08 | -1.10 | 0.00 |
| ENSG00000162849 | 487.67 | 3.19 | 0.00 | ENSG00000106299 | 3163.97 | -1.10 | 0.00 |
| ENSG00000009950 | 1477.25 | 3.18 | 0.00 | ENSG00000007264 | 98.92 | -1.10 | 0.00 |
| ENSG00000149295 | 147.84 | 3.18 | 0.00 | ENSG00000100412 | 4592.83 | -1.11 | 0.00 |
| ENSG00000253161 | 85.05 | 3.18 | 0.00 | ENSG00000173599 | 708.85 | -1.11 | 0.00 |
| ENSG00000250829 | 77.00 | 3.17 | 0.00 | ENSG00000169435 | 897.42 | -1.11 | 0.00 |
| ENSG00000175894 | 60.47 | 3.17 | 0.00 | ENSG00000196139 | 2521.25 | -1.11 | 0.00 |
| ENSG00000156076 | 127.15 | 3.16 | 0.00 | ENSG00000148175 | 2867.54 | -1.11 | 0.00 |
| ENSG00000171388 | 326.98 | 3.16 | 0.00 | ENSG00000213977 | 1063.75 | -1.11 | 0.00 |
| ENSG00000159261 | 52.64 | 3.15 | 0.00 | ENSG00000088543 | 104.42 | -1.11 | 0.00 |
| ENSG00000114270 | 1268.35 | 3.14 | 0.00 | ENSG00000104043 | 83.25 | -1.11 | 0.00 |
| ENSG00000106483 | 1404.41 | 3.13 | 0.00 | ENSG00000260244 | 229.48 | -1.11 | 0.00 |
| ENSG00000173898 | 1390.34 | 3.13 | 0.00 | ENSG00000091157 | 469.25 | -1.11 | 0.00 |
| ENSG00000180730 | 135.86 | 3.13 | 0.00 | ENSG00000204472 | 399.58 | -1.11 | 0.00 |
| ENSG00000142102 | 2882.79 | 3.13 | 0.00 | ENSG00000196507 | 225.19 | -1.11 | 0.00 |
| ENSG00000160862 | 2583.89 | 3.12 | 0.00 | ENSG00000128266 | 118.61 | -1.11 | 0.00 |
| ENSG00000103257 | 6901.70 | 3.11 | 0.00 | ENSG00000170745 | 330.10 | -1.11 | 0.00 |
| ENSG00000151224 | 136.20 | 3.10 | 0.00 | ENSG00000099330 | 516.71 | -1.11 | 0.00 |
| ENSG00000137877 | 262.00 | 3.09 | 0.00 | ENSG00000131831 | 270.22 | -1.11 | 0.00 |
| ENSG00000198807 | 84.08 | 3.09 | 0.00 | ENSG00000131849 | 62.84 | -1.11 | 0.00 |
| ENSG00000166840 | 80.65 | 3.09 | 0.00 | ENSG00000081237 | 1090.50 | -1.11 | 0.00 |
| ENSG00000086991 | 83.98 | 3.09 | 0.00 | ENSG00000243646 | 1831.57 | -1.11 | 0.00 |
| ENSG00000200087 | 53.32 | 3.09 | 0.00 | ENSG00000050555 | 141.61 | -1.11 | 0.00 |
| ENSG00000186081 | 61.13 | 3.09 | 0.00 | ENSG00000272221 | 57.15 | -1.11 | 0.00 |
| ENSG00000094755 | 258.79 | 3.08 | 0.00 | ENSG00000133731 | 1020.67 | -1.11 | 0.00 |
| ENSG00000107984 | 58.65 | 3.08 | 0.00 | ENSG00000187098 | 234.43 | -1.11 | 0.00 |
| ENSG00000111432 | 247.41 | 3.08 | 0.00 | ENSG00000196159 | 365.53 | -1.12 | 0.00 |
| ENSG00000170577 | 56.64 | 3.07 | 0.00 | ENSG00000100014 | 960.44 | -1.12 | 0.00 |
| ENSG00000064195 | 65.64 | 3.07 | 0.00 | ENSG00000211950 | 341.21 | -1.12 | 0.00 |
| ENSG00000157766 | 324.21 | 3.06 | 0.00 | ENSG00000099812 | 14224.29 | -1.12 | 0.00 |
| ENSG00000265688 | 198.52 | 3.05 | 0.00 | ENSG00000153094 | 893.06 | -1.12 | 0.00 |
| ENSG00000187642 | 53.69 | 3.05 | 0.00 | ENSG00000137502 | 440.53 | -1.12 | 0.00 |
| ENSG00000262585 | 71.42 | 3.04 | 0.00 | ENSG00000004468 | 197.47 | -1.12 | 0.00 |
| ENSG00000092758 | 925.72 | 3.04 | 0.00 | ENSG00000151067 | 602.84 | -1.12 | 0.00 |
| ENSG00000241186 | 942.04 | 3.03 | 0.00 | ENSG00000164938 | 834.81 | -1.12 | 0.00 |
| ENSG00000177984 | 919.42 | 3.03 | 0.00 | ENSG00000180787 | 270.23 | -1.12 | 0.00 |
| ENSG00000164822 | 670.06 | 3.02 | 0.00 | ENSG00000067141 | 4398.70 | -1.12 | 0.00 |
| ENSG00000152669 | 368.79 | 3.02 | 0.00 | ENSG00000170989 | 415.24 | -1.12 | 0.00 |
| ENSG00000175592 | 595.91 | 3.02 | 0.00 | ENSG00000101596 | 1547.94 | -1.12 | 0.00 |
| ENSG00000011201 | 293.56 | 3.01 | 0.00 | ENSG00000117298 | 5550.48 | -1.12 | 0.00 |
| ENSG00000102359 | 1062.80 | 3.00 | 0.00 | ENSG00000100485 | 1251.57 | -1.13 | 0.00 |
| ENSG00000101104 | 941.80 | 3.00 | 0.00 | ENSG00000110848 | 138.21 | -1.13 | 0.00 |
| ENSG00000166415 | 584.92 | 3.00 | 0.00 | ENSG00000188153 | 163.14 | -1.13 | 0.00 |
| ENSG00000115008 | 93.45 | 2.99 | 0.00 | ENSG00000005108 | 69.86 | -1.13 | 0.00 |
| ENSG00000129474 | 829.12 | 2.99 | 0.00 | ENSG00000116171 | 4608.91 | -1.13 | 0.00 |
| ENSG00000172927 | 1108.35 | 2.98 | 0.00 | ENSG00000164736 | 63.90 | -1.13 | 0.00 |
| ENSG00000163734 | 1643.24 | 2.98 | 0.00 | ENSG00000163297 | 2555.37 | -1.13 | 0.00 |
| ENSG00000157193 | 788.73 | 2.98 | 0.00 | ENSG00000091656 | 88.87 | -1.13 | 0.00 |
| ENSG00000185105 | 85.29 | 2.97 | 0.00 | ENSG00000203943 | 275.31 | -1.13 | 0.00 |
| ENSG00000102854 | 2863.45 | 2.97 | 0.00 | ENSG00000165424 | 874.45 | -1.13 | 0.00 |
| ENSG00000186377 | 381.63 | 2.96 | 0.00 | ENSG00000107242 | 1791.01 | -1.13 | 0.00 |
| ENSG00000183914 | 199.06 | 2.96 | 0.00 | ENSG00000120833 | 383.14 | -1.13 | 0.00 |
| ENSG00000147889 | 334.32 | 2.96 | 0.00 | ENSG00000181885 | 12639.12 | -1.13 | 0.00 |
| ENSG00000224114 | 175.82 | 2.95 | 0.00 | ENSG00000124762 | 6307.54 | -1.13 | 0.00 |
| ENSG00000160183 | 515.92 | 2.95 | 0.00 | ENSG00000156381 | 1674.43 | -1.13 | 0.00 |
| ENSG00000279184 | 66.90 | 2.94 | 0.00 | ENSG00000163219 | 248.15 | -1.13 | 0.00 |
| ENSG00000204740 | 106.35 | 2.93 | 0.00 | ENSG00000137486 | 1341.11 | -1.13 | 0.00 |
| ENSG00000179431 | 174.26 | 2.92 | 0.00 | ENSG00000173467 | 1509.54 | -1.13 | 0.00 |
| ENSG00000109255 | 183.64 | 2.92 | 0.00 | ENSG00000175482 | 963.19 | -1.13 | 0.00 |
| ENSG00000163739 | 2831.55 | 2.91 | 0.00 | ENSG00000112139 | 111.42 | -1.14 | 0.00 |
| ENSG00000240616 | 79.46 | 2.91 | 0.00 | ENSG00000145423 | 2403.36 | -1.14 | 0.00 |
| ENSG00000280693 | 155.21 | 2.88 | 0.00 | ENSG00000169071 | 354.47 | -1.14 | 0.00 |
| ENSG00000187867 | 133.18 | 2.88 | 0.00 | ENSG00000157833 | 156.63 | -1.14 | 0.00 |
| ENSG00000125895 | 250.20 | 2.88 | 0.00 | ENSG00000135540 | 2003.28 | -1.14 | 0.00 |
| ENSG00000203805 | 89.06 | 2.87 | 0.00 | ENSG00000198643 | 8609.61 | -1.14 | 0.00 |
| ENSG00000232445 | 179.34 | 2.87 | 0.00 | ENSG00000096060 | 1841.90 | -1.14 | 0.00 |
| ENSG00000203499 | 1239.68 | 2.87 | 0.00 | ENSG00000172661 | 891.35 | -1.14 | 0.00 |
| ENSG00000183019 | 50.82 | 2.87 | 0.00 | ENSG00000109062 | 4538.42 | -1.14 | 0.00 |
| ENSG00000174951 | 314.73 | 2.86 | 0.00 | ENSG00000205744 | 1048.52 | -1.14 | 0.00 |
| ENSG00000187730 | 51.23 | 2.86 | 0.00 | ENSG00000163406 | 102.42 | -1.14 | 0.00 |
| ENSG00000233922 | 51.96 | 2.86 | 0.00 | ENSG00000071205 | 311.31 | -1.14 | 0.00 |
| ENSG00000244300 | 139.89 | 2.86 | 0.00 | ENSG00000132718 | 398.39 | -1.14 | 0.00 |
| ENSG00000254726 | 545.24 | 2.86 | 0.00 | ENSG00000214900 | 86.73 | -1.14 | 0.00 |
| ENSG00000135960 | 604.13 | 2.84 | 0.00 | ENSG00000185156 | 122.65 | -1.14 | 0.00 |
| ENSG00000139292 | 2666.93 | 2.84 | 0.00 | ENSG00000186564 | 518.13 | -1.15 | 0.00 |
| ENSG00000147206 | 90.64 | 2.84 | 0.00 | ENSG00000169026 | 134.89 | -1.15 | 0.00 |
| ENSG00000211676 | 78.20 | 2.83 | 0.00 | ENSG00000115129 | 1203.49 | -1.15 | 0.00 |
| ENSG00000106327 | 176.84 | 2.82 | 0.00 | ENSG00000100266 | 3583.46 | -1.15 | 0.00 |
| ENSG00000186493 | 55.12 | 2.81 | 0.00 | ENSG00000155066 | 1250.04 | -1.15 | 0.00 |
| ENSG00000133048 | 1037.63 | 2.80 | 0.00 | ENSG00000125354 | 963.35 | -1.15 | 0.00 |
| ENSG00000088340 | 650.06 | 2.80 | 0.00 | ENSG00000128815 | 263.77 | -1.15 | 0.00 |
| ENSG00000180914 | 78.25 | 2.80 | 0.00 | ENSG00000111424 | 3813.32 | -1.15 | 0.00 |
| ENSG00000220583 | 69.58 | 2.80 | 0.00 | ENSG00000182107 | 2379.19 | -1.15 | 0.00 |
| ENSG00000125931 | 57.37 | 2.80 | 0.00 | ENSG00000269113 | 61.63 | -1.15 | 0.00 |
| ENSG00000198203 | 439.42 | 2.80 | 0.00 | ENSG00000105227 | 108.82 | -1.15 | 0.00 |
| ENSG00000110944 | 99.77 | 2.79 | 0.00 | ENSG00000221890 | 217.56 | -1.16 | 0.00 |
| ENSG00000272502 | 154.97 | 2.78 | 0.00 | ENSG00000211970 | 174.45 | -1.16 | 0.00 |
| ENSG00000120068 | 1264.41 | 2.78 | 0.00 | ENSG00000111077 | 1244.03 | -1.16 | 0.00 |
| ENSG00000105707 | 145.53 | 2.76 | 0.00 | ENSG00000049130 | 1008.92 | -1.16 | 0.00 |
| ENSG00000249859 | 486.82 | 2.76 | 0.00 | ENSG00000111727 | 292.27 | -1.16 | 0.00 |
| ENSG00000160161 | 64.54 | 2.76 | 0.00 | ENSG00000272106 | 129.83 | -1.16 | 0.00 |
| ENSG00000138100 | 114.42 | 2.76 | 0.00 | ENSG00000052795 | 1442.96 | -1.16 | 0.00 |
| ENSG00000131389 | 4037.82 | 2.75 | 0.00 | ENSG00000131981 | 30530.98 | -1.16 | 0.00 |
| ENSG00000169174 | 2434.55 | 2.73 | 0.00 | ENSG00000180353 | 819.32 | -1.16 | 0.00 |
| ENSG00000183971 | 58.78 | 2.73 | 0.00 | ENSG00000139194 | 103.20 | -1.16 | 0.00 |
| ENSG00000135069 | 2111.62 | 2.72 | 0.00 | ENSG00000272462 | 86.35 | -1.16 | 0.00 |
| ENSG00000235297 | 94.77 | 2.72 | 0.00 | ENSG00000237289 | 648.01 | -1.16 | 0.00 |
| ENSG00000185332 | 69.42 | 2.72 | 0.00 | ENSG00000160746 | 1625.01 | -1.16 | 0.00 |
| ENSG00000174672 | 217.60 | 2.72 | 0.00 | ENSG00000267194 | 364.69 | -1.16 | 0.00 |
| ENSG00000151012 | 857.59 | 2.72 | 0.00 | ENSG00000108797 | 318.94 | -1.16 | 0.00 |
| ENSG00000196549 | 709.74 | 2.72 | 0.00 | ENSG00000147852 | 280.10 | -1.16 | 0.00 |
| ENSG00000158555 | 1271.66 | 2.72 | 0.00 | ENSG00000156030 | 1448.10 | -1.17 | 0.00 |
| ENSG00000163032 | 1017.56 | 2.71 | 0.00 | ENSG00000128872 | 310.85 | -1.17 | 0.00 |
| ENSG00000269242 | 118.71 | 2.69 | 0.00 | ENSG00000001460 | 369.87 | -1.17 | 0.00 |
| ENSG00000220472 | 94.13 | 2.68 | 0.00 | ENSG00000187534 | 82.27 | -1.17 | 0.00 |
| ENSG00000008300 | 795.74 | 2.67 | 0.00 | ENSG00000160613 | 1933.58 | -1.17 | 0.00 |
| ENSG00000170561 | 200.79 | 2.66 | 0.00 | ENSG00000120913 | 604.49 | -1.17 | 0.00 |
| ENSG00000241547 | 82.52 | 2.66 | 0.00 | ENSG00000171385 | 204.02 | -1.17 | 0.00 |
| ENSG00000124731 | 149.76 | 2.65 | 0.00 | ENSG00000137285 | 109.39 | -1.17 | 0.00 |
| ENSG00000106236 | 885.64 | 2.64 | 0.00 | ENSG00000162437 | 1585.46 | -1.17 | 0.00 |
| ENSG00000248626 | 50.35 | 2.64 | 0.00 | ENSG00000110076 | 195.74 | -1.17 | 0.00 |
| ENSG00000132031 | 98.45 | 2.64 | 0.00 | ENSG00000100842 | 120.47 | -1.17 | 0.00 |
| ENSG00000183091 | 121.87 | 2.64 | 0.00 | ENSG00000168490 | 53.03 | -1.17 | 0.00 |
| ENSG00000085741 | 825.10 | 2.63 | 0.00 | ENSG00000060762 | 1331.65 | -1.17 | 0.00 |
| ENSG00000108821 | 88628.96 | 2.63 | 0.00 | ENSG00000100784 | 452.84 | -1.17 | 0.00 |
| ENSG00000139289 | 5389.62 | 2.62 | 0.00 | ENSG00000106351 | 1589.80 | -1.18 | 0.00 |
| ENSG00000131910 | 143.89 | 2.62 | 0.00 | ENSG00000081320 | 1439.11 | -1.18 | 0.00 |
| ENSG00000197046 | 80.69 | 2.62 | 0.00 | ENSG00000115896 | 88.07 | -1.18 | 0.00 |
| ENSG00000158023 | 98.69 | 2.62 | 0.00 | ENSG00000163131 | 9050.00 | -1.18 | 0.00 |
| ENSG00000104415 | 270.37 | 2.61 | 0.00 | ENSG00000077782 | 1158.49 | -1.18 | 0.00 |
| ENSG00000050344 | 4465.28 | 2.61 | 0.00 | ENSG00000005844 | 481.58 | -1.18 | 0.00 |
| ENSG00000119547 | 189.93 | 2.60 | 0.00 | ENSG00000167552 | 3009.83 | -1.18 | 0.00 |
| ENSG00000182272 | 367.71 | 2.60 | 0.00 | ENSG00000156299 | 154.02 | -1.18 | 0.00 |
| ENSG00000104313 | 57.61 | 2.59 | 0.00 | ENSG00000176014 | 1133.70 | -1.18 | 0.00 |
| ENSG00000226887 | 114.04 | 2.59 | 0.00 | ENSG00000089486 | 530.10 | -1.18 | 0.00 |
| ENSG00000168243 | 1412.94 | 2.59 | 0.00 | ENSG00000185303 | 68.92 | -1.19 | 0.00 |
| ENSG00000185686 | 110.01 | 2.59 | 0.00 | ENSG00000176148 | 258.21 | -1.19 | 0.00 |
| ENSG00000215417 | 110.17 | 2.59 | 0.00 | ENSG00000172380 | 4998.27 | -1.19 | 0.00 |
| ENSG00000006118 | 1441.52 | 2.59 | 0.00 | ENSG00000165895 | 376.86 | -1.19 | 0.00 |
| ENSG00000140519 | 80.26 | 2.58 | 0.00 | ENSG00000134853 | 1126.16 | -1.19 | 0.00 |
| ENSG00000100625 | 53.44 | 2.58 | 0.00 | ENSG00000170296 | 1145.45 | -1.19 | 0.00 |
| ENSG00000140093 | 76.25 | 2.57 | 0.00 | ENSG00000158006 | 1143.04 | -1.19 | 0.00 |
| ENSG00000181773 | 55.90 | 2.57 | 0.00 | ENSG00000280798 | 307.19 | -1.19 | 0.00 |
| ENSG00000141505 | 160.94 | 2.56 | 0.00 | ENSG00000109743 | 177.55 | -1.19 | 0.00 |
| ENSG00000205502 | 300.56 | 2.56 | 0.00 | ENSG00000164116 | 481.11 | -1.19 | 0.00 |
| ENSG00000232888 | 228.34 | 2.55 | 0.00 | ENSG00000081026 | 893.38 | -1.19 | 0.00 |
| ENSG00000110427 | 161.27 | 2.54 | 0.00 | ENSG00000259291 | 191.38 | -1.19 | 0.00 |
| ENSG00000268104 | 590.63 | 2.54 | 0.00 | ENSG00000173212 | 67.80 | -1.19 | 0.00 |
| ENSG00000099625 | 52.26 | 2.54 | 0.00 | ENSG00000166428 | 62.57 | -1.19 | 0.00 |
| ENSG00000261373 | 436.44 | 2.54 | 0.00 | ENSG00000116473 | 2161.81 | -1.20 | 0.00 |
| ENSG00000222033 | 105.93 | 2.53 | 0.00 | ENSG00000162779 | 105.43 | -1.20 | 0.00 |
| ENSG00000139055 | 228.30 | 2.53 | 0.00 | ENSG00000110934 | 261.68 | -1.20 | 0.00 |
| ENSG00000134193 | 22711.88 | 2.53 | 0.00 | ENSG00000162909 | 8975.65 | -1.20 | 0.00 |
| ENSG00000197506 | 294.02 | 2.52 | 0.00 | ENSG00000185070 | 188.27 | -1.20 | 0.00 |
| ENSG00000205702 | 55.29 | 2.52 | 0.00 | ENSG00000274993 | 378.09 | -1.20 | 0.00 |
| ENSG00000251562 | 13527.85 | 2.52 | 0.00 | ENSG00000225953 | 460.20 | -1.20 | 0.00 |
| ENSG00000182492 | 8810.55 | 2.51 | 0.00 | ENSG00000251039 | 53.86 | -1.20 | 0.01 |
| ENSG00000213763 | 134.47 | 2.51 | 0.00 | ENSG00000026103 | 622.31 | -1.20 | 0.00 |
| ENSG00000246228 | 89.16 | 2.50 | 0.00 | ENSG00000126353 | 113.68 | -1.20 | 0.00 |
| ENSG00000115386 | 13064.46 | 2.50 | 0.00 | ENSG00000163681 | 2303.33 | -1.20 | 0.00 |
| ENSG00000154856 | 3365.73 | 2.50 | 0.00 | ENSG00000176595 | 1209.09 | -1.20 | 0.00 |
| ENSG00000021826 | 281.44 | 2.50 | 0.00 | ENSG00000175662 | 1819.62 | -1.20 | 0.00 |
| ENSG00000198542 | 168.38 | 2.49 | 0.00 | ENSG00000198695 | 24513.75 | -1.20 | 0.00 |
| ENSG00000163993 | 8745.94 | 2.49 | 0.00 | ENSG00000069702 | 745.47 | -1.20 | 0.00 |
| ENSG00000226380 | 114.34 | 2.49 | 0.00 | ENSG00000183741 | 1167.89 | -1.20 | 0.00 |
| ENSG00000183960 | 164.51 | 2.47 | 0.00 | ENSG00000111962 | 123.93 | -1.20 | 0.00 |
| ENSG00000003249 | 847.01 | 2.47 | 0.00 | ENSG00000107130 | 1215.51 | -1.20 | 0.00 |
| ENSG00000179698 | 76.12 | 2.47 | 0.00 | ENSG00000082196 | 170.18 | -1.20 | 0.00 |
| ENSG00000155265 | 316.75 | 2.46 | 0.00 | ENSG00000173369 | 3478.07 | -1.20 | 0.00 |
| ENSG00000213937 | 69.85 | 2.46 | 0.00 | ENSG00000112799 | 88.80 | -1.20 | 0.00 |
| ENSG00000169248 | 581.38 | 2.46 | 0.00 | ENSG00000069431 | 134.88 | -1.20 | 0.00 |
| ENSG00000174939 | 327.91 | 2.45 | 0.00 | ENSG00000120279 | 123.80 | -1.20 | 0.00 |
| ENSG00000167914 | 133.98 | 2.45 | 0.00 | ENSG00000162545 | 4885.57 | -1.20 | 0.00 |
| ENSG00000128578 | 258.70 | 2.44 | 0.00 | ENSG00000101188 | 153.48 | -1.21 | 0.00 |
| ENSG00000006327 | 1992.97 | 2.44 | 0.00 | ENSG00000176658 | 14886.12 | -1.21 | 0.00 |
| ENSG00000243587 | 90.74 | 2.43 | 0.00 | ENSG00000068615 | 309.07 | -1.21 | 0.00 |
| ENSG00000198723 | 144.57 | 2.43 | 0.00 | ENSG00000106853 | 1169.23 | -1.21 | 0.00 |
| ENSG00000101194 | 1865.00 | 2.43 | 0.00 | ENSG00000170456 | 366.17 | -1.21 | 0.00 |
| ENSG00000168646 | 5119.63 | 2.42 | 0.00 | ENSG00000102349 | 75.10 | -1.21 | 0.00 |
| ENSG00000134339 | 115.39 | 2.42 | 0.00 | ENSG00000135362 | 1754.69 | -1.21 | 0.00 |
| ENSG00000182489 | 224.96 | 2.42 | 0.00 | ENSG00000076356 | 2214.26 | -1.21 | 0.00 |
| ENSG00000238279 | 88.71 | 2.41 | 0.00 | ENSG00000105289 | 3889.46 | -1.21 | 0.00 |
| ENSG00000175426 | 838.70 | 2.41 | 0.00 | ENSG00000104324 | 674.72 | -1.21 | 0.00 |
| ENSG00000075275 | 1450.42 | 2.41 | 0.00 | ENSG00000088756 | 66.17 | -1.21 | 0.00 |
| ENSG00000117122 | 667.18 | 2.41 | 0.00 | ENSG00000101265 | 502.00 | -1.21 | 0.00 |
| ENSG00000185633 | 771.30 | 2.41 | 0.00 | ENSG00000145088 | 102.99 | -1.21 | 0.00 |
| ENSG00000189431 | 253.97 | 2.41 | 0.00 | ENSG00000168824 | 132.70 | -1.21 | 0.00 |
| ENSG00000198720 | 619.51 | 2.40 | 0.00 | ENSG00000165633 | 311.49 | -1.21 | 0.00 |
| ENSG00000120254 | 1717.92 | 2.40 | 0.00 | ENSG00000146555 | 227.64 | -1.22 | 0.00 |
| ENSG00000172023 | 1511.45 | 2.40 | 0.00 | ENSG00000134352 | 2296.88 | -1.22 | 0.00 |
| ENSG00000234753 | 82.06 | 2.39 | 0.00 | ENSG00000121900 | 8102.70 | -1.22 | 0.00 |
| ENSG00000231503 | 118.91 | 2.39 | 0.00 | ENSG00000103034 | 158.21 | -1.22 | 0.00 |
| ENSG00000135245 | 1129.76 | 2.38 | 0.00 | ENSG00000240184 | 309.92 | -1.22 | 0.00 |
| ENSG00000039139 | 57.32 | 2.38 | 0.00 | ENSG00000122122 | 438.63 | -1.22 | 0.00 |
| ENSG00000112299 | 447.23 | 2.37 | 0.00 | ENSG00000105711 | 100.03 | -1.22 | 0.00 |
| ENSG00000140274 | 1272.51 | 2.37 | 0.00 | ENSG00000132640 | 1456.17 | -1.22 | 0.00 |
| ENSG00000262370 | 93.26 | 2.37 | 0.00 | ENSG00000104723 | 248.84 | -1.22 | 0.00 |
| ENSG00000127418 | 3210.67 | 2.37 | 0.00 | ENSG00000235501 | 103.33 | -1.22 | 0.00 |
| ENSG00000087116 | 1633.97 | 2.36 | 0.00 | ENSG00000130766 | 911.49 | -1.22 | 0.00 |
| ENSG00000088826 | 874.32 | 2.36 | 0.00 | ENSG00000115468 | 91.14 | -1.23 | 0.00 |
| ENSG00000204876 | 683.42 | 2.36 | 0.00 | ENSG00000170390 | 114.22 | -1.23 | 0.00 |
| ENSG00000108379 | 119.64 | 2.36 | 0.00 | ENSG00000063438 | 69.83 | -1.23 | 0.00 |
| ENSG00000149599 | 99.10 | 2.36 | 0.00 | ENSG00000128487 | 1068.43 | -1.23 | 0.00 |
| ENSG00000029153 | 923.02 | 2.36 | 0.00 | ENSG00000159189 | 3396.07 | -1.23 | 0.00 |
| ENSG00000143882 | 302.83 | 2.36 | 0.00 | ENSG00000143248 | 2477.51 | -1.23 | 0.00 |
| ENSG00000018280 | 327.92 | 2.35 | 0.00 | ENSG00000234456 | 164.71 | -1.23 | 0.00 |
| ENSG00000078114 | 3184.17 | 2.35 | 0.00 | ENSG00000117682 | 1415.14 | -1.23 | 0.00 |
| ENSG00000225093 | 117.67 | 2.34 | 0.00 | ENSG00000123360 | 73.38 | -1.23 | 0.00 |
| ENSG00000184949 | 77.89 | 2.34 | 0.00 | ENSG00000137672 | 55.02 | -1.23 | 0.00 |
| ENSG00000081041 | 884.98 | 2.34 | 0.00 | ENSG00000159200 | 1402.84 | -1.23 | 0.00 |
| ENSG00000138823 | 187.12 | 2.34 | 0.00 | ENSG00000250510 | 55.87 | -1.24 | 0.00 |
| ENSG00000071539 | 952.99 | 2.32 | 0.00 | ENSG00000277443 | 6353.45 | -1.24 | 0.00 |
| ENSG00000116661 | 326.76 | 2.31 | 0.00 | ENSG00000109458 | 904.62 | -1.24 | 0.00 |
| ENSG00000124875 | 99.10 | 2.31 | 0.00 | ENSG00000047346 | 596.61 | -1.24 | 0.00 |
| ENSG00000099194 | 17572.43 | 2.30 | 0.00 | ENSG00000024422 | 1881.06 | -1.24 | 0.00 |
| ENSG00000111110 | 1580.02 | 2.30 | 0.00 | ENSG00000077420 | 221.24 | -1.24 | 0.00 |
| ENSG00000198734 | 280.73 | 2.30 | 0.00 | ENSG00000186212 | 494.35 | -1.24 | 0.00 |
| ENSG00000197905 | 1035.45 | 2.30 | 0.00 | ENSG00000166532 | 165.64 | -1.24 | 0.00 |
| ENSG00000134317 | 210.38 | 2.30 | 0.00 | ENSG00000137269 | 1522.55 | -1.24 | 0.00 |
| ENSG00000186340 | 4699.41 | 2.30 | 0.00 | ENSG00000184613 | 118.78 | -1.24 | 0.00 |
| ENSG00000078900 | 179.12 | 2.29 | 0.00 | ENSG00000239697 | 419.38 | -1.25 | 0.00 |
| ENSG00000180211 | 75.67 | 2.29 | 0.00 | ENSG00000135363 | 211.20 | -1.25 | 0.00 |
| ENSG00000143839 | 80.85 | 2.29 | 0.00 | ENSG00000099958 | 643.97 | -1.25 | 0.00 |
| ENSG00000270885 | 105.62 | 2.28 | 0.00 | ENSG00000129675 | 376.79 | -1.25 | 0.00 |
| ENSG00000122778 | 934.80 | 2.28 | 0.00 | ENSG00000169442 | 412.26 | -1.25 | 0.00 |
| ENSG00000204839 | 1221.36 | 2.27 | 0.00 | ENSG00000000971 | 876.78 | -1.25 | 0.00 |
| ENSG00000148346 | 23865.69 | 2.27 | 0.00 | ENSG00000135052 | 10691.69 | -1.25 | 0.00 |
| ENSG00000199753 | 56.04 | 2.26 | 0.00 | ENSG00000204161 | 187.89 | -1.25 | 0.00 |
| ENSG00000261295 | 52.95 | 2.26 | 0.00 | ENSG00000133424 | 901.58 | -1.25 | 0.00 |
| ENSG00000175920 | 118.15 | 2.26 | 0.00 | ENSG00000165807 | 140.25 | -1.25 | 0.00 |
| ENSG00000108375 | 9365.12 | 2.25 | 0.00 | ENSG00000011198 | 484.06 | -1.25 | 0.00 |
| ENSG00000184489 | 2223.07 | 2.25 | 0.00 | ENSG00000197128 | 63.13 | -1.25 | 0.00 |
| ENSG00000172164 | 2122.25 | 2.25 | 0.00 | ENSG00000227766 | 344.75 | -1.25 | 0.00 |
| ENSG00000100003 | 273.39 | 2.25 | 0.00 | ENSG00000177575 | 986.30 | -1.25 | 0.00 |
| ENSG00000134668 | 95.82 | 2.24 | 0.00 | ENSG00000126860 | 153.46 | -1.26 | 0.00 |
| ENSG00000211896 | 48523.27 | 2.23 | 0.00 | ENSG00000113790 | 759.66 | -1.26 | 0.00 |
| ENSG00000160181 | 216.69 | 2.23 | 0.00 | ENSG00000160539 | 53.58 | -1.26 | 0.00 |
| ENSG00000262001 | 348.96 | 2.23 | 0.00 | ENSG00000167555 | 135.15 | -1.26 | 0.00 |
| ENSG00000102287 | 1125.36 | 2.22 | 0.00 | ENSG00000127914 | 2093.73 | -1.26 | 0.00 |
| ENSG00000160957 | 1591.98 | 2.22 | 0.00 | ENSG00000159433 | 242.14 | -1.26 | 0.00 |
| ENSG00000142623 | 83.95 | 2.22 | 0.00 | ENSG00000129682 | 99.64 | -1.26 | 0.00 |
| ENSG00000204889 | 81.84 | 2.21 | 0.00 | ENSG00000075651 | 1120.06 | -1.26 | 0.00 |
| ENSG00000215030 | 1772.55 | 2.21 | 0.00 | ENSG00000075884 | 134.22 | -1.26 | 0.00 |
| ENSG00000226958 | 83.18 | 2.21 | 0.00 | ENSG00000116991 | 1416.66 | -1.27 | 0.00 |
| ENSG00000143333 | 661.07 | 2.21 | 0.00 | ENSG00000181035 | 257.95 | -1.27 | 0.00 |
| ENSG00000130720 | 591.09 | 2.21 | 0.00 | ENSG00000228672 | 174.03 | -1.27 | 0.00 |
| ENSG00000235587 | 102.81 | 2.21 | 0.00 | ENSG00000171243 | 237.04 | -1.27 | 0.00 |
| ENSG00000088899 | 1395.87 | 2.20 | 0.00 | ENSG00000163898 | 2342.27 | -1.27 | 0.00 |
| ENSG00000163083 | 314.67 | 2.20 | 0.00 | ENSG00000188761 | 1639.34 | -1.27 | 0.00 |
| ENSG00000269821 | 314.24 | 2.19 | 0.00 | ENSG00000258837 | 144.73 | -1.27 | 0.00 |
| ENSG00000131650 | 84.64 | 2.19 | 0.00 | ENSG00000073417 | 1430.29 | -1.27 | 0.00 |
| ENSG00000127423 | 187.70 | 2.19 | 0.00 | ENSG00000198160 | 1600.23 | -1.27 | 0.00 |
| ENSG00000158014 | 127.96 | 2.19 | 0.00 | ENSG00000157823 | 905.56 | -1.27 | 0.00 |
| ENSG00000213058 | 577.63 | 2.19 | 0.00 | ENSG00000123338 | 661.14 | -1.27 | 0.00 |
| ENSG00000272899 | 158.18 | 2.19 | 0.00 | ENSG00000161929 | 103.49 | -1.27 | 0.00 |
| ENSG00000102048 | 294.62 | 2.18 | 0.00 | ENSG00000205213 | 2684.17 | -1.27 | 0.00 |
| ENSG00000179603 | 532.33 | 2.18 | 0.00 | ENSG00000266714 | 5576.50 | -1.28 | 0.00 |
| ENSG00000103569 | 191.36 | 2.17 | 0.00 | ENSG00000211898 | 109.58 | -1.28 | 0.00 |
| ENSG00000101224 | 5702.93 | 2.17 | 0.00 | ENSG00000154153 | 1137.86 | -1.28 | 0.00 |
| ENSG00000141527 | 161.41 | 2.17 | 0.00 | ENSG00000125740 | 1509.21 | -1.28 | 0.00 |
| ENSG00000163618 | 737.68 | 2.17 | 0.00 | ENSG00000178301 | 133.10 | -1.28 | 0.00 |
| ENSG00000164690 | 951.92 | 2.17 | 0.00 | ENSG00000172238 | 786.83 | -1.28 | 0.00 |
| ENSG00000006704 | 2498.36 | 2.17 | 0.00 | ENSG00000076351 | 231.94 | -1.28 | 0.00 |
| ENSG00000270933 | 76.88 | 2.16 | 0.00 | ENSG00000179981 | 1004.93 | -1.28 | 0.00 |
| ENSG00000183742 | 2277.72 | 2.16 | 0.00 | ENSG00000116016 | 5252.53 | -1.28 | 0.00 |
| ENSG00000278919 | 79.14 | 2.15 | 0.00 | ENSG00000164251 | 3574.23 | -1.28 | 0.00 |
| ENSG00000099985 | 213.17 | 2.15 | 0.00 | ENSG00000120899 | 2088.48 | -1.28 | 0.00 |
| ENSG00000184368 | 349.45 | 2.15 | 0.00 | ENSG00000220205 | 1243.10 | -1.28 | 0.00 |
| ENSG00000211897 | 5902.17 | 2.14 | 0.00 | ENSG00000227258 | 171.09 | -1.28 | 0.00 |
| ENSG00000186862 | 54.97 | 2.14 | 0.00 | ENSG00000134516 | 463.86 | -1.29 | 0.00 |
| ENSG00000184925 | 150.47 | 2.14 | 0.00 | ENSG00000184005 | 75.25 | -1.29 | 0.00 |
| ENSG00000149043 | 78.40 | 2.13 | 0.00 | ENSG00000198513 | 200.55 | -1.29 | 0.00 |
| ENSG00000196756 | 1716.16 | 2.13 | 0.00 | ENSG00000177469 | 4631.35 | -1.29 | 0.00 |
| ENSG00000175063 | 2992.80 | 2.13 | 0.00 | ENSG00000113448 | 630.88 | -1.29 | 0.00 |
| ENSG00000245648 | 143.08 | 2.13 | 0.00 | ENSG00000178226 | 101.13 | -1.29 | 0.00 |
| ENSG00000125508 | 231.14 | 2.12 | 0.00 | ENSG00000189067 | 5737.61 | -1.29 | 0.00 |
| ENSG00000128564 | 119.89 | 2.12 | 0.00 | ENSG00000139163 | 3083.34 | -1.29 | 0.00 |
| ENSG00000109205 | 126.54 | 2.12 | 0.00 | ENSG00000184349 | 174.77 | -1.29 | 0.00 |
| ENSG00000101057 | 4014.27 | 2.12 | 0.00 | ENSG00000135905 | 312.14 | -1.29 | 0.00 |
| ENSG00000101850 | 276.33 | 2.12 | 0.00 | ENSG00000211658 | 110.52 | -1.29 | 0.00 |
| ENSG00000138795 | 514.73 | 2.11 | 0.00 | ENSG00000141404 | 176.05 | -1.30 | 0.00 |
| ENSG00000141682 | 500.13 | 2.11 | 0.00 | ENSG00000157514 | 1823.67 | -1.30 | 0.00 |
| ENSG00000089327 | 4103.58 | 2.11 | 0.00 | ENSG00000100368 | 480.14 | -1.30 | 0.00 |
| ENSG00000183199 | 98.57 | 2.11 | 0.00 | ENSG00000186204 | 886.21 | -1.30 | 0.00 |
| ENSG00000068650 | 3127.14 | 2.10 | 0.00 | ENSG00000049759 | 2399.63 | -1.30 | 0.00 |
| ENSG00000100453 | 474.45 | 2.10 | 0.00 | ENSG00000104081 | 619.68 | -1.30 | 0.00 |
| ENSG00000146858 | 262.98 | 2.10 | 0.00 | ENSG00000154265 | 621.33 | -1.30 | 0.00 |
| ENSG00000114113 | 177.64 | 2.09 | 0.00 | ENSG00000182578 | 1272.73 | -1.30 | 0.00 |
| ENSG00000142235 | 274.36 | 2.09 | 0.00 | ENSG00000117226 | 2006.68 | -1.30 | 0.00 |
| ENSG00000146054 | 280.72 | 2.09 | 0.00 | ENSG00000185532 | 308.43 | -1.30 | 0.00 |
| ENSG00000197472 | 58.79 | 2.08 | 0.00 | ENSG00000151702 | 306.82 | -1.30 | 0.00 |
| ENSG00000122861 | 2415.54 | 2.08 | 0.00 | ENSG00000168398 | 715.72 | -1.30 | 0.00 |
| ENSG00000197168 | 108.07 | 2.08 | 0.00 | ENSG00000164691 | 204.63 | -1.30 | 0.00 |
| ENSG00000133067 | 963.38 | 2.08 | 0.00 | ENSG00000107954 | 514.52 | -1.30 | 0.00 |
| ENSG00000131019 | 54.75 | 2.08 | 0.00 | ENSG00000225969 | 304.66 | -1.30 | 0.00 |
| ENSG00000205622 | 133.34 | 2.08 | 0.00 | ENSG00000272686 | 193.30 | -1.30 | 0.00 |
| ENSG00000011426 | 1934.03 | 2.08 | 0.00 | ENSG00000117090 | 85.20 | -1.30 | 0.00 |
| ENSG00000112655 | 3461.60 | 2.08 | 0.00 | ENSG00000159399 | 6165.33 | -1.30 | 0.00 |
| ENSG00000116017 | 1620.53 | 2.08 | 0.00 | ENSG00000075239 | 2153.46 | -1.30 | 0.00 |
| ENSG00000276644 | 1165.39 | 2.07 | 0.00 | ENSG00000079257 | 597.31 | -1.30 | 0.00 |
| ENSG00000163584 | 2263.90 | 2.07 | 0.00 | ENSG00000180861 | 1376.53 | -1.30 | 0.00 |
| ENSG00000165480 | 883.62 | 2.07 | 0.00 | ENSG00000163520 | 1752.67 | -1.31 | 0.00 |
| ENSG00000243742 | 91.72 | 2.07 | 0.00 | ENSG00000147027 | 480.20 | -1.31 | 0.00 |
| ENSG00000180389 | 53.07 | 2.06 | 0.00 | ENSG00000104321 | 300.00 | -1.31 | 0.00 |
| ENSG00000049192 | 80.65 | 2.06 | 0.00 | ENSG00000146859 | 544.29 | -1.31 | 0.00 |
| ENSG00000196584 | 303.70 | 2.06 | 0.00 | ENSG00000111684 | 795.28 | -1.31 | 0.00 |
| ENSG00000164620 | 132.06 | 2.05 | 0.00 | ENSG00000160445 | 2054.18 | -1.31 | 0.00 |
| ENSG00000171617 | 5762.67 | 2.05 | 0.00 | ENSG00000145113 | 3631.88 | -1.31 | 0.00 |
| ENSG00000186193 | 2588.27 | 2.05 | 0.00 | ENSG00000110090 | 5467.71 | -1.31 | 0.00 |
| ENSG00000185008 | 203.00 | 2.04 | 0.00 | ENSG00000159733 | 469.99 | -1.31 | 0.00 |
| ENSG00000256940 | 64.50 | 2.04 | 0.00 | ENSG00000134874 | 153.06 | -1.31 | 0.00 |
| ENSG00000182782 | 73.59 | 2.04 | 0.00 | ENSG00000179954 | 504.17 | -1.31 | 0.00 |
| ENSG00000102265 | 10274.42 | 2.03 | 0.00 | ENSG00000187391 | 104.89 | -1.31 | 0.00 |
| ENSG00000152413 | 302.90 | 2.02 | 0.00 | ENSG00000127995 | 731.37 | -1.31 | 0.00 |
| ENSG00000163013 | 740.59 | 2.02 | 0.00 | ENSG00000104936 | 1041.43 | -1.31 | 0.00 |
| ENSG00000074211 | 177.51 | 2.02 | 0.00 | ENSG00000256802 | 75.18 | -1.31 | 0.00 |
| ENSG00000261713 | 75.52 | 2.02 | 0.00 | ENSG00000237070 | 91.32 | -1.31 | 0.00 |
| ENSG00000188176 | 61.98 | 2.02 | 0.00 | ENSG00000104756 | 916.49 | -1.31 | 0.00 |
| ENSG00000255874 | 133.45 | 2.02 | 0.00 | ENSG00000181350 | 865.51 | -1.31 | 0.00 |
| ENSG00000164764 | 548.61 | 2.01 | 0.00 | ENSG00000260314 | 617.43 | -1.32 | 0.00 |
| ENSG00000143217 | 687.44 | 2.01 | 0.00 | ENSG00000146374 | 132.69 | -1.32 | 0.00 |
| ENSG00000107719 | 1607.95 | 2.01 | 0.00 | ENSG00000163694 | 5802.78 | -1.32 | 0.00 |
| ENSG00000169583 | 258.12 | 2.01 | 0.00 | ENSG00000090006 | 5037.37 | -1.32 | 0.00 |
| ENSG00000226396 | 83.79 | 2.00 | 0.00 | ENSG00000169946 | 71.25 | -1.32 | 0.00 |
| ENSG00000017483 | 2934.75 | 2.00 | 0.00 | ENSG00000115112 | 2479.00 | -1.32 | 0.00 |
| ENSG00000196155 | 1477.84 | 2.00 | 0.00 | ENSG00000113389 | 104.97 | -1.32 | 0.00 |
| ENSG00000157551 | 58.41 | 2.00 | 0.00 | ENSG00000053254 | 1582.97 | -1.32 | 0.00 |
| ENSG00000148935 | 81.82 | 2.00 | 0.00 | ENSG00000133574 | 451.72 | -1.32 | 0.00 |
| ENSG00000160932 | 6038.98 | 2.00 | 0.00 | ENSG00000183463 | 300.80 | -1.32 | 0.00 |
| ENSG00000146477 | 758.96 | 1.99 | 0.00 | ENSG00000116704 | 2350.98 | -1.32 | 0.00 |
| ENSG00000164761 | 622.70 | 1.99 | 0.00 | ENSG00000117054 | 1454.34 | -1.32 | 0.00 |
| ENSG00000112877 | 383.22 | 1.99 | 0.00 | ENSG00000143344 | 484.48 | -1.32 | 0.00 |
| ENSG00000102878 | 373.67 | 1.99 | 0.00 | ENSG00000244921 | 57.76 | -1.32 | 0.00 |
| ENSG00000159167 | 494.50 | 1.99 | 0.00 | ENSG00000197892 | 3233.41 | -1.32 | 0.00 |
| ENSG00000091651 | 550.73 | 1.98 | 0.00 | ENSG00000172955 | 343.15 | -1.32 | 0.00 |
| ENSG00000135744 | 967.51 | 1.98 | 0.00 | ENSG00000183801 | 190.99 | -1.32 | 0.00 |
| ENSG00000136689 | 701.16 | 1.98 | 0.00 | ENSG00000109436 | 526.43 | -1.33 | 0.00 |
| ENSG00000144395 | 94.47 | 1.98 | 0.00 | ENSG00000263155 | 144.19 | -1.33 | 0.00 |
| ENSG00000133519 | 209.50 | 1.98 | 0.00 | ENSG00000140285 | 201.50 | -1.33 | 0.00 |
| ENSG00000197696 | 247.10 | 1.97 | 0.00 | ENSG00000104894 | 407.92 | -1.33 | 0.00 |
| ENSG00000163737 | 90.16 | 1.97 | 0.00 | ENSG00000104419 | 14612.08 | -1.33 | 0.00 |
| ENSG00000239467 | 68.43 | 1.97 | 0.00 | ENSG00000182013 | 61.67 | -1.33 | 0.00 |
| ENSG00000135119 | 149.69 | 1.97 | 0.00 | ENSG00000122986 | 120.29 | -1.33 | 0.00 |
| ENSG00000144485 | 2504.39 | 1.96 | 0.00 | ENSG00000109099 | 1810.15 | -1.33 | 0.00 |
| ENSG00000136982 | 510.52 | 1.96 | 0.00 | ENSG00000198453 | 59.78 | -1.33 | 0.00 |
| ENSG00000124602 | 569.97 | 1.96 | 0.00 | ENSG00000183036 | 140.31 | -1.33 | 0.00 |
| ENSG00000215915 | 113.05 | 1.96 | 0.00 | ENSG00000226564 | 55.36 | -1.33 | 0.00 |
| ENSG00000182580 | 7094.48 | 1.96 | 0.00 | ENSG00000279117 | 356.07 | -1.33 | 0.00 |
| ENSG00000228109 | 83.65 | 1.95 | 0.00 | ENSG00000233608 | 50.20 | -1.33 | 0.00 |
| ENSG00000184916 | 1603.97 | 1.95 | 0.00 | ENSG00000183317 | 646.26 | -1.33 | 0.00 |
| ENSG00000226608 | 297.05 | 1.95 | 0.00 | ENSG00000067191 | 230.79 | -1.33 | 0.00 |
| ENSG00000111012 | 146.05 | 1.95 | 0.00 | ENSG00000188906 | 137.45 | -1.33 | 0.00 |
| ENSG00000228887 | 56.23 | 1.95 | 0.00 | ENSG00000166333 | 449.60 | -1.33 | 0.00 |
| ENSG00000225614 | 318.08 | 1.95 | 0.00 | ENSG00000166821 | 490.09 | -1.33 | 0.00 |
| ENSG00000261236 | 4591.38 | 1.95 | 0.00 | ENSG00000003137 | 103.55 | -1.33 | 0.00 |
| ENSG00000125864 | 106.74 | 1.94 | 0.00 | ENSG00000140832 | 1441.30 | -1.34 | 0.00 |
| ENSG00000142661 | 806.79 | 1.94 | 0.00 | ENSG00000139540 | 3240.12 | -1.34 | 0.00 |
| ENSG00000105717 | 127.82 | 1.94 | 0.00 | ENSG00000137501 | 2228.23 | -1.34 | 0.00 |
| ENSG00000188015 | 62.06 | 1.94 | 0.00 | ENSG00000105383 | 64.07 | -1.34 | 0.00 |
| ENSG00000167747 | 4136.31 | 1.94 | 0.00 | ENSG00000155962 | 210.68 | -1.34 | 0.00 |
| ENSG00000106927 | 55.24 | 1.94 | 0.00 | ENSG00000173546 | 849.02 | -1.34 | 0.00 |
| ENSG00000189120 | 925.56 | 1.94 | 0.00 | ENSG00000172543 | 116.50 | -1.34 | 0.00 |
| ENSG00000188290 | 309.55 | 1.94 | 0.00 | ENSG00000143061 | 1003.45 | -1.34 | 0.00 |
| ENSG00000141391 | 120.82 | 1.94 | 0.00 | ENSG00000241399 | 151.88 | -1.34 | 0.00 |
| ENSG00000135451 | 906.11 | 1.93 | 0.00 | ENSG00000198944 | 663.74 | -1.34 | 0.00 |
| ENSG00000026036 | 205.01 | 1.93 | 0.00 | ENSG00000271503 | 781.31 | -1.34 | 0.00 |
| ENSG00000127564 | 1225.98 | 1.93 | 0.00 | ENSG00000154188 | 92.30 | -1.34 | 0.00 |
| ENSG00000165490 | 332.56 | 1.93 | 0.00 | ENSG00000120658 | 50.06 | -1.34 | 0.00 |
| ENSG00000243479 | 144.70 | 1.93 | 0.00 | ENSG00000135828 | 479.46 | -1.34 | 0.00 |
| ENSG00000188483 | 563.74 | 1.93 | 0.00 | ENSG00000146966 | 332.65 | -1.35 | 0.00 |
| ENSG00000163191 | 14955.22 | 1.93 | 0.00 | ENSG00000167912 | 61.98 | -1.35 | 0.00 |
| ENSG00000147689 | 77.13 | 1.93 | 0.00 | ENSG00000120156 | 227.06 | -1.35 | 0.00 |
| ENSG00000197582 | 102.79 | 1.93 | 0.00 | ENSG00000103544 | 925.50 | -1.35 | 0.00 |
| ENSG00000160401 | 113.94 | 1.93 | 0.00 | ENSG00000134245 | 226.92 | -1.35 | 0.00 |
| ENSG00000211448 | 350.19 | 1.92 | 0.00 | ENSG00000162458 | 3339.90 | -1.35 | 0.00 |
| ENSG00000100228 | 379.00 | 1.92 | 0.00 | ENSG00000185909 | 329.03 | -1.35 | 0.00 |
| ENSG00000115255 | 878.70 | 1.92 | 0.00 | ENSG00000122707 | 207.48 | -1.35 | 0.00 |
| ENSG00000141756 | 3684.30 | 1.92 | 0.00 | ENSG00000121297 | 215.97 | -1.35 | 0.00 |
| ENSG00000183856 | 2360.47 | 1.92 | 0.00 | ENSG00000262655 | 1699.93 | -1.35 | 0.00 |
| ENSG00000106366 | 1496.95 | 1.92 | 0.00 | ENSG00000104814 | 186.71 | -1.35 | 0.00 |
| ENSG00000109321 | 3235.49 | 1.92 | 0.00 | ENSG00000153283 | 161.91 | -1.35 | 0.00 |
| ENSG00000136997 | 9824.31 | 1.92 | 0.00 | ENSG00000104880 | 389.32 | -1.35 | 0.00 |
| ENSG00000188322 | 250.37 | 1.92 | 0.00 | ENSG00000036473 | 125.97 | -1.35 | 0.00 |
| ENSG00000276043 | 888.35 | 1.91 | 0.00 | ENSG00000072133 | 262.18 | -1.35 | 0.00 |
| ENSG00000179241 | 513.58 | 1.91 | 0.00 | ENSG00000135124 | 1597.27 | -1.36 | 0.00 |
| ENSG00000125378 | 2667.23 | 1.91 | 0.00 | ENSG00000164976 | 3016.32 | -1.36 | 0.00 |
| ENSG00000246898 | 163.88 | 1.90 | 0.00 | ENSG00000139874 | 278.79 | -1.36 | 0.00 |
| ENSG00000229891 | 167.29 | 1.90 | 0.00 | ENSG00000171433 | 67.66 | -1.36 | 0.00 |
| ENSG00000139618 | 545.66 | 1.90 | 0.00 | ENSG00000071073 | 2268.61 | -1.36 | 0.00 |
| ENSG00000158352 | 858.49 | 1.90 | 0.00 | ENSG00000139946 | 384.82 | -1.36 | 0.00 |
| ENSG00000122691 | 101.00 | 1.90 | 0.00 | ENSG00000005469 | 525.15 | -1.36 | 0.00 |
| ENSG00000228205 | 162.95 | 1.90 | 0.00 | ENSG00000111913 | 184.48 | -1.36 | 0.00 |
| ENSG00000081059 | 1822.56 | 1.90 | 0.00 | ENSG00000198189 | 4049.66 | -1.36 | 0.00 |
| ENSG00000182324 | 86.91 | 1.90 | 0.00 | ENSG00000105246 | 62.02 | -1.36 | 0.00 |
| ENSG00000211892 | 6928.07 | 1.90 | 0.00 | ENSG00000046889 | 105.60 | -1.36 | 0.00 |
| ENSG00000204410 | 168.19 | 1.90 | 0.00 | ENSG00000167261 | 61.76 | -1.36 | 0.00 |
| ENSG00000196950 | 1332.51 | 1.89 | 0.00 | ENSG00000175287 | 72.68 | -1.36 | 0.00 |
| ENSG00000197757 | 71.79 | 1.89 | 0.00 | ENSG00000181631 | 96.71 | -1.36 | 0.00 |
| ENSG00000169876 | 3045.64 | 1.89 | 0.00 | ENSG00000280411 | 441.92 | -1.37 | 0.00 |
| ENSG00000134323 | 99.85 | 1.89 | 0.00 | ENSG00000172340 | 4606.77 | -1.37 | 0.00 |
| ENSG00000239528 | 83.55 | 1.89 | 0.00 | ENSG00000064652 | 212.32 | -1.37 | 0.00 |
| ENSG00000101412 | 1193.05 | 1.89 | 0.00 | ENSG00000110324 | 576.53 | -1.37 | 0.00 |
| ENSG00000151846 | 84.50 | 1.89 | 0.00 | ENSG00000254395 | 102.78 | -1.37 | 0.00 |
| ENSG00000118707 | 1370.84 | 1.89 | 0.00 | ENSG00000163293 | 1024.34 | -1.37 | 0.00 |
| ENSG00000176170 | 499.45 | 1.89 | 0.00 | ENSG00000110077 | 756.95 | -1.37 | 0.00 |
| ENSG00000243716 | 164.30 | 1.88 | 0.00 | ENSG00000155846 | 704.15 | -1.37 | 0.00 |
| ENSG00000092445 | 513.63 | 1.88 | 0.00 | ENSG00000175899 | 10202.13 | -1.37 | 0.00 |
| ENSG00000226085 | 50.21 | 1.87 | 0.00 | ENSG00000143382 | 286.56 | -1.37 | 0.00 |
| ENSG00000125398 | 8477.44 | 1.87 | 0.00 | ENSG00000064886 | 50.12 | -1.37 | 0.00 |
| ENSG00000181544 | 74.64 | 1.87 | 0.00 | ENSG00000124406 | 1235.87 | -1.37 | 0.00 |
| ENSG00000181218 | 447.12 | 1.87 | 0.00 | ENSG00000137825 | 577.76 | -1.37 | 0.00 |
| ENSG00000146677 | 194.36 | 1.87 | 0.00 | ENSG00000198721 | 1073.55 | -1.37 | 0.00 |
| ENSG00000106089 | 388.58 | 1.87 | 0.00 | ENSG00000005102 | 75.08 | -1.37 | 0.00 |
| ENSG00000137809 | 1068.55 | 1.87 | 0.00 | ENSG00000182963 | 210.93 | -1.38 | 0.00 |
| ENSG00000196739 | 818.30 | 1.86 | 0.00 | ENSG00000184163 | 161.33 | -1.38 | 0.00 |
| ENSG00000146038 | 147.11 | 1.86 | 0.00 | ENSG00000123612 | 232.37 | -1.38 | 0.00 |
| ENSG00000165891 | 282.50 | 1.86 | 0.00 | ENSG00000178695 | 2043.58 | -1.38 | 0.00 |
| ENSG00000175602 | 1339.54 | 1.86 | 0.00 | ENSG00000148584 | 907.77 | -1.38 | 0.00 |
| ENSG00000137571 | 143.19 | 1.86 | 0.00 | ENSG00000172367 | 305.83 | -1.38 | 0.00 |
| ENSG00000075702 | 581.77 | 1.86 | 0.00 | ENSG00000254838 | 99.34 | -1.38 | 0.00 |
| ENSG00000163975 | 2059.76 | 1.86 | 0.00 | ENSG00000107282 | 226.52 | -1.38 | 0.00 |
| ENSG00000197275 | 136.86 | 1.86 | 0.00 | ENSG00000205413 | 824.08 | -1.38 | 0.00 |
| ENSG00000062282 | 1122.97 | 1.85 | 0.00 | ENSG00000198894 | 1047.69 | -1.38 | 0.00 |
| ENSG00000159423 | 1023.71 | 1.85 | 0.00 | ENSG00000107317 | 653.62 | -1.38 | 0.00 |
| ENSG00000122034 | 6749.09 | 1.85 | 0.00 | ENSG00000139626 | 205.55 | -1.38 | 0.00 |
| ENSG00000089723 | 253.50 | 1.85 | 0.00 | ENSG00000025423 | 92.25 | -1.38 | 0.00 |
| ENSG00000156427 | 72.45 | 1.85 | 0.00 | ENSG00000121413 | 298.84 | -1.38 | 0.00 |
| ENSG00000277363 | 403.61 | 1.85 | 0.00 | ENSG00000042980 | 300.20 | -1.38 | 0.00 |
| ENSG00000088325 | 4410.86 | 1.85 | 0.00 | ENSG00000091622 | 590.97 | -1.39 | 0.00 |
| ENSG00000127586 | 1176.83 | 1.85 | 0.00 | ENSG00000165795 | 2824.45 | -1.39 | 0.00 |
| ENSG00000105976 | 5608.88 | 1.85 | 0.00 | ENSG00000101695 | 366.97 | -1.39 | 0.00 |
| ENSG00000226812 | 291.12 | 1.84 | 0.00 | ENSG00000107736 | 65.87 | -1.39 | 0.00 |
| ENSG00000111799 | 7580.59 | 1.84 | 0.00 | ENSG00000184012 | 7958.40 | -1.39 | 0.00 |
| ENSG00000106178 | 1263.58 | 1.84 | 0.00 | ENSG00000116678 | 221.42 | -1.39 | 0.00 |
| ENSG00000124466 | 142.02 | 1.84 | 0.00 | ENSG00000157570 | 515.49 | -1.39 | 0.00 |
| ENSG00000124225 | 6822.11 | 1.84 | 0.00 | ENSG00000171174 | 172.89 | -1.39 | 0.00 |
| ENSG00000128342 | 1210.75 | 1.83 | 0.00 | ENSG00000117016 | 275.36 | -1.39 | 0.00 |
| ENSG00000134824 | 1801.28 | 1.83 | 0.00 | ENSG00000064309 | 179.61 | -1.39 | 0.00 |
| ENSG00000108106 | 1346.35 | 1.83 | 0.00 | ENSG00000131845 | 119.25 | -1.39 | 0.00 |
| ENSG00000230076 | 163.12 | 1.83 | 0.00 | ENSG00000173372 | 2652.97 | -1.39 | 0.00 |
| ENSG00000121904 | 67.29 | 1.83 | 0.00 | ENSG00000197594 | 363.81 | -1.39 | 0.00 |
| ENSG00000133119 | 1243.46 | 1.83 | 0.00 | ENSG00000143001 | 61.24 | -1.39 | 0.00 |
| ENSG00000139629 | 2759.54 | 1.83 | 0.00 | ENSG00000169122 | 63.67 | -1.39 | 0.00 |
| ENSG00000182472 | 225.05 | 1.83 | 0.00 | ENSG00000144426 | 382.14 | -1.39 | 0.00 |
| ENSG00000163689 | 131.78 | 1.83 | 0.00 | ENSG00000103056 | 1495.22 | -1.39 | 0.00 |
| ENSG00000114346 | 2996.21 | 1.82 | 0.00 | ENSG00000115380 | 1160.02 | -1.39 | 0.00 |
| ENSG00000218175 | 412.72 | 1.82 | 0.00 | ENSG00000166165 | 23644.88 | -1.40 | 0.00 |
| ENSG00000218227 | 175.97 | 1.82 | 0.00 | ENSG00000166780 | 270.09 | -1.40 | 0.00 |
| ENSG00000233913 | 582.30 | 1.82 | 0.00 | ENSG00000165548 | 310.68 | -1.40 | 0.00 |
| ENSG00000232956 | 962.98 | 1.82 | 0.00 | ENSG00000095203 | 3794.43 | -1.40 | 0.00 |
| ENSG00000149380 | 91.54 | 1.82 | 0.00 | ENSG00000151338 | 172.03 | -1.40 | 0.00 |
| ENSG00000160345 | 76.61 | 1.82 | 0.00 | ENSG00000198961 | 2539.18 | -1.40 | 0.00 |
| ENSG00000169903 | 112.92 | 1.82 | 0.00 | ENSG00000183580 | 193.52 | -1.40 | 0.00 |
| ENSG00000162073 | 1133.81 | 1.82 | 0.00 | ENSG00000188921 | 199.63 | -1.40 | 0.00 |
| ENSG00000146411 | 250.59 | 1.81 | 0.00 | ENSG00000169554 | 742.92 | -1.40 | 0.00 |
| ENSG00000171877 | 267.10 | 1.81 | 0.00 | ENSG00000215018 | 127.52 | -1.40 | 0.00 |
| ENSG00000184428 | 2433.14 | 1.81 | 0.00 | ENSG00000133116 | 56.09 | -1.41 | 0.00 |
| ENSG00000117707 | 1285.23 | 1.81 | 0.00 | ENSG00000112182 | 61.97 | -1.41 | 0.00 |
| ENSG00000232442 | 214.57 | 1.81 | 0.00 | ENSG00000134184 | 192.02 | -1.41 | 0.00 |
| ENSG00000151617 | 451.22 | 1.81 | 0.00 | ENSG00000156050 | 126.19 | -1.41 | 0.00 |
| ENSG00000170827 | 190.81 | 1.80 | 0.00 | ENSG00000070759 | 280.14 | -1.41 | 0.00 |
| ENSG00000138074 | 4082.40 | 1.80 | 0.00 | ENSG00000070526 | 5222.31 | -1.41 | 0.00 |
| ENSG00000177410 | 4665.35 | 1.80 | 0.00 | ENSG00000187955 | 1508.98 | -1.41 | 0.00 |
| ENSG00000186185 | 816.90 | 1.80 | 0.00 | ENSG00000117215 | 91.31 | -1.41 | 0.00 |
| ENSG00000182118 | 398.14 | 1.80 | 0.00 | ENSG00000185630 | 1534.63 | -1.42 | 0.00 |
| ENSG00000196793 | 238.55 | 1.80 | 0.00 | ENSG00000153064 | 115.76 | -1.42 | 0.00 |
| ENSG00000099954 | 73.28 | 1.80 | 0.00 | ENSG00000185905 | 111.18 | -1.42 | 0.00 |
| ENSG00000070882 | 1418.21 | 1.80 | 0.00 | ENSG00000163249 | 859.36 | -1.42 | 0.00 |
| ENSG00000273319 | 62.30 | 1.80 | 0.00 | ENSG00000114859 | 1458.77 | -1.42 | 0.00 |
| ENSG00000137573 | 6559.70 | 1.79 | 0.00 | ENSG00000093072 | 1080.57 | -1.42 | 0.00 |
| ENSG00000103021 | 370.27 | 1.79 | 0.00 | ENSG00000167705 | 470.55 | -1.42 | 0.00 |
| ENSG00000060656 | 647.10 | 1.79 | 0.00 | ENSG00000103241 | 605.94 | -1.42 | 0.00 |
| ENSG00000188372 | 471.74 | 1.78 | 0.00 | ENSG00000171100 | 778.08 | -1.42 | 0.00 |
| ENSG00000214110 | 210.97 | 1.78 | 0.00 | ENSG00000211659 | 1027.79 | -1.42 | 0.00 |
| ENSG00000135253 | 109.46 | 1.78 | 0.00 | ENSG00000139725 | 238.87 | -1.42 | 0.00 |
| ENSG00000183018 | 2636.06 | 1.78 | 0.00 | ENSG00000140092 | 559.19 | -1.42 | 0.00 |
| ENSG00000118193 | 391.30 | 1.78 | 0.00 | ENSG00000050405 | 6254.35 | -1.42 | 0.00 |
| ENSG00000161249 | 605.49 | 1.78 | 0.00 | ENSG00000187068 | 384.25 | -1.42 | 0.00 |
| ENSG00000117394 | 9432.89 | 1.77 | 0.00 | ENSG00000130592 | 1257.84 | -1.43 | 0.00 |
| ENSG00000073464 | 344.16 | 1.77 | 0.00 | ENSG00000274576 | 118.04 | -1.43 | 0.00 |
| ENSG00000143590 | 722.92 | 1.77 | 0.00 | ENSG00000172156 | 184.02 | -1.43 | 0.00 |
| ENSG00000255717 | 1752.60 | 1.77 | 0.00 | ENSG00000069764 | 132.79 | -1.43 | 0.00 |
| ENSG00000117877 | 309.07 | 1.77 | 0.00 | ENSG00000119865 | 162.81 | -1.43 | 0.00 |
| ENSG00000183010 | 4450.90 | 1.77 | 0.00 | ENSG00000169851 | 373.08 | -1.43 | 0.00 |
| ENSG00000224411 | 52.86 | 1.77 | 0.00 | ENSG00000165757 | 721.53 | -1.43 | 0.00 |
| ENSG00000095739 | 735.45 | 1.76 | 0.00 | ENSG00000122188 | 74.06 | -1.43 | 0.00 |
| ENSG00000261685 | 472.46 | 1.76 | 0.00 | ENSG00000165959 | 2007.30 | -1.43 | 0.00 |
| ENSG00000276023 | 539.62 | 1.76 | 0.00 | ENSG00000184785 | 70.88 | -1.43 | 0.00 |
| ENSG00000158806 | 81.57 | 1.76 | 0.00 | ENSG00000204175 | 326.93 | -1.43 | 0.00 |
| ENSG00000025772 | 3212.13 | 1.76 | 0.00 | ENSG00000101210 | 80.92 | -1.43 | 0.00 |
| ENSG00000178429 | 109.92 | 1.76 | 0.00 | ENSG00000107864 | 186.84 | -1.44 | 0.00 |
| ENSG00000243829 | 62.80 | 1.76 | 0.00 | ENSG00000205277 | 6987.34 | -1.44 | 0.00 |
| ENSG00000237361 | 132.18 | 1.76 | 0.00 | ENSG00000198846 | 390.40 | -1.44 | 0.00 |
| ENSG00000237857 | 88.58 | 1.75 | 0.00 | ENSG00000131711 | 522.95 | -1.44 | 0.00 |
| ENSG00000123989 | 6084.38 | 1.75 | 0.00 | ENSG00000171777 | 53.34 | -1.44 | 0.00 |
| ENSG00000162062 | 320.32 | 1.75 | 0.00 | ENSG00000280237 | 129.48 | -1.44 | 0.00 |
| ENSG00000101003 | 1127.32 | 1.75 | 0.00 | ENSG00000145012 | 4659.69 | -1.44 | 0.00 |
| ENSG00000217130 | 63.44 | 1.75 | 0.00 | ENSG00000111885 | 2305.75 | -1.44 | 0.00 |
| ENSG00000185842 | 215.73 | 1.75 | 0.00 | ENSG00000100154 | 318.77 | -1.44 | 0.00 |
| ENSG00000101074 | 59.05 | 1.75 | 0.00 | ENSG00000167037 | 107.76 | -1.44 | 0.00 |
| ENSG00000172456 | 1247.39 | 1.75 | 0.00 | ENSG00000171428 | 408.53 | -1.44 | 0.00 |
| ENSG00000184160 | 165.26 | 1.75 | 0.00 | ENSG00000119514 | 1749.26 | -1.44 | 0.00 |
| ENSG00000272235 | 54.01 | 1.75 | 0.00 | ENSG00000149289 | 428.46 | -1.44 | 0.00 |
| ENSG00000169245 | 956.97 | 1.74 | 0.00 | ENSG00000251615 | 50.06 | -1.44 | 0.00 |
| ENSG00000213886 | 584.17 | 1.74 | 0.00 | ENSG00000139508 | 1085.89 | -1.44 | 0.00 |
| ENSG00000165443 | 85.61 | 1.74 | 0.00 | ENSG00000163531 | 447.41 | -1.44 | 0.00 |
| ENSG00000181634 | 576.52 | 1.74 | 0.00 | ENSG00000255248 | 161.37 | -1.45 | 0.00 |
| ENSG00000143228 | 557.43 | 1.74 | 0.00 | ENSG00000078018 | 67.57 | -1.45 | 0.00 |
| ENSG00000226419 | 126.54 | 1.73 | 0.00 | ENSG00000136160 | 352.31 | -1.45 | 0.00 |
| ENSG00000204262 | 6045.90 | 1.73 | 0.00 | ENSG00000121440 | 592.65 | -1.45 | 0.00 |
| ENSG00000169436 | 56.65 | 1.73 | 0.00 | ENSG00000185345 | 62.93 | -1.45 | 0.00 |
| ENSG00000168993 | 268.14 | 1.73 | 0.00 | ENSG00000164330 | 111.20 | -1.45 | 0.00 |
| ENSG00000197927 | 81.95 | 1.73 | 0.00 | ENSG00000259674 | 90.43 | -1.45 | 0.00 |
| ENSG00000214078 | 7900.00 | 1.73 | 0.00 | ENSG00000134256 | 81.59 | -1.45 | 0.00 |
| ENSG00000110881 | 608.62 | 1.73 | 0.00 | ENSG00000203497 | 51.54 | -1.46 | 0.00 |
| ENSG00000281398 | 197.50 | 1.73 | 0.00 | ENSG00000183578 | 124.09 | -1.46 | 0.00 |
| ENSG00000262406 | 2235.62 | 1.73 | 0.00 | ENSG00000213366 | 153.74 | -1.46 | 0.00 |
| ENSG00000011347 | 2297.39 | 1.73 | 0.00 | ENSG00000137936 | 719.66 | -1.46 | 0.00 |
| ENSG00000185615 | 70.21 | 1.73 | 0.00 | ENSG00000165406 | 1236.71 | -1.46 | 0.00 |
| ENSG00000186907 | 53.37 | 1.73 | 0.00 | ENSG00000181751 | 1027.99 | -1.46 | 0.00 |
| ENSG00000239672 | 2737.83 | 1.73 | 0.00 | ENSG00000090020 | 2665.93 | -1.46 | 0.00 |
| ENSG00000108342 | 105.51 | 1.73 | 0.00 | ENSG00000204219 | 2241.66 | -1.46 | 0.00 |
| ENSG00000186871 | 307.89 | 1.73 | 0.00 | ENSG00000136960 | 675.55 | -1.46 | 0.00 |
| ENSG00000223705 | 549.76 | 1.72 | 0.00 | ENSG00000117592 | 7149.45 | -1.46 | 0.00 |
| ENSG00000278709 | 102.29 | 1.72 | 0.00 | ENSG00000149260 | 6330.28 | -1.46 | 0.00 |
| ENSG00000203688 | 91.65 | 1.72 | 0.00 | ENSG00000239819 | 76.55 | -1.46 | 0.00 |
| ENSG00000186648 | 109.09 | 1.72 | 0.00 | ENSG00000197872 | 199.88 | -1.47 | 0.00 |
| ENSG00000069011 | 1787.43 | 1.72 | 0.00 | ENSG00000150995 | 474.85 | -1.47 | 0.00 |
| ENSG00000243449 | 615.59 | 1.72 | 0.00 | ENSG00000260401 | 186.55 | -1.47 | 0.00 |
| ENSG00000171346 | 300.64 | 1.72 | 0.00 | ENSG00000089356 | 20423.38 | -1.47 | 0.00 |
| ENSG00000140511 | 592.60 | 1.72 | 0.00 | ENSG00000152495 | 88.79 | -1.47 | 0.00 |
| ENSG00000065328 | 471.46 | 1.71 | 0.00 | ENSG00000165995 | 139.86 | -1.47 | 0.00 |
| ENSG00000136383 | 754.51 | 1.71 | 0.00 | ENSG00000140682 | 1129.55 | -1.47 | 0.00 |
| ENSG00000160182 | 5349.35 | 1.71 | 0.00 | ENSG00000174600 | 319.50 | -1.47 | 0.00 |
| ENSG00000141570 | 556.10 | 1.71 | 0.00 | ENSG00000185862 | 306.98 | -1.47 | 0.00 |
| ENSG00000172167 | 256.45 | 1.71 | 0.00 | ENSG00000178075 | 237.81 | -1.47 | 0.00 |
| ENSG00000104524 | 1176.40 | 1.71 | 0.00 | ENSG00000249669 | 135.87 | -1.47 | 0.00 |
| ENSG00000117148 | 52.24 | 1.70 | 0.00 | ENSG00000028277 | 181.36 | -1.47 | 0.00 |
| ENSG00000123485 | 878.60 | 1.70 | 0.00 | ENSG00000110002 | 1527.66 | -1.47 | 0.00 |
| ENSG00000104369 | 1059.10 | 1.70 | 0.00 | ENSG00000166927 | 395.14 | -1.48 | 0.00 |
| ENSG00000182584 | 303.30 | 1.70 | 0.00 | ENSG00000136059 | 2700.96 | -1.48 | 0.00 |
| ENSG00000093009 | 767.21 | 1.70 | 0.00 | ENSG00000079739 | 2767.83 | -1.48 | 0.00 |
| ENSG00000154920 | 158.49 | 1.70 | 0.00 | ENSG00000120756 | 7288.73 | -1.48 | 0.00 |
| ENSG00000105948 | 317.69 | 1.70 | 0.00 | ENSG00000187446 | 6117.33 | -1.48 | 0.00 |
| ENSG00000104549 | 3311.20 | 1.69 | 0.00 | ENSG00000120915 | 1782.67 | -1.48 | 0.00 |
| ENSG00000144354 | 5169.55 | 1.69 | 0.00 | ENSG00000275718 | 195.41 | -1.48 | 0.00 |
| ENSG00000124216 | 223.73 | 1.69 | 0.00 | ENSG00000158517 | 83.02 | -1.48 | 0.00 |
| ENSG00000151790 | 184.60 | 1.69 | 0.00 | ENSG00000164406 | 59.35 | -1.48 | 0.00 |
| ENSG00000144810 | 1316.68 | 1.69 | 0.00 | ENSG00000176273 | 403.26 | -1.48 | 0.00 |
| ENSG00000083807 | 373.55 | 1.69 | 0.00 | ENSG00000127955 | 498.85 | -1.48 | 0.00 |
| ENSG00000198732 | 366.15 | 1.69 | 0.00 | ENSG00000179841 | 211.03 | -1.48 | 0.00 |
| ENSG00000167880 | 4427.71 | 1.69 | 0.00 | ENSG00000131018 | 693.30 | -1.49 | 0.00 |
| ENSG00000110092 | 7929.37 | 1.69 | 0.00 | ENSG00000256262 | 54.79 | -1.49 | 0.00 |
| ENSG00000078814 | 173.97 | 1.69 | 0.00 | ENSG00000143375 | 4656.00 | -1.49 | 0.00 |
| ENSG00000133985 | 361.91 | 1.69 | 0.00 | ENSG00000213713 | 54.06 | -1.49 | 0.00 |
| ENSG00000273066 | 166.31 | 1.69 | 0.00 | ENSG00000079102 | 120.98 | -1.49 | 0.00 |
| ENSG00000236144 | 331.39 | 1.69 | 0.00 | ENSG00000157184 | 1309.34 | -1.49 | 0.00 |
| ENSG00000168268 | 2579.58 | 1.68 | 0.00 | ENSG00000149582 | 161.98 | -1.49 | 0.00 |
| ENSG00000258947 | 109.21 | 1.68 | 0.00 | ENSG00000259330 | 210.36 | -1.49 | 0.00 |
| ENSG00000187961 | 287.39 | 1.68 | 0.00 | ENSG00000240225 | 74.07 | -1.49 | 0.00 |
| ENSG00000148735 | 1184.14 | 1.68 | 0.00 | ENSG00000129116 | 4642.24 | -1.49 | 0.00 |
| ENSG00000126709 | 4036.09 | 1.68 | 0.00 | ENSG00000111452 | 125.77 | -1.49 | 0.00 |
| ENSG00000065923 | 453.24 | 1.68 | 0.00 | ENSG00000119782 | 77.99 | -1.49 | 0.00 |
| ENSG00000142544 | 283.52 | 1.68 | 0.00 | ENSG00000135114 | 633.29 | -1.50 | 0.00 |
| ENSG00000087495 | 98.67 | 1.68 | 0.00 | ENSG00000255774 | 155.38 | -1.50 | 0.00 |
| ENSG00000185885 | 10049.37 | 1.68 | 0.00 | ENSG00000161281 | 162.24 | -1.50 | 0.00 |
| ENSG00000230897 | 82.24 | 1.68 | 0.00 | ENSG00000171451 | 56.86 | -1.50 | 0.00 |
| ENSG00000091127 | 1123.82 | 1.68 | 0.00 | ENSG00000124212 | 378.71 | -1.50 | 0.00 |
| ENSG00000162591 | 700.06 | 1.67 | 0.00 | ENSG00000211677 | 5254.01 | -1.50 | 0.00 |
| ENSG00000276672 | 70.28 | 1.67 | 0.00 | ENSG00000172578 | 234.10 | -1.50 | 0.00 |
| ENSG00000242299 | 582.92 | 1.67 | 0.00 | ENSG00000115252 | 119.04 | -1.50 | 0.00 |
| ENSG00000132000 | 244.21 | 1.67 | 0.00 | ENSG00000170954 | 88.04 | -1.50 | 0.00 |
| ENSG00000094804 | 1377.82 | 1.66 | 0.00 | ENSG00000133104 | 343.13 | -1.50 | 0.00 |
| ENSG00000101444 | 17690.74 | 1.66 | 0.00 | ENSG00000235863 | 429.70 | -1.51 | 0.00 |
| ENSG00000172061 | 493.89 | 1.66 | 0.00 | ENSG00000165178 | 54.29 | -1.51 | 0.00 |
| ENSG00000250982 | 67.45 | 1.66 | 0.00 | ENSG00000110013 | 3691.16 | -1.51 | 0.00 |
| ENSG00000234782 | 79.31 | 1.66 | 0.00 | ENSG00000166143 | 1470.23 | -1.51 | 0.00 |
| ENSG00000225138 | 1091.24 | 1.65 | 0.00 | ENSG00000185585 | 617.59 | -1.51 | 0.00 |
| ENSG00000184497 | 316.26 | 1.65 | 0.00 | ENSG00000162104 | 982.46 | -1.51 | 0.00 |
| ENSG00000167550 | 71.49 | 1.65 | 0.00 | ENSG00000152642 | 2117.72 | -1.51 | 0.00 |
| ENSG00000138772 | 3477.10 | 1.65 | 0.00 | ENSG00000065809 | 1788.69 | -1.51 | 0.00 |
| ENSG00000103269 | 185.93 | 1.65 | 0.00 | ENSG00000166086 | 514.55 | -1.51 | 0.00 |
| ENSG00000124249 | 56.14 | 1.65 | 0.00 | ENSG00000177181 | 469.05 | -1.51 | 0.00 |
| ENSG00000130748 | 508.57 | 1.65 | 0.00 | ENSG00000144857 | 313.59 | -1.51 | 0.00 |
| ENSG00000085465 | 202.59 | 1.65 | 0.00 | ENSG00000137767 | 3580.64 | -1.51 | 0.00 |
| ENSG00000162493 | 904.80 | 1.64 | 0.00 | ENSG00000023902 | 950.03 | -1.51 | 0.00 |
| ENSG00000138180 | 1368.50 | 1.64 | 0.00 | ENSG00000107186 | 238.56 | -1.51 | 0.00 |
| ENSG00000124766 | 5396.41 | 1.64 | 0.00 | ENSG00000133561 | 403.19 | -1.51 | 0.00 |
| ENSG00000124743 | 55.79 | 1.64 | 0.00 | ENSG00000179094 | 968.61 | -1.51 | 0.00 |
| ENSG00000104783 | 3553.23 | 1.64 | 0.00 | ENSG00000187837 | 821.56 | -1.51 | 0.00 |
| ENSG00000131187 | 762.86 | 1.64 | 0.00 | ENSG00000160685 | 3276.01 | -1.52 | 0.00 |
| ENSG00000134297 | 101.29 | 1.64 | 0.00 | ENSG00000010671 | 151.25 | -1.52 | 0.00 |
| ENSG00000213889 | 77.87 | 1.63 | 0.00 | ENSG00000074416 | 6473.88 | -1.52 | 0.00 |
| ENSG00000275437 | 89.11 | 1.63 | 0.00 | ENSG00000131401 | 154.61 | -1.52 | 0.00 |
| ENSG00000182557 | 119.99 | 1.63 | 0.00 | ENSG00000204385 | 11469.99 | -1.52 | 0.00 |
| ENSG00000253522 | 119.07 | 1.63 | 0.00 | ENSG00000157168 | 93.30 | -1.52 | 0.00 |
| ENSG00000228929 | 92.11 | 1.63 | 0.00 | ENSG00000128311 | 6289.92 | -1.52 | 0.00 |
| ENSG00000177839 | 77.37 | 1.63 | 0.00 | ENSG00000167107 | 1346.30 | -1.53 | 0.00 |
| ENSG00000181392 | 307.51 | 1.63 | 0.00 | ENSG00000167077 | 104.95 | -1.53 | 0.00 |
| ENSG00000092621 | 2153.15 | 1.63 | 0.00 | ENSG00000143297 | 157.87 | -1.53 | 0.00 |
| ENSG00000079462 | 1726.58 | 1.62 | 0.00 | ENSG00000162722 | 51.06 | -1.53 | 0.00 |
| ENSG00000109084 | 2580.01 | 1.62 | 0.00 | ENSG00000137266 | 1475.90 | -1.53 | 0.00 |
| ENSG00000139734 | 651.44 | 1.62 | 0.00 | ENSG00000137251 | 285.15 | -1.53 | 0.00 |
| ENSG00000102445 | 3104.47 | 1.62 | 0.00 | ENSG00000070778 | 599.35 | -1.53 | 0.00 |
| ENSG00000101189 | 1427.77 | 1.62 | 0.00 | ENSG00000115556 | 112.52 | -1.53 | 0.00 |
| ENSG00000040608 | 319.25 | 1.62 | 0.00 | ENSG00000119508 | 193.52 | -1.53 | 0.00 |
| ENSG00000106348 | 3386.41 | 1.62 | 0.00 | ENSG00000145649 | 251.96 | -1.54 | 0.00 |
| ENSG00000179772 | 70.92 | 1.62 | 0.00 | ENSG00000158828 | 894.27 | -1.54 | 0.00 |
| ENSG00000105926 | 340.92 | 1.62 | 0.00 | ENSG00000121858 | 2658.40 | -1.54 | 0.00 |
| ENSG00000128965 | 344.51 | 1.62 | 0.00 | ENSG00000235098 | 65.36 | -1.54 | 0.00 |
| ENSG00000104044 | 56.98 | 1.62 | 0.00 | ENSG00000116574 | 1256.23 | -1.54 | 0.00 |
| ENSG00000235703 | 80.26 | 1.62 | 0.00 | ENSG00000135773 | 401.60 | -1.54 | 0.00 |
| ENSG00000113356 | 192.74 | 1.62 | 0.00 | ENSG00000103175 | 381.39 | -1.54 | 0.00 |
| ENSG00000134013 | 2140.17 | 1.62 | 0.00 | ENSG00000101782 | 2855.95 | -1.54 | 0.00 |
| ENSG00000115009 | 2061.75 | 1.62 | 0.00 | ENSG00000244437 | 924.27 | -1.54 | 0.00 |
| ENSG00000164816 | 701.09 | 1.62 | 0.00 | ENSG00000152284 | 214.10 | -1.54 | 0.00 |
| ENSG00000164220 | 229.88 | 1.62 | 0.00 | ENSG00000170915 | 3346.23 | -1.55 | 0.00 |
| ENSG00000105173 | 466.54 | 1.62 | 0.00 | ENSG00000169116 | 6763.52 | -1.55 | 0.00 |
| ENSG00000061337 | 191.16 | 1.61 | 0.00 | ENSG00000010327 | 1777.79 | -1.55 | 0.00 |
| ENSG00000075618 | 2425.24 | 1.61 | 0.00 | ENSG00000105290 | 59.24 | -1.55 | 0.00 |
| ENSG00000100373 | 77.09 | 1.61 | 0.00 | ENSG00000162894 | 189.92 | -1.55 | 0.00 |
| ENSG00000181029 | 99.00 | 1.61 | 0.00 | ENSG00000137941 | 87.93 | -1.55 | 0.00 |
| ENSG00000240376 | 266.85 | 1.61 | 0.00 | ENSG00000172794 | 175.69 | -1.55 | 0.00 |
| ENSG00000134070 | 467.69 | 1.60 | 0.00 | ENSG00000185811 | 345.92 | -1.55 | 0.00 |
| ENSG00000088305 | 220.38 | 1.60 | 0.00 | ENSG00000196517 | 708.00 | -1.55 | 0.00 |
| ENSG00000181649 | 1729.80 | 1.60 | 0.00 | ENSG00000182575 | 121.95 | -1.55 | 0.00 |
| ENSG00000280734 | 215.73 | 1.60 | 0.00 | ENSG00000168077 | 326.64 | -1.55 | 0.00 |
| ENSG00000140451 | 267.48 | 1.60 | 0.00 | ENSG00000120162 | 1207.67 | -1.56 | 0.00 |
| ENSG00000156802 | 1785.09 | 1.60 | 0.00 | ENSG00000149557 | 176.75 | -1.56 | 0.00 |
| ENSG00000235888 | 62.03 | 1.60 | 0.00 | ENSG00000152503 | 318.60 | -1.56 | 0.00 |
| ENSG00000166123 | 2758.15 | 1.60 | 0.00 | ENSG00000198910 | 536.35 | -1.56 | 0.00 |
| ENSG00000164362 | 90.69 | 1.60 | 0.00 | ENSG00000141447 | 785.07 | -1.56 | 0.00 |
| ENSG00000107833 | 1500.98 | 1.60 | 0.00 | ENSG00000185483 | 114.29 | -1.56 | 0.00 |
| ENSG00000120659 | 121.60 | 1.60 | 0.00 | ENSG00000112425 | 142.89 | -1.56 | 0.00 |
| ENSG00000136943 | 531.14 | 1.59 | 0.00 | ENSG00000233041 | 8671.73 | -1.56 | 0.00 |
| ENSG00000104312 | 956.47 | 1.59 | 0.00 | ENSG00000110079 | 271.67 | -1.56 | 0.00 |
| ENSG00000203896 | 144.84 | 1.59 | 0.00 | ENSG00000082497 | 172.92 | -1.56 | 0.00 |
| ENSG00000170312 | 2177.84 | 1.59 | 0.00 | ENSG00000181826 | 156.42 | -1.56 | 0.00 |
| ENSG00000111199 | 88.92 | 1.59 | 0.00 | ENSG00000105205 | 72.21 | -1.56 | 0.00 |
| ENSG00000150556 | 155.91 | 1.59 | 0.00 | ENSG00000135426 | 54.06 | -1.56 | 0.00 |
| ENSG00000126249 | 390.53 | 1.59 | 0.00 | ENSG00000171522 | 988.37 | -1.57 | 0.00 |
| ENSG00000197961 | 1406.85 | 1.59 | 0.00 | ENSG00000107796 | 7387.34 | -1.57 | 0.00 |
| ENSG00000114854 | 94.54 | 1.59 | 0.00 | ENSG00000104154 | 303.87 | -1.57 | 0.00 |
| ENSG00000161996 | 1769.17 | 1.58 | 0.00 | ENSG00000176894 | 597.84 | -1.57 | 0.00 |
| ENSG00000249992 | 447.90 | 1.58 | 0.00 | ENSG00000178537 | 836.58 | -1.57 | 0.00 |
| ENSG00000177192 | 1687.27 | 1.58 | 0.00 | ENSG00000155659 | 476.51 | -1.57 | 0.00 |
| ENSG00000173531 | 208.17 | 1.58 | 0.00 | ENSG00000162407 | 1227.70 | -1.57 | 0.00 |
| ENSG00000161091 | 2352.12 | 1.57 | 0.00 | ENSG00000007237 | 712.55 | -1.57 | 0.00 |
| ENSG00000213553 | 670.87 | 1.57 | 0.00 | ENSG00000166126 | 2253.20 | -1.57 | 0.00 |
| ENSG00000089351 | 2953.60 | 1.57 | 0.00 | ENSG00000134755 | 4240.24 | -1.57 | 0.00 |
| ENSG00000173535 | 96.71 | 1.57 | 0.00 | ENSG00000054356 | 70.45 | -1.57 | 0.00 |
| ENSG00000167513 | 1924.77 | 1.57 | 0.00 | ENSG00000111728 | 61.03 | -1.58 | 0.00 |
| ENSG00000167393 | 590.22 | 1.57 | 0.00 | ENSG00000176438 | 238.06 | -1.58 | 0.00 |
| ENSG00000128059 | 880.93 | 1.57 | 0.00 | ENSG00000265972 | 16722.59 | -1.58 | 0.00 |
| ENSG00000172244 | 139.06 | 1.57 | 0.00 | ENSG00000138685 | 145.72 | -1.58 | 0.00 |
| ENSG00000114631 | 2126.19 | 1.57 | 0.00 | ENSG00000026559 | 84.28 | -1.58 | 0.00 |
| ENSG00000281344 | 50.05 | 1.57 | 0.00 | ENSG00000137628 | 1389.87 | -1.58 | 0.00 |
| ENSG00000171163 | 832.78 | 1.57 | 0.00 | ENSG00000143816 | 96.31 | -1.58 | 0.00 |
| ENSG00000164692 | 55915.68 | 1.56 | 0.00 | ENSG00000203710 | 77.95 | -1.58 | 0.00 |
| ENSG00000149948 | 620.45 | 1.56 | 0.00 | ENSG00000114248 | 491.18 | -1.58 | 0.00 |
| ENSG00000187840 | 2283.16 | 1.56 | 0.00 | ENSG00000076662 | 65.59 | -1.59 | 0.00 |
| ENSG00000188910 | 1097.85 | 1.56 | 0.00 | ENSG00000183963 | 4516.89 | -1.59 | 0.00 |
| ENSG00000102384 | 286.52 | 1.56 | 0.00 | ENSG00000106772 | 1881.85 | -1.59 | 0.00 |
| ENSG00000254815 | 61.79 | 1.56 | 0.00 | ENSG00000154917 | 170.92 | -1.59 | 0.00 |
| ENSG00000141582 | 1426.18 | 1.55 | 0.00 | ENSG00000185561 | 694.01 | -1.59 | 0.00 |
| ENSG00000077152 | 922.86 | 1.55 | 0.00 | ENSG00000074370 | 5357.77 | -1.59 | 0.00 |
| ENSG00000124207 | 6117.43 | 1.55 | 0.00 | ENSG00000211955 | 572.85 | -1.59 | 0.00 |
| ENSG00000130635 | 8839.76 | 1.55 | 0.00 | ENSG00000166313 | 308.27 | -1.60 | 0.00 |
| ENSG00000174371 | 488.43 | 1.55 | 0.00 | ENSG00000128596 | 82.88 | -1.60 | 0.00 |
| ENSG00000167771 | 213.16 | 1.55 | 0.00 | ENSG00000068831 | 137.06 | -1.60 | 0.00 |
| ENSG00000125798 | 1549.05 | 1.55 | 0.00 | ENSG00000105755 | 5738.80 | -1.60 | 0.00 |
| ENSG00000134057 | 3085.62 | 1.55 | 0.00 | ENSG00000083814 | 60.50 | -1.60 | 0.00 |
| ENSG00000116883 | 120.85 | 1.54 | 0.00 | ENSG00000154678 | 64.42 | -1.60 | 0.00 |
| ENSG00000123975 | 2036.85 | 1.54 | 0.00 | ENSG00000168405 | 183.22 | -1.61 | 0.00 |
| ENSG00000065600 | 385.37 | 1.54 | 0.00 | ENSG00000126803 | 1045.47 | -1.61 | 0.00 |
| ENSG00000108773 | 3121.79 | 1.54 | 0.00 | ENSG00000171115 | 252.22 | -1.61 | 0.00 |
| ENSG00000047634 | 1027.35 | 1.53 | 0.00 | ENSG00000214357 | 2129.98 | -1.61 | 0.00 |
| ENSG00000087586 | 1766.83 | 1.53 | 0.00 | ENSG00000139567 | 2508.79 | -1.61 | 0.00 |
| ENSG00000167702 | 855.54 | 1.53 | 0.00 | ENSG00000177191 | 918.08 | -1.61 | 0.00 |
| ENSG00000142185 | 1065.03 | 1.53 | 0.00 | ENSG00000140416 | 12993.67 | -1.61 | 0.00 |
| ENSG00000160193 | 824.93 | 1.53 | 0.00 | ENSG00000183023 | 363.92 | -1.61 | 0.00 |
| ENSG00000112578 | 1161.95 | 1.53 | 0.00 | ENSG00000142583 | 242.27 | -1.61 | 0.00 |
| ENSG00000164109 | 1720.06 | 1.53 | 0.00 | ENSG00000171659 | 148.89 | -1.61 | 0.00 |
| ENSG00000140534 | 477.78 | 1.53 | 0.00 | ENSG00000139433 | 1497.16 | -1.61 | 0.00 |
| ENSG00000171858 | 32333.30 | 1.52 | 0.00 | ENSG00000124942 | 18806.96 | -1.61 | 0.00 |
| ENSG00000111665 | 886.87 | 1.52 | 0.00 | ENSG00000062524 | 275.61 | -1.61 | 0.00 |
| ENSG00000136295 | 6673.98 | 1.52 | 0.00 | ENSG00000081189 | 431.29 | -1.62 | 0.00 |
| ENSG00000088882 | 517.65 | 1.52 | 0.00 | ENSG00000204361 | 111.41 | -1.62 | 0.00 |
| ENSG00000226287 | 175.55 | 1.52 | 0.00 | ENSG00000131016 | 1124.21 | -1.62 | 0.00 |
| ENSG00000112984 | 913.55 | 1.52 | 0.00 | ENSG00000160111 | 52.11 | -1.62 | 0.00 |
| ENSG00000146670 | 1613.16 | 1.52 | 0.00 | ENSG00000161714 | 4149.94 | -1.62 | 0.00 |
| ENSG00000146197 | 156.06 | 1.52 | 0.00 | ENSG00000075142 | 6899.01 | -1.62 | 0.00 |
| ENSG00000140937 | 1762.54 | 1.52 | 0.00 | ENSG00000180543 | 140.64 | -1.62 | 0.00 |
| ENSG00000089685 | 2491.63 | 1.52 | 0.00 | ENSG00000219481 | 464.16 | -1.62 | 0.00 |
| ENSG00000253368 | 348.05 | 1.51 | 0.00 | ENSG00000134321 | 436.71 | -1.62 | 0.00 |
| ENSG00000175711 | 263.29 | 1.51 | 0.00 | ENSG00000211946 | 60.06 | -1.62 | 0.00 |
| ENSG00000167711 | 235.31 | 1.51 | 0.00 | ENSG00000253313 | 936.04 | -1.62 | 0.00 |
| ENSG00000249007 | 156.39 | 1.51 | 0.00 | ENSG00000211956 | 537.30 | -1.62 | 0.00 |
| ENSG00000130475 | 614.01 | 1.51 | 0.00 | ENSG00000151632 | 138.81 | -1.62 | 0.00 |
| ENSG00000235280 | 171.29 | 1.51 | 0.00 | ENSG00000171791 | 373.87 | -1.62 | 0.00 |
| ENSG00000136883 | 1179.81 | 1.51 | 0.00 | ENSG00000271824 | 182.19 | -1.63 | 0.00 |
| ENSG00000218014 | 50.68 | 1.51 | 0.00 | ENSG00000110900 | 375.37 | -1.63 | 0.00 |
| ENSG00000121716 | 249.14 | 1.51 | 0.00 | ENSG00000270550 | 796.38 | -1.63 | 0.00 |
| ENSG00000198088 | 169.65 | 1.51 | 0.00 | ENSG00000197928 | 53.22 | -1.63 | 0.00 |
| ENSG00000185838 | 382.58 | 1.51 | 0.00 | ENSG00000133710 | 184.48 | -1.63 | 0.00 |
| ENSG00000129667 | 1735.49 | 1.51 | 0.00 | ENSG00000069974 | 1002.61 | -1.64 | 0.00 |
| ENSG00000234062 | 77.10 | 1.51 | 0.00 | ENSG00000150764 | 542.57 | -1.64 | 0.00 |
| ENSG00000169607 | 539.67 | 1.51 | 0.00 | ENSG00000173068 | 199.90 | -1.64 | 0.00 |
| ENSG00000104889 | 1592.30 | 1.50 | 0.00 | ENSG00000152078 | 1019.16 | -1.64 | 0.00 |
| ENSG00000136108 | 1880.01 | 1.50 | 0.00 | ENSG00000144820 | 351.00 | -1.64 | 0.00 |
| ENSG00000226332 | 61.09 | 1.50 | 0.00 | ENSG00000100739 | 68.71 | -1.64 | 0.00 |
| ENSG00000182199 | 6519.08 | 1.50 | 0.00 | ENSG00000160593 | 328.18 | -1.64 | 0.00 |
| ENSG00000204366 | 345.43 | 1.50 | 0.00 | ENSG00000149218 | 1994.24 | -1.65 | 0.00 |
| ENSG00000107815 | 1032.03 | 1.50 | 0.00 | ENSG00000211655 | 103.32 | -1.65 | 0.00 |
| ENSG00000158050 | 524.47 | 1.50 | 0.00 | ENSG00000227372 | 139.16 | -1.65 | 0.00 |
| ENSG00000173432 | 400.24 | 1.50 | 0.00 | ENSG00000207834 | 72.46 | -1.65 | 0.00 |
| ENSG00000143320 | 282.62 | 1.49 | 0.00 | ENSG00000156234 | 268.03 | -1.65 | 0.00 |
| ENSG00000261115 | 202.13 | 1.49 | 0.00 | ENSG00000145703 | 1797.45 | -1.65 | 0.00 |
| ENSG00000121211 | 221.84 | 1.49 | 0.00 | ENSG00000136052 | 740.31 | -1.65 | 0.00 |
| ENSG00000223803 | 64.22 | 1.49 | 0.00 | ENSG00000159958 | 88.16 | -1.65 | 0.00 |
| ENSG00000152056 | 319.45 | 1.49 | 0.00 | ENSG00000183111 | 481.82 | -1.65 | 0.00 |
| ENSG00000125319 | 254.56 | 1.49 | 0.00 | ENSG00000170962 | 207.03 | -1.66 | 0.00 |
| ENSG00000227939 | 91.51 | 1.49 | 0.00 | ENSG00000137558 | 129.35 | -1.66 | 0.00 |
| ENSG00000065911 | 3274.57 | 1.49 | 0.00 | ENSG00000077942 | 3851.14 | -1.66 | 0.00 |
| ENSG00000142089 | 17827.32 | 1.49 | 0.00 | ENSG00000123096 | 378.76 | -1.66 | 0.00 |
| ENSG00000260807 | 132.72 | 1.49 | 0.00 | ENSG00000153814 | 230.80 | -1.66 | 0.00 |
| ENSG00000130826 | 5137.24 | 1.49 | 0.00 | ENSG00000182162 | 164.31 | -1.66 | 0.00 |
| ENSG00000198108 | 63.59 | 1.49 | 0.00 | ENSG00000103710 | 258.02 | -1.66 | 0.00 |
| ENSG00000151490 | 2024.74 | 1.49 | 0.00 | ENSG00000153823 | 779.46 | -1.66 | 0.00 |
| ENSG00000171848 | 4039.41 | 1.48 | 0.00 | ENSG00000138600 | 2381.84 | -1.66 | 0.00 |
| ENSG00000085999 | 448.37 | 1.48 | 0.00 | ENSG00000144642 | 132.99 | -1.66 | 0.00 |
| ENSG00000115163 | 401.68 | 1.48 | 0.00 | ENSG00000106571 | 184.03 | -1.67 | 0.00 |
| ENSG00000137692 | 973.15 | 1.48 | 0.00 | ENSG00000072952 | 858.96 | -1.67 | 0.00 |
| ENSG00000233559 | 97.83 | 1.48 | 0.00 | ENSG00000081923 | 7011.74 | -1.67 | 0.00 |
| ENSG00000082512 | 1942.23 | 1.48 | 0.00 | ENSG00000259207 | 95.53 | -1.67 | 0.00 |
| ENSG00000100473 | 138.43 | 1.48 | 0.00 | ENSG00000049246 | 355.52 | -1.67 | 0.00 |
| ENSG00000101898 | 57.48 | 1.48 | 0.00 | ENSG00000259342 | 195.84 | -1.67 | 0.00 |
| ENSG00000187486 | 158.02 | 1.48 | 0.00 | ENSG00000136732 | 350.04 | -1.68 | 0.00 |
| ENSG00000164542 | 234.30 | 1.48 | 0.00 | ENSG00000144136 | 2457.03 | -1.68 | 0.00 |
| ENSG00000259479 | 119.81 | 1.48 | 0.00 | ENSG00000211640 | 773.28 | -1.68 | 0.00 |
| ENSG00000120694 | 6691.31 | 1.47 | 0.00 | ENSG00000147036 | 83.81 | -1.68 | 0.00 |
| ENSG00000237988 | 500.65 | 1.47 | 0.00 | ENSG00000162896 | 133087.51 | -1.68 | 0.00 |
| ENSG00000141738 | 1883.99 | 1.47 | 0.00 | ENSG00000167117 | 471.24 | -1.68 | 0.00 |
| ENSG00000114115 | 750.61 | 1.47 | 0.00 | ENSG00000161533 | 4084.68 | -1.68 | 0.00 |
| ENSG00000189007 | 560.79 | 1.47 | 0.00 | ENSG00000213203 | 130.90 | -1.69 | 0.00 |
| ENSG00000185666 | 99.21 | 1.47 | 0.00 | ENSG00000114698 | 581.99 | -1.69 | 0.00 |
| ENSG00000161647 | 130.36 | 1.47 | 0.00 | ENSG00000167800 | 146.98 | -1.69 | 0.00 |
| ENSG00000214796 | 276.10 | 1.47 | 0.00 | ENSG00000242732 | 91.03 | -1.69 | 0.00 |
| ENSG00000064651 | 18219.68 | 1.47 | 0.00 | ENSG00000172236 | 280.71 | -1.69 | 0.00 |
| ENSG00000166278 | 1921.33 | 1.46 | 0.00 | ENSG00000134061 | 127.60 | -1.69 | 0.00 |
| ENSG00000116132 | 641.75 | 1.46 | 0.00 | ENSG00000276855 | 56.51 | -1.69 | 0.00 |
| ENSG00000117650 | 927.67 | 1.46 | 0.00 | ENSG00000107738 | 1569.44 | -1.69 | 0.00 |
| ENSG00000116649 | 3187.90 | 1.46 | 0.00 | ENSG00000139970 | 113.76 | -1.69 | 0.00 |
| ENSG00000168005 | 877.23 | 1.46 | 0.00 | ENSG00000198515 | 126.01 | -1.69 | 0.00 |
| ENSG00000168209 | 3128.71 | 1.46 | 0.00 | ENSG00000158473 | 101.12 | -1.69 | 0.00 |
| ENSG00000213420 | 61.76 | 1.46 | 0.00 | ENSG00000129595 | 103.06 | -1.70 | 0.00 |
| ENSG00000146410 | 293.10 | 1.46 | 0.00 | ENSG00000140450 | 1102.88 | -1.70 | 0.00 |
| ENSG00000272168 | 108.84 | 1.46 | 0.00 | ENSG00000163637 | 225.41 | -1.70 | 0.00 |
| ENSG00000130054 | 109.98 | 1.46 | 0.00 | ENSG00000135083 | 394.08 | -1.70 | 0.00 |
| ENSG00000162063 | 1191.57 | 1.46 | 0.00 | ENSG00000159184 | 978.27 | -1.70 | 0.00 |
| ENSG00000104341 | 6210.35 | 1.46 | 0.00 | ENSG00000133808 | 120.50 | -1.70 | 0.00 |
| ENSG00000174137 | 63.57 | 1.46 | 0.00 | ENSG00000253304 | 96.70 | -1.70 | 0.00 |
| ENSG00000184220 | 941.00 | 1.46 | 0.00 | ENSG00000114812 | 1474.63 | -1.70 | 0.00 |
| ENSG00000142945 | 1311.91 | 1.46 | 0.00 | ENSG00000154274 | 692.23 | -1.70 | 0.00 |
| ENSG00000090382 | 20225.60 | 1.46 | 0.00 | ENSG00000119950 | 1633.90 | -1.70 | 0.00 |
| ENSG00000213903 | 270.05 | 1.46 | 0.00 | ENSG00000169083 | 76.25 | -1.71 | 0.00 |
| ENSG00000205664 | 279.06 | 1.46 | 0.00 | ENSG00000116741 | 823.49 | -1.71 | 0.00 |
| ENSG00000172216 | 2101.55 | 1.46 | 0.00 | ENSG00000148516 | 624.33 | -1.71 | 0.00 |
| ENSG00000185201 | 4420.83 | 1.46 | 0.00 | ENSG00000188641 | 371.42 | -1.71 | 0.00 |
| ENSG00000221944 | 173.09 | 1.46 | 0.00 | ENSG00000169918 | 75.09 | -1.71 | 0.00 |
| ENSG00000133131 | 1306.87 | 1.45 | 0.00 | ENSG00000150510 | 75.47 | -1.71 | 0.00 |
| ENSG00000166851 | 2471.90 | 1.45 | 0.00 | ENSG00000185339 | 919.21 | -1.71 | 0.00 |
| ENSG00000146733 | 912.00 | 1.45 | 0.00 | ENSG00000134769 | 169.29 | -1.72 | 0.00 |
| ENSG00000114767 | 1670.43 | 1.45 | 0.00 | ENSG00000101445 | 316.92 | -1.72 | 0.00 |
| ENSG00000242125 | 1341.34 | 1.45 | 0.00 | ENSG00000224259 | 536.14 | -1.72 | 0.00 |
| ENSG00000171224 | 277.66 | 1.45 | 0.00 | ENSG00000091986 | 1913.71 | -1.72 | 0.00 |
| ENSG00000197859 | 338.39 | 1.45 | 0.00 | ENSG00000006555 | 1284.81 | -1.72 | 0.00 |
| ENSG00000180071 | 72.80 | 1.45 | 0.00 | ENSG00000166963 | 512.65 | -1.72 | 0.00 |
| ENSG00000119969 | 892.86 | 1.45 | 0.00 | ENSG00000211899 | 10391.77 | -1.72 | 0.00 |
| ENSG00000165304 | 1064.29 | 1.45 | 0.00 | ENSG00000167315 | 4773.19 | -1.72 | 0.00 |
| ENSG00000070669 | 2112.70 | 1.45 | 0.00 | ENSG00000158315 | 367.85 | -1.72 | 0.00 |
| ENSG00000130590 | 556.69 | 1.45 | 0.00 | ENSG00000198771 | 317.74 | -1.72 | 0.00 |
| ENSG00000100036 | 733.65 | 1.45 | 0.00 | ENSG00000158158 | 3210.38 | -1.72 | 0.00 |
| ENSG00000273619 | 72.15 | 1.45 | 0.00 | ENSG00000155324 | 800.44 | -1.73 | 0.00 |
| ENSG00000188343 | 606.64 | 1.45 | 0.00 | ENSG00000085265 | 125.19 | -1.73 | 0.00 |
| ENSG00000090447 | 1027.50 | 1.45 | 0.00 | ENSG00000153898 | 399.00 | -1.73 | 0.00 |
| ENSG00000249464 | 63.22 | 1.44 | 0.00 | ENSG00000114166 | 456.46 | -1.73 | 0.00 |
| ENSG00000112541 | 146.57 | 1.44 | 0.00 | ENSG00000171992 | 2623.16 | -1.74 | 0.00 |
| ENSG00000112715 | 5920.63 | 1.44 | 0.00 | ENSG00000102003 | 132.10 | -1.74 | 0.00 |
| ENSG00000243547 | 50.54 | 1.44 | 0.00 | ENSG00000162949 | 331.84 | -1.74 | 0.00 |
| ENSG00000169710 | 12795.21 | 1.44 | 0.00 | ENSG00000173267 | 98.47 | -1.74 | 0.00 |
| ENSG00000112837 | 108.73 | 1.44 | 0.00 | ENSG00000164237 | 3205.10 | -1.74 | 0.00 |
| ENSG00000132661 | 1031.18 | 1.44 | 0.00 | ENSG00000100292 | 1140.31 | -1.74 | 0.00 |
| ENSG00000204899 | 1225.74 | 1.44 | 0.00 | ENSG00000060140 | 410.63 | -1.74 | 0.00 |
| ENSG00000165568 | 95.35 | 1.44 | 0.00 | ENSG00000179750 | 333.99 | -1.74 | 0.00 |
| ENSG00000171241 | 504.92 | 1.44 | 0.00 | ENSG00000153303 | 417.00 | -1.74 | 0.00 |
| ENSG00000167476 | 181.05 | 1.44 | 0.00 | ENSG00000243244 | 199.55 | -1.74 | 0.00 |
| ENSG00000120526 | 1200.39 | 1.44 | 0.00 | ENSG00000132561 | 1636.53 | -1.75 | 0.00 |
| ENSG00000139438 | 399.19 | 1.44 | 0.00 | ENSG00000011465 | 7427.36 | -1.75 | 0.00 |
| ENSG00000118557 | 127.45 | 1.44 | 0.00 | ENSG00000048052 | 184.38 | -1.75 | 0.00 |
| ENSG00000165724 | 994.43 | 1.44 | 0.00 | ENSG00000169418 | 171.06 | -1.75 | 0.00 |
| ENSG00000123610 | 148.63 | 1.44 | 0.00 | ENSG00000165806 | 2743.55 | -1.75 | 0.00 |
| ENSG00000169679 | 1374.00 | 1.44 | 0.00 | ENSG00000117091 | 339.80 | -1.75 | 0.00 |
| ENSG00000204764 | 181.63 | 1.44 | 0.00 | ENSG00000127990 | 246.97 | -1.75 | 0.00 |
| ENSG00000170779 | 971.79 | 1.44 | 0.00 | ENSG00000173198 | 59.70 | -1.75 | 0.00 |
| ENSG00000230629 | 112.07 | 1.43 | 0.00 | ENSG00000148842 | 644.03 | -1.75 | 0.00 |
| ENSG00000172965 | 510.64 | 1.43 | 0.00 | ENSG00000211951 | 174.87 | -1.75 | 0.00 |
| ENSG00000173110 | 167.67 | 1.43 | 0.00 | ENSG00000090530 | 502.80 | -1.75 | 0.00 |
| ENSG00000133740 | 626.15 | 1.43 | 0.00 | ENSG00000149090 | 196.39 | -1.75 | 0.00 |
| ENSG00000104356 | 545.17 | 1.43 | 0.00 | ENSG00000101049 | 984.47 | -1.75 | 0.00 |
| ENSG00000101361 | 5629.40 | 1.43 | 0.00 | ENSG00000157404 | 335.03 | -1.76 | 0.00 |
| ENSG00000217801 | 72.48 | 1.42 | 0.00 | ENSG00000156006 | 217.09 | -1.76 | 0.00 |
| ENSG00000258839 | 130.31 | 1.42 | 0.00 | ENSG00000211668 | 1359.19 | -1.76 | 0.00 |
| ENSG00000178821 | 215.84 | 1.42 | 0.00 | ENSG00000196263 | 54.61 | -1.76 | 0.00 |
| ENSG00000117724 | 2348.53 | 1.42 | 0.00 | ENSG00000179144 | 196.96 | -1.76 | 0.00 |
| ENSG00000176826 | 101.59 | 1.42 | 0.00 | ENSG00000164442 | 1079.70 | -1.77 | 0.00 |
| ENSG00000131747 | 5685.32 | 1.42 | 0.00 | ENSG00000243466 | 3048.36 | -1.77 | 0.00 |
| ENSG00000254290 | 728.62 | 1.42 | 0.00 | ENSG00000125648 | 2877.39 | -1.77 | 0.00 |
| ENSG00000167695 | 1389.20 | 1.42 | 0.00 | ENSG00000054277 | 277.10 | -1.77 | 0.00 |
| ENSG00000112280 | 158.31 | 1.42 | 0.00 | ENSG00000130558 | 195.60 | -1.77 | 0.00 |
| ENSG00000186603 | 703.67 | 1.42 | 0.00 | ENSG00000138079 | 552.36 | -1.77 | 0.00 |
| ENSG00000140263 | 1880.16 | 1.42 | 0.00 | ENSG00000231475 | 342.58 | -1.77 | 0.00 |
| ENSG00000101311 | 9763.52 | 1.42 | 0.00 | ENSG00000088827 | 330.85 | -1.77 | 0.00 |
| ENSG00000113140 | 30789.05 | 1.42 | 0.00 | ENSG00000151229 | 535.52 | -1.77 | 0.00 |
| ENSG00000137309 | 17624.05 | 1.41 | 0.00 | ENSG00000170271 | 579.58 | -1.78 | 0.00 |
| ENSG00000178896 | 1345.06 | 1.41 | 0.00 | ENSG00000211964 | 270.78 | -1.78 | 0.00 |
| ENSG00000106133 | 58.11 | 1.41 | 0.00 | ENSG00000113580 | 561.38 | -1.78 | 0.00 |
| ENSG00000262902 | 153.04 | 1.41 | 0.00 | ENSG00000073712 | 839.47 | -1.78 | 0.00 |
| ENSG00000130731 | 2401.75 | 1.41 | 0.00 | ENSG00000170075 | 91.65 | -1.78 | 0.00 |
| ENSG00000236255 | 66.42 | 1.41 | 0.00 | ENSG00000088256 | 5273.06 | -1.78 | 0.00 |
| ENSG00000077348 | 1378.68 | 1.41 | 0.00 | ENSG00000092096 | 202.63 | -1.78 | 0.00 |
| ENSG00000172893 | 3853.43 | 1.41 | 0.00 | ENSG00000167332 | 54.99 | -1.78 | 0.00 |
| ENSG00000187741 | 913.35 | 1.41 | 0.00 | ENSG00000211962 | 786.81 | -1.78 | 0.00 |
| ENSG00000243554 | 125.04 | 1.41 | 0.00 | ENSG00000165124 | 417.23 | -1.78 | 0.00 |
| ENSG00000115828 | 1235.95 | 1.41 | 0.00 | ENSG00000186594 | 455.69 | -1.79 | 0.00 |
| ENSG00000232346 | 60.60 | 1.41 | 0.00 | ENSG00000172349 | 332.38 | -1.79 | 0.00 |
| ENSG00000052749 | 2150.77 | 1.41 | 0.00 | ENSG00000197208 | 105.76 | -1.79 | 0.00 |
| ENSG00000143494 | 92.89 | 1.41 | 0.00 | ENSG00000059728 | 2604.97 | -1.79 | 0.00 |
| ENSG00000084774 | 3609.60 | 1.41 | 0.00 | ENSG00000167741 | 2849.04 | -1.79 | 0.00 |
| ENSG00000104290 | 547.58 | 1.40 | 0.00 | ENSG00000196092 | 128.67 | -1.79 | 0.00 |
| ENSG00000111641 | 2345.58 | 1.40 | 0.00 | ENSG00000211653 | 1556.11 | -1.79 | 0.00 |
| ENSG00000145194 | 751.34 | 1.40 | 0.00 | ENSG00000134531 | 4951.05 | -1.80 | 0.00 |
| ENSG00000127863 | 219.02 | 1.40 | 0.00 | ENSG00000126759 | 77.34 | -1.80 | 0.00 |
| ENSG00000124116 | 79.83 | 1.40 | 0.00 | ENSG00000249096 | 50.66 | -1.80 | 0.00 |
| ENSG00000101158 | 5141.67 | 1.40 | 0.00 | ENSG00000026751 | 609.93 | -1.80 | 0.00 |
| ENSG00000037897 | 806.79 | 1.40 | 0.00 | ENSG00000163683 | 2686.92 | -1.80 | 0.00 |
| ENSG00000198208 | 368.92 | 1.40 | 0.00 | ENSG00000232216 | 114.37 | -1.80 | 0.00 |
| ENSG00000274922 | 61.75 | 1.40 | 0.00 | ENSG00000111341 | 2301.61 | -1.80 | 0.00 |
| ENSG00000204618 | 182.71 | 1.40 | 0.00 | ENSG00000150594 | 605.97 | -1.80 | 0.00 |
| ENSG00000143786 | 112.49 | 1.40 | 0.00 | ENSG00000133687 | 267.59 | -1.80 | 0.00 |
| ENSG00000049768 | 134.70 | 1.40 | 0.00 | ENSG00000074755 | 2697.53 | -1.80 | 0.00 |
| ENSG00000145220 | 1052.04 | 1.39 | 0.00 | ENSG00000100490 | 68.25 | -1.81 | 0.00 |
| ENSG00000153551 | 714.40 | 1.39 | 0.00 | ENSG00000148541 | 52.49 | -1.81 | 0.00 |
| ENSG00000119630 | 369.67 | 1.39 | 0.00 | ENSG00000166311 | 1172.51 | -1.81 | 0.00 |
| ENSG00000138207 | 560.13 | 1.39 | 0.00 | ENSG00000181061 | 2311.62 | -1.81 | 0.00 |
| ENSG00000181026 | 1652.38 | 1.39 | 0.00 | ENSG00000182636 | 356.98 | -1.81 | 0.00 |
| ENSG00000149485 | 887.91 | 1.39 | 0.00 | ENSG00000105851 | 124.71 | -1.81 | 0.00 |
| ENSG00000260428 | 95.85 | 1.39 | 0.00 | ENSG00000148180 | 14126.30 | -1.82 | 0.00 |
| ENSG00000161179 | 1504.10 | 1.39 | 0.00 | ENSG00000085563 | 1688.25 | -1.82 | 0.00 |
| ENSG00000167280 | 3005.15 | 1.38 | 0.00 | ENSG00000119227 | 1287.04 | -1.82 | 0.00 |
| ENSG00000221947 | 99.93 | 1.38 | 0.00 | ENSG00000138131 | 76.97 | -1.82 | 0.00 |
| ENSG00000135919 | 3062.18 | 1.38 | 0.00 | ENSG00000099864 | 287.47 | -1.82 | 0.00 |
| ENSG00000186416 | 603.02 | 1.38 | 0.00 | ENSG00000211648 | 755.43 | -1.82 | 0.00 |
| ENSG00000181449 | 103.33 | 1.38 | 0.01 | ENSG00000158270 | 264.55 | -1.82 | 0.00 |
| ENSG00000186792 | 187.16 | 1.38 | 0.00 | ENSG00000076641 | 1098.22 | -1.82 | 0.00 |
| ENSG00000205517 | 227.48 | 1.38 | 0.00 | ENSG00000151150 | 894.18 | -1.82 | 0.00 |
| ENSG00000239415 | 74.29 | 1.38 | 0.00 | ENSG00000185274 | 59.48 | -1.82 | 0.00 |
| ENSG00000132003 | 661.93 | 1.38 | 0.00 | ENSG00000085552 | 933.36 | -1.82 | 0.00 |
| ENSG00000153044 | 538.92 | 1.37 | 0.00 | ENSG00000153790 | 196.42 | -1.82 | 0.00 |
| ENSG00000174501 | 80.62 | 1.37 | 0.00 | ENSG00000146267 | 65.50 | -1.82 | 0.00 |
| ENSG00000108479 | 1110.08 | 1.37 | 0.00 | ENSG00000211663 | 1451.06 | -1.82 | 0.00 |
| ENSG00000104907 | 1751.70 | 1.37 | 0.00 | ENSG00000100307 | 730.83 | -1.83 | 0.00 |
| ENSG00000165621 | 208.36 | 1.37 | 0.00 | ENSG00000179163 | 3415.55 | -1.83 | 0.00 |
| ENSG00000267523 | 66.15 | 1.37 | 0.00 | ENSG00000259120 | 122.18 | -1.83 | 0.00 |
| ENSG00000183444 | 166.11 | 1.37 | 0.00 | ENSG00000138735 | 846.30 | -1.83 | 0.00 |
| ENSG00000091129 | 190.44 | 1.37 | 0.00 | ENSG00000137860 | 407.39 | -1.83 | 0.00 |
| ENSG00000215252 | 279.50 | 1.37 | 0.00 | ENSG00000230006 | 72.48 | -1.83 | 0.00 |
| ENSG00000175792 | 2467.85 | 1.37 | 0.00 | ENSG00000154556 | 1010.01 | -1.83 | 0.00 |
| ENSG00000137807 | 949.98 | 1.37 | 0.00 | ENSG00000145335 | 62.66 | -1.83 | 0.00 |
| ENSG00000146233 | 361.21 | 1.37 | 0.00 | ENSG00000184730 | 2059.94 | -1.83 | 0.00 |
| ENSG00000088356 | 1299.16 | 1.37 | 0.00 | ENSG00000109819 | 516.64 | -1.83 | 0.00 |
| ENSG00000138346 | 406.36 | 1.37 | 0.00 | ENSG00000198932 | 146.70 | -1.83 | 0.00 |
| ENSG00000164611 | 1465.89 | 1.37 | 0.00 | ENSG00000211974 | 68.76 | -1.83 | 0.00 |
| ENSG00000179041 | 1616.31 | 1.37 | 0.00 | ENSG00000088854 | 206.36 | -1.84 | 0.00 |
| ENSG00000231607 | 155.08 | 1.37 | 0.00 | ENSG00000167779 | 433.99 | -1.84 | 0.00 |
| ENSG00000116455 | 1935.04 | 1.37 | 0.00 | ENSG00000144821 | 54.10 | -1.84 | 0.00 |
| ENSG00000141293 | 436.25 | 1.37 | 0.00 | ENSG00000169291 | 128.21 | -1.84 | 0.00 |
| ENSG00000125538 | 819.68 | 1.37 | 0.00 | ENSG00000064300 | 123.88 | -1.84 | 0.00 |
| ENSG00000273142 | 248.81 | 1.37 | 0.00 | ENSG00000109814 | 3017.15 | -1.84 | 0.00 |
| ENSG00000142632 | 675.70 | 1.37 | 0.00 | ENSG00000169282 | 59.69 | -1.84 | 0.00 |
| ENSG00000258429 | 169.49 | 1.36 | 0.00 | ENSG00000153233 | 465.04 | -1.84 | 0.00 |
| ENSG00000100985 | 1853.33 | 1.36 | 0.00 | ENSG00000197256 | 2289.03 | -1.84 | 0.00 |
| ENSG00000160949 | 1411.15 | 1.36 | 0.00 | ENSG00000178573 | 757.07 | -1.84 | 0.00 |
| ENSG00000167207 | 229.74 | 1.36 | 0.00 | ENSG00000134138 | 207.69 | -1.84 | 0.00 |
| ENSG00000280123 | 76.47 | 1.36 | 0.00 | ENSG00000005249 | 290.49 | -1.85 | 0.00 |
| ENSG00000168785 | 859.78 | 1.36 | 0.00 | ENSG00000043039 | 296.56 | -1.85 | 0.00 |
| ENSG00000179862 | 319.96 | 1.36 | 0.00 | ENSG00000163751 | 324.14 | -1.85 | 0.00 |
| ENSG00000038427 | 3633.21 | 1.36 | 0.00 | ENSG00000139193 | 139.96 | -1.85 | 0.00 |
| ENSG00000188818 | 82.77 | 1.36 | 0.00 | ENSG00000196664 | 79.54 | -1.85 | 0.00 |
| ENSG00000164171 | 3152.28 | 1.36 | 0.00 | ENSG00000143167 | 10470.58 | -1.85 | 0.00 |
| ENSG00000142530 | 110.64 | 1.36 | 0.00 | ENSG00000167074 | 710.63 | -1.85 | 0.00 |
| ENSG00000101333 | 4821.27 | 1.36 | 0.00 | ENSG00000163412 | 873.93 | -1.85 | 0.00 |
| ENSG00000051341 | 413.78 | 1.36 | 0.00 | ENSG00000124615 | 361.93 | -1.85 | 0.00 |
| ENSG00000204950 | 98.89 | 1.35 | 0.00 | ENSG00000184113 | 364.71 | -1.86 | 0.00 |
| ENSG00000228594 | 365.02 | 1.35 | 0.00 | ENSG00000263429 | 882.63 | -1.86 | 0.00 |
| ENSG00000134222 | 477.67 | 1.35 | 0.00 | ENSG00000175497 | 78.26 | -1.86 | 0.00 |
| ENSG00000091879 | 265.09 | 1.35 | 0.00 | ENSG00000100433 | 70.52 | -1.86 | 0.00 |
| ENSG00000151746 | 383.49 | 1.35 | 0.00 | ENSG00000134198 | 352.43 | -1.86 | 0.00 |
| ENSG00000100033 | 147.56 | 1.35 | 0.00 | ENSG00000126947 | 153.03 | -1.86 | 0.00 |
| ENSG00000075218 | 735.37 | 1.35 | 0.00 | ENSG00000076555 | 784.65 | -1.86 | 0.00 |
| ENSG00000101190 | 697.89 | 1.35 | 0.00 | ENSG00000166025 | 616.38 | -1.86 | 0.00 |
| ENSG00000111206 | 2226.79 | 1.35 | 0.00 | ENSG00000211952 | 155.51 | -1.87 | 0.00 |
| ENSG00000132330 | 180.23 | 1.35 | 0.00 | ENSG00000076258 | 132.61 | -1.87 | 0.00 |
| ENSG00000132182 | 3866.69 | 1.35 | 0.00 | ENSG00000197943 | 430.69 | -1.87 | 0.00 |
| ENSG00000245970 | 145.51 | 1.35 | 0.00 | ENSG00000164236 | 62.29 | -1.87 | 0.00 |
| ENSG00000121621 | 330.46 | 1.35 | 0.00 | ENSG00000166770 | 58.48 | -1.87 | 0.00 |
| ENSG00000184575 | 3860.75 | 1.35 | 0.00 | ENSG00000119138 | 942.11 | -1.87 | 0.00 |
| ENSG00000090889 | 1201.98 | 1.35 | 0.00 | ENSG00000243264 | 162.85 | -1.87 | 0.00 |
| ENSG00000253716 | 180.62 | 1.35 | 0.00 | ENSG00000122786 | 7617.68 | -1.87 | 0.00 |
| ENSG00000240972 | 2156.31 | 1.34 | 0.00 | ENSG00000002726 | 9451.42 | -1.87 | 0.00 |
| ENSG00000180785 | 133.80 | 1.34 | 0.00 | ENSG00000215193 | 2753.98 | -1.87 | 0.00 |
| ENSG00000138411 | 193.16 | 1.34 | 0.00 | ENSG00000198488 | 682.91 | -1.88 | 0.00 |
| ENSG00000236017 | 102.23 | 1.34 | 0.00 | ENSG00000196557 | 811.66 | -1.88 | 0.00 |
| ENSG00000203876 | 80.32 | 1.34 | 0.00 | ENSG00000211945 | 1325.54 | -1.88 | 0.00 |
| ENSG00000140525 | 1745.54 | 1.34 | 0.00 | ENSG00000177363 | 100.21 | -1.89 | 0.00 |
| ENSG00000114251 | 818.97 | 1.34 | 0.00 | ENSG00000253998 | 54.02 | -1.89 | 0.00 |
| ENSG00000156697 | 1526.47 | 1.34 | 0.00 | ENSG00000185437 | 79.98 | -1.89 | 0.00 |
| ENSG00000066279 | 931.74 | 1.34 | 0.00 | ENSG00000115590 | 495.83 | -1.89 | 0.00 |
| ENSG00000083635 | 504.26 | 1.34 | 0.00 | ENSG00000145147 | 221.04 | -1.89 | 0.00 |
| ENSG00000245910 | 2454.33 | 1.34 | 0.00 | ENSG00000111802 | 3869.11 | -1.89 | 0.00 |
| ENSG00000225968 | 88.73 | 1.34 | 0.00 | ENSG00000143995 | 310.02 | -1.90 | 0.00 |
| ENSG00000159259 | 588.16 | 1.34 | 0.00 | ENSG00000168229 | 139.42 | -1.90 | 0.00 |
| ENSG00000189366 | 85.03 | 1.34 | 0.00 | ENSG00000102409 | 296.08 | -1.90 | 0.00 |
| ENSG00000262468 | 112.55 | 1.34 | 0.00 | ENSG00000110777 | 415.67 | -1.90 | 0.00 |
| ENSG00000102030 | 1987.17 | 1.34 | 0.00 | ENSG00000125845 | 721.15 | -1.91 | 0.00 |
| ENSG00000261061 | 176.86 | 1.34 | 0.00 | ENSG00000166402 | 71.97 | -1.91 | 0.00 |
| ENSG00000026508 | 11792.73 | 1.34 | 0.00 | ENSG00000171747 | 49820.10 | -1.91 | 0.00 |
| ENSG00000163577 | 351.24 | 1.34 | 0.00 | ENSG00000241224 | 132.20 | -1.91 | 0.00 |
| ENSG00000196497 | 246.46 | 1.34 | 0.00 | ENSG00000171503 | 778.47 | -1.91 | 0.00 |
| ENSG00000101457 | 2591.19 | 1.34 | 0.00 | ENSG00000240671 | 100.64 | -1.91 | 0.00 |
| ENSG00000067177 | 629.39 | 1.33 | 0.00 | ENSG00000187239 | 1327.74 | -1.92 | 0.00 |
| ENSG00000102221 | 769.25 | 1.33 | 0.00 | ENSG00000211660 | 1753.94 | -1.92 | 0.00 |
| ENSG00000111490 | 568.22 | 1.33 | 0.00 | ENSG00000122694 | 800.19 | -1.92 | 0.00 |
| ENSG00000179588 | 583.99 | 1.33 | 0.00 | ENSG00000197253 | 279.54 | -1.92 | 0.00 |
| ENSG00000101146 | 2206.72 | 1.33 | 0.00 | ENSG00000188993 | 290.03 | -1.92 | 0.00 |
| ENSG00000160072 | 1100.01 | 1.33 | 0.00 | ENSG00000114790 | 206.42 | -1.92 | 0.00 |
| ENSG00000129195 | 431.41 | 1.33 | 0.00 | ENSG00000109705 | 53.70 | -1.92 | 0.00 |
| ENSG00000222041 | 291.62 | 1.33 | 0.00 | ENSG00000166250 | 459.64 | -1.93 | 0.00 |
| ENSG00000151136 | 109.63 | 1.33 | 0.00 | ENSG00000113296 | 419.30 | -1.93 | 0.00 |
| ENSG00000166451 | 637.62 | 1.33 | 0.00 | ENSG00000211685 | 151.98 | -1.93 | 0.00 |
| ENSG00000234449 | 57.92 | 1.33 | 0.00 | ENSG00000111796 | 100.55 | -1.93 | 0.00 |
| ENSG00000138778 | 603.50 | 1.33 | 0.00 | ENSG00000179277 | 54.00 | -1.94 | 0.00 |
| ENSG00000067955 | 2177.49 | 1.33 | 0.00 | ENSG00000106688 | 788.76 | -1.94 | 0.00 |
| ENSG00000169750 | 192.15 | 1.33 | 0.00 | ENSG00000145687 | 229.58 | -1.94 | 0.00 |
| ENSG00000205336 | 8759.48 | 1.33 | 0.00 | ENSG00000211679 | 3087.88 | -1.94 | 0.00 |
| ENSG00000159216 | 2571.66 | 1.33 | 0.00 | ENSG00000135424 | 589.82 | -1.94 | 0.00 |
| ENSG00000197119 | 981.57 | 1.33 | 0.00 | ENSG00000154734 | 908.85 | -1.94 | 0.00 |
| ENSG00000164946 | 694.74 | 1.33 | 0.00 | ENSG00000162733 | 933.37 | -1.94 | 0.00 |
| ENSG00000129654 | 255.24 | 1.33 | 0.00 | ENSG00000172348 | 318.01 | -1.94 | 0.00 |
| ENSG00000163040 | 143.22 | 1.33 | 0.00 | ENSG00000174562 | 50.96 | -1.94 | 0.00 |
| ENSG00000101470 | 514.69 | 1.32 | 0.00 | ENSG00000115295 | 157.04 | -1.95 | 0.00 |
| ENSG00000188760 | 166.52 | 1.32 | 0.00 | ENSG00000158201 | 1436.59 | -1.95 | 0.00 |
| ENSG00000123131 | 4805.53 | 1.32 | 0.00 | ENSG00000153956 | 174.10 | -1.95 | 0.00 |
| ENSG00000178719 | 8135.41 | 1.32 | 0.00 | ENSG00000136044 | 2344.71 | -1.95 | 0.00 |
| ENSG00000124787 | 307.21 | 1.32 | 0.00 | ENSG00000105270 | 427.20 | -1.95 | 0.00 |
| ENSG00000154096 | 3338.23 | 1.32 | 0.00 | ENSG00000042445 | 4690.76 | -1.95 | 0.00 |
| ENSG00000197653 | 61.01 | 1.32 | 0.00 | ENSG00000204872 | 52.71 | -1.95 | 0.00 |
| ENSG00000151287 | 304.43 | 1.32 | 0.00 | ENSG00000088280 | 514.96 | -1.95 | 0.00 |
| ENSG00000095970 | 204.89 | 1.32 | 0.00 | ENSG00000146122 | 324.20 | -1.96 | 0.00 |
| ENSG00000125726 | 54.32 | 1.32 | 0.00 | ENSG00000104059 | 182.25 | -1.96 | 0.00 |
| ENSG00000171453 | 1437.96 | 1.32 | 0.00 | ENSG00000254709 | 1939.54 | -1.96 | 0.00 |
| ENSG00000146918 | 1462.76 | 1.31 | 0.00 | ENSG00000172137 | 155.32 | -1.96 | 0.00 |
| ENSG00000126457 | 7331.51 | 1.31 | 0.00 | ENSG00000105974 | 1764.59 | -1.96 | 0.00 |
| ENSG00000134107 | 6331.47 | 1.31 | 0.00 | ENSG00000134917 | 78.05 | -1.96 | 0.00 |
| ENSG00000068489 | 839.93 | 1.31 | 0.00 | ENSG00000177455 | 64.54 | -1.97 | 0.00 |
| ENSG00000244405 | 643.60 | 1.31 | 0.00 | ENSG00000197375 | 959.65 | -1.97 | 0.00 |
| ENSG00000279605 | 51.26 | 1.31 | 0.00 | ENSG00000211959 | 1557.11 | -1.97 | 0.00 |
| ENSG00000163507 | 441.16 | 1.31 | 0.00 | ENSG00000123243 | 829.52 | -1.97 | 0.00 |
| ENSG00000114554 | 2927.95 | 1.31 | 0.00 | ENSG00000162520 | 76.39 | -1.97 | 0.00 |
| ENSG00000265415 | 87.81 | 1.31 | 0.00 | ENSG00000118804 | 142.81 | -1.98 | 0.00 |
| ENSG00000170293 | 536.58 | 1.31 | 0.00 | ENSG00000115841 | 205.94 | -1.98 | 0.00 |
| ENSG00000100526 | 691.55 | 1.31 | 0.00 | ENSG00000079335 | 256.15 | -1.98 | 0.00 |
| ENSG00000163597 | 966.54 | 1.31 | 0.00 | ENSG00000183508 | 1095.60 | -1.98 | 0.00 |
| ENSG00000233016 | 1448.07 | 1.31 | 0.00 | ENSG00000172159 | 288.14 | -1.98 | 0.00 |
| ENSG00000219438 | 152.60 | 1.31 | 0.00 | ENSG00000138356 | 122.80 | -1.98 | 0.00 |
| ENSG00000016402 | 1007.07 | 1.31 | 0.00 | ENSG00000268388 | 462.77 | -1.98 | 0.00 |
| ENSG00000072571 | 1006.13 | 1.31 | 0.00 | ENSG00000067113 | 980.10 | -1.98 | 0.00 |
| ENSG00000050438 | 139.37 | 1.31 | 0.00 | ENSG00000162409 | 98.26 | -1.99 | 0.00 |
| ENSG00000073111 | 3430.93 | 1.31 | 0.00 | ENSG00000134955 | 482.47 | -1.99 | 0.00 |
| ENSG00000185480 | 543.45 | 1.31 | 0.00 | ENSG00000211669 | 514.63 | -1.99 | 0.00 |
| ENSG00000106628 | 5476.45 | 1.31 | 0.00 | ENSG00000170476 | 619.80 | -1.99 | 0.00 |
| ENSG00000163393 | 164.79 | 1.30 | 0.00 | ENSG00000188373 | 3288.84 | -1.99 | 0.00 |
| ENSG00000118690 | 126.00 | 1.30 | 0.00 | ENSG00000239855 | 376.17 | -1.99 | 0.00 |
| ENSG00000116670 | 872.49 | 1.30 | 0.00 | ENSG00000197321 | 3038.68 | -2.00 | 0.00 |
| ENSG00000164934 | 2348.22 | 1.30 | 0.00 | ENSG00000010319 | 242.35 | -2.00 | 0.00 |
| ENSG00000117586 | 159.39 | 1.30 | 0.00 | ENSG00000154277 | 201.31 | -2.00 | 0.00 |
| ENSG00000126602 | 4524.59 | 1.30 | 0.00 | ENSG00000186469 | 356.06 | -2.00 | 0.00 |
| ENSG00000161888 | 546.69 | 1.30 | 0.00 | ENSG00000153162 | 81.55 | -2.00 | 0.00 |
| ENSG00000135749 | 550.53 | 1.30 | 0.00 | ENSG00000211967 | 282.76 | -2.00 | 0.00 |
| ENSG00000101670 | 1367.27 | 1.30 | 0.00 | ENSG00000100302 | 396.25 | -2.00 | 0.00 |
| ENSG00000109674 | 156.89 | 1.30 | 0.00 | ENSG00000135709 | 643.69 | -2.00 | 0.00 |
| ENSG00000166508 | 8812.39 | 1.30 | 0.00 | ENSG00000080644 | 92.01 | -2.00 | 0.00 |
| ENSG00000196074 | 57.61 | 1.30 | 0.00 | ENSG00000211650 | 183.65 | -2.01 | 0.00 |
| ENSG00000196296 | 61.17 | 1.30 | 0.00 | ENSG00000137819 | 811.66 | -2.01 | 0.00 |
| ENSG00000265148 | 101.61 | 1.30 | 0.00 | ENSG00000100321 | 135.31 | -2.01 | 0.00 |
| ENSG00000133110 | 6014.20 | 1.30 | 0.00 | ENSG00000211644 | 1154.54 | -2.01 | 0.00 |
| ENSG00000224397 | 135.27 | 1.30 | 0.00 | ENSG00000158560 | 57.59 | -2.02 | 0.00 |
| ENSG00000235173 | 1827.16 | 1.30 | 0.00 | ENSG00000189221 | 5148.33 | -2.02 | 0.00 |
| ENSG00000134815 | 1329.89 | 1.29 | 0.00 | ENSG00000137077 | 671.04 | -2.02 | 0.00 |
| ENSG00000147536 | 349.58 | 1.29 | 0.00 | ENSG00000211598 | 3849.34 | -2.02 | 0.00 |
| ENSG00000234741 | 7075.25 | 1.29 | 0.00 | ENSG00000061455 | 64.03 | -2.02 | 0.00 |
| ENSG00000127585 | 288.33 | 1.29 | 0.00 | ENSG00000143416 | 14271.65 | -2.02 | 0.00 |
| ENSG00000108641 | 355.94 | 1.29 | 0.00 | ENSG00000172572 | 892.02 | -2.02 | 0.00 |
| ENSG00000129295 | 216.65 | 1.29 | 0.00 | ENSG00000211632 | 68.61 | -2.02 | 0.00 |
| ENSG00000143067 | 327.12 | 1.29 | 0.00 | ENSG00000077943 | 175.09 | -2.02 | 0.00 |
| ENSG00000154928 | 202.75 | 1.29 | 0.00 | ENSG00000170323 | 182.65 | -2.03 | 0.00 |
| ENSG00000189046 | 581.46 | 1.29 | 0.00 | ENSG00000198682 | 3792.00 | -2.03 | 0.00 |
| ENSG00000106462 | 1251.98 | 1.29 | 0.00 | ENSG00000166819 | 101.42 | -2.04 | 0.00 |
| ENSG00000160606 | 548.36 | 1.29 | 0.00 | ENSG00000124772 | 247.34 | -2.04 | 0.00 |
| ENSG00000272141 | 471.98 | 1.29 | 0.00 | ENSG00000142875 | 1950.04 | -2.04 | 0.00 |
| ENSG00000153291 | 194.52 | 1.29 | 0.00 | ENSG00000109339 | 65.19 | -2.04 | 0.00 |
| ENSG00000166012 | 2527.38 | 1.29 | 0.00 | ENSG00000164825 | 135.46 | -2.04 | 0.00 |
| ENSG00000264350 | 111.81 | 1.29 | 0.00 | ENSG00000155545 | 1438.03 | -2.04 | 0.00 |
| ENSG00000197457 | 1240.57 | 1.29 | 0.00 | ENSG00000119686 | 253.76 | -2.04 | 0.00 |
| ENSG00000103494 | 323.62 | 1.29 | 0.00 | ENSG00000166866 | 3317.67 | -2.05 | 0.00 |
| ENSG00000056277 | 206.01 | 1.29 | 0.00 | ENSG00000166959 | 380.37 | -2.05 | 0.00 |
| ENSG00000170191 | 428.47 | 1.29 | 0.00 | ENSG00000047457 | 164.03 | -2.05 | 0.00 |
| ENSG00000197989 | 519.63 | 1.28 | 0.00 | ENSG00000165269 | 124.79 | -2.05 | 0.00 |
| ENSG00000230082 | 78.07 | 1.28 | 0.00 | ENSG00000156804 | 1654.92 | -2.05 | 0.00 |
| ENSG00000120889 | 3184.01 | 1.28 | 0.00 | ENSG00000108852 | 67.33 | -2.05 | 0.00 |
| ENSG00000104522 | 6772.45 | 1.28 | 0.00 | ENSG00000170298 | 125.87 | -2.05 | 0.00 |
| ENSG00000203760 | 751.01 | 1.28 | 0.00 | ENSG00000139209 | 173.53 | -2.07 | 0.00 |
| ENSG00000167600 | 6699.33 | 1.28 | 0.00 | ENSG00000146938 | 50.71 | -2.07 | 0.00 |
| ENSG00000213186 | 371.18 | 1.28 | 0.00 | ENSG00000211637 | 649.92 | -2.07 | 0.00 |
| ENSG00000181588 | 1197.87 | 1.28 | 0.00 | ENSG00000227051 | 222.48 | -2.08 | 0.00 |
| ENSG00000256546 | 79.84 | 1.28 | 0.00 | ENSG00000078295 | 53.18 | -2.08 | 0.00 |
| ENSG00000074181 | 2738.11 | 1.28 | 0.00 | ENSG00000188536 | 172.49 | -2.08 | 0.00 |
| ENSG00000064545 | 1718.98 | 1.28 | 0.00 | ENSG00000211639 | 147.61 | -2.08 | 0.00 |
| ENSG00000168542 | 59544.21 | 1.28 | 0.00 | ENSG00000140297 | 4850.72 | -2.08 | 0.00 |
| ENSG00000021300 | 1533.52 | 1.28 | 0.00 | ENSG00000123643 | 1036.54 | -2.08 | 0.00 |
| ENSG00000160867 | 4122.23 | 1.28 | 0.00 | ENSG00000150593 | 3796.01 | -2.08 | 0.00 |
| ENSG00000263266 | 373.69 | 1.28 | 0.00 | ENSG00000116176 | 275.30 | -2.08 | 0.00 |
| ENSG00000121957 | 2252.20 | 1.28 | 0.00 | ENSG00000164128 | 73.43 | -2.08 | 0.00 |
| ENSG00000140104 | 371.21 | 1.27 | 0.00 | ENSG00000130518 | 92.61 | -2.08 | 0.00 |
| ENSG00000234432 | 87.44 | 1.27 | 0.00 | ENSG00000164176 | 939.03 | -2.09 | 0.00 |
| ENSG00000260196 | 437.32 | 1.27 | 0.00 | ENSG00000080031 | 1981.30 | -2.09 | 0.00 |
| ENSG00000144381 | 22686.32 | 1.27 | 0.00 | ENSG00000211949 | 2100.85 | -2.09 | 0.00 |
| ENSG00000054598 | 209.26 | 1.27 | 0.00 | ENSG00000224189 | 478.92 | -2.09 | 0.00 |
| ENSG00000229638 | 229.30 | 1.27 | 0.00 | ENSG00000171724 | 92.92 | -2.09 | 0.00 |
| ENSG00000070404 | 859.53 | 1.27 | 0.00 | ENSG00000164342 | 417.53 | -2.10 | 0.00 |
| ENSG00000261971 | 188.60 | 1.27 | 0.00 | ENSG00000213901 | 72.13 | -2.10 | 0.00 |
| ENSG00000228232 | 289.37 | 1.27 | 0.00 | ENSG00000151882 | 1232.26 | -2.10 | 0.00 |
| ENSG00000115257 | 123.61 | 1.27 | 0.00 | ENSG00000138193 | 1128.66 | -2.10 | 0.00 |
| ENSG00000198915 | 138.60 | 1.27 | 0.00 | ENSG00000078596 | 301.37 | -2.10 | 0.00 |
| ENSG00000073536 | 1271.05 | 1.27 | 0.00 | ENSG00000007312 | 132.42 | -2.11 | 0.00 |
| ENSG00000142765 | 767.01 | 1.27 | 0.00 | ENSG00000131781 | 719.50 | -2.11 | 0.00 |
| ENSG00000112742 | 728.36 | 1.27 | 0.00 | ENSG00000211666 | 1983.68 | -2.11 | 0.00 |
| ENSG00000146521 | 102.39 | 1.27 | 0.00 | ENSG00000168280 | 66.50 | -2.11 | 0.00 |
| ENSG00000177732 | 916.32 | 1.27 | 0.00 | ENSG00000122224 | 64.28 | -2.11 | 0.00 |
| ENSG00000133063 | 152.19 | 1.27 | 0.00 | ENSG00000178031 | 171.03 | -2.11 | 0.00 |
| ENSG00000137563 | 4706.31 | 1.27 | 0.00 | ENSG00000131386 | 112.80 | -2.11 | 0.00 |
| ENSG00000177602 | 196.43 | 1.27 | 0.00 | ENSG00000073711 | 301.38 | -2.11 | 0.00 |
| ENSG00000175928 | 81.45 | 1.27 | 0.00 | ENSG00000121933 | 98.91 | -2.12 | 0.00 |
| ENSG00000143476 | 815.81 | 1.26 | 0.00 | ENSG00000226777 | 80.83 | -2.12 | 0.00 |
| ENSG00000128408 | 65.10 | 1.26 | 0.00 | ENSG00000174514 | 536.55 | -2.12 | 0.00 |
| ENSG00000128050 | 7186.81 | 1.26 | 0.00 | ENSG00000211592 | 42259.68 | -2.12 | 0.00 |
| ENSG00000101182 | 11346.90 | 1.26 | 0.00 | ENSG00000159307 | 134.79 | -2.12 | 0.00 |
| ENSG00000157388 | 557.29 | 1.26 | 0.00 | ENSG00000197629 | 996.77 | -2.12 | 0.00 |
| ENSG00000110104 | 1340.57 | 1.26 | 0.00 | ENSG00000149534 | 51.49 | -2.12 | 0.00 |
| ENSG00000159674 | 2615.16 | 1.26 | 0.00 | ENSG00000182916 | 54.33 | -2.12 | 0.00 |
| ENSG00000198134 | 58.01 | 1.26 | 0.00 | ENSG00000166510 | 539.84 | -2.13 | 0.00 |
| ENSG00000102924 | 66.59 | 1.26 | 0.00 | ENSG00000136274 | 59.12 | -2.13 | 0.00 |
| ENSG00000105982 | 159.51 | 1.26 | 0.00 | ENSG00000241351 | 3066.50 | -2.13 | 0.00 |
| ENSG00000135446 | 3858.88 | 1.26 | 0.00 | ENSG00000184828 | 952.12 | -2.13 | 0.00 |
| ENSG00000229132 | 53.24 | 1.26 | 0.00 | ENSG00000243955 | 68.94 | -2.13 | 0.00 |
| ENSG00000278291 | 139.99 | 1.26 | 0.00 | ENSG00000172831 | 8838.53 | -2.13 | 0.00 |
| ENSG00000187801 | 80.61 | 1.26 | 0.00 | ENSG00000211649 | 476.09 | -2.13 | 0.00 |
| ENSG00000112378 | 16297.62 | 1.26 | 0.00 | ENSG00000164850 | 119.57 | -2.13 | 0.00 |
| ENSG00000077063 | 392.98 | 1.26 | 0.00 | ENSG00000153902 | 180.21 | -2.13 | 0.00 |
| ENSG00000224186 | 64.60 | 1.26 | 0.00 | ENSG00000071967 | 1785.55 | -2.13 | 0.00 |
| ENSG00000279495 | 100.13 | 1.26 | 0.00 | ENSG00000164161 | 147.10 | -2.14 | 0.00 |
| ENSG00000165661 | 1146.06 | 1.26 | 0.00 | ENSG00000211976 | 297.03 | -2.14 | 0.00 |
| ENSG00000231721 | 179.73 | 1.26 | 0.00 | ENSG00000162614 | 389.86 | -2.14 | 0.00 |
| ENSG00000168071 | 2165.62 | 1.26 | 0.00 | ENSG00000205795 | 92.44 | -2.14 | 0.00 |
| ENSG00000183763 | 342.52 | 1.25 | 0.00 | ENSG00000196924 | 31673.42 | -2.14 | 0.00 |
| ENSG00000080839 | 657.81 | 1.25 | 0.00 | ENSG00000171533 | 59.05 | -2.14 | 0.00 |
| ENSG00000169908 | 5086.28 | 1.25 | 0.00 | ENSG00000109265 | 503.46 | -2.15 | 0.00 |
| ENSG00000130332 | 3229.91 | 1.25 | 0.00 | ENSG00000198947 | 525.30 | -2.15 | 0.00 |
| ENSG00000158042 | 2179.70 | 1.25 | 0.00 | ENSG00000122971 | 2457.62 | -2.15 | 0.00 |
| ENSG00000264112 | 448.30 | 1.25 | 0.00 | ENSG00000152217 | 209.48 | -2.15 | 0.00 |
| ENSG00000178999 | 1132.04 | 1.25 | 0.00 | ENSG00000138744 | 1341.24 | -2.15 | 0.00 |
| ENSG00000106686 | 112.28 | 1.25 | 0.00 | ENSG00000058668 | 2500.98 | -2.16 | 0.00 |
| ENSG00000272455 | 86.31 | 1.25 | 0.00 | ENSG00000211966 | 1633.44 | -2.16 | 0.00 |
| ENSG00000125772 | 2614.57 | 1.25 | 0.00 | ENSG00000013297 | 63.95 | -2.16 | 0.00 |
| ENSG00000185761 | 253.49 | 1.25 | 0.00 | ENSG00000176472 | 145.94 | -2.16 | 0.00 |
| ENSG00000130701 | 570.31 | 1.25 | 0.00 | ENSG00000177098 | 82.37 | -2.16 | 0.00 |
| ENSG00000121152 | 899.19 | 1.25 | 0.00 | ENSG00000112186 | 187.23 | -2.16 | 0.00 |
| ENSG00000196081 | 56.36 | 1.25 | 0.00 | ENSG00000115616 | 954.84 | -2.16 | 0.00 |
| ENSG00000133627 | 372.73 | 1.25 | 0.00 | ENSG00000169764 | 4202.13 | -2.16 | 0.00 |
| ENSG00000182481 | 4630.13 | 1.25 | 0.00 | ENSG00000174640 | 1095.17 | -2.17 | 0.00 |
| ENSG00000178715 | 91.20 | 1.24 | 0.00 | ENSG00000153993 | 51.38 | -2.17 | 0.00 |
| ENSG00000174365 | 464.19 | 1.24 | 0.00 | ENSG00000171408 | 56.12 | -2.17 | 0.00 |
| ENSG00000233762 | 324.98 | 1.24 | 0.00 | ENSG00000103647 | 53.79 | -2.17 | 0.00 |
| ENSG00000245149 | 71.10 | 1.24 | 0.00 | ENSG00000213088 | 302.80 | -2.17 | 0.00 |
| ENSG00000233328 | 53.11 | 1.24 | 0.00 | ENSG00000198125 | 148.19 | -2.17 | 0.00 |
| ENSG00000185238 | 883.09 | 1.24 | 0.00 | ENSG00000130224 | 53.49 | -2.18 | 0.00 |
| ENSG00000196411 | 5582.41 | 1.24 | 0.00 | ENSG00000179914 | 3540.86 | -2.18 | 0.00 |
| ENSG00000109805 | 807.71 | 1.24 | 0.00 | ENSG00000181804 | 124.72 | -2.18 | 0.00 |
| ENSG00000271533 | 185.86 | 1.24 | 0.00 | ENSG00000121807 | 89.35 | -2.18 | 0.00 |
| ENSG00000198276 | 2328.61 | 1.24 | 0.00 | ENSG00000178343 | 63.31 | -2.18 | 0.00 |
| ENSG00000111247 | 563.93 | 1.24 | 0.00 | ENSG00000149451 | 134.57 | -2.18 | 0.00 |
| ENSG00000280206 | 283.54 | 1.24 | 0.00 | ENSG00000211664 | 149.47 | -2.18 | 0.00 |
| ENSG00000164070 | 483.69 | 1.24 | 0.00 | ENSG00000266036 | 60.32 | -2.18 | 0.00 |
| ENSG00000278023 | 51.41 | 1.24 | 0.00 | ENSG00000095637 | 2218.78 | -2.18 | 0.00 |
| ENSG00000146263 | 571.08 | 1.24 | 0.00 | ENSG00000198121 | 368.38 | -2.19 | 0.00 |
| ENSG00000180758 | 898.29 | 1.24 | 0.00 | ENSG00000112964 | 126.76 | -2.19 | 0.00 |
| ENSG00000104738 | 5253.90 | 1.24 | 0.00 | ENSG00000102886 | 456.95 | -2.19 | 0.00 |
| ENSG00000055044 | 3242.76 | 1.24 | 0.00 | ENSG00000185774 | 59.67 | -2.19 | 0.00 |
| ENSG00000154839 | 382.04 | 1.24 | 0.00 | ENSG00000154553 | 950.53 | -2.19 | 0.00 |
| ENSG00000012048 | 945.74 | 1.24 | 0.00 | ENSG00000137757 | 270.87 | -2.19 | 0.00 |
| ENSG00000102595 | 883.59 | 1.24 | 0.00 | ENSG00000112782 | 2281.85 | -2.19 | 0.00 |
| ENSG00000237649 | 1759.90 | 1.24 | 0.00 | ENSG00000186868 | 81.85 | -2.19 | 0.00 |
| ENSG00000279407 | 81.14 | 1.24 | 0.00 | ENSG00000175600 | 111.86 | -2.19 | 0.00 |
| ENSG00000106105 | 5954.72 | 1.23 | 0.00 | ENSG00000007216 | 420.82 | -2.19 | 0.00 |
| ENSG00000205089 | 541.37 | 1.23 | 0.00 | ENSG00000174236 | 67.42 | -2.19 | 0.00 |
| ENSG00000160796 | 5280.01 | 1.23 | 0.00 | ENSG00000172828 | 1863.08 | -2.20 | 0.00 |
| ENSG00000129810 | 276.55 | 1.23 | 0.00 | ENSG00000066468 | 732.70 | -2.20 | 0.00 |
| ENSG00000118420 | 209.66 | 1.23 | 0.00 | ENSG00000139117 | 228.89 | -2.20 | 0.00 |
| ENSG00000241741 | 56.89 | 1.23 | 0.00 | ENSG00000241755 | 703.89 | -2.20 | 0.00 |
| ENSG00000152253 | 402.20 | 1.23 | 0.00 | ENSG00000187288 | 138.62 | -2.21 | 0.00 |
| ENSG00000151364 | 650.33 | 1.23 | 0.00 | ENSG00000151320 | 118.34 | -2.21 | 0.00 |
| ENSG00000035499 | 562.63 | 1.23 | 0.00 | ENSG00000152583 | 3057.47 | -2.21 | 0.00 |
| ENSG00000163918 | 828.08 | 1.23 | 0.00 | ENSG00000166482 | 1774.59 | -2.21 | 0.00 |
| ENSG00000159685 | 557.69 | 1.23 | 0.00 | ENSG00000196502 | 877.84 | -2.22 | 0.00 |
| ENSG00000149150 | 1258.71 | 1.23 | 0.00 | ENSG00000125148 | 2877.36 | -2.22 | 0.00 |
| ENSG00000180385 | 122.50 | 1.23 | 0.00 | ENSG00000172724 | 161.04 | -2.22 | 0.00 |
| ENSG00000178460 | 74.22 | 1.23 | 0.00 | ENSG00000168016 | 1379.56 | -2.22 | 0.00 |
| ENSG00000143498 | 204.98 | 1.23 | 0.00 | ENSG00000241294 | 359.12 | -2.23 | 0.00 |
| ENSG00000225855 | 230.43 | 1.23 | 0.00 | ENSG00000134533 | 137.31 | -2.23 | 0.00 |
| ENSG00000147955 | 5238.67 | 1.23 | 0.00 | ENSG00000144712 | 52.31 | -2.23 | 0.00 |
| ENSG00000005379 | 709.48 | 1.23 | 0.00 | ENSG00000223648 | 76.38 | -2.24 | 0.00 |
| ENSG00000113460 | 1411.87 | 1.23 | 0.00 | ENSG00000211651 | 872.12 | -2.24 | 0.00 |
| ENSG00000237214 | 88.90 | 1.23 | 0.00 | ENSG00000147614 | 52.16 | -2.24 | 0.00 |
| ENSG00000215440 | 572.71 | 1.23 | 0.00 | ENSG00000269936 | 329.77 | -2.24 | 0.00 |
| ENSG00000214182 | 243.70 | 1.23 | 0.00 | ENSG00000137273 | 243.79 | -2.24 | 0.00 |
| ENSG00000178878 | 1697.85 | 1.23 | 0.00 | ENSG00000176928 | 74.36 | -2.24 | 0.00 |
| ENSG00000059588 | 1780.91 | 1.22 | 0.00 | ENSG00000159176 | 10409.30 | -2.24 | 0.00 |
| ENSG00000156970 | 951.18 | 1.22 | 0.00 | ENSG00000135916 | 22558.85 | -2.25 | 0.00 |
| ENSG00000164430 | 352.26 | 1.22 | 0.00 | ENSG00000211673 | 659.53 | -2.26 | 0.00 |
| ENSG00000261553 | 81.55 | 1.22 | 0.00 | ENSG00000197766 | 1206.60 | -2.26 | 0.00 |
| ENSG00000261804 | 52.01 | 1.22 | 0.00 | ENSG00000165821 | 83.25 | -2.26 | 0.00 |
| ENSG00000181524 | 204.47 | 1.22 | 0.00 | ENSG00000171951 | 152.60 | -2.26 | 0.00 |
| ENSG00000122711 | 4647.54 | 1.22 | 0.00 | ENSG00000175906 | 109.75 | -2.26 | 0.00 |
| ENSG00000164045 | 750.86 | 1.22 | 0.00 | ENSG00000211943 | 1113.20 | -2.26 | 0.00 |
| ENSG00000273045 | 162.31 | 1.22 | 0.00 | ENSG00000211638 | 454.00 | -2.26 | 0.00 |
| ENSG00000160813 | 809.79 | 1.22 | 0.00 | ENSG00000117472 | 15341.83 | -2.26 | 0.00 |
| ENSG00000265298 | 244.74 | 1.22 | 0.00 | ENSG00000143867 | 58.59 | -2.27 | 0.00 |
| ENSG00000080573 | 715.78 | 1.22 | 0.00 | ENSG00000128573 | 202.65 | -2.27 | 0.00 |
| ENSG00000087077 | 2628.42 | 1.22 | 0.00 | ENSG00000211942 | 137.96 | -2.27 | 0.00 |
| ENSG00000126787 | 842.51 | 1.22 | 0.00 | ENSG00000006740 | 816.07 | -2.27 | 0.00 |
| ENSG00000138101 | 383.28 | 1.22 | 0.00 | ENSG00000119938 | 97.91 | -2.27 | 0.00 |
| ENSG00000149554 | 1002.32 | 1.22 | 0.00 | ENSG00000211670 | 239.76 | -2.27 | 0.00 |
| ENSG00000135763 | 889.50 | 1.22 | 0.00 | ENSG00000066629 | 334.31 | -2.28 | 0.00 |
| ENSG00000182240 | 6714.89 | 1.22 | 0.00 | ENSG00000172915 | 114.12 | -2.28 | 0.00 |
| ENSG00000124571 | 2650.82 | 1.22 | 0.00 | ENSG00000074410 | 5538.94 | -2.28 | 0.00 |
| ENSG00000173156 | 576.30 | 1.22 | 0.00 | ENSG00000108242 | 129.55 | -2.28 | 0.00 |
| ENSG00000000460 | 362.34 | 1.21 | 0.00 | ENSG00000124440 | 105.76 | -2.29 | 0.00 |
| ENSG00000174938 | 2148.49 | 1.21 | 0.00 | ENSG00000169715 | 1910.50 | -2.29 | 0.00 |
| ENSG00000143847 | 53.73 | 1.21 | 0.00 | ENSG00000211972 | 118.14 | -2.29 | 0.00 |
| ENSG00000133316 | 1314.21 | 1.21 | 0.00 | ENSG00000275395 | 38311.72 | -2.29 | 0.00 |
| ENSG00000088451 | 767.44 | 1.21 | 0.00 | ENSG00000188833 | 988.63 | -2.29 | 0.00 |
| ENSG00000185513 | 255.63 | 1.21 | 0.00 | ENSG00000106034 | 361.43 | -2.30 | 0.00 |
| ENSG00000106397 | 7346.45 | 1.21 | 0.00 | ENSG00000143603 | 149.81 | -2.30 | 0.00 |
| ENSG00000117399 | 2788.29 | 1.21 | 0.00 | ENSG00000135437 | 88.62 | -2.30 | 0.00 |
| ENSG00000005448 | 403.49 | 1.21 | 0.00 | ENSG00000140323 | 231.11 | -2.30 | 0.00 |
| ENSG00000180817 | 11490.12 | 1.21 | 0.00 | ENSG00000108405 | 86.07 | -2.31 | 0.00 |
| ENSG00000165689 | 1844.32 | 1.21 | 0.00 | ENSG00000224373 | 997.57 | -2.31 | 0.00 |
| ENSG00000006015 | 1546.40 | 1.21 | 0.00 | ENSG00000160307 | 120.01 | -2.32 | 0.00 |
| ENSG00000267519 | 859.31 | 1.21 | 0.00 | ENSG00000157111 | 723.16 | -2.32 | 0.00 |
| ENSG00000069482 | 369.55 | 1.21 | 0.00 | ENSG00000118526 | 168.38 | -2.32 | 0.00 |
| ENSG00000158402 | 339.15 | 1.21 | 0.00 | ENSG00000187091 | 822.56 | -2.32 | 0.00 |
| ENSG00000167992 | 54.41 | 1.21 | 0.00 | ENSG00000165457 | 273.72 | -2.32 | 0.00 |
| ENSG00000139343 | 2388.38 | 1.21 | 0.00 | ENSG00000144063 | 508.39 | -2.32 | 0.00 |
| ENSG00000165810 | 310.23 | 1.21 | 0.00 | ENSG00000205683 | 76.34 | -2.32 | 0.00 |
| ENSG00000214706 | 3141.19 | 1.21 | 0.00 | ENSG00000074276 | 2219.84 | -2.32 | 0.00 |
| ENSG00000249115 | 753.20 | 1.21 | 0.00 | ENSG00000125775 | 2455.47 | -2.33 | 0.00 |
| ENSG00000197763 | 286.69 | 1.20 | 0.00 | ENSG00000266200 | 129.20 | -2.33 | 0.00 |
| ENSG00000163811 | 2490.63 | 1.20 | 0.00 | ENSG00000240864 | 472.91 | -2.33 | 0.00 |
| ENSG00000260920 | 88.83 | 1.20 | 0.00 | ENSG00000211445 | 1373.10 | -2.33 | 0.00 |
| ENSG00000101294 | 8964.12 | 1.20 | 0.00 | ENSG00000095110 | 728.58 | -2.33 | 0.00 |
| ENSG00000049449 | 5097.15 | 1.20 | 0.00 | ENSG00000181092 | 77.64 | -2.34 | 0.01 |
| ENSG00000258512 | 63.08 | 1.20 | 0.00 | ENSG00000120885 | 2803.39 | -2.34 | 0.00 |
| ENSG00000165794 | 171.16 | 1.20 | 0.01 | ENSG00000211965 | 543.21 | -2.34 | 0.00 |
| ENSG00000230202 | 441.34 | 1.20 | 0.00 | ENSG00000240771 | 266.95 | -2.34 | 0.00 |
| ENSG00000133315 | 1644.68 | 1.20 | 0.00 | ENSG00000179674 | 382.63 | -2.34 | 0.00 |
| ENSG00000101199 | 4474.73 | 1.20 | 0.00 | ENSG00000170703 | 90.15 | -2.35 | 0.00 |
| ENSG00000149636 | 1100.64 | 1.20 | 0.00 | ENSG00000165449 | 507.97 | -2.36 | 0.00 |
| ENSG00000139998 | 3269.88 | 1.20 | 0.00 | ENSG00000134240 | 9210.42 | -2.36 | 0.00 |
| ENSG00000092853 | 299.28 | 1.20 | 0.00 | ENSG00000239951 | 3192.64 | -2.36 | 0.00 |
| ENSG00000064666 | 6703.48 | 1.20 | 0.00 | ENSG00000278535 | 1774.71 | -2.36 | 0.00 |
| ENSG00000170801 | 1757.88 | 1.20 | 0.00 | ENSG00000244575 | 360.35 | -2.36 | 0.00 |
| ENSG00000160214 | 1648.44 | 1.20 | 0.00 | ENSG00000187193 | 859.29 | -2.36 | 0.00 |
| ENSG00000084207 | 22197.41 | 1.20 | 0.00 | ENSG00000254827 | 340.08 | -2.37 | 0.00 |
| ENSG00000262580 | 129.03 | 1.20 | 0.00 | ENSG00000102010 | 71.83 | -2.37 | 0.00 |
| ENSG00000167523 | 388.60 | 1.20 | 0.00 | ENSG00000116833 | 491.78 | -2.37 | 0.00 |
| ENSG00000163629 | 657.47 | 1.20 | 0.00 | ENSG00000204136 | 101.52 | -2.37 | 0.00 |
| ENSG00000264577 | 64.78 | 1.19 | 0.00 | ENSG00000158125 | 1189.29 | -2.37 | 0.00 |
| ENSG00000196187 | 6953.98 | 1.19 | 0.00 | ENSG00000182175 | 346.36 | -2.38 | 0.00 |
| ENSG00000141101 | 2879.35 | 1.19 | 0.00 | ENSG00000072163 | 909.62 | -2.38 | 0.00 |
| ENSG00000130193 | 2749.33 | 1.19 | 0.00 | ENSG00000176387 | 5745.22 | -2.38 | 0.00 |
| ENSG00000104881 | 1436.99 | 1.19 | 0.00 | ENSG00000121898 | 432.72 | -2.38 | 0.00 |
| ENSG00000101945 | 711.36 | 1.19 | 0.00 | ENSG00000197361 | 72.03 | -2.39 | 0.00 |
| ENSG00000114993 | 2719.19 | 1.19 | 0.00 | ENSG00000079385 | 10339.33 | -2.39 | 0.00 |
| ENSG00000148335 | 1034.63 | 1.19 | 0.00 | ENSG00000108187 | 976.23 | -2.39 | 0.00 |
| ENSG00000198901 | 2069.68 | 1.19 | 0.00 | ENSG00000099866 | 61.46 | -2.39 | 0.00 |
| ENSG00000273117 | 55.69 | 1.19 | 0.00 | ENSG00000092421 | 1097.81 | -2.39 | 0.00 |
| ENSG00000185252 | 641.82 | 1.19 | 0.00 | ENSG00000166501 | 265.29 | -2.39 | 0.00 |
| ENSG00000065833 | 1072.79 | 1.19 | 0.00 | ENSG00000012124 | 141.19 | -2.40 | 0.00 |
| ENSG00000141076 | 2185.52 | 1.19 | 0.00 | ENSG00000174099 | 830.53 | -2.40 | 0.00 |
| ENSG00000218891 | 482.28 | 1.19 | 0.00 | ENSG00000135218 | 417.79 | -2.40 | 0.00 |
| ENSG00000198780 | 138.53 | 1.19 | 0.00 | ENSG00000069535 | 395.91 | -2.41 | 0.00 |
| ENSG00000117385 | 958.63 | 1.19 | 0.00 | ENSG00000166920 | 5540.56 | -2.41 | 0.00 |
| ENSG00000156219 | 87.44 | 1.19 | 0.00 | ENSG00000152763 | 137.20 | -2.41 | 0.00 |
| ENSG00000179761 | 237.41 | 1.18 | 0.00 | ENSG00000090659 | 277.53 | -2.41 | 0.00 |
| ENSG00000013573 | 1121.03 | 1.18 | 0.00 | ENSG00000188175 | 855.45 | -2.41 | 0.00 |
| ENSG00000204387 | 3152.68 | 1.18 | 0.00 | ENSG00000145681 | 109.81 | -2.41 | 0.00 |
| ENSG00000125630 | 1459.20 | 1.18 | 0.00 | ENSG00000109846 | 492.45 | -2.41 | 0.00 |
| ENSG00000142684 | 205.43 | 1.18 | 0.00 | ENSG00000130294 | 86.19 | -2.42 | 0.00 |
| ENSG00000126453 | 921.18 | 1.18 | 0.00 | ENSG00000211968 | 109.20 | -2.42 | 0.00 |
| ENSG00000235605 | 50.82 | 1.18 | 0.00 | ENSG00000108823 | 71.44 | -2.42 | 0.00 |
| ENSG00000171793 | 1558.95 | 1.18 | 0.00 | ENSG00000016490 | 15840.74 | -2.42 | 0.00 |
| ENSG00000165886 | 301.18 | 1.18 | 0.00 | ENSG00000182168 | 117.24 | -2.43 | 0.00 |
| ENSG00000137135 | 538.46 | 1.18 | 0.00 | ENSG00000214814 | 247.52 | -2.43 | 0.00 |
| ENSG00000123473 | 831.08 | 1.18 | 0.00 | ENSG00000105369 | 426.33 | -2.43 | 0.00 |
| ENSG00000107338 | 825.70 | 1.18 | 0.00 | ENSG00000154262 | 53.53 | -2.43 | 0.00 |
| ENSG00000222009 | 110.84 | 1.18 | 0.00 | ENSG00000162817 | 1363.51 | -2.44 | 0.00 |
| ENSG00000279978 | 374.64 | 1.18 | 0.00 | ENSG00000204099 | 443.80 | -2.44 | 0.00 |
| ENSG00000272752 | 82.44 | 1.18 | 0.00 | ENSG00000148357 | 665.48 | -2.44 | 0.00 |
| ENSG00000007038 | 110.36 | 1.18 | 0.02 | ENSG00000151090 | 309.70 | -2.44 | 0.00 |
| ENSG00000214826 | 117.98 | 1.18 | 0.00 | ENSG00000211947 | 623.95 | -2.44 | 0.00 |
| ENSG00000213397 | 113.45 | 1.18 | 0.00 | ENSG00000132563 | 70.29 | -2.45 | 0.00 |
| ENSG00000106268 | 827.60 | 1.17 | 0.00 | ENSG00000167641 | 283.38 | -2.45 | 0.00 |
| ENSG00000109576 | 281.80 | 1.17 | 0.00 | ENSG00000175329 | 949.79 | -2.46 | 0.00 |
| ENSG00000189057 | 749.23 | 1.17 | 0.00 | ENSG00000100079 | 374.70 | -2.46 | 0.00 |
| ENSG00000250565 | 175.21 | 1.17 | 0.00 | ENSG00000211941 | 634.82 | -2.46 | 0.00 |
| ENSG00000174177 | 766.05 | 1.17 | 0.00 | ENSG00000118308 | 161.37 | -2.46 | 0.00 |
| ENSG00000068438 | 1673.74 | 1.17 | 0.00 | ENSG00000154721 | 218.66 | -2.46 | 0.00 |
| ENSG00000100814 | 1869.30 | 1.17 | 0.00 | ENSG00000154822 | 306.03 | -2.46 | 0.00 |
| ENSG00000176208 | 326.03 | 1.17 | 0.00 | ENSG00000099834 | 5947.18 | -2.46 | 0.00 |
| ENSG00000261040 | 74.39 | 1.17 | 0.00 | ENSG00000240382 | 461.93 | -2.47 | 0.00 |
| ENSG00000172315 | 1370.94 | 1.17 | 0.00 | ENSG00000135702 | 201.75 | -2.47 | 0.00 |
| ENSG00000176809 | 187.02 | 1.17 | 0.00 | ENSG00000184347 | 646.09 | -2.47 | 0.00 |
| ENSG00000196352 | 5987.84 | 1.17 | 0.00 | ENSG00000137265 | 230.79 | -2.47 | 0.00 |
| ENSG00000165732 | 5961.12 | 1.17 | 0.00 | ENSG00000181617 | 84.34 | -2.48 | 0.00 |
| ENSG00000162745 | 905.37 | 1.17 | 0.00 | ENSG00000136826 | 3738.98 | -2.48 | 0.00 |
| ENSG00000064692 | 218.22 | 1.17 | 0.00 | ENSG00000137094 | 228.37 | -2.48 | 0.00 |
| ENSG00000214279 | 54.10 | 1.16 | 0.00 | ENSG00000256643 | 76.18 | -2.48 | 0.00 |
| ENSG00000196976 | 906.90 | 1.16 | 0.00 | ENSG00000197614 | 334.34 | -2.49 | 0.00 |
| ENSG00000184445 | 1066.35 | 1.16 | 0.00 | ENSG00000197380 | 188.19 | -2.49 | 0.00 |
| ENSG00000185834 | 51.06 | 1.16 | 0.00 | ENSG00000064787 | 1947.59 | -2.49 | 0.00 |
| ENSG00000189343 | 511.16 | 1.16 | 0.00 | ENSG00000162391 | 61.95 | -2.49 | 0.00 |
| ENSG00000197785 | 1632.62 | 1.16 | 0.00 | ENSG00000101680 | 158.11 | -2.49 | 0.00 |
| ENSG00000146731 | 9859.26 | 1.16 | 0.00 | ENSG00000057704 | 1086.98 | -2.50 | 0.00 |
| ENSG00000145386 | 1781.31 | 1.16 | 0.00 | ENSG00000156738 | 140.80 | -2.50 | 0.00 |
| ENSG00000249395 | 212.97 | 1.16 | 0.00 | ENSG00000225335 | 71.63 | -2.50 | 0.00 |
| ENSG00000120685 | 3942.85 | 1.16 | 0.00 | ENSG00000180155 | 128.65 | -2.51 | 0.00 |
| ENSG00000106125 | 70.98 | 1.16 | 0.00 | ENSG00000174827 | 54.46 | -2.51 | 0.00 |
| ENSG00000103064 | 765.47 | 1.16 | 0.00 | ENSG00000196196 | 312.29 | -2.51 | 0.00 |
| ENSG00000171159 | 2364.56 | 1.16 | 0.00 | ENSG00000241244 | 84.39 | -2.51 | 0.00 |
| ENSG00000260942 | 89.18 | 1.16 | 0.00 | ENSG00000072657 | 81.19 | -2.51 | 0.00 |
| ENSG00000163599 | 73.52 | 1.16 | 0.00 | ENSG00000105784 | 76.34 | -2.51 | 0.00 |
| ENSG00000093010 | 5135.13 | 1.16 | 0.00 | ENSG00000198865 | 58.64 | -2.51 | 0.00 |
| ENSG00000065057 | 1334.93 | 1.16 | 0.00 | ENSG00000150471 | 87.14 | -2.51 | 0.00 |
| ENSG00000260261 | 67.44 | 1.16 | 0.00 | ENSG00000112818 | 5075.78 | -2.52 | 0.00 |
| ENSG00000272405 | 1152.29 | 1.15 | 0.00 | ENSG00000138678 | 368.50 | -2.52 | 0.00 |
| ENSG00000196839 | 432.48 | 1.15 | 0.00 | ENSG00000108700 | 80.89 | -2.52 | 0.00 |
| ENSG00000132821 | 145.96 | 1.15 | 0.00 | ENSG00000135678 | 776.13 | -2.52 | 0.00 |
| ENSG00000106263 | 11421.96 | 1.15 | 0.00 | ENSG00000172935 | 431.74 | -2.53 | 0.00 |
| ENSG00000101132 | 841.90 | 1.15 | 0.00 | ENSG00000185432 | 2077.32 | -2.54 | 0.00 |
| ENSG00000138316 | 315.30 | 1.15 | 0.00 | ENSG00000211934 | 641.58 | -2.54 | 0.00 |
| ENSG00000149257 | 7633.95 | 1.15 | 0.00 | ENSG00000053438 | 84.71 | -2.54 | 0.00 |
| ENSG00000064102 | 1160.12 | 1.15 | 0.00 | ENSG00000089199 | 140.97 | -2.54 | 0.00 |
| ENSG00000259994 | 66.67 | 1.15 | 0.00 | ENSG00000165410 | 509.23 | -2.55 | 0.00 |
| ENSG00000158062 | 645.36 | 1.15 | 0.00 | ENSG00000099139 | 585.37 | -2.55 | 0.00 |
| ENSG00000167105 | 457.87 | 1.15 | 0.00 | ENSG00000211625 | 183.51 | -2.55 | 0.00 |
| ENSG00000101407 | 1525.91 | 1.15 | 0.00 | ENSG00000188738 | 178.24 | -2.55 | 0.00 |
| ENSG00000250899 | 181.94 | 1.15 | 0.00 | ENSG00000243063 | 56.42 | -2.55 | 0.00 |
| ENSG00000132436 | 651.28 | 1.15 | 0.00 | ENSG00000271447 | 429.12 | -2.55 | 0.00 |
| ENSG00000167291 | 2964.22 | 1.15 | 0.00 | ENSG00000169860 | 375.86 | -2.56 | 0.00 |
| ENSG00000137331 | 6310.69 | 1.15 | 0.00 | ENSG00000137726 | 478.66 | -2.56 | 0.00 |
| ENSG00000188706 | 5062.95 | 1.15 | 0.00 | ENSG00000144619 | 102.99 | -2.56 | 0.00 |
| ENSG00000161692 | 366.78 | 1.15 | 0.00 | ENSG00000046653 | 145.52 | -2.56 | 0.00 |
| ENSG00000105281 | 9387.29 | 1.15 | 0.00 | ENSG00000188783 | 788.93 | -2.56 | 0.00 |
| ENSG00000186529 | 662.36 | 1.15 | 0.00 | ENSG00000176533 | 138.93 | -2.56 | 0.00 |
| ENSG00000198298 | 148.69 | 1.15 | 0.00 | ENSG00000181374 | 113.04 | -2.57 | 0.00 |
| ENSG00000162782 | 80.63 | 1.15 | 0.00 | ENSG00000134121 | 139.93 | -2.58 | 0.00 |
| ENSG00000146904 | 1367.68 | 1.15 | 0.00 | ENSG00000160408 | 1799.44 | -2.58 | 0.00 |
| ENSG00000120699 | 1257.08 | 1.15 | 0.00 | ENSG00000108924 | 109.62 | -2.58 | 0.00 |
| ENSG00000102024 | 1752.56 | 1.15 | 0.00 | ENSG00000183134 | 82.98 | -2.59 | 0.00 |
| ENSG00000101230 | 78.60 | 1.15 | 0.00 | ENSG00000186417 | 80.55 | -2.59 | 0.00 |
| ENSG00000162639 | 607.23 | 1.15 | 0.00 | ENSG00000160191 | 1057.52 | -2.59 | 0.00 |
| ENSG00000131153 | 1078.20 | 1.15 | 0.00 | ENSG00000112175 | 96.25 | -2.60 | 0.00 |
| ENSG00000168078 | 824.43 | 1.15 | 0.00 | ENSG00000118407 | 123.28 | -2.60 | 0.00 |
| ENSG00000048162 | 1307.66 | 1.15 | 0.00 | ENSG00000117322 | 216.01 | -2.60 | 0.00 |
| ENSG00000184661 | 395.55 | 1.15 | 0.00 | ENSG00000155980 | 62.95 | -2.60 | 0.00 |
| ENSG00000153395 | 2317.40 | 1.15 | 0.00 | ENSG00000198467 | 6587.16 | -2.60 | 0.00 |
| ENSG00000131620 | 1325.34 | 1.15 | 0.00 | ENSG00000175356 | 198.89 | -2.60 | 0.00 |
| ENSG00000122565 | 7857.37 | 1.15 | 0.00 | ENSG00000178462 | 331.60 | -2.61 | 0.00 |
| ENSG00000224578 | 182.53 | 1.14 | 0.00 | ENSG00000244734 | 837.18 | -2.61 | 0.00 |
| ENSG00000225489 | 173.24 | 1.14 | 0.00 | ENSG00000211895 | 57245.11 | -2.61 | 0.00 |
| ENSG00000140859 | 676.71 | 1.14 | 0.00 | ENSG00000078804 | 2033.52 | -2.61 | 0.00 |
| ENSG00000125835 | 10420.93 | 1.14 | 0.00 | ENSG00000189129 | 82.79 | -2.62 | 0.00 |
| ENSG00000123179 | 1650.29 | 1.14 | 0.00 | ENSG00000187097 | 2748.30 | -2.62 | 0.00 |
| ENSG00000165171 | 302.53 | 1.14 | 0.00 | ENSG00000113805 | 68.44 | -2.63 | 0.00 |
| ENSG00000175265 | 428.83 | 1.14 | 0.00 | ENSG00000107562 | 1095.84 | -2.63 | 0.00 |
| ENSG00000134901 | 206.17 | 1.14 | 0.00 | ENSG00000174944 | 107.03 | -2.63 | 0.00 |
| ENSG00000276085 | 85.75 | 1.14 | 0.00 | ENSG00000172594 | 952.88 | -2.64 | 0.00 |
| ENSG00000166171 | 647.37 | 1.14 | 0.00 | ENSG00000211933 | 54.77 | -2.64 | 0.00 |
| ENSG00000123136 | 4051.03 | 1.14 | 0.00 | ENSG00000186642 | 167.11 | -2.65 | 0.00 |
| ENSG00000253729 | 9107.16 | 1.14 | 0.00 | ENSG00000077157 | 2664.00 | -2.65 | 0.00 |
| ENSG00000110911 | 3481.62 | 1.14 | 0.00 | ENSG00000105894 | 116.36 | -2.65 | 0.00 |
| ENSG00000221955 | 788.54 | 1.14 | 0.00 | ENSG00000189056 | 69.23 | -2.66 | 0.00 |
| ENSG00000132646 | 6443.56 | 1.14 | 0.00 | ENSG00000167676 | 545.70 | -2.66 | 0.00 |
| ENSG00000136854 | 1376.68 | 1.14 | 0.00 | ENSG00000124491 | 685.93 | -2.66 | 0.00 |
| ENSG00000105821 | 1397.92 | 1.14 | 0.00 | ENSG00000127951 | 919.86 | -2.66 | 0.00 |
| ENSG00000267041 | 125.78 | 1.13 | 0.00 | ENSG00000198300 | 63.29 | -2.67 | 0.00 |
| ENSG00000171757 | 61.96 | 1.13 | 0.00 | ENSG00000183778 | 1490.65 | -2.69 | 0.00 |
| ENSG00000174791 | 954.43 | 1.13 | 0.00 | ENSG00000101955 | 244.28 | -2.69 | 0.00 |
| ENSG00000157227 | 9880.38 | 1.13 | 0.00 | ENSG00000277586 | 53.39 | -2.69 | 0.00 |
| ENSG00000115758 | 8318.47 | 1.13 | 0.00 | ENSG00000211611 | 78.61 | -2.70 | 0.00 |
| ENSG00000185803 | 4865.63 | 1.13 | 0.00 | ENSG00000256618 | 303.19 | -2.70 | 0.00 |
| ENSG00000137168 | 1434.92 | 1.13 | 0.00 | ENSG00000100626 | 52.91 | -2.70 | 0.00 |
| ENSG00000137804 | 1734.48 | 1.13 | 0.00 | ENSG00000131471 | 1137.47 | -2.71 | 0.00 |
| ENSG00000185101 | 5059.28 | 1.13 | 0.00 | ENSG00000224650 | 850.95 | -2.71 | 0.00 |
| ENSG00000162989 | 115.30 | 1.13 | 0.00 | ENSG00000065320 | 259.54 | -2.71 | 0.00 |
| ENSG00000197261 | 190.68 | 1.13 | 0.00 | ENSG00000132514 | 138.49 | -2.71 | 0.00 |
| ENSG00000058335 | 65.59 | 1.13 | 0.00 | ENSG00000173641 | 289.49 | -2.71 | 0.00 |
| ENSG00000101220 | 2211.51 | 1.13 | 0.00 | ENSG00000105609 | 146.83 | -2.72 | 0.00 |
| ENSG00000144554 | 903.59 | 1.13 | 0.00 | ENSG00000158467 | 4232.55 | -2.72 | 0.00 |
| ENSG00000139146 | 3448.75 | 1.13 | 0.00 | ENSG00000181856 | 178.96 | -2.73 | 0.00 |
| ENSG00000186184 | 5314.12 | 1.13 | 0.00 | ENSG00000082397 | 787.96 | -2.73 | 0.00 |
| ENSG00000109089 | 670.41 | 1.13 | 0.00 | ENSG00000158887 | 59.42 | -2.73 | 0.00 |
| ENSG00000105202 | 8495.12 | 1.13 | 0.00 | ENSG00000173597 | 2775.83 | -2.73 | 0.00 |
| ENSG00000164953 | 244.83 | 1.13 | 0.00 | ENSG00000019102 | 1415.14 | -2.74 | 0.00 |
| ENSG00000156469 | 684.37 | 1.13 | 0.00 | ENSG00000006747 | 782.16 | -2.74 | 0.00 |
| ENSG00000182841 | 241.12 | 1.13 | 0.00 | ENSG00000151623 | 1031.18 | -2.74 | 0.00 |
| ENSG00000130827 | 2115.39 | 1.13 | 0.00 | ENSG00000079308 | 4297.47 | -2.75 | 0.00 |
| ENSG00000149809 | 829.15 | 1.13 | 0.00 | ENSG00000136842 | 78.11 | -2.75 | 0.00 |
| ENSG00000173621 | 1455.53 | 1.12 | 0.00 | ENSG00000132517 | 59.23 | -2.76 | 0.00 |
| ENSG00000163931 | 15982.41 | 1.12 | 0.00 | ENSG00000040199 | 889.11 | -2.76 | 0.00 |
| ENSG00000115657 | 177.32 | 1.12 | 0.00 | ENSG00000211642 | 287.18 | -2.76 | 0.00 |
| ENSG00000255737 | 258.99 | 1.12 | 0.00 | ENSG00000163145 | 58.17 | -2.76 | 0.00 |
| ENSG00000197182 | 88.58 | 1.12 | 0.00 | ENSG00000211652 | 245.15 | -2.77 | 0.00 |
| ENSG00000006634 | 746.47 | 1.12 | 0.00 | ENSG00000278196 | 604.54 | -2.77 | 0.00 |
| ENSG00000230207 | 57.27 | 1.12 | 0.00 | ENSG00000119919 | 172.77 | -2.78 | 0.00 |
| ENSG00000080608 | 1645.01 | 1.12 | 0.00 | ENSG00000171431 | 14478.11 | -2.79 | 0.00 |
| ENSG00000136872 | 1450.44 | 1.12 | 0.00 | ENSG00000118515 | 2037.31 | -2.79 | 0.00 |
| ENSG00000112759 | 3414.71 | 1.12 | 0.00 | ENSG00000138615 | 273.33 | -2.80 | 0.00 |
| ENSG00000212123 | 100.70 | 1.12 | 0.00 | ENSG00000229619 | 153.09 | -2.80 | 0.00 |
| ENSG00000242294 | 206.11 | 1.12 | 0.00 | ENSG00000156298 | 755.39 | -2.80 | 0.00 |
| ENSG00000130713 | 1147.15 | 1.12 | 0.00 | ENSG00000105737 | 54.48 | -2.80 | 0.00 |
| ENSG00000140988 | 59308.12 | 1.12 | 0.00 | ENSG00000171916 | 179.56 | -2.81 | 0.00 |
| ENSG00000163938 | 3857.99 | 1.12 | 0.00 | ENSG00000168903 | 667.92 | -2.81 | 0.00 |
| ENSG00000147799 | 848.14 | 1.12 | 0.00 | ENSG00000112276 | 94.97 | -2.81 | 0.00 |
| ENSG00000135740 | 51.80 | 1.12 | 0.00 | ENSG00000018236 | 58.07 | -2.81 | 0.00 |
| ENSG00000060558 | 449.71 | 1.12 | 0.00 | ENSG00000160712 | 633.94 | -2.81 | 0.00 |
| ENSG00000212864 | 340.34 | 1.12 | 0.00 | ENSG00000152580 | 59.49 | -2.82 | 0.00 |
| ENSG00000125977 | 5056.49 | 1.12 | 0.00 | ENSG00000163586 | 20911.93 | -2.82 | 0.00 |
| ENSG00000174567 | 439.03 | 1.12 | 0.00 | ENSG00000124253 | 2464.86 | -2.82 | 0.00 |
| ENSG00000241127 | 806.91 | 1.12 | 0.00 | ENSG00000280143 | 211.95 | -2.84 | 0.00 |
| ENSG00000173145 | 744.71 | 1.12 | 0.00 | ENSG00000064655 | 199.58 | -2.84 | 0.00 |
| ENSG00000080823 | 257.34 | 1.12 | 0.00 | ENSG00000114455 | 1639.45 | -2.84 | 0.00 |
| ENSG00000243649 | 670.83 | 1.11 | 0.00 | ENSG00000133083 | 88.77 | -2.84 | 0.00 |
| ENSG00000123843 | 333.29 | 1.11 | 0.00 | ENSG00000184434 | 1099.98 | -2.85 | 0.00 |
| ENSG00000105677 | 3427.20 | 1.11 | 0.00 | ENSG00000259459 | 53.62 | -2.85 | 0.00 |
| ENSG00000001617 | 1492.86 | 1.11 | 0.00 | ENSG00000198624 | 943.04 | -2.86 | 0.00 |
| ENSG00000107937 | 2425.74 | 1.11 | 0.00 | ENSG00000164120 | 1682.56 | -2.86 | 0.00 |
| ENSG00000280027 | 60.05 | 1.11 | 0.00 | ENSG00000223573 | 60.75 | -2.86 | 0.00 |
| ENSG00000116852 | 1212.95 | 1.11 | 0.00 | ENSG00000166391 | 1039.65 | -2.86 | 0.00 |
| ENSG00000183527 | 1710.22 | 1.11 | 0.00 | ENSG00000088386 | 241.27 | -2.88 | 0.00 |
| ENSG00000125485 | 740.92 | 1.11 | 0.00 | ENSG00000127129 | 57.37 | -2.89 | 0.00 |
| ENSG00000075914 | 1368.14 | 1.11 | 0.00 | ENSG00000095303 | 830.96 | -2.90 | 0.00 |
| ENSG00000100479 | 291.52 | 1.11 | 0.00 | ENSG00000173175 | 252.05 | -2.90 | 0.00 |
| ENSG00000186231 | 77.13 | 1.11 | 0.00 | ENSG00000167701 | 643.03 | -2.90 | 0.00 |
| ENSG00000109065 | 1260.43 | 1.11 | 0.00 | ENSG00000149591 | 11518.55 | -2.91 | 0.00 |
| ENSG00000126003 | 3330.86 | 1.11 | 0.00 | ENSG00000231292 | 76.34 | -2.91 | 0.00 |
| ENSG00000151651 | 645.71 | 1.11 | 0.00 | ENSG00000137634 | 1453.42 | -2.92 | 0.00 |
| ENSG00000196305 | 6276.59 | 1.11 | 0.00 | ENSG00000100628 | 224.88 | -2.92 | 0.00 |
| ENSG00000070814 | 3221.01 | 1.11 | 0.00 | ENSG00000072195 | 320.03 | -2.92 | 0.00 |
| ENSG00000170786 | 567.40 | 1.11 | 0.00 | ENSG00000149294 | 204.58 | -2.93 | 0.00 |
| ENSG00000100068 | 236.74 | 1.11 | 0.00 | ENSG00000198417 | 520.57 | -2.95 | 0.00 |
| ENSG00000188157 | 7820.57 | 1.11 | 0.00 | ENSG00000134201 | 56.67 | -2.95 | 0.00 |
| ENSG00000124802 | 383.19 | 1.11 | 0.00 | ENSG00000176641 | 327.72 | -2.95 | 0.00 |
| ENSG00000182325 | 1910.37 | 1.10 | 0.00 | ENSG00000158246 | 83.49 | -2.95 | 0.00 |
| ENSG00000139514 | 5379.76 | 1.10 | 0.00 | ENSG00000141574 | 1450.86 | -2.97 | 0.00 |
| ENSG00000135912 | 1199.08 | 1.10 | 0.00 | ENSG00000253958 | 922.75 | -2.97 | 0.00 |
| ENSG00000099901 | 3453.85 | 1.10 | 0.00 | ENSG00000172260 | 192.92 | -2.97 | 0.00 |
| ENSG00000125821 | 1526.79 | 1.10 | 0.00 | ENSG00000166816 | 616.95 | -2.97 | 0.00 |
| ENSG00000155100 | 576.91 | 1.10 | 0.00 | ENSG00000211937 | 105.03 | -2.97 | 0.00 |
| ENSG00000234912 | 223.71 | 1.10 | 0.00 | ENSG00000101938 | 436.46 | -2.98 | 0.00 |
| ENSG00000184786 | 82.79 | 1.10 | 0.00 | ENSG00000168497 | 370.67 | -2.99 | 0.00 |
| ENSG00000213742 | 57.64 | 1.10 | 0.00 | ENSG00000141052 | 224.13 | -2.99 | 0.00 |
| ENSG00000180822 | 747.15 | 1.10 | 0.00 | ENSG00000225698 | 336.26 | -3.01 | 0.00 |
| ENSG00000137474 | 397.08 | 1.10 | 0.00 | ENSG00000138722 | 183.15 | -3.02 | 0.00 |
| ENSG00000152455 | 490.65 | 1.10 | 0.00 | ENSG00000167723 | 76.51 | -3.03 | 0.00 |
| ENSG00000197299 | 436.87 | 1.10 | 0.00 | ENSG00000156113 | 732.26 | -3.03 | 0.00 |
| ENSG00000268218 | 50.73 | 1.10 | 0.00 | ENSG00000144218 | 68.72 | -3.03 | 0.00 |
| ENSG00000265763 | 122.24 | 1.10 | 0.00 | ENSG00000177675 | 314.26 | -3.04 | 0.00 |
| ENSG00000184162 | 970.03 | 1.10 | 0.00 | ENSG00000145287 | 2511.05 | -3.04 | 0.00 |
| ENSG00000244560 | 56.42 | 1.10 | 0.00 | ENSG00000232070 | 65.82 | -3.04 | 0.00 |
| ENSG00000176619 | 6373.39 | 1.10 | 0.00 | ENSG00000179314 | 365.74 | -3.04 | 0.00 |
| ENSG00000204060 | 51.56 | 1.10 | 0.00 | ENSG00000136457 | 58.86 | -3.05 | 0.00 |
| ENSG00000251136 | 116.14 | 1.10 | 0.00 | ENSG00000151892 | 160.81 | -3.05 | 0.00 |
| ENSG00000161800 | 2261.64 | 1.10 | 0.00 | ENSG00000187824 | 154.84 | -3.06 | 0.00 |
| ENSG00000226415 | 119.07 | 1.10 | 0.00 | ENSG00000198074 | 1000.24 | -3.07 | 0.00 |
| ENSG00000171097 | 179.58 | 1.10 | 0.00 | ENSG00000135842 | 751.20 | -3.08 | 0.00 |
| ENSG00000233822 | 58.49 | 1.10 | 0.00 | ENSG00000149596 | 271.58 | -3.09 | 0.00 |
| ENSG00000196188 | 2218.84 | 1.10 | 0.01 | ENSG00000163710 | 75.31 | -3.09 | 0.00 |
| ENSG00000118473 | 117.75 | 1.09 | 0.00 | ENSG00000170482 | 126.96 | -3.10 | 0.00 |
| ENSG00000125885 | 796.83 | 1.09 | 0.00 | ENSG00000145362 | 230.96 | -3.10 | 0.00 |
| ENSG00000176485 | 1312.82 | 1.09 | 0.00 | ENSG00000203685 | 141.19 | -3.11 | 0.00 |
| ENSG00000126226 | 2034.16 | 1.09 | 0.00 | ENSG00000166183 | 86.75 | -3.11 | 0.00 |
| ENSG00000213609 | 66.77 | 1.09 | 0.00 | ENSG00000138944 | 125.50 | -3.12 | 0.00 |
| ENSG00000143507 | 487.94 | 1.09 | 0.00 | ENSG00000154175 | 343.61 | -3.12 | 0.00 |
| ENSG00000111907 | 1122.72 | 1.09 | 0.00 | ENSG00000171227 | 883.53 | -3.13 | 0.00 |
| ENSG00000105011 | 1702.63 | 1.09 | 0.00 | ENSG00000111863 | 876.24 | -3.13 | 0.00 |
| ENSG00000049089 | 641.57 | 1.09 | 0.00 | ENSG00000204614 | 108.56 | -3.14 | 0.00 |
| ENSG00000134690 | 1612.00 | 1.09 | 0.00 | ENSG00000137872 | 393.19 | -3.15 | 0.00 |
| ENSG00000270344 | 110.04 | 1.09 | 0.00 | ENSG00000127241 | 202.05 | -3.16 | 0.00 |
| ENSG00000128944 | 717.24 | 1.09 | 0.00 | ENSG00000156218 | 125.27 | -3.17 | 0.00 |
| ENSG00000120256 | 2285.99 | 1.09 | 0.00 | ENSG00000156966 | 1891.19 | -3.17 | 0.00 |
| ENSG00000204394 | 5561.50 | 1.09 | 0.00 | ENSG00000145384 | 437.10 | -3.18 | 0.00 |
| ENSG00000143256 | 1754.58 | 1.09 | 0.00 | ENSG00000067840 | 67.17 | -3.19 | 0.00 |
| ENSG00000214050 | 67.98 | 1.09 | 0.00 | ENSG00000101335 | 10286.40 | -3.20 | 0.00 |
| ENSG00000188158 | 431.57 | 1.09 | 0.00 | ENSG00000196620 | 247.65 | -3.21 | 0.00 |
| ENSG00000054967 | 350.15 | 1.09 | 0.00 | ENSG00000171714 | 77.57 | -3.21 | 0.00 |
| ENSG00000105185 | 2416.02 | 1.09 | 0.00 | ENSG00000059915 | 325.65 | -3.21 | 0.00 |
| ENSG00000103148 | 1837.71 | 1.09 | 0.00 | ENSG00000170153 | 169.09 | -3.22 | 0.00 |
| ENSG00000186767 | 600.07 | 1.09 | 0.00 | ENSG00000181541 | 533.80 | -3.22 | 0.00 |
| ENSG00000139880 | 426.37 | 1.09 | 0.00 | ENSG00000100448 | 54.38 | -3.23 | 0.00 |
| ENSG00000113083 | 722.38 | 1.09 | 0.00 | ENSG00000145936 | 340.68 | -3.23 | 0.00 |
| ENSG00000236552 | 998.64 | 1.09 | 0.00 | ENSG00000090402 | 894.11 | -3.24 | 0.00 |
| ENSG00000168003 | 6691.34 | 1.09 | 0.00 | ENSG00000135220 | 762.97 | -3.24 | 0.00 |
| ENSG00000196436 | 122.60 | 1.09 | 0.00 | ENSG00000048462 | 85.74 | -3.24 | 0.00 |
| ENSG00000162366 | 4419.74 | 1.09 | 0.00 | ENSG00000250722 | 4110.40 | -3.25 | 0.00 |
| ENSG00000156127 | 179.67 | 1.08 | 0.00 | ENSG00000162670 | 79.11 | -3.25 | 0.00 |
| ENSG00000228288 | 70.76 | 1.08 | 0.00 | ENSG00000130055 | 162.54 | -3.26 | 0.00 |
| ENSG00000254682 | 146.25 | 1.08 | 0.00 | ENSG00000073737 | 1056.93 | -3.26 | 0.00 |
| ENSG00000188878 | 131.96 | 1.08 | 0.00 | ENSG00000188242 | 2047.02 | -3.27 | 0.00 |
| ENSG00000006625 | 2022.90 | 1.08 | 0.00 | ENSG00000101605 | 118.16 | -3.27 | 0.00 |
| ENSG00000133612 | 2993.02 | 1.08 | 0.00 | ENSG00000022267 | 1429.36 | -3.27 | 0.00 |
| ENSG00000101825 | 3165.03 | 1.08 | 0.00 | ENSG00000163687 | 177.85 | -3.28 | 0.00 |
| ENSG00000132341 | 13456.42 | 1.08 | 0.00 | ENSG00000130822 | 103.95 | -3.28 | 0.00 |
| ENSG00000229358 | 88.36 | 1.08 | 0.00 | ENSG00000213996 | 53.54 | -3.29 | 0.00 |
| ENSG00000115368 | 1594.80 | 1.08 | 0.00 | ENSG00000113303 | 619.98 | -3.29 | 0.00 |
| ENSG00000099889 | 669.77 | 1.08 | 0.00 | ENSG00000117115 | 3536.94 | -3.29 | 0.00 |
| ENSG00000166197 | 5850.33 | 1.08 | 0.00 | ENSG00000133328 | 79.64 | -3.31 | 0.00 |
| ENSG00000137198 | 418.97 | 1.08 | 0.00 | ENSG00000147883 | 931.31 | -3.32 | 0.00 |
| ENSG00000096384 | 51214.96 | 1.08 | 0.00 | ENSG00000172318 | 100.09 | -3.32 | 0.00 |
| ENSG00000106344 | 1340.39 | 1.08 | 0.00 | ENSG00000004799 | 1075.52 | -3.32 | 0.00 |
| ENSG00000119771 | 424.15 | 1.08 | 0.00 | ENSG00000154330 | 766.27 | -3.34 | 0.00 |
| ENSG00000162129 | 680.44 | 1.08 | 0.00 | ENSG00000124205 | 416.93 | -3.35 | 0.00 |
| ENSG00000188486 | 2905.55 | 1.08 | 0.00 | ENSG00000162460 | 116.75 | -3.36 | 0.00 |
| ENSG00000013810 | 2337.35 | 1.08 | 0.00 | ENSG00000065534 | 5836.89 | -3.37 | 0.00 |
| ENSG00000218426 | 505.59 | 1.08 | 0.00 | ENSG00000226306 | 50.08 | -3.37 | 0.00 |
| ENSG00000177674 | 1359.82 | 1.07 | 0.00 | ENSG00000243064 | 52.55 | -3.37 | 0.00 |
| ENSG00000164951 | 1253.26 | 1.07 | 0.00 | ENSG00000113594 | 175.04 | -3.37 | 0.00 |
| ENSG00000177426 | 3255.41 | 1.07 | 0.00 | ENSG00000125144 | 2130.17 | -3.38 | 0.00 |
| ENSG00000168393 | 1647.02 | 1.07 | 0.00 | ENSG00000121577 | 140.20 | -3.39 | 0.00 |
| ENSG00000124228 | 3634.83 | 1.07 | 0.00 | ENSG00000086696 | 1063.10 | -3.41 | 0.00 |
| ENSG00000150456 | 301.33 | 1.07 | 0.00 | ENSG00000128591 | 2867.53 | -3.41 | 0.00 |
| ENSG00000111364 | 827.71 | 1.07 | 0.00 | ENSG00000135333 | 136.94 | -3.41 | 0.00 |
| ENSG00000144161 | 501.75 | 1.07 | 0.00 | ENSG00000087258 | 229.19 | -3.41 | 0.00 |
| ENSG00000188342 | 1659.21 | 1.07 | 0.00 | ENSG00000146039 | 298.04 | -3.42 | 0.00 |
| ENSG00000226696 | 90.46 | 1.07 | 0.00 | ENSG00000166831 | 149.98 | -3.43 | 0.00 |
| ENSG00000154743 | 605.97 | 1.07 | 0.00 | ENSG00000153446 | 56.01 | -3.44 | 0.00 |
| ENSG00000070501 | 699.78 | 1.07 | 0.00 | ENSG00000168060 | 287.93 | -3.46 | 0.00 |
| ENSG00000164933 | 1198.00 | 1.07 | 0.00 | ENSG00000198523 | 416.70 | -3.47 | 0.00 |
| ENSG00000138658 | 191.93 | 1.07 | 0.00 | ENSG00000168309 | 170.67 | -3.47 | 0.00 |
| ENSG00000187498 | 12503.34 | 1.07 | 0.00 | ENSG00000163431 | 1496.90 | -3.51 | 0.00 |
| ENSG00000135976 | 95.01 | 1.07 | 0.00 | ENSG00000104435 | 147.83 | -3.52 | 0.00 |
| ENSG00000225978 | 65.34 | 1.07 | 0.00 | ENSG00000146469 | 302.28 | -3.52 | 0.00 |
| ENSG00000141497 | 134.88 | 1.07 | 0.00 | ENSG00000004776 | 567.86 | -3.52 | 0.00 |
| ENSG00000204271 | 122.49 | 1.07 | 0.00 | ENSG00000169432 | 101.40 | -3.53 | 0.00 |
| ENSG00000135632 | 1430.46 | 1.07 | 0.00 | ENSG00000048540 | 164.93 | -3.54 | 0.00 |
| ENSG00000102981 | 291.87 | 1.07 | 0.00 | ENSG00000154165 | 65.05 | -3.55 | 0.00 |
| ENSG00000254154 | 53.49 | 1.07 | 0.00 | ENSG00000132915 | 66.23 | -3.56 | 0.00 |
| ENSG00000103811 | 4872.66 | 1.06 | 0.00 | ENSG00000181072 | 81.31 | -3.57 | 0.00 |
| ENSG00000115598 | 91.19 | 1.06 | 0.00 | ENSG00000129167 | 66.67 | -3.57 | 0.00 |
| ENSG00000140678 | 577.60 | 1.06 | 0.00 | ENSG00000211890 | 40786.69 | -3.59 | 0.00 |
| ENSG00000277632 | 182.06 | 1.06 | 0.00 | ENSG00000134028 | 1603.12 | -3.59 | 0.00 |
| ENSG00000136271 | 3285.39 | 1.06 | 0.00 | ENSG00000152137 | 557.95 | -3.60 | 0.00 |
| ENSG00000074696 | 4681.16 | 1.06 | 0.00 | ENSG00000064205 | 71.56 | -3.62 | 0.00 |
| ENSG00000197385 | 63.65 | 1.06 | 0.00 | ENSG00000112936 | 645.48 | -3.66 | 0.00 |
| ENSG00000225177 | 50.58 | 1.06 | 0.00 | ENSG00000173237 | 210.00 | -3.66 | 0.00 |
| ENSG00000165271 | 2624.66 | 1.06 | 0.00 | ENSG00000259823 | 2408.65 | -3.67 | 0.00 |
| ENSG00000177542 | 1532.04 | 1.06 | 0.00 | ENSG00000187699 | 608.41 | -3.67 | 0.00 |
| ENSG00000164284 | 874.71 | 1.06 | 0.00 | ENSG00000183287 | 77.14 | -3.70 | 0.00 |
| ENSG00000224877 | 760.45 | 1.06 | 0.00 | ENSG00000242515 | 286.17 | -3.70 | 0.00 |
| ENSG00000112118 | 5746.67 | 1.06 | 0.00 | ENSG00000075073 | 267.15 | -3.73 | 0.00 |
| ENSG00000165272 | 1220.57 | 1.06 | 0.00 | ENSG00000175084 | 11953.50 | -3.73 | 0.00 |
| ENSG00000173272 | 1728.50 | 1.06 | 0.00 | ENSG00000039987 | 204.62 | -3.75 | 0.00 |
| ENSG00000165802 | 3645.19 | 1.06 | 0.00 | ENSG00000172403 | 2893.44 | -3.75 | 0.00 |
| ENSG00000165244 | 496.76 | 1.06 | 0.00 | ENSG00000170382 | 230.77 | -3.77 | 0.00 |
| ENSG00000103855 | 3140.44 | 1.06 | 0.00 | ENSG00000148483 | 600.00 | -3.77 | 0.00 |
| ENSG00000261556 | 77.81 | 1.06 | 0.00 | ENSG00000132465 | 23652.11 | -3.78 | 0.00 |
| ENSG00000184465 | 478.28 | 1.06 | 0.00 | ENSG00000111846 | 129.77 | -3.79 | 0.00 |
| ENSG00000185090 | 780.42 | 1.06 | 0.00 | ENSG00000180875 | 262.69 | -3.80 | 0.00 |
| ENSG00000261123 | 310.39 | 1.06 | 0.00 | ENSG00000166825 | 10146.07 | -3.81 | 0.00 |
| ENSG00000196236 | 771.09 | 1.06 | 0.00 | ENSG00000243238 | 57.06 | -3.82 | 0.00 |
| ENSG00000102898 | 4517.98 | 1.06 | 0.00 | ENSG00000068976 | 79.39 | -3.85 | 0.00 |
| ENSG00000180198 | 3023.69 | 1.06 | 0.00 | ENSG00000166292 | 57.16 | -3.85 | 0.00 |
| ENSG00000103599 | 55.36 | 1.06 | 0.00 | ENSG00000205358 | 279.93 | -3.87 | 0.00 |
| ENSG00000183207 | 5193.78 | 1.06 | 0.00 | ENSG00000066230 | 2035.50 | -3.88 | 0.00 |
| ENSG00000153944 | 3207.76 | 1.06 | 0.00 | ENSG00000164107 | 149.23 | -3.89 | 0.00 |
| ENSG00000181610 | 1866.39 | 1.06 | 0.00 | ENSG00000197165 | 109.70 | -3.89 | 0.00 |
| ENSG00000136492 | 360.34 | 1.06 | 0.00 | ENSG00000012504 | 92.61 | -3.89 | 0.00 |
| ENSG00000115946 | 1144.82 | 1.06 | 0.00 | ENSG00000135549 | 735.27 | -3.91 | 0.00 |
| ENSG00000101000 | 1452.18 | 1.06 | 0.00 | ENSG00000065325 | 62.65 | -3.91 | 0.00 |
| ENSG00000255182 | 68.32 | 1.06 | 0.00 | ENSG00000143196 | 576.38 | -3.91 | 0.00 |
| ENSG00000242193 | 1777.50 | 1.06 | 0.00 | ENSG00000122756 | 109.75 | -3.92 | 0.00 |
| ENSG00000103485 | 2776.66 | 1.06 | 0.00 | ENSG00000113196 | 76.87 | -3.93 | 0.00 |
| ENSG00000015475 | 2076.70 | 1.06 | 0.00 | ENSG00000106809 | 209.67 | -3.95 | 0.00 |
| ENSG00000169684 | 156.04 | 1.06 | 0.00 | ENSG00000168477 | 571.44 | -3.96 | 0.00 |
| ENSG00000196912 | 54.79 | 1.05 | 0.00 | ENSG00000116194 | 125.08 | -3.98 | 0.00 |
| ENSG00000109610 | 3814.63 | 1.05 | 0.00 | ENSG00000109182 | 217.22 | -4.00 | 0.00 |
| ENSG00000259933 | 390.32 | 1.05 | 0.00 | ENSG00000211938 | 55.79 | -4.03 | 0.00 |
| ENSG00000088727 | 389.73 | 1.05 | 0.00 | ENSG00000126950 | 97.25 | -4.04 | 0.00 |
| ENSG00000157456 | 1553.85 | 1.05 | 0.00 | ENSG00000166869 | 1805.78 | -4.04 | 0.00 |
| ENSG00000111602 | 1800.88 | 1.05 | 0.00 | ENSG00000165966 | 69.90 | -4.07 | 0.00 |
| ENSG00000142149 | 1233.76 | 1.05 | 0.00 | ENSG00000122367 | 110.56 | -4.08 | 0.00 |
| ENSG00000204860 | 131.21 | 1.05 | 0.00 | ENSG00000197888 | 2849.31 | -4.09 | 0.00 |
| ENSG00000244486 | 343.80 | 1.05 | 0.00 | ENSG00000248144 | 2092.50 | -4.09 | 0.00 |
| ENSG00000178105 | 1203.66 | 1.05 | 0.00 | ENSG00000036672 | 202.22 | -4.09 | 0.00 |
| ENSG00000119285 | 1757.95 | 1.05 | 0.00 | ENSG00000162461 | 94.14 | -4.10 | 0.00 |
| ENSG00000166226 | 7958.66 | 1.05 | 0.00 | ENSG00000114200 | 66.48 | -4.13 | 0.00 |
| ENSG00000136144 | 925.01 | 1.05 | 0.00 | ENSG00000138669 | 73.68 | -4.13 | 0.00 |
| ENSG00000119403 | 1811.30 | 1.05 | 0.00 | ENSG00000163017 | 10230.94 | -4.16 | 0.00 |
| ENSG00000160131 | 1685.46 | 1.05 | 0.00 | ENSG00000130176 | 3888.72 | -4.16 | 0.00 |
| ENSG00000198169 | 584.10 | 1.05 | 0.00 | ENSG00000186198 | 385.52 | -4.17 | 0.00 |
| ENSG00000102837 | 67262.51 | 1.05 | 0.01 | ENSG00000133800 | 231.01 | -4.18 | 0.00 |
| ENSG00000117335 | 16340.01 | 1.05 | 0.00 | ENSG00000168447 | 595.94 | -4.19 | 0.00 |
| ENSG00000065150 | 8577.96 | 1.05 | 0.00 | ENSG00000104332 | 248.67 | -4.21 | 0.00 |
| ENSG00000196208 | 55.95 | 1.05 | 0.00 | ENSG00000163815 | 202.49 | -4.21 | 0.00 |
| ENSG00000165138 | 1545.03 | 1.05 | 0.00 | ENSG00000150625 | 79.55 | -4.21 | 0.00 |
| ENSG00000145604 | 1177.92 | 1.05 | 0.00 | ENSG00000167080 | 910.91 | -4.24 | 0.00 |
| ENSG00000198546 | 1055.23 | 1.05 | 0.00 | ENSG00000155850 | 9025.37 | -4.28 | 0.00 |
| ENSG00000151503 | 1725.64 | 1.05 | 0.00 | ENSG00000205364 | 304.36 | -4.28 | 0.00 |
| ENSG00000132635 | 917.94 | 1.05 | 0.00 | ENSG00000182253 | 1988.73 | -4.31 | 0.00 |
| ENSG00000086289 | 1124.41 | 1.05 | 0.00 | ENSG00000119121 | 595.22 | -4.32 | 0.00 |
| ENSG00000135372 | 3210.71 | 1.05 | 0.00 | ENSG00000242366 | 75.39 | -4.33 | 0.00 |
| ENSG00000176978 | 3724.70 | 1.05 | 0.00 | ENSG00000118729 | 145.32 | -4.34 | 0.00 |
| ENSG00000101391 | 1325.09 | 1.04 | 0.00 | ENSG00000163959 | 347.98 | -4.35 | 0.00 |
| ENSG00000116337 | 1931.19 | 1.04 | 0.00 | ENSG00000196616 | 576.26 | -4.36 | 0.00 |
| ENSG00000187109 | 11032.95 | 1.04 | 0.00 | ENSG00000007306 | 14551.24 | -4.36 | 0.00 |
| ENSG00000171984 | 335.24 | 1.04 | 0.00 | ENSG00000164530 | 110.56 | -4.42 | 0.00 |
| ENSG00000280987 | 79.19 | 1.04 | 0.00 | ENSG00000166828 | 53.83 | -4.43 | 0.00 |
| ENSG00000115687 | 644.08 | 1.04 | 0.00 | ENSG00000163295 | 232.84 | -4.44 | 0.00 |
| ENSG00000111788 | 129.01 | 1.04 | 0.00 | ENSG00000133392 | 22462.61 | -4.45 | 0.00 |
| ENSG00000197774 | 482.15 | 1.04 | 0.00 | ENSG00000187783 | 109.26 | -4.46 | 0.00 |
| ENSG00000262714 | 81.64 | 1.04 | 0.00 | ENSG00000060566 | 144.99 | -4.50 | 0.00 |
| ENSG00000161016 | 102136.86 | 1.04 | 0.00 | ENSG00000175785 | 93.90 | -4.52 | 0.00 |
| ENSG00000133216 | 9343.44 | 1.04 | 0.00 | ENSG00000237125 | 79.54 | -4.52 | 0.00 |
| ENSG00000235065 | 64.12 | 1.04 | 0.00 | ENSG00000162706 | 105.79 | -4.53 | 0.00 |
| ENSG00000205763 | 172.92 | 1.04 | 0.00 | ENSG00000141338 | 166.51 | -4.55 | 0.00 |
| ENSG00000176153 | 22615.37 | 1.04 | 0.00 | ENSG00000080493 | 1270.12 | -4.57 | 0.00 |
| ENSG00000181163 | 21552.88 | 1.04 | 0.00 | ENSG00000100604 | 1236.23 | -4.58 | 0.00 |
| ENSG00000162383 | 252.91 | 1.04 | 0.00 | ENSG00000174992 | 5822.40 | -4.59 | 0.00 |
| ENSG00000213918 | 906.76 | 1.04 | 0.00 | ENSG00000269404 | 258.07 | -4.61 | 0.00 |
| ENSG00000138363 | 4904.57 | 1.04 | 0.00 | ENSG00000091138 | 16968.71 | -4.69 | 0.00 |
| ENSG00000070950 | 858.82 | 1.04 | 0.00 | ENSG00000165072 | 117.34 | -4.72 | 0.00 |
| ENSG00000149527 | 211.05 | 1.04 | 0.00 | ENSG00000104267 | 5911.70 | -4.74 | 0.00 |
| ENSG00000186481 | 56.26 | 1.04 | 0.00 | ENSG00000168079 | 392.18 | -4.78 | 0.00 |
| ENSG00000163995 | 846.61 | 1.04 | 0.00 | ENSG00000018625 | 191.61 | -4.81 | 0.00 |
| ENSG00000138442 | 1655.14 | 1.04 | 0.00 | ENSG00000136546 | 114.98 | -4.81 | 0.00 |
| ENSG00000275004 | 89.96 | 1.04 | 0.00 | ENSG00000171246 | 122.89 | -4.81 | 0.00 |
| ENSG00000225131 | 93.85 | 1.04 | 0.00 | ENSG00000118777 | 357.39 | -4.84 | 0.00 |
| ENSG00000173239 | 94.48 | 1.04 | 0.00 | ENSG00000167281 | 85.22 | -4.84 | 0.00 |
| ENSG00000243753 | 892.80 | 1.04 | 0.00 | ENSG00000249948 | 129.75 | -4.90 | 0.00 |
| ENSG00000086712 | 1328.73 | 1.04 | 0.00 | ENSG00000157005 | 65.52 | -4.94 | 0.00 |
| ENSG00000089195 | 927.84 | 1.04 | 0.00 | ENSG00000079689 | 66.49 | -4.97 | 0.00 |
| ENSG00000097046 | 342.84 | 1.04 | 0.00 | ENSG00000240498 | 183.84 | -5.02 | 0.00 |
| ENSG00000152749 | 777.36 | 1.04 | 0.00 | ENSG00000167434 | 1859.32 | -5.06 | 0.00 |
| ENSG00000184216 | 8600.92 | 1.04 | 0.00 | ENSG00000115263 | 197.36 | -5.19 | 0.00 |
| ENSG00000188312 | 203.40 | 1.04 | 0.00 | ENSG00000197273 | 2281.87 | -5.26 | 0.00 |
| ENSG00000138031 | 1670.70 | 1.04 | 0.00 | ENSG00000123560 | 107.10 | -5.28 | 0.00 |
| ENSG00000102977 | 920.95 | 1.04 | 0.00 | ENSG00000152785 | 254.80 | -5.29 | 0.00 |
| ENSG00000168918 | 2250.82 | 1.03 | 0.00 | ENSG00000174358 | 367.82 | -5.36 | 0.00 |
| ENSG00000247077 | 3434.49 | 1.03 | 0.00 | ENSG00000016602 | 4595.19 | -5.38 | 0.00 |
| ENSG00000073756 | 557.62 | 1.03 | 0.00 | ENSG00000071203 | 1536.54 | -5.43 | 0.00 |
| ENSG00000138035 | 1642.45 | 1.03 | 0.00 | ENSG00000141434 | 175.52 | -5.63 | 0.00 |
| ENSG00000100196 | 1388.62 | 1.03 | 0.00 | ENSG00000204936 | 2490.35 | -5.65 | 0.00 |
| ENSG00000105865 | 304.45 | 1.03 | 0.00 | ENSG00000156284 | 189.71 | -5.66 | 0.00 |
| ENSG00000105855 | 780.30 | 1.03 | 0.00 | ENSG00000203859 | 91.50 | -5.69 | 0.00 |
| ENSG00000132382 | 2919.02 | 1.03 | 0.00 | ENSG00000168748 | 402.41 | -5.72 | 0.00 |
| ENSG00000116857 | 3326.97 | 1.03 | 0.00 | ENSG00000131096 | 304.53 | -5.76 | 0.00 |
| ENSG00000100350 | 906.36 | 1.03 | 0.00 | ENSG00000142959 | 311.52 | -5.89 | 0.00 |
| ENSG00000136877 | 2594.69 | 1.03 | 0.00 | ENSG00000196660 | 82.33 | -5.90 | 0.00 |
| ENSG00000166803 | 922.16 | 1.03 | 0.00 | ENSG00000172410 | 100.20 | -6.12 | 0.00 |
| ENSG00000171604 | 2894.28 | 1.03 | 0.00 | ENSG00000044012 | 420.96 | -6.22 | 0.00 |
| ENSG00000244313 | 512.23 | 1.03 | 0.00 | ENSG00000182271 | 434.40 | -6.31 | 0.00 |
| ENSG00000198796 | 51.98 | 1.03 | 0.00 | ENSG00000133742 | 3424.14 | -6.78 | 0.00 |
| ENSG00000101181 | 1662.66 | 1.03 | 0.00 | ENSG00000103375 | 4000.91 | -7.13 | 0.00 |
| ENSG00000172731 | 709.57 | 1.03 | 0.00 | ENSG00000183034 | 315.86 | -8.15 | 0.00 |
| ENSG00000124588 | 1022.33 | 1.03 | 0.00 |  |  |  |  |

**Supplementary table 4 Differentially expressed mRNAs for READ**

| Tag | baseMean | log2FoldChange | padj | Tag | baseMean | log2FoldChange | padj |
| --- | --- | --- | --- | --- | --- | --- | --- |
| ENSG00000221867 | 208.41 | 11.69 | 0.00 | ENSG00000164885 | 797.23 | 1.04 | 0.00 |
| ENSG00000204542 | 169.12 | 10.11 | 0.00 | ENSG00000128917 | 990.21 | 1.04 | 0.00 |
| ENSG00000172016 | 3529.34 | 9.64 | 0.00 | ENSG00000168394 | 7071.40 | 1.04 | 0.00 |
| ENSG00000276122 | 108.98 | 9.47 | 0.00 | ENSG00000228989 | 167.38 | 1.04 | 0.00 |
| ENSG00000213401 | 83.08 | 9.31 | 0.00 | ENSG00000100416 | 1127.60 | 1.04 | 0.00 |
| ENSG00000129455 | 87.75 | 9.16 | 0.00 | ENSG00000213563 | 1762.52 | 1.04 | 0.01 |
| ENSG00000197172 | 158.60 | 9.07 | 0.00 | ENSG00000115598 | 81.11 | 1.04 | 0.03 |
| ENSG00000115386 | 8839.79 | 9.06 | 0.00 | ENSG00000163472 | 284.24 | 1.03 | 0.00 |
| ENSG00000168875 | 63.71 | 8.70 | 0.00 | ENSG00000104738 | 5152.14 | 1.03 | 0.00 |
| ENSG00000128610 | 94.27 | 8.69 | 0.00 | ENSG00000125991 | 16809.28 | 1.03 | 0.00 |
| ENSG00000167755 | 868.83 | 8.65 | 0.00 | ENSG00000174938 | 1989.19 | 1.03 | 0.00 |
| ENSG00000237412 | 99.53 | 8.48 | 0.00 | ENSG00000067955 | 2220.27 | 1.03 | 0.00 |
| ENSG00000169035 | 137.07 | 8.15 | 0.00 | ENSG00000186184 | 5963.61 | 1.03 | 0.00 |
| ENSG00000139800 | 71.31 | 7.99 | 0.00 | ENSG00000153395 | 2199.44 | 1.03 | 0.00 |
| ENSG00000100665 | 53.66 | 7.87 | 0.00 | ENSG00000186056 | 59.44 | 1.03 | 0.00 |
| ENSG00000108244 | 3718.83 | 7.81 | 0.00 | ENSG00000112787 | 3634.94 | 1.03 | 0.00 |
| ENSG00000170373 | 925.80 | 7.79 | 0.00 | ENSG00000055044 | 3482.04 | 1.03 | 0.00 |
| ENSG00000185269 | 3804.28 | 7.79 | 0.00 | ENSG00000263272 | 96.36 | 1.03 | 0.00 |
| ENSG00000224099 | 160.00 | 7.70 | 0.00 | ENSG00000181649 | 1837.28 | 1.03 | 0.01 |
| ENSG00000015413 | 11682.87 | 7.56 | 0.00 | ENSG00000180817 | 11853.73 | 1.03 | 0.00 |
| ENSG00000170454 | 50.23 | 7.52 | 0.00 | ENSG00000075643 | 542.94 | 1.03 | 0.00 |
| ENSG00000123500 | 880.77 | 7.51 | 0.00 | ENSG00000160972 | 2317.18 | 1.03 | 0.01 |
| ENSG00000162344 | 181.47 | 7.45 | 0.00 | ENSG00000176125 | 99.95 | 1.03 | 0.00 |
| ENSG00000230316 | 163.94 | 7.38 | 0.00 | ENSG00000145545 | 993.31 | 1.03 | 0.00 |
| ENSG00000164379 | 1906.80 | 7.32 | 0.00 | ENSG00000100288 | 115.83 | 1.03 | 0.00 |
| ENSG00000249550 | 154.72 | 7.24 | 0.00 | ENSG00000104951 | 284.09 | 1.03 | 0.02 |
| ENSG00000198610 | 61.83 | 7.23 | 0.00 | ENSG00000132879 | 480.45 | 1.03 | 0.00 |
| ENSG00000275896 | 252.07 | 7.09 | 0.00 | ENSG00000086712 | 1499.93 | 1.03 | 0.00 |
| ENSG00000170835 | 1511.96 | 7.09 | 0.00 | ENSG00000280027 | 64.50 | 1.03 | 0.02 |
| ENSG00000259485 | 70.01 | 7.04 | 0.00 | ENSG00000278619 | 515.88 | 1.03 | 0.00 |
| ENSG00000104371 | 90.30 | 7.04 | 0.00 | ENSG00000130713 | 1237.51 | 1.03 | 0.00 |
| ENSG00000134827 | 754.87 | 6.98 | 0.00 | ENSG00000107819 | 1773.06 | 1.03 | 0.00 |
| ENSG00000111700 | 124.02 | 6.97 | 0.00 | ENSG00000129810 | 296.70 | 1.03 | 0.00 |
| ENSG00000198842 | 2298.09 | 6.88 | 0.00 | ENSG00000109674 | 163.63 | 1.03 | 0.00 |
| ENSG00000231826 | 386.35 | 6.86 | 0.00 | ENSG00000074071 | 4233.42 | 1.03 | 0.00 |
| ENSG00000185247 | 97.08 | 6.84 | 0.00 | ENSG00000054967 | 350.54 | 1.03 | 0.00 |
| ENSG00000251164 | 81.62 | 6.84 | 0.00 | ENSG00000166012 | 2474.04 | 1.03 | 0.00 |
| ENSG00000214039 | 734.18 | 6.73 | 0.00 | ENSG00000263266 | 430.89 | 1.03 | 0.02 |
| ENSG00000137673 | 2135.37 | 6.65 | 0.00 | ENSG00000141076 | 2372.22 | 1.03 | 0.00 |
| ENSG00000249306 | 104.57 | 6.57 | 0.00 | ENSG00000160766 | 83.35 | 1.03 | 0.00 |
| ENSG00000165376 | 4458.57 | 6.55 | 0.00 | ENSG00000187498 | 14532.88 | 1.03 | 0.00 |
| ENSG00000170231 | 334.32 | 6.49 | 0.00 | ENSG00000108515 | 155.36 | 1.03 | 0.00 |
| ENSG00000186007 | 147.70 | 6.45 | 0.00 | ENSG00000185298 | 1198.59 | 1.02 | 0.00 |
| ENSG00000228705 | 50.45 | 6.43 | 0.00 | ENSG00000143630 | 420.42 | 1.02 | 0.00 |
| ENSG00000170369 | 79.19 | 6.42 | 0.00 | ENSG00000014164 | 1546.65 | 1.02 | 0.00 |
| ENSG00000205420 | 79.64 | 6.32 | 0.00 | ENSG00000141873 | 971.29 | 1.02 | 0.00 |
| ENSG00000233532 | 74.52 | 6.29 | 0.00 | ENSG00000143256 | 1809.16 | 1.02 | 0.00 |
| ENSG00000228742 | 64.75 | 6.27 | 0.00 | ENSG00000215769 | 118.72 | 1.02 | 0.00 |
| ENSG00000167767 | 1490.95 | 6.26 | 0.00 | ENSG00000231113 | 66.47 | 1.02 | 0.00 |
| ENSG00000178773 | 1298.94 | 6.18 | 0.00 | ENSG00000005448 | 413.39 | 1.02 | 0.00 |
| ENSG00000043355 | 207.91 | 6.12 | 0.00 | ENSG00000186166 | 451.59 | 1.02 | 0.00 |
| ENSG00000135374 | 125.81 | 6.11 | 0.00 | ENSG00000027847 | 1416.39 | 1.02 | 0.00 |
| ENSG00000159217 | 277.57 | 6.09 | 0.00 | ENSG00000271971 | 52.24 | 1.02 | 0.01 |
| ENSG00000164778 | 57.21 | 6.08 | 0.00 | ENSG00000130758 | 547.72 | 1.02 | 0.00 |
| ENSG00000185479 | 334.14 | 6.07 | 0.00 | ENSG00000149150 | 1381.35 | 1.02 | 0.00 |
| ENSG00000187258 | 178.25 | 6.02 | 0.00 | ENSG00000181104 | 1429.11 | 1.02 | 0.00 |
| ENSG00000196260 | 110.47 | 6.00 | 0.00 | ENSG00000164880 | 7210.85 | 1.02 | 0.00 |
| ENSG00000137868 | 601.30 | 5.99 | 0.00 | ENSG00000015475 | 2278.28 | 1.02 | 0.00 |
| ENSG00000105664 | 832.24 | 5.94 | 0.00 | ENSG00000112118 | 6042.99 | 1.02 | 0.00 |
| ENSG00000213468 | 53.93 | 5.91 | 0.00 | ENSG00000113721 | 3547.61 | 1.02 | 0.01 |
| ENSG00000105464 | 1455.02 | 5.89 | 0.00 | ENSG00000178685 | 2735.94 | 1.02 | 0.00 |
| ENSG00000172023 | 522.48 | 5.78 | 0.00 | ENSG00000189091 | 8476.19 | 1.02 | 0.00 |
| ENSG00000134757 | 643.10 | 5.69 | 0.00 | ENSG00000056998 | 827.12 | 1.02 | 0.01 |
| ENSG00000239605 | 54.54 | 5.66 | 0.00 | ENSG00000119285 | 1916.62 | 1.02 | 0.00 |
| ENSG00000171564 | 54.08 | 5.66 | 0.00 | ENSG00000133985 | 263.63 | 1.02 | 0.03 |
| ENSG00000101115 | 153.64 | 5.65 | 0.00 | ENSG00000161036 | 982.35 | 1.02 | 0.00 |
| ENSG00000103355 | 361.91 | 5.65 | 0.00 | ENSG00000163482 | 783.32 | 1.02 | 0.00 |
| ENSG00000253929 | 81.71 | 5.65 | 0.00 | ENSG00000170684 | 272.99 | 1.02 | 0.00 |
| ENSG00000182798 | 100.17 | 5.63 | 0.00 | ENSG00000110057 | 2697.02 | 1.02 | 0.00 |
| ENSG00000184292 | 1976.18 | 5.56 | 0.00 | ENSG00000180198 | 2970.83 | 1.02 | 0.00 |
| ENSG00000183844 | 646.52 | 5.52 | 0.00 | ENSG00000143786 | 122.75 | 1.02 | 0.01 |
| ENSG00000164822 | 586.20 | 5.50 | 0.00 | ENSG00000197363 | 451.95 | 1.01 | 0.00 |
| ENSG00000060718 | 1259.04 | 5.48 | 0.00 | ENSG00000103855 | 3327.31 | 1.01 | 0.00 |
| ENSG00000229404 | 55.59 | 5.44 | 0.00 | ENSG00000183527 | 1802.87 | 1.01 | 0.00 |
| ENSG00000281406 | 218.52 | 5.40 | 0.00 | ENSG00000164610 | 376.25 | 1.01 | 0.00 |
| ENSG00000115507 | 94.38 | 5.40 | 0.00 | ENSG00000142207 | 1956.10 | 1.01 | 0.00 |
| ENSG00000105989 | 353.39 | 5.38 | 0.00 | ENSG00000141552 | 3985.84 | 1.01 | 0.01 |
| ENSG00000171759 | 117.37 | 5.34 | 0.00 | ENSG00000171208 | 747.29 | 1.01 | 0.04 |
| ENSG00000234985 | 84.97 | 5.33 | 0.00 | ENSG00000007392 | 1234.66 | 1.01 | 0.00 |
| ENSG00000250641 | 488.00 | 5.27 | 0.00 | ENSG00000103249 | 2584.96 | 1.01 | 0.00 |
| ENSG00000128422 | 857.26 | 5.27 | 0.00 | ENSG00000198901 | 2103.03 | 1.01 | 0.00 |
| ENSG00000149968 | 1927.44 | 5.25 | 0.00 | ENSG00000130684 | 95.12 | 1.01 | 0.00 |
| ENSG00000204869 | 82.01 | 5.25 | 0.00 | ENSG00000204713 | 3198.18 | 1.01 | 0.00 |
| ENSG00000196611 | 3792.64 | 5.17 | 0.00 | ENSG00000162836 | 961.78 | 1.01 | 0.00 |
| ENSG00000185105 | 112.26 | 5.16 | 0.00 | ENSG00000136943 | 698.56 | 1.01 | 0.02 |
| ENSG00000184937 | 86.44 | 5.13 | 0.00 | ENSG00000189325 | 1061.95 | 1.01 | 0.00 |
| ENSG00000175832 | 4571.79 | 5.12 | 0.00 | ENSG00000213339 | 2495.59 | 1.01 | 0.00 |
| ENSG00000212993 | 340.46 | 5.12 | 0.00 | ENSG00000213462 | 357.35 | 1.01 | 0.01 |
| ENSG00000168065 | 125.34 | 5.11 | 0.00 | ENSG00000146830 | 2450.30 | 1.01 | 0.00 |
| ENSG00000105219 | 142.64 | 5.11 | 0.00 | ENSG00000102898 | 4838.07 | 1.01 | 0.00 |
| ENSG00000062038 | 3117.64 | 5.08 | 0.00 | ENSG00000242071 | 154.21 | 1.01 | 0.01 |
| ENSG00000186832 | 50.29 | 5.07 | 0.00 | ENSG00000179886 | 720.53 | 1.01 | 0.00 |
| ENSG00000103888 | 5051.30 | 5.07 | 0.00 | ENSG00000135722 | 340.67 | 1.01 | 0.00 |
| ENSG00000107807 | 125.18 | 5.05 | 0.00 | ENSG00000101138 | 1804.08 | 1.01 | 0.00 |
| ENSG00000225616 | 118.45 | 5.02 | 0.00 | ENSG00000204860 | 117.49 | 1.01 | 0.04 |
| ENSG00000214049 | 1761.47 | 5.02 | 0.00 | ENSG00000160211 | 2234.61 | 1.01 | 0.00 |
| ENSG00000244355 | 477.54 | 4.97 | 0.00 | ENSG00000173457 | 2819.62 | 1.01 | 0.00 |
| ENSG00000230061 | 159.51 | 4.96 | 0.00 | ENSG00000099364 | 1205.02 | 1.01 | 0.00 |
| ENSG00000106536 | 108.83 | 4.96 | 0.00 | ENSG00000134690 | 1636.81 | 1.01 | 0.00 |
| ENSG00000182968 | 107.12 | 4.95 | 0.00 | ENSG00000101150 | 6712.75 | 1.00 | 0.00 |
| ENSG00000213981 | 63.65 | 4.93 | 0.00 | ENSG00000188735 | 552.55 | 1.00 | 0.00 |
| ENSG00000163736 | 109.46 | 4.93 | 0.00 | ENSG00000179832 | 3082.72 | 1.00 | 0.00 |
| ENSG00000164283 | 271.68 | 4.90 | 0.00 | ENSG00000109501 | 3393.36 | 1.00 | 0.02 |
| ENSG00000164076 | 66.29 | 4.89 | 0.00 | ENSG00000102119 | 2961.67 | 1.00 | 0.00 |
| ENSG00000206069 | 185.17 | 4.89 | 0.00 | ENSG00000105364 | 3391.59 | 1.00 | 0.00 |
| ENSG00000164816 | 410.04 | 4.87 | 0.00 | ENSG00000130309 | 5693.39 | 1.00 | 0.00 |
| ENSG00000099953 | 4729.83 | 4.83 | 0.00 | ENSG00000006625 | 2037.15 | 1.00 | 0.00 |
| ENSG00000240668 | 449.04 | 4.82 | 0.00 | ENSG00000016391 | 2789.64 | 1.00 | 0.00 |
| ENSG00000107159 | 1263.59 | 4.82 | 0.00 | ENSG00000166965 | 794.24 | 1.00 | 0.00 |
| ENSG00000132746 | 100.77 | 4.81 | 0.00 | ENSG00000168393 | 1795.91 | 1.00 | 0.00 |
| ENSG00000259439 | 51.04 | 4.79 | 0.00 | ENSG00000158042 | 2243.84 | 1.00 | 0.00 |
| ENSG00000233017 | 51.67 | 4.77 | 0.00 | ENSG00000120055 | 71.98 | 1.00 | 0.00 |
| ENSG00000163347 | 3587.59 | 4.77 | 0.00 | ENSG00000233834 | 102.79 | 1.00 | 0.00 |
| ENSG00000100593 | 141.32 | 4.77 | 0.00 | ENSG00000267519 | 886.15 | 1.00 | 0.01 |
| ENSG00000129991 | 59.90 | 4.77 | 0.00 | ENSG00000260401 | 189.22 | -1.00 | 0.00 |
| ENSG00000095752 | 169.30 | 4.77 | 0.00 | ENSG00000166262 | 58.76 | -1.00 | 0.00 |
| ENSG00000248810 | 77.63 | 4.76 | 0.00 | ENSG00000151470 | 502.47 | -1.00 | 0.00 |
| ENSG00000226476 | 72.77 | 4.75 | 0.00 | ENSG00000162437 | 1478.13 | -1.00 | 0.00 |
| ENSG00000140835 | 80.19 | 4.75 | 0.00 | ENSG00000198879 | 141.90 | -1.00 | 0.01 |
| ENSG00000177238 | 93.87 | 4.74 | 0.00 | ENSG00000158985 | 3468.87 | -1.00 | 0.00 |
| ENSG00000170827 | 259.79 | 4.74 | 0.00 | ENSG00000143119 | 816.82 | -1.00 | 0.02 |
| ENSG00000129451 | 1261.12 | 4.74 | 0.00 | ENSG00000169372 | 288.02 | -1.00 | 0.00 |
| ENSG00000112761 | 131.61 | 4.71 | 0.00 | ENSG00000176273 | 367.47 | -1.01 | 0.00 |
| ENSG00000088992 | 2140.50 | 4.68 | 0.00 | ENSG00000100592 | 854.84 | -1.01 | 0.00 |
| ENSG00000164093 | 469.70 | 4.63 | 0.00 | ENSG00000213639 | 7341.90 | -1.01 | 0.00 |
| ENSG00000183734 | 6113.53 | 4.63 | 0.00 | ENSG00000015285 | 231.75 | -1.01 | 0.00 |
| ENSG00000134762 | 637.06 | 4.63 | 0.00 | ENSG00000107242 | 1758.72 | -1.01 | 0.00 |
| ENSG00000158055 | 238.75 | 4.62 | 0.00 | ENSG00000169744 | 237.11 | -1.01 | 0.00 |
| ENSG00000145506 | 1298.09 | 4.61 | 0.00 | ENSG00000113595 | 234.32 | -1.01 | 0.00 |
| ENSG00000159263 | 647.22 | 4.60 | 0.00 | ENSG00000013619 | 90.44 | -1.01 | 0.02 |
| ENSG00000162009 | 67.18 | 4.60 | 0.00 | ENSG00000143878 | 8204.11 | -1.01 | 0.00 |
| ENSG00000100078 | 59.28 | 4.59 | 0.00 | ENSG00000075426 | 5127.43 | -1.01 | 0.00 |
| ENSG00000182379 | 127.30 | 4.58 | 0.00 | ENSG00000264920 | 62.74 | -1.01 | 0.00 |
| ENSG00000275216 | 351.86 | 4.58 | 0.00 | ENSG00000162852 | 903.27 | -1.01 | 0.00 |
| ENSG00000236081 | 370.40 | 4.58 | 0.00 | ENSG00000100485 | 1211.62 | -1.01 | 0.00 |
| ENSG00000142619 | 91.11 | 4.55 | 0.00 | ENSG00000109586 | 2828.17 | -1.01 | 0.00 |
| ENSG00000139515 | 713.07 | 4.54 | 0.00 | ENSG00000081026 | 828.22 | -1.01 | 0.00 |
| ENSG00000165905 | 1823.61 | 4.52 | 0.00 | ENSG00000154889 | 398.02 | -1.01 | 0.00 |
| ENSG00000274979 | 85.91 | 4.50 | 0.00 | ENSG00000068024 | 976.43 | -1.02 | 0.00 |
| ENSG00000250328 | 68.77 | 4.50 | 0.00 | ENSG00000152409 | 656.86 | -1.02 | 0.00 |
| ENSG00000185686 | 111.32 | 4.50 | 0.00 | ENSG00000100614 | 1989.04 | -1.02 | 0.00 |
| ENSG00000088002 | 1106.90 | 4.49 | 0.00 | ENSG00000135454 | 53.61 | -1.02 | 0.01 |
| ENSG00000142700 | 56.21 | 4.48 | 0.00 | ENSG00000169184 | 241.13 | -1.02 | 0.03 |
| ENSG00000167244 | 32460.93 | 4.47 | 0.00 | ENSG00000248508 | 74.71 | -1.02 | 0.00 |
| ENSG00000081277 | 484.88 | 4.47 | 0.00 | ENSG00000162692 | 586.14 | -1.02 | 0.01 |
| ENSG00000213820 | 50.71 | 4.44 | 0.00 | ENSG00000129518 | 1307.35 | -1.02 | 0.00 |
| ENSG00000006377 | 52.74 | 4.42 | 0.00 | ENSG00000147100 | 212.43 | -1.02 | 0.01 |
| ENSG00000238178 | 146.77 | 4.40 | 0.00 | ENSG00000276600 | 92.41 | -1.02 | 0.00 |
| ENSG00000122641 | 1250.30 | 4.39 | 0.00 | ENSG00000080561 | 97.22 | -1.02 | 0.02 |
| ENSG00000254560 | 83.16 | 4.38 | 0.00 | ENSG00000266094 | 461.12 | -1.02 | 0.00 |
| ENSG00000180438 | 52.84 | 4.32 | 0.00 | ENSG00000118922 | 335.94 | -1.02 | 0.03 |
| ENSG00000128683 | 82.87 | 4.29 | 0.00 | ENSG00000163788 | 942.14 | -1.02 | 0.00 |
| ENSG00000130600 | 2527.26 | 4.27 | 0.00 | ENSG00000280798 | 286.47 | -1.02 | 0.00 |
| ENSG00000174171 | 97.21 | 4.27 | 0.00 | ENSG00000110422 | 2223.92 | -1.02 | 0.00 |
| ENSG00000196917 | 126.87 | 4.26 | 0.00 | ENSG00000108061 | 1375.84 | -1.02 | 0.00 |
| ENSG00000198535 | 819.49 | 4.26 | 0.00 | ENSG00000159399 | 5079.75 | -1.02 | 0.00 |
| ENSG00000266402 | 190.37 | 4.25 | 0.00 | ENSG00000107099 | 550.36 | -1.02 | 0.01 |
| ENSG00000165816 | 837.97 | 4.24 | 0.00 | ENSG00000104695 | 2368.86 | -1.02 | 0.00 |
| ENSG00000088836 | 304.24 | 4.24 | 0.00 | ENSG00000076770 | 932.55 | -1.02 | 0.00 |
| ENSG00000255026 | 182.80 | 4.23 | 0.00 | ENSG00000107779 | 932.86 | -1.02 | 0.00 |
| ENSG00000151650 | 256.62 | 4.21 | 0.00 | ENSG00000182107 | 2309.33 | -1.03 | 0.00 |
| ENSG00000164398 | 908.15 | 4.20 | 0.00 | ENSG00000213949 | 2322.72 | -1.03 | 0.00 |
| ENSG00000224114 | 162.35 | 4.19 | 0.00 | ENSG00000104880 | 342.53 | -1.03 | 0.00 |
| ENSG00000088340 | 832.61 | 4.18 | 0.00 | ENSG00000145685 | 1369.08 | -1.03 | 0.00 |
| ENSG00000182747 | 310.73 | 4.15 | 0.00 | ENSG00000101049 | 928.90 | -1.03 | 0.01 |
| ENSG00000181418 | 124.92 | 4.15 | 0.00 | ENSG00000173083 | 307.20 | -1.03 | 0.00 |
| ENSG00000158296 | 400.89 | 4.14 | 0.00 | ENSG00000258289 | 1239.09 | -1.03 | 0.00 |
| ENSG00000183145 | 64.39 | 4.13 | 0.00 | ENSG00000131089 | 791.92 | -1.03 | 0.00 |
| ENSG00000262188 | 136.07 | 4.11 | 0.00 | ENSG00000140367 | 1312.77 | -1.03 | 0.00 |
| ENSG00000213058 | 597.26 | 4.10 | 0.00 | ENSG00000231389 | 4291.84 | -1.03 | 0.03 |
| ENSG00000158764 | 76.28 | 4.09 | 0.00 | ENSG00000106299 | 3035.61 | -1.03 | 0.00 |
| ENSG00000237643 | 551.15 | 4.09 | 0.00 | ENSG00000023445 | 1089.74 | -1.03 | 0.00 |
| ENSG00000104899 | 96.84 | 4.08 | 0.00 | ENSG00000115232 | 341.03 | -1.03 | 0.00 |
| ENSG00000232237 | 61.26 | 4.08 | 0.00 | ENSG00000154723 | 2814.47 | -1.03 | 0.00 |
| ENSG00000232803 | 384.73 | 4.08 | 0.00 | ENSG00000269113 | 78.10 | -1.04 | 0.04 |
| ENSG00000237721 | 116.60 | 4.08 | 0.00 | ENSG00000197208 | 105.96 | -1.04 | 0.00 |
| ENSG00000120149 | 378.91 | 4.07 | 0.00 | ENSG00000090020 | 2474.08 | -1.04 | 0.00 |
| ENSG00000245694 | 164.56 | 4.04 | 0.00 | ENSG00000114107 | 435.70 | -1.04 | 0.00 |
| ENSG00000183248 | 414.20 | 4.03 | 0.00 | ENSG00000117090 | 77.85 | -1.04 | 0.01 |
| ENSG00000214612 | 72.28 | 4.03 | 0.00 | ENSG00000153339 | 1076.91 | -1.04 | 0.00 |
| ENSG00000015520 | 269.69 | 4.01 | 0.00 | ENSG00000116729 | 2635.38 | -1.04 | 0.00 |
| ENSG00000157766 | 401.75 | 3.99 | 0.00 | ENSG00000107758 | 1331.80 | -1.04 | 0.00 |
| ENSG00000064195 | 81.40 | 3.99 | 0.00 | ENSG00000103056 | 1530.82 | -1.04 | 0.00 |
| ENSG00000181085 | 292.92 | 3.97 | 0.00 | ENSG00000140030 | 85.00 | -1.04 | 0.01 |
| ENSG00000165188 | 179.62 | 3.97 | 0.00 | ENSG00000105426 | 472.32 | -1.04 | 0.02 |
| ENSG00000110195 | 247.21 | 3.94 | 0.00 | ENSG00000131791 | 853.53 | -1.04 | 0.00 |
| ENSG00000172031 | 272.97 | 3.93 | 0.00 | ENSG00000083290 | 389.69 | -1.04 | 0.00 |
| ENSG00000120068 | 1117.52 | 3.93 | 0.00 | ENSG00000117010 | 79.26 | -1.04 | 0.00 |
| ENSG00000131188 | 573.28 | 3.92 | 0.00 | ENSG00000156103 | 57.12 | -1.04 | 0.04 |
| ENSG00000187556 | 74.66 | 3.92 | 0.00 | ENSG00000141429 | 2857.30 | -1.04 | 0.00 |
| ENSG00000126583 | 92.53 | 3.91 | 0.00 | ENSG00000085491 | 2361.51 | -1.04 | 0.00 |
| ENSG00000231764 | 64.00 | 3.91 | 0.00 | ENSG00000108797 | 300.67 | -1.04 | 0.01 |
| ENSG00000223749 | 63.26 | 3.90 | 0.00 | ENSG00000162909 | 7744.52 | -1.04 | 0.00 |
| ENSG00000166415 | 453.60 | 3.89 | 0.00 | ENSG00000069956 | 1724.48 | -1.04 | 0.00 |
| ENSG00000213293 | 68.66 | 3.86 | 0.00 | ENSG00000166128 | 594.76 | -1.04 | 0.00 |
| ENSG00000206195 | 76.26 | 3.84 | 0.00 | ENSG00000113638 | 357.38 | -1.04 | 0.00 |
| ENSG00000140807 | 4185.05 | 3.84 | 0.00 | ENSG00000197045 | 2225.71 | -1.04 | 0.00 |
| ENSG00000237686 | 72.77 | 3.82 | 0.00 | ENSG00000156011 | 756.69 | -1.04 | 0.00 |
| ENSG00000196754 | 344.47 | 3.82 | 0.00 | ENSG00000259354 | 56.07 | -1.04 | 0.00 |
| ENSG00000096088 | 57.75 | 3.82 | 0.00 | ENSG00000155760 | 997.96 | -1.04 | 0.01 |
| ENSG00000057593 | 105.36 | 3.81 | 0.00 | ENSG00000129422 | 2035.38 | -1.04 | 0.00 |
| ENSG00000163735 | 698.69 | 3.80 | 0.00 | ENSG00000121350 | 503.11 | -1.04 | 0.00 |
| ENSG00000180176 | 64.67 | 3.80 | 0.00 | ENSG00000095203 | 3957.22 | -1.04 | 0.00 |
| ENSG00000101255 | 3381.34 | 3.79 | 0.00 | ENSG00000118292 | 164.09 | -1.04 | 0.00 |
| ENSG00000181577 | 1593.24 | 3.79 | 0.00 | ENSG00000171488 | 249.64 | -1.05 | 0.00 |
| ENSG00000181652 | 233.86 | 3.79 | 0.00 | ENSG00000205758 | 504.12 | -1.05 | 0.00 |
| ENSG00000173894 | 595.93 | 3.79 | 0.00 | ENSG00000168398 | 745.54 | -1.05 | 0.00 |
| ENSG00000111432 | 332.42 | 3.77 | 0.00 | ENSG00000143933 | 17778.25 | -1.05 | 0.00 |
| ENSG00000204335 | 587.18 | 3.76 | 0.00 | ENSG00000075790 | 1510.09 | -1.05 | 0.00 |
| ENSG00000101104 | 1134.43 | 3.75 | 0.00 | ENSG00000197712 | 1538.76 | -1.05 | 0.00 |
| ENSG00000204421 | 63.14 | 3.73 | 0.00 | ENSG00000187446 | 5103.73 | -1.05 | 0.00 |
| ENSG00000162004 | 125.80 | 3.72 | 0.00 | ENSG00000101844 | 461.76 | -1.05 | 0.00 |
| ENSG00000240950 | 124.49 | 3.72 | 0.00 | ENSG00000122122 | 400.34 | -1.05 | 0.01 |
| ENSG00000136872 | 1130.85 | 3.72 | 0.00 | ENSG00000240583 | 2537.12 | -1.05 | 0.03 |
| ENSG00000160862 | 2904.43 | 3.71 | 0.00 | ENSG00000084676 | 1416.80 | -1.05 | 0.00 |
| ENSG00000210144 | 436.67 | 3.70 | 0.00 | ENSG00000143515 | 518.58 | -1.05 | 0.00 |
| ENSG00000262585 | 87.04 | 3.70 | 0.00 | ENSG00000175662 | 1687.35 | -1.05 | 0.00 |
| ENSG00000151224 | 118.84 | 3.70 | 0.00 | ENSG00000124762 | 5400.89 | -1.05 | 0.00 |
| ENSG00000115363 | 325.37 | 3.69 | 0.00 | ENSG00000063438 | 59.08 | -1.05 | 0.00 |
| ENSG00000103253 | 419.00 | 3.69 | 0.00 | ENSG00000145390 | 1679.80 | -1.05 | 0.00 |
| ENSG00000137747 | 471.54 | 3.69 | 0.00 | ENSG00000147526 | 4092.69 | -1.06 | 0.00 |
| ENSG00000112559 | 564.21 | 3.69 | 0.00 | ENSG00000068305 | 932.58 | -1.06 | 0.00 |
| ENSG00000280109 | 64.71 | 3.69 | 0.00 | ENSG00000244509 | 1149.80 | -1.06 | 0.00 |
| ENSG00000172927 | 1144.90 | 3.68 | 0.00 | ENSG00000138434 | 5545.45 | -1.06 | 0.00 |
| ENSG00000242814 | 64.71 | 3.68 | 0.00 | ENSG00000174348 | 874.99 | -1.06 | 0.04 |
| ENSG00000112494 | 166.38 | 3.68 | 0.00 | ENSG00000138463 | 524.01 | -1.06 | 0.00 |
| ENSG00000114270 | 1424.38 | 3.67 | 0.00 | ENSG00000144655 | 1255.93 | -1.06 | 0.00 |
| ENSG00000163817 | 983.58 | 3.65 | 0.00 | ENSG00000175348 | 2197.46 | -1.06 | 0.00 |
| ENSG00000149243 | 184.68 | 3.63 | 0.00 | ENSG00000135828 | 439.98 | -1.06 | 0.00 |
| ENSG00000243449 | 919.99 | 3.63 | 0.00 | ENSG00000256393 | 143.71 | -1.06 | 0.01 |
| ENSG00000187583 | 161.20 | 3.62 | 0.00 | ENSG00000150907 | 979.66 | -1.06 | 0.00 |
| ENSG00000143839 | 109.73 | 3.61 | 0.00 | ENSG00000183648 | 1320.76 | -1.06 | 0.00 |
| ENSG00000166682 | 89.41 | 3.61 | 0.00 | ENSG00000179362 | 72.22 | -1.07 | 0.00 |
| ENSG00000177984 | 1466.32 | 3.61 | 0.00 | ENSG00000044459 | 141.56 | -1.07 | 0.01 |
| ENSG00000240204 | 74.81 | 3.60 | 0.00 | ENSG00000135617 | 800.36 | -1.07 | 0.00 |
| ENSG00000042832 | 386.11 | 3.59 | 0.00 | ENSG00000119711 | 820.51 | -1.07 | 0.00 |
| ENSG00000197408 | 1308.15 | 3.59 | 0.00 | ENSG00000105227 | 105.02 | -1.07 | 0.00 |
| ENSG00000023839 | 252.05 | 3.59 | 0.00 | ENSG00000180139 | 62.66 | -1.07 | 0.00 |
| ENSG00000246228 | 85.95 | 3.58 | 0.00 | ENSG00000169413 | 321.53 | -1.07 | 0.00 |
| ENSG00000073067 | 3168.64 | 3.58 | 0.00 | ENSG00000198515 | 116.96 | -1.07 | 0.03 |
| ENSG00000187642 | 60.56 | 3.58 | 0.00 | ENSG00000142227 | 734.17 | -1.07 | 0.01 |
| ENSG00000152669 | 316.62 | 3.58 | 0.00 | ENSG00000139194 | 104.24 | -1.07 | 0.00 |
| ENSG00000108576 | 201.48 | 3.57 | 0.00 | ENSG00000111670 | 1975.96 | -1.07 | 0.00 |
| ENSG00000169248 | 364.16 | 3.57 | 0.00 | ENSG00000130449 | 589.58 | -1.07 | 0.00 |
| ENSG00000196335 | 115.15 | 3.57 | 0.00 | ENSG00000171433 | 75.52 | -1.07 | 0.01 |
| ENSG00000225138 | 1000.60 | 3.56 | 0.00 | ENSG00000187554 | 91.52 | -1.07 | 0.01 |
| ENSG00000247844 | 455.55 | 3.56 | 0.00 | ENSG00000071073 | 2063.72 | -1.07 | 0.00 |
| ENSG00000198759 | 136.47 | 3.56 | 0.00 | ENSG00000151692 | 534.03 | -1.07 | 0.00 |
| ENSG00000239528 | 83.96 | 3.55 | 0.00 | ENSG00000048707 | 1736.19 | -1.07 | 0.00 |
| ENSG00000145244 | 97.24 | 3.55 | 0.00 | ENSG00000161955 | 529.22 | -1.07 | 0.00 |
| ENSG00000189280 | 115.89 | 3.55 | 0.00 | ENSG00000172748 | 69.88 | -1.07 | 0.00 |
| ENSG00000196711 | 80.00 | 3.55 | 0.00 | ENSG00000248663 | 78.45 | -1.07 | 0.02 |
| ENSG00000241547 | 80.01 | 3.54 | 0.00 | ENSG00000166710 | 77624.59 | -1.07 | 0.00 |
| ENSG00000101187 | 2948.84 | 3.54 | 0.00 | ENSG00000258818 | 95.98 | -1.08 | 0.00 |
| ENSG00000179698 | 95.54 | 3.54 | 0.00 | ENSG00000181007 | 61.74 | -1.08 | 0.02 |
| ENSG00000141316 | 53.43 | 3.53 | 0.00 | ENSG00000069493 | 121.94 | -1.08 | 0.00 |
| ENSG00000164932 | 1074.27 | 3.53 | 0.00 | ENSG00000100478 | 194.20 | -1.08 | 0.00 |
| ENSG00000136231 | 144.69 | 3.52 | 0.00 | ENSG00000141642 | 166.30 | -1.08 | 0.00 |
| ENSG00000141527 | 179.85 | 3.51 | 0.00 | ENSG00000117791 | 390.63 | -1.08 | 0.00 |
| ENSG00000171388 | 380.97 | 3.51 | 0.00 | ENSG00000178977 | 90.63 | -1.08 | 0.00 |
| ENSG00000158106 | 1110.87 | 3.51 | 0.00 | ENSG00000111859 | 2304.36 | -1.08 | 0.00 |
| ENSG00000253414 | 127.12 | 3.51 | 0.00 | ENSG00000170458 | 1856.26 | -1.08 | 0.01 |
| ENSG00000273604 | 603.69 | 3.50 | 0.00 | ENSG00000119227 | 1314.43 | -1.08 | 0.00 |
| ENSG00000131730 | 633.48 | 3.50 | 0.00 | ENSG00000166046 | 159.46 | -1.08 | 0.00 |
| ENSG00000009950 | 1882.60 | 3.49 | 0.00 | ENSG00000167705 | 404.36 | -1.08 | 0.00 |
| ENSG00000183971 | 104.43 | 3.49 | 0.00 | ENSG00000163406 | 107.09 | -1.08 | 0.00 |
| ENSG00000142102 | 2941.21 | 3.49 | 0.00 | ENSG00000171766 | 489.23 | -1.08 | 0.02 |
| ENSG00000075461 | 227.81 | 3.48 | 0.00 | ENSG00000117528 | 2308.71 | -1.08 | 0.00 |
| ENSG00000148848 | 646.81 | 3.48 | 0.00 | ENSG00000106541 | 23592.39 | -1.08 | 0.02 |
| ENSG00000120708 | 36540.54 | 3.48 | 0.00 | ENSG00000105383 | 56.21 | -1.08 | 0.01 |
| ENSG00000231369 | 68.29 | 3.47 | 0.00 | ENSG00000182463 | 168.01 | -1.08 | 0.03 |
| ENSG00000142235 | 251.32 | 3.46 | 0.00 | ENSG00000156869 | 147.63 | -1.09 | 0.00 |
| ENSG00000233695 | 604.03 | 3.45 | 0.00 | ENSG00000196437 | 64.08 | -1.09 | 0.00 |
| ENSG00000176842 | 64.16 | 3.44 | 0.00 | ENSG00000141540 | 153.73 | -1.09 | 0.00 |
| ENSG00000173391 | 140.99 | 3.42 | 0.00 | ENSG00000172493 | 2179.05 | -1.09 | 0.00 |
| ENSG00000213315 | 195.55 | 3.41 | 0.00 | ENSG00000107742 | 718.07 | -1.09 | 0.00 |
| ENSG00000138100 | 124.79 | 3.39 | 0.00 | ENSG00000170456 | 349.91 | -1.09 | 0.00 |
| ENSG00000163132 | 303.86 | 3.39 | 0.00 | ENSG00000180530 | 1424.83 | -1.09 | 0.00 |
| ENSG00000131746 | 4421.83 | 3.38 | 0.00 | ENSG00000107738 | 1426.10 | -1.09 | 0.00 |
| ENSG00000188338 | 62.27 | 3.37 | 0.00 | ENSG00000136630 | 273.21 | -1.09 | 0.00 |
| ENSG00000163032 | 1034.15 | 3.36 | 0.00 | ENSG00000278948 | 53.92 | -1.09 | 0.00 |
| ENSG00000166670 | 181.12 | 3.36 | 0.00 | ENSG00000117013 | 141.88 | -1.09 | 0.04 |
| ENSG00000125895 | 291.82 | 3.35 | 0.00 | ENSG00000153790 | 192.79 | -1.09 | 0.00 |
| ENSG00000099937 | 79.21 | 3.33 | 0.00 | ENSG00000247774 | 89.90 | -1.09 | 0.00 |
| ENSG00000205702 | 63.75 | 3.33 | 0.00 | ENSG00000137269 | 1467.64 | -1.09 | 0.00 |
| ENSG00000118785 | 4851.86 | 3.33 | 0.00 | ENSG00000157107 | 1121.33 | -1.10 | 0.00 |
| ENSG00000253161 | 97.85 | 3.33 | 0.00 | ENSG00000145780 | 1231.04 | -1.10 | 0.00 |
| ENSG00000113739 | 1186.49 | 3.32 | 0.00 | ENSG00000197013 | 146.67 | -1.10 | 0.00 |
| ENSG00000149295 | 215.07 | 3.32 | 0.00 | ENSG00000196735 | 1875.18 | -1.10 | 0.02 |
| ENSG00000240652 | 85.56 | 3.31 | 0.00 | ENSG00000165406 | 1102.46 | -1.10 | 0.00 |
| ENSG00000179546 | 296.43 | 3.29 | 0.00 | ENSG00000120738 | 6977.64 | -1.10 | 0.02 |
| ENSG00000210140 | 345.78 | 3.29 | 0.00 | ENSG00000165476 | 1609.11 | -1.10 | 0.00 |
| ENSG00000213937 | 95.55 | 3.29 | 0.00 | ENSG00000099785 | 816.78 | -1.10 | 0.00 |
| ENSG00000137877 | 252.42 | 3.28 | 0.00 | ENSG00000235501 | 111.45 | -1.10 | 0.00 |
| ENSG00000158555 | 1676.30 | 3.28 | 0.00 | ENSG00000116117 | 726.95 | -1.10 | 0.00 |
| ENSG00000138028 | 1056.98 | 3.28 | 0.00 | ENSG00000204314 | 78.31 | -1.10 | 0.00 |
| ENSG00000182272 | 524.53 | 3.27 | 0.00 | ENSG00000122862 | 1628.88 | -1.10 | 0.02 |
| ENSG00000131389 | 4888.86 | 3.26 | 0.00 | ENSG00000115935 | 1138.81 | -1.10 | 0.00 |
| ENSG00000110680 | 51.46 | 3.26 | 0.00 | ENSG00000269293 | 90.71 | -1.10 | 0.00 |
| ENSG00000100867 | 113.51 | 3.25 | 0.00 | ENSG00000266412 | 5832.56 | -1.11 | 0.00 |
| ENSG00000140274 | 1173.62 | 3.24 | 0.00 | ENSG00000113263 | 97.72 | -1.11 | 0.01 |
| ENSG00000102359 | 1276.13 | 3.24 | 0.00 | ENSG00000260966 | 78.95 | -1.11 | 0.00 |
| ENSG00000181333 | 53.32 | 3.24 | 0.00 | ENSG00000166428 | 63.78 | -1.11 | 0.03 |
| ENSG00000138152 | 50.81 | 3.22 | 0.00 | ENSG00000198668 | 15023.34 | -1.11 | 0.00 |
| ENSG00000170561 | 281.40 | 3.18 | 0.00 | ENSG00000109794 | 217.29 | -1.11 | 0.01 |
| ENSG00000114656 | 158.25 | 3.16 | 0.00 | ENSG00000196569 | 460.65 | -1.11 | 0.03 |
| ENSG00000134339 | 89.73 | 3.16 | 0.00 | ENSG00000137825 | 496.28 | -1.11 | 0.02 |
| ENSG00000133048 | 1188.76 | 3.16 | 0.00 | ENSG00000136167 | 2343.98 | -1.11 | 0.01 |
| ENSG00000204876 | 853.19 | 3.16 | 0.00 | ENSG00000154864 | 134.34 | -1.11 | 0.02 |
| ENSG00000175894 | 65.08 | 3.16 | 0.00 | ENSG00000174282 | 1871.24 | -1.11 | 0.00 |
| ENSG00000159261 | 56.82 | 3.15 | 0.00 | ENSG00000152270 | 822.93 | -1.11 | 0.00 |
| ENSG00000187867 | 153.67 | 3.14 | 0.00 | ENSG00000171444 | 209.37 | -1.11 | 0.00 |
| ENSG00000102854 | 2792.24 | 3.13 | 0.00 | ENSG00000128609 | 1959.35 | -1.11 | 0.00 |
| ENSG00000227619 | 131.53 | 3.13 | 0.00 | ENSG00000197702 | 2884.10 | -1.12 | 0.00 |
| ENSG00000147889 | 391.68 | 3.13 | 0.00 | ENSG00000173372 | 2107.61 | -1.12 | 0.00 |
| ENSG00000226085 | 82.16 | 3.12 | 0.00 | ENSG00000134982 | 734.42 | -1.12 | 0.00 |
| ENSG00000124602 | 623.81 | 3.11 | 0.00 | ENSG00000158158 | 3031.50 | -1.12 | 0.00 |
| ENSG00000186493 | 83.29 | 3.11 | 0.00 | ENSG00000197381 | 441.56 | -1.12 | 0.00 |
| ENSG00000187730 | 64.05 | 3.11 | 0.00 | ENSG00000187079 | 2035.10 | -1.12 | 0.00 |
| ENSG00000085741 | 1146.76 | 3.10 | 0.00 | ENSG00000150054 | 869.28 | -1.12 | 0.00 |
| ENSG00000265688 | 233.13 | 3.10 | 0.00 | ENSG00000158006 | 1010.99 | -1.12 | 0.00 |
| ENSG00000148734 | 179.27 | 3.09 | 0.00 | ENSG00000261534 | 75.47 | -1.12 | 0.00 |
| ENSG00000166840 | 88.13 | 3.08 | 0.00 | ENSG00000204175 | 341.14 | -1.12 | 0.01 |
| ENSG00000261373 | 524.61 | 3.08 | 0.00 | ENSG00000160613 | 1786.50 | -1.12 | 0.00 |
| ENSG00000180537 | 61.77 | 3.07 | 0.00 | ENSG00000279117 | 348.61 | -1.12 | 0.00 |
| ENSG00000248626 | 53.28 | 3.07 | 0.00 | ENSG00000128739 | 512.18 | -1.12 | 0.02 |
| ENSG00000139055 | 345.68 | 3.07 | 0.00 | ENSG00000110318 | 138.64 | -1.12 | 0.00 |
| ENSG00000137699 | 3342.28 | 3.07 | 0.00 | ENSG00000109743 | 181.06 | -1.12 | 0.01 |
| ENSG00000258227 | 74.19 | 3.06 | 0.00 | ENSG00000165959 | 1865.36 | -1.12 | 0.00 |
| ENSG00000008300 | 845.68 | 3.04 | 0.00 | ENSG00000185052 | 421.26 | -1.12 | 0.01 |
| ENSG00000100453 | 475.40 | 3.04 | 0.00 | ENSG00000120899 | 1767.26 | -1.12 | 0.00 |
| ENSG00000106327 | 156.21 | 3.04 | 0.00 | ENSG00000171055 | 1068.41 | -1.13 | 0.00 |
| ENSG00000269242 | 132.40 | 3.04 | 0.00 | ENSG00000256043 | 829.05 | -1.13 | 0.00 |
| ENSG00000211676 | 67.89 | 3.03 | 0.01 | ENSG00000170270 | 399.51 | -1.13 | 0.00 |
| ENSG00000101850 | 371.95 | 3.03 | 0.00 | ENSG00000170647 | 150.61 | -1.13 | 0.00 |
| ENSG00000139292 | 2756.91 | 3.02 | 0.00 | ENSG00000235109 | 237.79 | -1.13 | 0.00 |
| ENSG00000180730 | 136.07 | 3.02 | 0.00 | ENSG00000102524 | 166.87 | -1.13 | 0.02 |
| ENSG00000225093 | 121.05 | 3.02 | 0.00 | ENSG00000171992 | 2416.86 | -1.13 | 0.00 |
| ENSG00000169429 | 2664.17 | 3.02 | 0.00 | ENSG00000086619 | 175.93 | -1.13 | 0.00 |
| ENSG00000151388 | 742.42 | 3.02 | 0.00 | ENSG00000137076 | 12241.75 | -1.13 | 0.00 |
| ENSG00000101074 | 78.29 | 3.01 | 0.00 | ENSG00000137414 | 1527.42 | -1.13 | 0.00 |
| ENSG00000232445 | 180.65 | 3.00 | 0.00 | ENSG00000249751 | 56.00 | -1.13 | 0.00 |
| ENSG00000237988 | 370.09 | 3.00 | 0.00 | ENSG00000145416 | 182.66 | -1.13 | 0.00 |
| ENSG00000262370 | 114.66 | 3.00 | 0.00 | ENSG00000005844 | 383.85 | -1.13 | 0.00 |
| ENSG00000167757 | 285.18 | 2.99 | 0.00 | ENSG00000117682 | 1332.06 | -1.14 | 0.00 |
| ENSG00000215417 | 116.02 | 2.99 | 0.00 | ENSG00000277443 | 6512.93 | -1.14 | 0.00 |
| ENSG00000279184 | 63.03 | 2.97 | 0.00 | ENSG00000233452 | 51.32 | -1.14 | 0.00 |
| ENSG00000092758 | 991.13 | 2.97 | 0.00 | ENSG00000082074 | 313.33 | -1.14 | 0.01 |
| ENSG00000254726 | 640.82 | 2.97 | 0.00 | ENSG00000204385 | 11152.33 | -1.14 | 0.00 |
| ENSG00000173557 | 210.00 | 2.97 | 0.00 | ENSG00000155744 | 619.10 | -1.14 | 0.00 |
| ENSG00000279806 | 204.43 | 2.97 | 0.00 | ENSG00000179476 | 123.28 | -1.14 | 0.00 |
| ENSG00000189433 | 152.45 | 2.97 | 0.00 | ENSG00000170989 | 417.60 | -1.14 | 0.00 |
| ENSG00000272502 | 217.52 | 2.96 | 0.00 | ENSG00000068383 | 1060.21 | -1.14 | 0.00 |
| ENSG00000105707 | 184.80 | 2.96 | 0.00 | ENSG00000150281 | 66.60 | -1.14 | 0.03 |
| ENSG00000158014 | 164.42 | 2.96 | 0.00 | ENSG00000163694 | 5133.46 | -1.14 | 0.00 |
| ENSG00000203499 | 1322.13 | 2.95 | 0.00 | ENSG00000184898 | 205.85 | -1.14 | 0.00 |
| ENSG00000174951 | 329.90 | 2.94 | 0.00 | ENSG00000120137 | 2565.13 | -1.14 | 0.00 |
| ENSG00000159708 | 144.36 | 2.94 | 0.00 | ENSG00000143669 | 435.46 | -1.14 | 0.00 |
| ENSG00000243716 | 209.03 | 2.94 | 0.00 | ENSG00000156642 | 3589.64 | -1.14 | 0.00 |
| ENSG00000135960 | 749.82 | 2.93 | 0.00 | ENSG00000164463 | 600.00 | -1.14 | 0.00 |
| ENSG00000249859 | 545.47 | 2.93 | 0.00 | ENSG00000111897 | 4450.07 | -1.14 | 0.00 |
| ENSG00000106038 | 324.46 | 2.92 | 0.00 | ENSG00000227507 | 256.35 | -1.14 | 0.00 |
| ENSG00000141505 | 204.50 | 2.92 | 0.00 | ENSG00000110934 | 234.89 | -1.14 | 0.00 |
| ENSG00000103257 | 7700.84 | 2.92 | 0.00 | ENSG00000123612 | 210.42 | -1.14 | 0.01 |
| ENSG00000230076 | 111.28 | 2.91 | 0.00 | ENSG00000272106 | 127.31 | -1.15 | 0.00 |
| ENSG00000101144 | 2014.22 | 2.91 | 0.00 | ENSG00000189067 | 5061.65 | -1.15 | 0.00 |
| ENSG00000163739 | 2925.54 | 2.91 | 0.00 | ENSG00000157184 | 1177.86 | -1.15 | 0.00 |
| ENSG00000173898 | 1346.67 | 2.89 | 0.00 | ENSG00000175899 | 10493.31 | -1.15 | 0.00 |
| ENSG00000241186 | 1160.77 | 2.89 | 0.00 | ENSG00000197128 | 89.38 | -1.15 | 0.02 |
| ENSG00000163083 | 414.59 | 2.88 | 0.00 | ENSG00000161638 | 2676.53 | -1.15 | 0.00 |
| ENSG00000116017 | 2167.29 | 2.87 | 0.00 | ENSG00000180861 | 1123.66 | -1.15 | 0.01 |
| ENSG00000135744 | 1187.32 | 2.87 | 0.00 | ENSG00000074527 | 610.87 | -1.15 | 0.00 |
| ENSG00000197582 | 165.26 | 2.86 | 0.00 | ENSG00000155090 | 2423.07 | -1.15 | 0.00 |
| ENSG00000168243 | 2011.74 | 2.85 | 0.00 | ENSG00000082014 | 270.28 | -1.16 | 0.00 |
| ENSG00000184368 | 571.09 | 2.85 | 0.00 | ENSG00000155957 | 1282.88 | -1.16 | 0.00 |
| ENSG00000270933 | 85.67 | 2.85 | 0.00 | ENSG00000167644 | 1570.42 | -1.16 | 0.00 |
| ENSG00000183091 | 96.30 | 2.85 | 0.00 | ENSG00000170522 | 1220.69 | -1.16 | 0.00 |
| ENSG00000185666 | 123.72 | 2.85 | 0.00 | ENSG00000180353 | 692.63 | -1.16 | 0.00 |
| ENSG00000102287 | 1471.75 | 2.84 | 0.00 | ENSG00000119899 | 1226.78 | -1.16 | 0.00 |
| ENSG00000243587 | 104.75 | 2.83 | 0.00 | ENSG00000101745 | 777.25 | -1.16 | 0.00 |
| ENSG00000204839 | 1242.80 | 2.83 | 0.00 | ENSG00000081320 | 1273.95 | -1.16 | 0.00 |
| ENSG00000172460 | 52.82 | 2.83 | 0.00 | ENSG00000117620 | 2843.29 | -1.16 | 0.00 |
| ENSG00000171004 | 497.99 | 2.82 | 0.00 | ENSG00000119900 | 473.12 | -1.16 | 0.00 |
| ENSG00000235297 | 93.24 | 2.81 | 0.00 | ENSG00000235505 | 66.22 | -1.16 | 0.01 |
| ENSG00000279487 | 50.29 | 2.81 | 0.00 | ENSG00000100368 | 417.73 | -1.16 | 0.01 |
| ENSG00000119547 | 128.44 | 2.81 | 0.00 | ENSG00000204252 | 387.14 | -1.16 | 0.01 |
| ENSG00000129474 | 883.23 | 2.81 | 0.00 | ENSG00000128487 | 902.26 | -1.16 | 0.00 |
| ENSG00000280587 | 52.14 | 2.81 | 0.00 | ENSG00000166912 | 711.70 | -1.16 | 0.00 |
| ENSG00000197191 | 55.46 | 2.80 | 0.00 | ENSG00000100266 | 3296.22 | -1.16 | 0.00 |
| ENSG00000232176 | 63.38 | 2.79 | 0.00 | ENSG00000103544 | 994.17 | -1.16 | 0.00 |
| ENSG00000169247 | 387.23 | 2.79 | 0.00 | ENSG00000163346 | 3053.11 | -1.17 | 0.00 |
| ENSG00000125531 | 57.45 | 2.79 | 0.00 | ENSG00000213463 | 1251.77 | -1.17 | 0.00 |
| ENSG00000185885 | 12000.61 | 2.78 | 0.00 | ENSG00000113532 | 217.60 | -1.17 | 0.00 |
| ENSG00000108821 | 94004.72 | 2.78 | 0.00 | ENSG00000173040 | 51.57 | -1.17 | 0.01 |
| ENSG00000240616 | 81.62 | 2.78 | 0.00 | ENSG00000182578 | 1155.48 | -1.17 | 0.01 |
| ENSG00000180211 | 92.67 | 2.78 | 0.00 | ENSG00000171777 | 52.36 | -1.17 | 0.01 |
| ENSG00000106483 | 1706.37 | 2.78 | 0.00 | ENSG00000107282 | 281.28 | -1.17 | 0.00 |
| ENSG00000162849 | 474.68 | 2.77 | 0.00 | ENSG00000088256 | 4821.03 | -1.17 | 0.00 |
| ENSG00000168646 | 6182.36 | 2.77 | 0.00 | ENSG00000101558 | 3578.58 | -1.17 | 0.00 |
| ENSG00000135069 | 2147.77 | 2.77 | 0.00 | ENSG00000204219 | 2034.94 | -1.17 | 0.00 |
| ENSG00000179431 | 214.43 | 2.76 | 0.00 | ENSG00000162378 | 937.53 | -1.17 | 0.00 |
| ENSG00000174672 | 279.34 | 2.76 | 0.00 | ENSG00000079739 | 2433.36 | -1.17 | 0.00 |
| ENSG00000226608 | 337.59 | 2.76 | 0.00 | ENSG00000115738 | 2941.61 | -1.17 | 0.00 |
| ENSG00000182352 | 85.84 | 2.75 | 0.00 | ENSG00000173482 | 645.45 | -1.17 | 0.00 |
| ENSG00000050344 | 4958.76 | 2.75 | 0.00 | ENSG00000153179 | 2644.58 | -1.17 | 0.00 |
| ENSG00000163734 | 1637.25 | 2.74 | 0.00 | ENSG00000067715 | 184.31 | -1.17 | 0.05 |
| ENSG00000213763 | 139.88 | 2.74 | 0.00 | ENSG00000083720 | 848.10 | -1.17 | 0.00 |
| ENSG00000278919 | 93.14 | 2.73 | 0.00 | ENSG00000171608 | 325.93 | -1.18 | 0.00 |
| ENSG00000275437 | 109.80 | 2.73 | 0.00 | ENSG00000174600 | 269.05 | -1.18 | 0.00 |
| ENSG00000215030 | 2122.20 | 2.73 | 0.00 | ENSG00000142583 | 160.32 | -1.18 | 0.02 |
| ENSG00000186377 | 369.18 | 2.72 | 0.00 | ENSG00000135835 | 81.07 | -1.18 | 0.00 |
| ENSG00000109255 | 204.20 | 2.72 | 0.00 | ENSG00000118762 | 586.95 | -1.18 | 0.00 |
| ENSG00000162383 | 376.70 | 2.71 | 0.00 | ENSG00000204323 | 80.59 | -1.18 | 0.01 |
| ENSG00000184160 | 260.49 | 2.71 | 0.00 | ENSG00000064225 | 71.94 | -1.18 | 0.00 |
| ENSG00000273079 | 231.78 | 2.70 | 0.00 | ENSG00000197375 | 895.69 | -1.18 | 0.00 |
| ENSG00000099985 | 189.47 | 2.70 | 0.00 | ENSG00000224078 | 430.59 | -1.18 | 0.05 |
| ENSG00000204740 | 88.90 | 2.70 | 0.00 | ENSG00000196177 | 901.30 | -1.18 | 0.00 |
| ENSG00000125508 | 221.52 | 2.70 | 0.00 | ENSG00000176148 | 237.02 | -1.18 | 0.00 |
| ENSG00000213886 | 417.10 | 2.69 | 0.00 | ENSG00000152527 | 138.37 | -1.18 | 0.00 |
| ENSG00000182489 | 255.73 | 2.68 | 0.00 | ENSG00000170464 | 178.33 | -1.18 | 0.00 |
| ENSG00000149599 | 151.03 | 2.68 | 0.00 | ENSG00000112139 | 96.61 | -1.18 | 0.01 |
| ENSG00000026036 | 221.57 | 2.67 | 0.00 | ENSG00000137601 | 296.88 | -1.18 | 0.00 |
| ENSG00000196155 | 1553.67 | 2.67 | 0.00 | ENSG00000139132 | 808.87 | -1.18 | 0.00 |
| ENSG00000116661 | 417.98 | 2.67 | 0.00 | ENSG00000129473 | 1369.70 | -1.18 | 0.00 |
| ENSG00000184489 | 3043.05 | 2.67 | 0.00 | ENSG00000255112 | 2148.38 | -1.18 | 0.00 |
| ENSG00000230291 | 50.79 | 2.66 | 0.00 | ENSG00000167315 | 3941.98 | -1.18 | 0.00 |
| ENSG00000148346 | 23619.84 | 2.66 | 0.00 | ENSG00000162688 | 908.82 | -1.19 | 0.00 |
| ENSG00000088882 | 597.68 | 2.66 | 0.00 | ENSG00000242574 | 853.08 | -1.19 | 0.01 |
| ENSG00000155265 | 400.29 | 2.66 | 0.00 | ENSG00000178498 | 349.78 | -1.19 | 0.00 |
| ENSG00000205502 | 305.12 | 2.66 | 0.00 | ENSG00000148660 | 1888.82 | -1.19 | 0.00 |
| ENSG00000135480 | 222.35 | 2.65 | 0.00 | ENSG00000117758 | 1522.63 | -1.19 | 0.00 |
| ENSG00000156427 | 92.30 | 2.65 | 0.00 | ENSG00000096060 | 1577.29 | -1.19 | 0.00 |
| ENSG00000220472 | 102.07 | 2.65 | 0.00 | ENSG00000106066 | 667.47 | -1.19 | 0.01 |
| ENSG00000169174 | 2607.80 | 2.64 | 0.00 | ENSG00000159921 | 2951.84 | -1.19 | 0.01 |
| ENSG00000186474 | 142.21 | 2.64 | 0.00 | ENSG00000166265 | 223.66 | -1.19 | 0.00 |
| ENSG00000188483 | 646.30 | 2.63 | 0.00 | ENSG00000178537 | 787.52 | -1.19 | 0.00 |
| ENSG00000182492 | 9923.45 | 2.63 | 0.00 | ENSG00000163219 | 211.06 | -1.19 | 0.00 |
| ENSG00000005001 | 982.68 | 2.63 | 0.00 | ENSG00000069667 | 229.11 | -1.19 | 0.00 |
| ENSG00000238133 | 52.42 | 2.63 | 0.00 | ENSG00000206418 | 546.35 | -1.19 | 0.00 |
| ENSG00000131650 | 85.71 | 2.63 | 0.00 | ENSG00000131981 | 28600.51 | -1.19 | 0.00 |
| ENSG00000209082 | 56.62 | 2.61 | 0.00 | ENSG00000112874 | 640.70 | -1.19 | 0.00 |
| ENSG00000142544 | 411.68 | 2.61 | 0.00 | ENSG00000105967 | 89.23 | -1.19 | 0.01 |
| ENSG00000280693 | 166.69 | 2.61 | 0.00 | ENSG00000009790 | 112.06 | -1.19 | 0.00 |
| ENSG00000167165 | 52.03 | 2.60 | 0.00 | ENSG00000152234 | 14358.28 | -1.20 | 0.00 |
| ENSG00000233922 | 52.80 | 2.60 | 0.00 | ENSG00000153233 | 390.14 | -1.20 | 0.04 |
| ENSG00000127418 | 3422.78 | 2.59 | 0.00 | ENSG00000135604 | 81.01 | -1.20 | 0.00 |
| ENSG00000180914 | 80.58 | 2.59 | 0.00 | ENSG00000162998 | 737.07 | -1.20 | 0.01 |
| ENSG00000220583 | 63.98 | 2.59 | 0.00 | ENSG00000148411 | 1312.79 | -1.20 | 0.00 |
| ENSG00000225356 | 58.14 | 2.56 | 0.00 | ENSG00000178184 | 142.90 | -1.20 | 0.00 |
| ENSG00000196756 | 2009.17 | 2.55 | 0.00 | ENSG00000180354 | 1011.16 | -1.20 | 0.00 |
| ENSG00000108379 | 142.78 | 2.55 | 0.00 | ENSG00000119801 | 2320.49 | -1.20 | 0.00 |
| ENSG00000226396 | 89.92 | 2.54 | 0.00 | ENSG00000005243 | 210.77 | -1.20 | 0.00 |
| ENSG00000232888 | 233.72 | 2.54 | 0.00 | ENSG00000075142 | 6414.41 | -1.20 | 0.00 |
| ENSG00000160957 | 1952.06 | 2.54 | 0.00 | ENSG00000116209 | 10331.39 | -1.20 | 0.00 |
| ENSG00000134317 | 210.95 | 2.54 | 0.00 | ENSG00000214900 | 92.26 | -1.20 | 0.00 |
| ENSG00000106236 | 643.72 | 2.53 | 0.00 | ENSG00000215193 | 2469.10 | -1.21 | 0.00 |
| ENSG00000109205 | 171.43 | 2.53 | 0.00 | ENSG00000172236 | 290.88 | -1.21 | 0.05 |
| ENSG00000006118 | 1587.00 | 2.53 | 0.00 | ENSG00000188549 | 588.83 | -1.21 | 0.00 |
| ENSG00000233913 | 421.13 | 2.53 | 0.00 | ENSG00000115112 | 2757.09 | -1.21 | 0.00 |
| ENSG00000156076 | 141.56 | 2.53 | 0.02 | ENSG00000169435 | 725.07 | -1.21 | 0.00 |
| ENSG00000003249 | 894.62 | 2.52 | 0.00 | ENSG00000041353 | 295.80 | -1.21 | 0.03 |
| ENSG00000185615 | 70.69 | 2.52 | 0.00 | ENSG00000165029 | 808.54 | -1.21 | 0.00 |
| ENSG00000087116 | 1600.76 | 2.51 | 0.00 | ENSG00000137393 | 197.84 | -1.21 | 0.00 |
| ENSG00000189120 | 963.89 | 2.51 | 0.00 | ENSG00000177576 | 181.10 | -1.21 | 0.00 |
| ENSG00000102878 | 403.44 | 2.51 | 0.00 | ENSG00000163131 | 8398.33 | -1.21 | 0.00 |
| ENSG00000196549 | 1122.42 | 2.51 | 0.00 | ENSG00000107317 | 619.59 | -1.21 | 0.02 |
| ENSG00000215915 | 136.48 | 2.51 | 0.00 | ENSG00000135299 | 85.98 | -1.21 | 0.00 |
| ENSG00000130748 | 801.68 | 2.51 | 0.00 | ENSG00000156052 | 2362.07 | -1.21 | 0.00 |
| ENSG00000160183 | 419.82 | 2.51 | 0.00 | ENSG00000134758 | 847.88 | -1.22 | 0.00 |
| ENSG00000151846 | 99.12 | 2.50 | 0.00 | ENSG00000172340 | 4018.53 | -1.22 | 0.00 |
| ENSG00000141738 | 2614.89 | 2.50 | 0.00 | ENSG00000162711 | 87.24 | -1.22 | 0.00 |
| ENSG00000068650 | 3785.18 | 2.50 | 0.00 | ENSG00000138641 | 758.93 | -1.22 | 0.00 |
| ENSG00000108375 | 10571.19 | 2.49 | 0.00 | ENSG00000117226 | 1577.68 | -1.22 | 0.00 |
| ENSG00000197905 | 1123.53 | 2.48 | 0.00 | ENSG00000003436 | 541.28 | -1.22 | 0.00 |
| ENSG00000154856 | 4755.36 | 2.48 | 0.00 | ENSG00000143416 | 12466.24 | -1.22 | 0.01 |
| ENSG00000166278 | 2290.80 | 2.48 | 0.00 | ENSG00000140092 | 634.71 | -1.22 | 0.00 |
| ENSG00000018280 | 300.29 | 2.47 | 0.00 | ENSG00000109436 | 506.63 | -1.22 | 0.00 |
| ENSG00000088899 | 1649.24 | 2.46 | 0.00 | ENSG00000126821 | 546.81 | -1.22 | 0.00 |
| ENSG00000183742 | 2443.45 | 2.45 | 0.00 | ENSG00000113742 | 711.40 | -1.22 | 0.00 |
| ENSG00000101224 | 6168.01 | 2.44 | 0.00 | ENSG00000138764 | 1185.72 | -1.22 | 0.00 |
| ENSG00000164620 | 132.14 | 2.43 | 0.00 | ENSG00000100433 | 50.97 | -1.22 | 0.02 |
| ENSG00000198720 | 729.05 | 2.43 | 0.00 | ENSG00000117152 | 99.64 | -1.23 | 0.03 |
| ENSG00000107984 | 57.20 | 2.43 | 0.00 | ENSG00000124191 | 115.84 | -1.23 | 0.00 |
| ENSG00000105717 | 141.72 | 2.43 | 0.00 | ENSG00000133619 | 74.02 | -1.23 | 0.00 |
| ENSG00000172456 | 1744.93 | 2.42 | 0.00 | ENSG00000142867 | 1279.21 | -1.23 | 0.00 |
| ENSG00000203805 | 88.21 | 2.42 | 0.00 | ENSG00000118496 | 684.78 | -1.23 | 0.00 |
| ENSG00000125207 | 111.11 | 2.42 | 0.00 | ENSG00000111885 | 2087.36 | -1.23 | 0.00 |
| ENSG00000181029 | 134.23 | 2.42 | 0.00 | ENSG00000170296 | 1036.43 | -1.23 | 0.00 |
| ENSG00000110944 | 94.59 | 2.41 | 0.00 | ENSG00000174130 | 51.55 | -1.23 | 0.00 |
| ENSG00000235888 | 88.13 | 2.41 | 0.00 | ENSG00000197892 | 2818.21 | -1.23 | 0.00 |
| ENSG00000160161 | 77.19 | 2.40 | 0.00 | ENSG00000164414 | 546.94 | -1.23 | 0.00 |
| ENSG00000157193 | 807.53 | 2.40 | 0.00 | ENSG00000088881 | 183.45 | -1.23 | 0.00 |
| ENSG00000183914 | 132.36 | 2.40 | 0.00 | ENSG00000182287 | 553.37 | -1.23 | 0.00 |
| ENSG00000196739 | 1030.69 | 2.39 | 0.00 | ENSG00000115993 | 1687.19 | -1.23 | 0.00 |
| ENSG00000143882 | 301.87 | 2.39 | 0.00 | ENSG00000106123 | 95.74 | -1.23 | 0.03 |
| ENSG00000272899 | 188.66 | 2.39 | 0.00 | ENSG00000159212 | 164.40 | -1.24 | 0.03 |
| ENSG00000276644 | 1574.10 | 2.39 | 0.00 | ENSG00000168490 | 55.98 | -1.24 | 0.03 |
| ENSG00000183960 | 203.34 | 2.39 | 0.00 | ENSG00000137936 | 676.41 | -1.24 | 0.00 |
| ENSG00000188176 | 87.50 | 2.38 | 0.00 | ENSG00000198643 | 10545.67 | -1.24 | 0.02 |
| ENSG00000078098 | 439.49 | 2.38 | 0.00 | ENSG00000103034 | 174.01 | -1.24 | 0.00 |
| ENSG00000006704 | 2950.13 | 2.38 | 0.00 | ENSG00000155307 | 182.45 | -1.24 | 0.00 |
| ENSG00000218175 | 446.73 | 2.38 | 0.00 | ENSG00000118564 | 2008.60 | -1.24 | 0.00 |
| ENSG00000203896 | 168.17 | 2.38 | 0.00 | ENSG00000235863 | 399.17 | -1.24 | 0.00 |
| ENSG00000130513 | 6463.47 | 2.37 | 0.00 | ENSG00000163520 | 1704.25 | -1.24 | 0.03 |
| ENSG00000234753 | 98.19 | 2.36 | 0.00 | ENSG00000110002 | 1338.48 | -1.24 | 0.00 |
| ENSG00000117122 | 753.93 | 2.36 | 0.00 | ENSG00000135052 | 9269.93 | -1.24 | 0.00 |
| ENSG00000104808 | 57.42 | 2.36 | 0.00 | ENSG00000133731 | 931.65 | -1.24 | 0.00 |
| ENSG00000149043 | 89.97 | 2.35 | 0.00 | ENSG00000187601 | 328.84 | -1.24 | 0.00 |
| ENSG00000184916 | 1897.56 | 2.35 | 0.00 | ENSG00000058272 | 1919.66 | -1.24 | 0.00 |
| ENSG00000182324 | 99.49 | 2.35 | 0.00 | ENSG00000186496 | 53.75 | -1.24 | 0.00 |
| ENSG00000235587 | 98.65 | 2.34 | 0.00 | ENSG00000116171 | 4358.15 | -1.25 | 0.00 |
| ENSG00000149634 | 55.68 | 2.34 | 0.00 | ENSG00000137628 | 1080.70 | -1.25 | 0.00 |
| ENSG00000134323 | 104.21 | 2.34 | 0.00 | ENSG00000072840 | 265.09 | -1.25 | 0.01 |
| ENSG00000017483 | 3797.96 | 2.33 | 0.00 | ENSG00000067082 | 6312.59 | -1.25 | 0.00 |
| ENSG00000238279 | 105.91 | 2.33 | 0.00 | ENSG00000204128 | 882.97 | -1.25 | 0.00 |
| ENSG00000188883 | 55.34 | 2.33 | 0.00 | ENSG00000138166 | 813.00 | -1.25 | 0.00 |
| ENSG00000223705 | 611.25 | 2.33 | 0.00 | ENSG00000135363 | 208.03 | -1.25 | 0.00 |
| ENSG00000273142 | 310.50 | 2.31 | 0.00 | ENSG00000158517 | 68.61 | -1.25 | 0.01 |
| ENSG00000174939 | 318.13 | 2.31 | 0.00 | ENSG00000196923 | 2076.40 | -1.25 | 0.00 |
| ENSG00000102837 | 60808.24 | 2.31 | 0.01 | ENSG00000154265 | 546.09 | -1.25 | 0.00 |
| ENSG00000280219 | 50.70 | 2.31 | 0.00 | ENSG00000162104 | 898.89 | -1.25 | 0.00 |
| ENSG00000186862 | 60.91 | 2.30 | 0.00 | ENSG00000101916 | 62.67 | -1.25 | 0.02 |
| ENSG00000244300 | 178.58 | 2.30 | 0.00 | ENSG00000198695 | 19845.26 | -1.25 | 0.00 |
| ENSG00000163618 | 829.92 | 2.30 | 0.00 | ENSG00000120063 | 2358.01 | -1.25 | 0.00 |
| ENSG00000189366 | 100.25 | 2.29 | 0.00 | ENSG00000198189 | 3744.97 | -1.25 | 0.00 |
| ENSG00000142661 | 949.35 | 2.29 | 0.00 | ENSG00000157315 | 52.29 | -1.26 | 0.00 |
| ENSG00000132000 | 287.03 | 2.29 | 0.00 | ENSG00000076258 | 122.81 | -1.26 | 0.00 |
| ENSG00000099625 | 61.64 | 2.28 | 0.00 | ENSG00000137266 | 1363.51 | -1.26 | 0.00 |
| ENSG00000243742 | 102.87 | 2.28 | 0.00 | ENSG00000163171 | 968.02 | -1.26 | 0.00 |
| ENSG00000198723 | 142.42 | 2.28 | 0.00 | ENSG00000066926 | 450.64 | -1.26 | 0.00 |
| ENSG00000081041 | 1003.63 | 2.28 | 0.00 | ENSG00000163293 | 905.34 | -1.26 | 0.00 |
| ENSG00000173531 | 224.02 | 2.27 | 0.00 | ENSG00000174804 | 481.67 | -1.26 | 0.00 |
| ENSG00000164690 | 1032.37 | 2.27 | 0.00 | ENSG00000116774 | 926.30 | -1.26 | 0.01 |
| ENSG00000142185 | 1073.95 | 2.27 | 0.00 | ENSG00000104043 | 66.96 | -1.26 | 0.00 |
| ENSG00000261295 | 61.14 | 2.26 | 0.00 | ENSG00000113924 | 340.60 | -1.27 | 0.01 |
| ENSG00000133067 | 947.69 | 2.26 | 0.00 | ENSG00000161714 | 3803.29 | -1.27 | 0.00 |
| ENSG00000228594 | 528.28 | 2.26 | 0.00 | ENSG00000139567 | 2735.39 | -1.27 | 0.00 |
| ENSG00000101194 | 2324.41 | 2.25 | 0.00 | ENSG00000123091 | 1806.48 | -1.27 | 0.00 |
| ENSG00000088826 | 985.76 | 2.25 | 0.00 | ENSG00000152804 | 116.68 | -1.27 | 0.00 |
| ENSG00000158023 | 112.40 | 2.25 | 0.00 | ENSG00000274012 | 938.48 | -1.27 | 0.02 |
| ENSG00000236144 | 429.71 | 2.25 | 0.00 | ENSG00000181751 | 981.11 | -1.27 | 0.00 |
| ENSG00000123405 | 54.85 | 2.25 | 0.00 | ENSG00000153283 | 124.51 | -1.27 | 0.00 |
| ENSG00000189431 | 273.42 | 2.25 | 0.00 | ENSG00000110076 | 287.01 | -1.27 | 0.05 |
| ENSG00000270885 | 130.77 | 2.24 | 0.00 | ENSG00000139874 | 242.40 | -1.27 | 0.02 |
| ENSG00000232442 | 277.84 | 2.24 | 0.00 | ENSG00000122188 | 56.41 | -1.27 | 0.01 |
| ENSG00000122778 | 1069.81 | 2.24 | 0.00 | ENSG00000175600 | 104.16 | -1.27 | 0.00 |
| ENSG00000131910 | 141.76 | 2.23 | 0.00 | ENSG00000138639 | 161.77 | -1.27 | 0.00 |
| ENSG00000184925 | 202.69 | 2.23 | 0.00 | ENSG00000090006 | 5274.08 | -1.27 | 0.00 |
| ENSG00000135245 | 1193.95 | 2.23 | 0.00 | ENSG00000214189 | 70.54 | -1.27 | 0.00 |
| ENSG00000174885 | 119.38 | 2.23 | 0.00 | ENSG00000145349 | 1643.45 | -1.27 | 0.00 |
| ENSG00000167880 | 4471.72 | 2.22 | 0.00 | ENSG00000119636 | 87.18 | -1.27 | 0.00 |
| ENSG00000094755 | 116.42 | 2.22 | 0.00 | ENSG00000105971 | 638.37 | -1.27 | 0.00 |
| ENSG00000249007 | 191.76 | 2.22 | 0.00 | ENSG00000126091 | 184.96 | -1.27 | 0.00 |
| ENSG00000204889 | 109.50 | 2.22 | 0.03 | ENSG00000178573 | 667.63 | -1.27 | 0.00 |
| ENSG00000183199 | 104.03 | 2.22 | 0.00 | ENSG00000179820 | 4627.66 | -1.28 | 0.00 |
| ENSG00000237361 | 167.68 | 2.22 | 0.00 | ENSG00000182195 | 122.64 | -1.28 | 0.00 |
| ENSG00000121716 | 250.73 | 2.22 | 0.00 | ENSG00000133424 | 887.73 | -1.28 | 0.00 |
| ENSG00000101057 | 5088.68 | 2.21 | 0.00 | ENSG00000172086 | 1501.11 | -1.28 | 0.00 |
| ENSG00000261236 | 5476.53 | 2.21 | 0.00 | ENSG00000126878 | 240.97 | -1.28 | 0.01 |
| ENSG00000228887 | 65.39 | 2.21 | 0.00 | ENSG00000118804 | 124.19 | -1.28 | 0.00 |
| ENSG00000164362 | 99.57 | 2.20 | 0.00 | ENSG00000117643 | 266.10 | -1.28 | 0.00 |
| ENSG00000146216 | 133.31 | 2.20 | 0.00 | ENSG00000139926 | 479.77 | -1.28 | 0.01 |
| ENSG00000099194 | 18361.32 | 2.20 | 0.00 | ENSG00000178562 | 66.48 | -1.28 | 0.00 |
| ENSG00000167702 | 962.31 | 2.20 | 0.00 | ENSG00000204472 | 351.41 | -1.28 | 0.00 |
| ENSG00000239345 | 50.28 | 2.20 | 0.00 | ENSG00000164023 | 946.17 | -1.28 | 0.00 |
| ENSG00000175602 | 1668.96 | 2.20 | 0.00 | ENSG00000179981 | 993.08 | -1.28 | 0.00 |
| ENSG00000140519 | 124.28 | 2.20 | 0.00 | ENSG00000198894 | 1015.10 | -1.28 | 0.00 |
| ENSG00000178752 | 53.49 | 2.20 | 0.00 | ENSG00000259674 | 69.02 | -1.28 | 0.01 |
| ENSG00000111701 | 169.93 | 2.19 | 0.00 | ENSG00000114450 | 380.85 | -1.29 | 0.00 |
| ENSG00000226887 | 102.88 | 2.19 | 0.00 | ENSG00000136002 | 180.90 | -1.29 | 0.04 |
| ENSG00000089327 | 4544.23 | 2.18 | 0.00 | ENSG00000103196 | 1416.97 | -1.29 | 0.00 |
| ENSG00000137203 | 142.69 | 2.18 | 0.00 | ENSG00000165807 | 118.72 | -1.29 | 0.00 |
| ENSG00000254206 | 51.16 | 2.18 | 0.00 | ENSG00000162897 | 78.38 | -1.29 | 0.01 |
| ENSG00000234449 | 65.23 | 2.18 | 0.01 | ENSG00000139725 | 188.07 | -1.29 | 0.00 |
| ENSG00000156510 | 2750.75 | 2.17 | 0.00 | ENSG00000203497 | 50.53 | -1.29 | 0.00 |
| ENSG00000197168 | 119.30 | 2.17 | 0.00 | ENSG00000162999 | 87.16 | -1.29 | 0.00 |
| ENSG00000176692 | 55.67 | 2.17 | 0.00 | ENSG00000167077 | 92.36 | -1.29 | 0.00 |
| ENSG00000146858 | 326.13 | 2.16 | 0.00 | ENSG00000187240 | 114.29 | -1.29 | 0.00 |
| ENSG00000253522 | 116.70 | 2.16 | 0.00 | ENSG00000188373 | 3324.59 | -1.29 | 0.01 |
| ENSG00000112655 | 3645.77 | 2.16 | 0.00 | ENSG00000196628 | 1018.21 | -1.29 | 0.00 |
| ENSG00000128564 | 143.98 | 2.16 | 0.00 | ENSG00000158828 | 889.42 | -1.29 | 0.00 |
| ENSG00000111199 | 93.72 | 2.16 | 0.00 | ENSG00000135144 | 131.24 | -1.29 | 0.00 |
| ENSG00000120254 | 1784.46 | 2.16 | 0.00 | ENSG00000166503 | 633.74 | -1.29 | 0.00 |
| ENSG00000123838 | 211.88 | 2.16 | 0.00 | ENSG00000114023 | 2556.12 | -1.29 | 0.00 |
| ENSG00000141449 | 52.90 | 2.15 | 0.03 | ENSG00000116667 | 1996.30 | -1.29 | 0.00 |
| ENSG00000204410 | 171.64 | 2.15 | 0.00 | ENSG00000058091 | 253.12 | -1.29 | 0.01 |
| ENSG00000226812 | 414.15 | 2.15 | 0.00 | ENSG00000184730 | 1849.68 | -1.30 | 0.00 |
| ENSG00000211448 | 498.95 | 2.15 | 0.00 | ENSG00000063180 | 121.48 | -1.30 | 0.00 |
| ENSG00000222033 | 107.60 | 2.15 | 0.00 | ENSG00000111961 | 628.20 | -1.30 | 0.00 |
| ENSG00000011201 | 307.02 | 2.15 | 0.00 | ENSG00000148842 | 619.45 | -1.30 | 0.00 |
| ENSG00000062282 | 1270.17 | 2.15 | 0.00 | ENSG00000088280 | 436.51 | -1.30 | 0.00 |
| ENSG00000133519 | 309.61 | 2.15 | 0.00 | ENSG00000119541 | 1848.42 | -1.30 | 0.00 |
| ENSG00000132031 | 118.44 | 2.15 | 0.00 | ENSG00000052795 | 1286.38 | -1.30 | 0.00 |
| ENSG00000272141 | 527.51 | 2.15 | 0.00 | ENSG00000204099 | 593.02 | -1.31 | 0.03 |
| ENSG00000226453 | 51.53 | 2.14 | 0.00 | ENSG00000070961 | 1874.28 | -1.31 | 0.00 |
| ENSG00000139629 | 3016.53 | 2.14 | 0.00 | ENSG00000137710 | 836.24 | -1.31 | 0.00 |
| ENSG00000172164 | 2470.83 | 2.14 | 0.00 | ENSG00000125868 | 15265.19 | -1.31 | 0.00 |
| ENSG00000111110 | 1709.86 | 2.14 | 0.00 | ENSG00000214376 | 76.43 | -1.31 | 0.01 |
| ENSG00000130054 | 144.69 | 2.14 | 0.01 | ENSG00000119782 | 80.28 | -1.31 | 0.01 |
| ENSG00000167992 | 68.68 | 2.14 | 0.00 | ENSG00000158301 | 171.76 | -1.31 | 0.00 |
| ENSG00000112877 | 368.89 | 2.14 | 0.00 | ENSG00000152642 | 2080.57 | -1.31 | 0.00 |
| ENSG00000081059 | 2364.95 | 2.13 | 0.00 | ENSG00000230630 | 55.43 | -1.31 | 0.00 |
| ENSG00000158352 | 1064.02 | 2.13 | 0.00 | ENSG00000163820 | 1623.83 | -1.31 | 0.00 |
| ENSG00000138074 | 4808.69 | 2.13 | 0.00 | ENSG00000165895 | 367.03 | -1.31 | 0.00 |
| ENSG00000162493 | 956.48 | 2.13 | 0.00 | ENSG00000110848 | 138.82 | -1.31 | 0.03 |
| ENSG00000273066 | 219.86 | 2.13 | 0.00 | ENSG00000176472 | 132.38 | -1.31 | 0.00 |
| ENSG00000157551 | 65.39 | 2.13 | 0.00 | ENSG00000203778 | 96.18 | -1.31 | 0.00 |
| ENSG00000089723 | 300.25 | 2.12 | 0.00 | ENSG00000272686 | 203.91 | -1.31 | 0.00 |
| ENSG00000144452 | 56.21 | 2.12 | 0.02 | ENSG00000169439 | 1391.55 | -1.31 | 0.00 |
| ENSG00000163013 | 779.59 | 2.11 | 0.00 | ENSG00000131831 | 312.70 | -1.31 | 0.00 |
| ENSG00000214078 | 9807.39 | 2.11 | 0.00 | ENSG00000136044 | 2125.98 | -1.31 | 0.00 |
| ENSG00000115255 | 964.81 | 2.11 | 0.00 | ENSG00000145649 | 159.66 | -1.32 | 0.00 |
| ENSG00000127564 | 1391.34 | 2.11 | 0.00 | ENSG00000055211 | 1105.88 | -1.32 | 0.00 |
| ENSG00000148735 | 1115.36 | 2.10 | 0.00 | ENSG00000115355 | 283.82 | -1.32 | 0.00 |
| ENSG00000182584 | 357.24 | 2.10 | 0.00 | ENSG00000183741 | 1175.33 | -1.32 | 0.00 |
| ENSG00000187908 | 12547.49 | 2.10 | 0.03 | ENSG00000171943 | 59.07 | -1.32 | 0.00 |
| ENSG00000280213 | 61.34 | 2.10 | 0.00 | ENSG00000176597 | 1083.40 | -1.32 | 0.00 |
| ENSG00000228205 | 162.83 | 2.10 | 0.00 | ENSG00000100842 | 132.41 | -1.32 | 0.00 |
| ENSG00000188372 | 556.31 | 2.09 | 0.00 | ENSG00000070759 | 279.84 | -1.32 | 0.00 |
| ENSG00000104415 | 265.20 | 2.09 | 0.00 | ENSG00000101298 | 149.43 | -1.32 | 0.00 |
| ENSG00000128965 | 382.89 | 2.09 | 0.00 | ENSG00000272462 | 92.40 | -1.32 | 0.00 |
| ENSG00000160401 | 135.02 | 2.09 | 0.00 | ENSG00000253313 | 910.07 | -1.32 | 0.00 |
| ENSG00000215182 | 682.35 | 2.08 | 0.02 | ENSG00000163531 | 542.57 | -1.32 | 0.00 |
| ENSG00000175063 | 3529.04 | 2.08 | 0.00 | ENSG00000080200 | 397.29 | -1.33 | 0.00 |
| ENSG00000196584 | 330.41 | 2.08 | 0.00 | ENSG00000126785 | 246.09 | -1.33 | 0.00 |
| ENSG00000104044 | 67.51 | 2.08 | 0.01 | ENSG00000077420 | 187.59 | -1.33 | 0.00 |
| ENSG00000127423 | 204.52 | 2.08 | 0.00 | ENSG00000187837 | 620.97 | -1.33 | 0.00 |
| ENSG00000128578 | 209.24 | 2.07 | 0.00 | ENSG00000069764 | 139.31 | -1.33 | 0.00 |
| ENSG00000021826 | 181.02 | 2.07 | 0.02 | ENSG00000100744 | 2447.63 | -1.33 | 0.00 |
| ENSG00000011347 | 2962.55 | 2.07 | 0.00 | ENSG00000151414 | 1761.07 | -1.33 | 0.00 |
| ENSG00000177398 | 53.58 | 2.07 | 0.00 | ENSG00000127954 | 158.27 | -1.33 | 0.00 |
| ENSG00000254389 | 57.84 | 2.07 | 0.00 | ENSG00000111696 | 965.32 | -1.33 | 0.00 |
| ENSG00000255874 | 157.08 | 2.07 | 0.00 | ENSG00000105755 | 5310.78 | -1.33 | 0.00 |
| ENSG00000136997 | 11618.32 | 2.06 | 0.00 | ENSG00000111554 | 276.24 | -1.34 | 0.00 |
| ENSG00000167393 | 701.90 | 2.06 | 0.00 | ENSG00000136235 | 2561.79 | -1.34 | 0.01 |
| ENSG00000078814 | 181.02 | 2.06 | 0.00 | ENSG00000101596 | 1249.71 | -1.34 | 0.00 |
| ENSG00000165480 | 1000.21 | 2.06 | 0.00 | ENSG00000188993 | 255.77 | -1.34 | 0.00 |
| ENSG00000182472 | 207.74 | 2.06 | 0.00 | ENSG00000180190 | 118.97 | -1.34 | 0.01 |
| ENSG00000025772 | 3752.90 | 2.06 | 0.00 | ENSG00000136160 | 413.76 | -1.34 | 0.00 |
| ENSG00000069011 | 1945.32 | 2.06 | 0.00 | ENSG00000177707 | 853.81 | -1.34 | 0.00 |
| ENSG00000124743 | 64.84 | 2.05 | 0.00 | ENSG00000172869 | 754.46 | -1.34 | 0.00 |
| ENSG00000256940 | 61.78 | 2.05 | 0.00 | ENSG00000263155 | 141.46 | -1.35 | 0.00 |
| ENSG00000140093 | 93.61 | 2.05 | 0.02 | ENSG00000092841 | 29618.41 | -1.35 | 0.00 |
| ENSG00000204866 | 58.70 | 2.05 | 0.01 | ENSG00000198513 | 201.58 | -1.35 | 0.00 |
| ENSG00000100625 | 52.74 | 2.05 | 0.00 | ENSG00000137502 | 409.23 | -1.35 | 0.00 |
| ENSG00000243479 | 163.08 | 2.04 | 0.00 | ENSG00000156535 | 365.53 | -1.35 | 0.01 |
| ENSG00000088305 | 261.04 | 2.04 | 0.00 | ENSG00000204381 | 190.40 | -1.35 | 0.00 |
| ENSG00000125378 | 3274.47 | 2.04 | 0.00 | ENSG00000152601 | 3836.53 | -1.35 | 0.00 |
| ENSG00000179603 | 564.44 | 2.04 | 0.00 | ENSG00000091157 | 401.96 | -1.35 | 0.00 |
| ENSG00000086991 | 84.41 | 2.04 | 0.00 | ENSG00000174125 | 111.01 | -1.35 | 0.00 |
| ENSG00000184428 | 2768.96 | 2.04 | 0.00 | ENSG00000141404 | 198.19 | -1.35 | 0.00 |
| ENSG00000258429 | 254.40 | 2.04 | 0.00 | ENSG00000134245 | 247.60 | -1.35 | 0.00 |
| ENSG00000281398 | 210.00 | 2.04 | 0.00 | ENSG00000155846 | 629.13 | -1.35 | 0.00 |
| ENSG00000125398 | 8697.05 | 2.03 | 0.00 | ENSG00000002726 | 9449.34 | -1.35 | 0.00 |
| ENSG00000178719 | 10469.45 | 2.03 | 0.00 | ENSG00000112245 | 7161.94 | -1.35 | 0.00 |
| ENSG00000188290 | 398.86 | 2.03 | 0.00 | ENSG00000100784 | 395.22 | -1.35 | 0.00 |
| ENSG00000108106 | 1646.89 | 2.03 | 0.00 | ENSG00000065413 | 233.35 | -1.35 | 0.00 |
| ENSG00000130701 | 679.26 | 2.03 | 0.00 | ENSG00000170954 | 131.07 | -1.35 | 0.01 |
| ENSG00000136883 | 1339.73 | 2.03 | 0.00 | ENSG00000153234 | 599.36 | -1.36 | 0.00 |
| ENSG00000138795 | 534.68 | 2.03 | 0.00 | ENSG00000109458 | 926.69 | -1.36 | 0.00 |
| ENSG00000262001 | 413.62 | 2.03 | 0.00 | ENSG00000116574 | 1241.43 | -1.36 | 0.00 |
| ENSG00000166123 | 3065.05 | 2.02 | 0.00 | ENSG00000121858 | 2348.60 | -1.36 | 0.00 |
| ENSG00000111012 | 136.54 | 2.02 | 0.00 | ENSG00000176014 | 1187.31 | -1.36 | 0.00 |
| ENSG00000182580 | 7816.67 | 2.02 | 0.00 | ENSG00000204370 | 2607.67 | -1.36 | 0.00 |
| ENSG00000174137 | 75.91 | 2.02 | 0.00 | ENSG00000107864 | 171.50 | -1.36 | 0.00 |
| ENSG00000146677 | 219.96 | 2.02 | 0.00 | ENSG00000160593 | 293.24 | -1.36 | 0.00 |
| ENSG00000167280 | 3231.35 | 2.02 | 0.00 | ENSG00000213713 | 51.63 | -1.36 | 0.00 |
| ENSG00000135253 | 110.63 | 2.02 | 0.00 | ENSG00000163898 | 2124.96 | -1.37 | 0.00 |
| ENSG00000122861 | 2355.14 | 2.01 | 0.00 | ENSG00000184113 | 364.24 | -1.37 | 0.00 |
| ENSG00000169245 | 646.31 | 2.01 | 0.00 | ENSG00000072858 | 513.90 | -1.37 | 0.00 |
| ENSG00000135119 | 150.32 | 2.01 | 0.00 | ENSG00000141655 | 1219.38 | -1.37 | 0.00 |
| ENSG00000237857 | 91.91 | 2.00 | 0.00 | ENSG00000111077 | 1246.13 | -1.37 | 0.00 |
| ENSG00000176170 | 466.19 | 2.00 | 0.00 | ENSG00000075239 | 1907.30 | -1.37 | 0.00 |
| ENSG00000197046 | 73.99 | 2.00 | 0.00 | ENSG00000048342 | 281.34 | -1.37 | 0.00 |
| ENSG00000224411 | 51.98 | 2.00 | 0.00 | ENSG00000213190 | 130.41 | -1.37 | 0.00 |
| ENSG00000161179 | 1812.14 | 2.00 | 0.00 | ENSG00000144369 | 111.97 | -1.37 | 0.00 |
| ENSG00000262580 | 129.06 | 2.00 | 0.00 | ENSG00000184012 | 7556.83 | -1.37 | 0.00 |
| ENSG00000135451 | 984.77 | 2.00 | 0.00 | ENSG00000183111 | 462.81 | -1.37 | 0.00 |
| ENSG00000130590 | 685.92 | 2.00 | 0.00 | ENSG00000121933 | 82.85 | -1.37 | 0.00 |
| ENSG00000167711 | 270.78 | 2.00 | 0.00 | ENSG00000211677 | 4401.43 | -1.37 | 0.04 |
| ENSG00000124875 | 133.23 | 1.99 | 0.00 | ENSG00000165731 | 118.97 | -1.37 | 0.03 |
| ENSG00000186648 | 129.09 | 1.99 | 0.00 | ENSG00000165806 | 2156.81 | -1.37 | 0.00 |
| ENSG00000103569 | 161.04 | 1.99 | 0.00 | ENSG00000157168 | 116.77 | -1.37 | 0.01 |
| ENSG00000136295 | 8025.76 | 1.99 | 0.00 | ENSG00000061918 | 370.43 | -1.37 | 0.00 |
| ENSG00000127586 | 1257.67 | 1.98 | 0.00 | ENSG00000088827 | 257.61 | -1.37 | 0.01 |
| ENSG00000186340 | 5139.58 | 1.98 | 0.00 | ENSG00000165269 | 102.41 | -1.38 | 0.00 |
| ENSG00000124216 | 267.99 | 1.98 | 0.00 | ENSG00000134321 | 361.29 | -1.38 | 0.01 |
| ENSG00000169583 | 365.62 | 1.98 | 0.00 | ENSG00000188536 | 160.30 | -1.38 | 0.02 |
| ENSG00000144395 | 91.82 | 1.98 | 0.00 | ENSG00000124406 | 996.97 | -1.38 | 0.00 |
| ENSG00000143217 | 710.82 | 1.97 | 0.00 | ENSG00000250303 | 70.56 | -1.38 | 0.00 |
| ENSG00000179772 | 85.84 | 1.97 | 0.00 | ENSG00000174080 | 680.70 | -1.38 | 0.00 |
| ENSG00000104313 | 72.26 | 1.97 | 0.02 | ENSG00000197535 | 358.72 | -1.38 | 0.00 |
| ENSG00000226958 | 72.27 | 1.96 | 0.00 | ENSG00000003137 | 97.12 | -1.38 | 0.00 |
| ENSG00000162073 | 1255.10 | 1.96 | 0.00 | ENSG00000164938 | 712.84 | -1.38 | 0.00 |
| ENSG00000187961 | 301.15 | 1.96 | 0.00 | ENSG00000137767 | 2931.78 | -1.38 | 0.00 |
| ENSG00000106133 | 70.34 | 1.96 | 0.00 | ENSG00000169442 | 371.74 | -1.38 | 0.00 |
| ENSG00000159423 | 1079.35 | 1.96 | 0.00 | ENSG00000172296 | 160.45 | -1.38 | 0.01 |
| ENSG00000184497 | 387.13 | 1.96 | 0.00 | ENSG00000135773 | 333.49 | -1.38 | 0.05 |
| ENSG00000278291 | 183.15 | 1.96 | 0.00 | ENSG00000123360 | 63.10 | -1.38 | 0.00 |
| ENSG00000183010 | 4629.56 | 1.96 | 0.00 | ENSG00000151702 | 282.61 | -1.38 | 0.00 |
| ENSG00000186193 | 3076.18 | 1.95 | 0.00 | ENSG00000271601 | 486.11 | -1.38 | 0.00 |
| ENSG00000161996 | 2054.47 | 1.95 | 0.00 | ENSG00000172543 | 81.66 | -1.39 | 0.00 |
| ENSG00000123989 | 6157.44 | 1.95 | 0.00 | ENSG00000205213 | 2487.11 | -1.39 | 0.00 |
| ENSG00000160867 | 5220.24 | 1.94 | 0.00 | ENSG00000133574 | 406.79 | -1.39 | 0.00 |
| ENSG00000091651 | 554.82 | 1.94 | 0.00 | ENSG00000255302 | 2836.72 | -1.39 | 0.00 |
| ENSG00000102265 | 10760.57 | 1.94 | 0.00 | ENSG00000120756 | 6378.12 | -1.39 | 0.00 |
| ENSG00000167747 | 4486.76 | 1.94 | 0.00 | ENSG00000137819 | 743.96 | -1.39 | 0.00 |
| ENSG00000184949 | 85.79 | 1.94 | 0.00 | ENSG00000116704 | 2261.06 | -1.39 | 0.00 |
| ENSG00000142089 | 19923.15 | 1.94 | 0.00 | ENSG00000169504 | 3740.09 | -1.39 | 0.00 |
| ENSG00000171163 | 914.48 | 1.93 | 0.00 | ENSG00000170677 | 968.37 | -1.39 | 0.00 |
| ENSG00000124225 | 7906.48 | 1.93 | 0.00 | ENSG00000164949 | 792.01 | -1.39 | 0.00 |
| ENSG00000159167 | 532.05 | 1.92 | 0.00 | ENSG00000220205 | 1185.79 | -1.39 | 0.00 |
| ENSG00000262251 | 52.73 | 1.92 | 0.00 | ENSG00000076662 | 55.80 | -1.39 | 0.00 |
| ENSG00000267523 | 73.57 | 1.92 | 0.00 | ENSG00000053254 | 1603.04 | -1.39 | 0.00 |
| ENSG00000172061 | 512.68 | 1.92 | 0.00 | ENSG00000130592 | 1095.26 | -1.39 | 0.00 |
| ENSG00000183856 | 2550.95 | 1.92 | 0.00 | ENSG00000106688 | 587.61 | -1.39 | 0.00 |
| ENSG00000147804 | 4977.92 | 1.92 | 0.00 | ENSG00000175329 | 986.22 | -1.40 | 0.01 |
| ENSG00000179869 | 57.00 | 1.92 | 0.00 | ENSG00000148175 | 2295.99 | -1.40 | 0.00 |
| ENSG00000182557 | 128.19 | 1.92 | 0.00 | ENSG00000136999 | 186.12 | -1.40 | 0.01 |
| ENSG00000069482 | 587.37 | 1.92 | 0.00 | ENSG00000130844 | 279.01 | -1.40 | 0.00 |
| ENSG00000139289 | 5189.65 | 1.92 | 0.00 | ENSG00000171033 | 138.41 | -1.40 | 0.01 |
| ENSG00000137809 | 1132.59 | 1.92 | 0.00 | ENSG00000137449 | 571.90 | -1.40 | 0.00 |
| ENSG00000120694 | 8718.51 | 1.92 | 0.00 | ENSG00000131196 | 222.49 | -1.40 | 0.00 |
| ENSG00000078114 | 3524.29 | 1.92 | 0.00 | ENSG00000151729 | 1254.95 | -1.40 | 0.00 |
| ENSG00000215458 | 59.83 | 1.91 | 0.00 | ENSG00000167601 | 1111.18 | -1.40 | 0.00 |
| ENSG00000103269 | 243.69 | 1.91 | 0.00 | ENSG00000128266 | 165.16 | -1.40 | 0.00 |
| ENSG00000120875 | 1033.76 | 1.91 | 0.00 | ENSG00000164251 | 3182.09 | -1.40 | 0.00 |
| ENSG00000248635 | 54.41 | 1.91 | 0.01 | ENSG00000106538 | 1991.82 | -1.41 | 0.00 |
| ENSG00000225614 | 299.42 | 1.91 | 0.00 | ENSG00000104814 | 150.46 | -1.41 | 0.00 |
| ENSG00000161091 | 2441.15 | 1.91 | 0.00 | ENSG00000205277 | 12509.78 | -1.41 | 0.03 |
| ENSG00000102048 | 349.00 | 1.91 | 0.00 | ENSG00000254122 | 84.94 | -1.41 | 0.00 |
| ENSG00000139618 | 558.79 | 1.90 | 0.00 | ENSG00000163069 | 1142.11 | -1.41 | 0.00 |
| ENSG00000123843 | 246.48 | 1.90 | 0.00 | ENSG00000166866 | 3072.12 | -1.41 | 0.00 |
| ENSG00000186185 | 855.87 | 1.90 | 0.00 | ENSG00000185156 | 130.21 | -1.41 | 0.00 |
| ENSG00000167550 | 75.22 | 1.90 | 0.00 | ENSG00000165092 | 2897.83 | -1.41 | 0.02 |
| ENSG00000088325 | 5113.11 | 1.90 | 0.00 | ENSG00000164741 | 617.87 | -1.41 | 0.00 |
| ENSG00000115008 | 67.90 | 1.89 | 0.00 | ENSG00000171428 | 301.04 | -1.41 | 0.00 |
| ENSG00000141570 | 544.20 | 1.88 | 0.00 | ENSG00000077943 | 192.56 | -1.41 | 0.00 |
| ENSG00000102445 | 4162.62 | 1.88 | 0.00 | ENSG00000143344 | 519.05 | -1.41 | 0.00 |
| ENSG00000124882 | 2212.43 | 1.88 | 0.00 | ENSG00000151914 | 3481.87 | -1.42 | 0.00 |
| ENSG00000160932 | 5275.91 | 1.88 | 0.00 | ENSG00000093072 | 971.35 | -1.42 | 0.00 |
| ENSG00000172216 | 2971.19 | 1.88 | 0.00 | ENSG00000049759 | 2321.06 | -1.42 | 0.00 |
| ENSG00000138316 | 362.26 | 1.88 | 0.00 | ENSG00000117054 | 1328.62 | -1.42 | 0.00 |
| ENSG00000124766 | 5782.74 | 1.88 | 0.00 | ENSG00000099260 | 134.78 | -1.42 | 0.00 |
| ENSG00000217130 | 66.35 | 1.87 | 0.00 | ENSG00000120162 | 1129.42 | -1.42 | 0.00 |
| ENSG00000117707 | 1434.73 | 1.87 | 0.00 | ENSG00000138593 | 975.97 | -1.42 | 0.00 |
| ENSG00000185101 | 5796.34 | 1.87 | 0.00 | ENSG00000147852 | 277.31 | -1.42 | 0.00 |
| ENSG00000175426 | 901.50 | 1.87 | 0.04 | ENSG00000196730 | 610.11 | -1.42 | 0.00 |
| ENSG00000101412 | 1318.50 | 1.87 | 0.00 | ENSG00000151067 | 708.63 | -1.42 | 0.00 |
| ENSG00000104524 | 1390.48 | 1.87 | 0.00 | ENSG00000102886 | 350.53 | -1.42 | 0.00 |
| ENSG00000272235 | 51.31 | 1.87 | 0.00 | ENSG00000133808 | 119.24 | -1.42 | 0.00 |
| ENSG00000078900 | 150.53 | 1.87 | 0.00 | ENSG00000114541 | 560.28 | -1.42 | 0.00 |
| ENSG00000071539 | 979.26 | 1.86 | 0.00 | ENSG00000047648 | 261.83 | -1.43 | 0.01 |
| ENSG00000226419 | 124.53 | 1.86 | 0.00 | ENSG00000162407 | 1236.75 | -1.43 | 0.00 |
| ENSG00000123496 | 51.87 | 1.86 | 0.00 | ENSG00000168421 | 150.21 | -1.43 | 0.00 |
| ENSG00000213903 | 286.51 | 1.86 | 0.00 | ENSG00000188153 | 148.06 | -1.43 | 0.00 |
| ENSG00000172965 | 544.95 | 1.86 | 0.00 | ENSG00000172955 | 242.11 | -1.43 | 0.01 |
| ENSG00000167914 | 91.22 | 1.86 | 0.00 | ENSG00000188404 | 211.09 | -1.43 | 0.00 |
| ENSG00000269821 | 230.87 | 1.86 | 0.00 | ENSG00000114948 | 70.40 | -1.43 | 0.00 |
| ENSG00000214110 | 180.80 | 1.85 | 0.00 | ENSG00000134853 | 1205.22 | -1.43 | 0.00 |
| ENSG00000180785 | 123.55 | 1.85 | 0.00 | ENSG00000144815 | 228.31 | -1.44 | 0.00 |
| ENSG00000236017 | 106.61 | 1.85 | 0.00 | ENSG00000104213 | 80.48 | -1.44 | 0.02 |
| ENSG00000249395 | 226.97 | 1.84 | 0.00 | ENSG00000035403 | 6052.42 | -1.44 | 0.00 |
| ENSG00000260942 | 103.42 | 1.84 | 0.00 | ENSG00000167741 | 2285.84 | -1.44 | 0.00 |
| ENSG00000130193 | 3411.31 | 1.84 | 0.00 | ENSG00000197852 | 181.88 | -1.44 | 0.00 |
| ENSG00000179241 | 620.61 | 1.84 | 0.00 | ENSG00000155324 | 716.65 | -1.44 | 0.00 |
| ENSG00000075702 | 640.66 | 1.84 | 0.00 | ENSG00000131849 | 61.24 | -1.44 | 0.00 |
| ENSG00000141756 | 4119.09 | 1.84 | 0.00 | ENSG00000100739 | 68.36 | -1.44 | 0.00 |
| ENSG00000279605 | 58.68 | 1.84 | 0.00 | ENSG00000156050 | 120.08 | -1.44 | 0.00 |
| ENSG00000277363 | 476.34 | 1.84 | 0.00 | ENSG00000137501 | 2129.03 | -1.44 | 0.00 |
| ENSG00000129295 | 203.18 | 1.84 | 0.00 | ENSG00000120915 | 1465.91 | -1.45 | 0.00 |
| ENSG00000198734 | 254.74 | 1.84 | 0.01 | ENSG00000079257 | 547.92 | -1.45 | 0.00 |
| ENSG00000162062 | 336.15 | 1.83 | 0.00 | ENSG00000135272 | 452.29 | -1.45 | 0.00 |
| ENSG00000106178 | 1565.32 | 1.83 | 0.00 | ENSG00000143001 | 51.30 | -1.45 | 0.02 |
| ENSG00000106927 | 51.14 | 1.83 | 0.00 | ENSG00000103175 | 480.77 | -1.45 | 0.00 |
| ENSG00000243649 | 668.04 | 1.83 | 0.00 | ENSG00000275993 | 165.72 | -1.45 | 0.00 |
| ENSG00000115257 | 147.62 | 1.83 | 0.00 | ENSG00000128606 | 77.95 | -1.45 | 0.00 |
| ENSG00000160796 | 5882.28 | 1.83 | 0.00 | ENSG00000064652 | 179.99 | -1.46 | 0.00 |
| ENSG00000108773 | 3406.24 | 1.83 | 0.00 | ENSG00000250510 | 58.13 | -1.46 | 0.00 |
| ENSG00000163975 | 2107.81 | 1.82 | 0.00 | ENSG00000088386 | 220.72 | -1.46 | 0.03 |
| ENSG00000141391 | 132.67 | 1.82 | 0.00 | ENSG00000171100 | 821.66 | -1.46 | 0.00 |
| ENSG00000117877 | 335.78 | 1.82 | 0.00 | ENSG00000219481 | 434.28 | -1.46 | 0.00 |
| ENSG00000118707 | 1646.05 | 1.82 | 0.00 | ENSG00000024422 | 1957.25 | -1.46 | 0.00 |
| ENSG00000181218 | 562.69 | 1.82 | 0.00 | ENSG00000134516 | 376.32 | -1.46 | 0.00 |
| ENSG00000262406 | 1869.33 | 1.81 | 0.00 | ENSG00000170558 | 83.45 | -1.46 | 0.02 |
| ENSG00000130589 | 3915.97 | 1.81 | 0.00 | ENSG00000114248 | 522.56 | -1.46 | 0.00 |
| ENSG00000163191 | 15551.28 | 1.81 | 0.00 | ENSG00000074755 | 2384.04 | -1.46 | 0.00 |
| ENSG00000181392 | 440.24 | 1.81 | 0.00 | ENSG00000167261 | 57.25 | -1.46 | 0.00 |
| ENSG00000260005 | 57.63 | 1.81 | 0.00 | ENSG00000122420 | 71.33 | -1.46 | 0.00 |
| ENSG00000198542 | 233.25 | 1.81 | 0.01 | ENSG00000170345 | 10363.01 | -1.46 | 0.00 |
| ENSG00000174567 | 464.77 | 1.80 | 0.00 | ENSG00000120156 | 225.89 | -1.46 | 0.00 |
| ENSG00000224397 | 135.31 | 1.80 | 0.00 | ENSG00000104447 | 184.85 | -1.46 | 0.00 |
| ENSG00000129667 | 1742.00 | 1.80 | 0.00 | ENSG00000112303 | 79.54 | -1.46 | 0.02 |
| ENSG00000226287 | 218.21 | 1.80 | 0.00 | ENSG00000162616 | 355.55 | -1.46 | 0.00 |
| ENSG00000205622 | 139.67 | 1.80 | 0.00 | ENSG00000157514 | 1685.95 | -1.47 | 0.00 |
| ENSG00000101333 | 5481.60 | 1.80 | 0.00 | ENSG00000104643 | 304.05 | -1.47 | 0.00 |
| ENSG00000140451 | 305.33 | 1.80 | 0.00 | ENSG00000112818 | 4859.18 | -1.47 | 0.00 |
| ENSG00000134668 | 90.73 | 1.80 | 0.00 | ENSG00000085563 | 1472.93 | -1.47 | 0.01 |
| ENSG00000171617 | 5591.91 | 1.80 | 0.00 | ENSG00000166165 | 28410.66 | -1.47 | 0.01 |
| ENSG00000180389 | 54.16 | 1.79 | 0.00 | ENSG00000123338 | 546.57 | -1.47 | 0.00 |
| ENSG00000261971 | 187.18 | 1.79 | 0.00 | ENSG00000134955 | 281.62 | -1.47 | 0.00 |
| ENSG00000089351 | 3375.74 | 1.79 | 0.00 | ENSG00000136052 | 567.44 | -1.47 | 0.00 |
| ENSG00000132003 | 729.95 | 1.79 | 0.00 | ENSG00000167081 | 472.20 | -1.47 | 0.00 |
| ENSG00000128342 | 1197.80 | 1.79 | 0.00 | ENSG00000143382 | 269.58 | -1.47 | 0.00 |
| ENSG00000075275 | 1615.92 | 1.79 | 0.00 | ENSG00000077942 | 4688.70 | -1.47 | 0.00 |
| ENSG00000206075 | 2025.03 | 1.79 | 0.00 | ENSG00000031003 | 629.29 | -1.47 | 0.00 |
| ENSG00000185633 | 511.99 | 1.79 | 0.00 | ENSG00000185010 | 177.88 | -1.47 | 0.00 |
| ENSG00000168268 | 2889.63 | 1.79 | 0.00 | ENSG00000117592 | 7227.54 | -1.48 | 0.00 |
| ENSG00000185338 | 335.41 | 1.79 | 0.00 | ENSG00000224259 | 527.15 | -1.48 | 0.00 |
| ENSG00000276043 | 892.01 | 1.79 | 0.00 | ENSG00000156006 | 178.11 | -1.48 | 0.00 |
| ENSG00000141582 | 1554.05 | 1.79 | 0.00 | ENSG00000171115 | 222.79 | -1.48 | 0.00 |
| ENSG00000214796 | 327.32 | 1.78 | 0.00 | ENSG00000165795 | 2761.95 | -1.48 | 0.00 |
| ENSG00000134815 | 1626.95 | 1.78 | 0.00 | ENSG00000161929 | 83.69 | -1.48 | 0.00 |
| ENSG00000198208 | 393.01 | 1.78 | 0.00 | ENSG00000060762 | 1219.28 | -1.48 | 0.00 |
| ENSG00000255182 | 79.55 | 1.78 | 0.00 | ENSG00000176894 | 573.73 | -1.48 | 0.00 |
| ENSG00000010030 | 442.24 | 1.78 | 0.00 | ENSG00000102349 | 66.07 | -1.49 | 0.00 |
| ENSG00000070882 | 1458.60 | 1.78 | 0.00 | ENSG00000146966 | 368.23 | -1.49 | 0.00 |
| ENSG00000185838 | 431.11 | 1.78 | 0.00 | ENSG00000106823 | 151.76 | -1.49 | 0.00 |
| ENSG00000053108 | 53.41 | 1.78 | 0.03 | ENSG00000204361 | 86.88 | -1.49 | 0.02 |
| ENSG00000140511 | 464.88 | 1.78 | 0.00 | ENSG00000165633 | 327.09 | -1.49 | 0.00 |
| ENSG00000227719 | 55.25 | 1.78 | 0.00 | ENSG00000136960 | 612.44 | -1.49 | 0.00 |
| ENSG00000173535 | 109.51 | 1.77 | 0.00 | ENSG00000146555 | 221.27 | -1.49 | 0.00 |
| ENSG00000181634 | 557.20 | 1.77 | 0.00 | ENSG00000162772 | 1710.59 | -1.49 | 0.00 |
| ENSG00000119630 | 406.49 | 1.77 | 0.00 | ENSG00000116991 | 1314.09 | -1.49 | 0.00 |
| ENSG00000228109 | 91.95 | 1.77 | 0.00 | ENSG00000163596 | 70.92 | -1.50 | 0.00 |
| ENSG00000146477 | 865.53 | 1.77 | 0.00 | ENSG00000152503 | 249.54 | -1.50 | 0.00 |
| ENSG00000117394 | 11736.82 | 1.77 | 0.00 | ENSG00000137070 | 244.17 | -1.50 | 0.00 |
| ENSG00000108511 | 1142.63 | 1.77 | 0.00 | ENSG00000121413 | 320.26 | -1.50 | 0.00 |
| ENSG00000163737 | 75.42 | 1.77 | 0.01 | ENSG00000099834 | 5305.02 | -1.50 | 0.00 |
| ENSG00000198732 | 340.50 | 1.77 | 0.01 | ENSG00000149582 | 161.02 | -1.50 | 0.00 |
| ENSG00000049192 | 75.67 | 1.77 | 0.00 | ENSG00000196196 | 352.10 | -1.50 | 0.00 |
| ENSG00000182325 | 2253.23 | 1.77 | 0.00 | ENSG00000111452 | 140.55 | -1.50 | 0.01 |
| ENSG00000157388 | 645.95 | 1.77 | 0.00 | ENSG00000102038 | 392.26 | -1.50 | 0.00 |
| ENSG00000163993 | 8881.42 | 1.76 | 0.00 | ENSG00000187091 | 758.15 | -1.50 | 0.00 |
| ENSG00000160949 | 1503.21 | 1.76 | 0.00 | ENSG00000167693 | 576.83 | -1.50 | 0.00 |
| ENSG00000230461 | 50.85 | 1.76 | 0.00 | ENSG00000120833 | 378.69 | -1.50 | 0.00 |
| ENSG00000258839 | 135.31 | 1.76 | 0.00 | ENSG00000149289 | 357.47 | -1.50 | 0.00 |
| ENSG00000186529 | 799.68 | 1.75 | 0.00 | ENSG00000114853 | 447.37 | -1.51 | 0.00 |
| ENSG00000196950 | 1381.48 | 1.75 | 0.00 | ENSG00000181826 | 126.24 | -1.51 | 0.00 |
| ENSG00000268756 | 57.96 | 1.75 | 0.01 | ENSG00000166333 | 459.55 | -1.51 | 0.00 |
| ENSG00000106366 | 1566.76 | 1.75 | 0.00 | ENSG00000162630 | 51.46 | -1.51 | 0.02 |
| ENSG00000101470 | 641.26 | 1.75 | 0.00 | ENSG00000241106 | 55.12 | -1.51 | 0.00 |
| ENSG00000235173 | 2147.67 | 1.75 | 0.00 | ENSG00000132718 | 403.81 | -1.51 | 0.00 |
| ENSG00000218227 | 161.42 | 1.75 | 0.00 | ENSG00000181631 | 77.67 | -1.51 | 0.00 |
| ENSG00000160606 | 668.03 | 1.75 | 0.00 | ENSG00000003989 | 165.42 | -1.51 | 0.01 |
| ENSG00000105173 | 520.49 | 1.75 | 0.00 | ENSG00000154917 | 152.74 | -1.51 | 0.00 |
| ENSG00000251562 | 7569.32 | 1.75 | 0.00 | ENSG00000204161 | 170.97 | -1.51 | 0.00 |
| ENSG00000134013 | 2444.22 | 1.75 | 0.00 | ENSG00000143772 | 983.81 | -1.51 | 0.00 |
| ENSG00000188322 | 262.97 | 1.75 | 0.00 | ENSG00000042980 | 207.55 | -1.52 | 0.00 |
| ENSG00000061337 | 198.80 | 1.75 | 0.00 | ENSG00000077782 | 1232.87 | -1.52 | 0.00 |
| ENSG00000079462 | 1988.35 | 1.75 | 0.00 | ENSG00000134504 | 133.47 | -1.52 | 0.00 |
| ENSG00000092621 | 2611.20 | 1.75 | 0.00 | ENSG00000155659 | 366.46 | -1.52 | 0.00 |
| ENSG00000115457 | 6757.08 | 1.74 | 0.00 | ENSG00000167748 | 1494.54 | -1.52 | 0.00 |
| ENSG00000140279 | 5802.60 | 1.74 | 0.01 | ENSG00000173376 | 229.54 | -1.52 | 0.00 |
| ENSG00000011426 | 1899.38 | 1.74 | 0.00 | ENSG00000135905 | 261.93 | -1.52 | 0.00 |
| ENSG00000167207 | 261.34 | 1.74 | 0.00 | ENSG00000129538 | 4356.68 | -1.52 | 0.00 |
| ENSG00000101444 | 21160.78 | 1.74 | 0.00 | ENSG00000050405 | 5102.07 | -1.52 | 0.00 |
| ENSG00000112715 | 6561.23 | 1.74 | 0.00 | ENSG00000198721 | 827.80 | -1.52 | 0.00 |
| ENSG00000074211 | 240.41 | 1.74 | 0.03 | ENSG00000137285 | 94.39 | -1.52 | 0.02 |
| ENSG00000074181 | 3061.02 | 1.74 | 0.00 | ENSG00000119514 | 1541.10 | -1.52 | 0.00 |
| ENSG00000188368 | 57.58 | 1.74 | 0.00 | ENSG00000170624 | 273.34 | -1.53 | 0.00 |
| ENSG00000258498 | 880.12 | 1.74 | 0.00 | ENSG00000133561 | 372.24 | -1.53 | 0.00 |
| ENSG00000272645 | 147.57 | 1.74 | 0.00 | ENSG00000120129 | 4230.13 | -1.53 | 0.00 |
| ENSG00000260807 | 160.53 | 1.74 | 0.02 | ENSG00000122986 | 105.89 | -1.53 | 0.00 |
| ENSG00000163689 | 191.80 | 1.73 | 0.00 | ENSG00000089041 | 89.21 | -1.53 | 0.00 |
| ENSG00000165891 | 260.22 | 1.73 | 0.00 | ENSG00000151338 | 125.45 | -1.53 | 0.00 |
| ENSG00000196187 | 8490.62 | 1.73 | 0.00 | ENSG00000104936 | 1099.55 | -1.53 | 0.00 |
| ENSG00000167513 | 2027.59 | 1.73 | 0.00 | ENSG00000172794 | 185.54 | -1.53 | 0.00 |
| ENSG00000164761 | 705.22 | 1.73 | 0.00 | ENSG00000089356 | 20969.45 | -1.54 | 0.00 |
| ENSG00000122034 | 8030.32 | 1.73 | 0.00 | ENSG00000088543 | 112.68 | -1.54 | 0.00 |
| ENSG00000101189 | 1647.91 | 1.73 | 0.00 | ENSG00000144366 | 265.16 | -1.54 | 0.00 |
| ENSG00000154920 | 158.26 | 1.72 | 0.00 | ENSG00000120279 | 132.27 | -1.54 | 0.00 |
| ENSG00000160193 | 897.40 | 1.72 | 0.00 | ENSG00000105650 | 370.66 | -1.54 | 0.00 |
| ENSG00000108342 | 97.96 | 1.72 | 0.01 | ENSG00000110324 | 482.75 | -1.54 | 0.00 |
| ENSG00000151790 | 191.05 | 1.72 | 0.01 | ENSG00000070778 | 573.86 | -1.54 | 0.00 |
| ENSG00000254815 | 55.00 | 1.72 | 0.00 | ENSG00000130413 | 55.81 | -1.54 | 0.03 |
| ENSG00000118557 | 124.13 | 1.72 | 0.00 | ENSG00000165424 | 1038.28 | -1.54 | 0.00 |
| ENSG00000234062 | 88.66 | 1.72 | 0.00 | ENSG00000198453 | 68.03 | -1.55 | 0.00 |
| ENSG00000124731 | 117.12 | 1.71 | 0.00 | ENSG00000112531 | 922.87 | -1.55 | 0.00 |
| ENSG00000142530 | 130.62 | 1.71 | 0.00 | ENSG00000159184 | 2967.95 | -1.55 | 0.02 |
| ENSG00000130475 | 719.64 | 1.71 | 0.00 | ENSG00000164035 | 200.68 | -1.55 | 0.00 |
| ENSG00000107719 | 1817.27 | 1.71 | 0.00 | ENSG00000177932 | 56.23 | -1.55 | 0.00 |
| ENSG00000175711 | 265.73 | 1.71 | 0.00 | ENSG00000198160 | 1440.59 | -1.55 | 0.00 |
| ENSG00000083807 | 375.46 | 1.71 | 0.00 | ENSG00000112799 | 82.73 | -1.55 | 0.00 |
| ENSG00000183018 | 2371.18 | 1.71 | 0.00 | ENSG00000105290 | 60.30 | -1.55 | 0.00 |
| ENSG00000101158 | 5991.40 | 1.71 | 0.00 | ENSG00000179841 | 210.18 | -1.55 | 0.00 |
| ENSG00000234782 | 84.20 | 1.71 | 0.00 | ENSG00000079263 | 76.43 | -1.55 | 0.00 |
| ENSG00000173638 | 1518.01 | 1.70 | 0.00 | ENSG00000166959 | 227.03 | -1.55 | 0.02 |
| ENSG00000138755 | 809.96 | 1.70 | 0.00 | ENSG00000148908 | 567.77 | -1.55 | 0.00 |
| ENSG00000106348 | 3890.03 | 1.70 | 0.00 | ENSG00000139433 | 1339.67 | -1.56 | 0.00 |
| ENSG00000225855 | 233.21 | 1.70 | 0.00 | ENSG00000159200 | 1329.07 | -1.56 | 0.00 |
| ENSG00000274922 | 83.54 | 1.70 | 0.00 | ENSG00000260314 | 481.85 | -1.56 | 0.00 |
| ENSG00000169436 | 63.85 | 1.70 | 0.00 | ENSG00000104324 | 678.46 | -1.56 | 0.00 |
| ENSG00000114631 | 2031.99 | 1.70 | 0.00 | ENSG00000177181 | 424.56 | -1.56 | 0.00 |
| ENSG00000151617 | 534.25 | 1.70 | 0.00 | ENSG00000143167 | 9553.80 | -1.56 | 0.00 |
| ENSG00000178821 | 257.52 | 1.69 | 0.00 | ENSG00000060566 | 64.30 | -1.56 | 0.02 |
| ENSG00000280734 | 219.53 | 1.69 | 0.00 | ENSG00000185585 | 752.22 | -1.56 | 0.00 |
| ENSG00000125657 | 206.61 | 1.69 | 0.00 | ENSG00000165995 | 166.44 | -1.56 | 0.00 |
| ENSG00000112280 | 247.23 | 1.69 | 0.01 | ENSG00000183098 | 341.91 | -1.56 | 0.00 |
| ENSG00000159247 | 60.72 | 1.69 | 0.04 | ENSG00000153162 | 69.86 | -1.56 | 0.00 |
| ENSG00000104881 | 1443.00 | 1.69 | 0.00 | ENSG00000108187 | 712.36 | -1.56 | 0.00 |
| ENSG00000230202 | 373.43 | 1.69 | 0.00 | ENSG00000173546 | 921.66 | -1.56 | 0.00 |
| ENSG00000217801 | 68.21 | 1.69 | 0.00 | ENSG00000131845 | 139.08 | -1.56 | 0.00 |
| ENSG00000077348 | 1618.49 | 1.69 | 0.00 | ENSG00000171747 | 45671.91 | -1.57 | 0.00 |
| ENSG00000198276 | 2791.98 | 1.69 | 0.00 | ENSG00000154274 | 621.03 | -1.57 | 0.00 |
| ENSG00000188015 | 80.11 | 1.69 | 0.00 | ENSG00000104154 | 270.00 | -1.57 | 0.00 |
| ENSG00000151012 | 686.13 | 1.69 | 0.00 | ENSG00000233608 | 62.10 | -1.57 | 0.00 |
| ENSG00000126003 | 4146.03 | 1.69 | 0.00 | ENSG00000053747 | 2690.50 | -1.57 | 0.00 |
| ENSG00000167476 | 135.54 | 1.68 | 0.00 | ENSG00000074370 | 4987.25 | -1.57 | 0.00 |
| ENSG00000183444 | 205.02 | 1.68 | 0.00 | ENSG00000275718 | 185.44 | -1.57 | 0.00 |
| ENSG00000168209 | 3107.24 | 1.68 | 0.00 | ENSG00000137267 | 350.73 | -1.57 | 0.00 |
| ENSG00000245248 | 70.42 | 1.68 | 0.00 | ENSG00000174640 | 909.34 | -1.57 | 0.00 |
| ENSG00000230082 | 89.34 | 1.68 | 0.00 | ENSG00000079385 | 9591.05 | -1.57 | 0.00 |
| ENSG00000260261 | 68.05 | 1.68 | 0.00 | ENSG00000070526 | 5515.29 | -1.57 | 0.01 |
| ENSG00000235703 | 73.15 | 1.68 | 0.00 | ENSG00000101265 | 468.25 | -1.57 | 0.00 |
| ENSG00000168071 | 1958.13 | 1.68 | 0.00 | ENSG00000239697 | 437.87 | -1.57 | 0.00 |
| ENSG00000143590 | 918.30 | 1.68 | 0.00 | ENSG00000113657 | 2854.32 | -1.58 | 0.00 |
| ENSG00000272455 | 94.84 | 1.68 | 0.00 | ENSG00000175395 | 208.56 | -1.58 | 0.00 |
| ENSG00000232956 | 1060.65 | 1.68 | 0.00 | ENSG00000259291 | 181.37 | -1.58 | 0.00 |
| ENSG00000110881 | 748.03 | 1.67 | 0.00 | ENSG00000134240 | 7564.07 | -1.58 | 0.03 |
| ENSG00000232346 | 64.57 | 1.67 | 0.00 | ENSG00000126759 | 78.25 | -1.58 | 0.00 |
| ENSG00000205664 | 325.03 | 1.67 | 0.00 | ENSG00000188833 | 977.90 | -1.58 | 0.02 |
| ENSG00000185803 | 5780.63 | 1.67 | 0.00 | ENSG00000265972 | 15988.48 | -1.58 | 0.00 |
| ENSG00000153551 | 834.20 | 1.66 | 0.00 | ENSG00000234155 | 313.03 | -1.59 | 0.00 |
| ENSG00000130731 | 2950.09 | 1.66 | 0.00 | ENSG00000171522 | 933.68 | -1.59 | 0.00 |
| ENSG00000171159 | 2904.57 | 1.66 | 0.00 | ENSG00000278535 | 1701.25 | -1.59 | 0.00 |
| ENSG00000174365 | 605.81 | 1.66 | 0.00 | ENSG00000211632 | 59.80 | -1.59 | 0.04 |
| ENSG00000230629 | 130.63 | 1.66 | 0.00 | ENSG00000182534 | 1546.20 | -1.59 | 0.00 |
| ENSG00000104907 | 1954.75 | 1.66 | 0.00 | ENSG00000125354 | 957.00 | -1.59 | 0.00 |
| ENSG00000114115 | 930.31 | 1.66 | 0.02 | ENSG00000088756 | 69.93 | -1.59 | 0.00 |
| ENSG00000064545 | 1946.59 | 1.66 | 0.00 | ENSG00000203943 | 303.09 | -1.59 | 0.00 |
| ENSG00000165802 | 4063.46 | 1.66 | 0.00 | ENSG00000107796 | 8185.00 | -1.59 | 0.00 |
| ENSG00000177192 | 1858.86 | 1.65 | 0.00 | ENSG00000167074 | 702.50 | -1.59 | 0.00 |
| ENSG00000140534 | 523.52 | 1.65 | 0.00 | ENSG00000151150 | 770.55 | -1.59 | 0.00 |
| ENSG00000188760 | 204.87 | 1.65 | 0.00 | ENSG00000211956 | 376.41 | -1.59 | 0.03 |
| ENSG00000273619 | 90.57 | 1.65 | 0.00 | ENSG00000185345 | 72.53 | -1.59 | 0.00 |
| ENSG00000132330 | 184.26 | 1.65 | 0.00 | ENSG00000138600 | 2037.82 | -1.59 | 0.00 |
| ENSG00000265298 | 232.16 | 1.65 | 0.00 | ENSG00000171385 | 187.02 | -1.59 | 0.00 |
| ENSG00000197696 | 257.23 | 1.65 | 0.00 | ENSG00000152078 | 900.68 | -1.59 | 0.00 |
| ENSG00000103021 | 420.36 | 1.65 | 0.00 | ENSG00000119686 | 185.14 | -1.60 | 0.00 |
| ENSG00000204366 | 385.97 | 1.65 | 0.00 | ENSG00000161405 | 257.27 | -1.60 | 0.00 |
| ENSG00000251396 | 60.82 | 1.65 | 0.02 | ENSG00000102003 | 157.04 | -1.60 | 0.00 |
| ENSG00000180767 | 153.12 | 1.65 | 0.02 | ENSG00000124772 | 229.03 | -1.60 | 0.00 |
| ENSG00000218891 | 581.74 | 1.64 | 0.00 | ENSG00000167552 | 3105.78 | -1.60 | 0.00 |
| ENSG00000112578 | 1344.68 | 1.64 | 0.00 | ENSG00000179163 | 3092.66 | -1.60 | 0.00 |
| ENSG00000165490 | 341.48 | 1.64 | 0.00 | ENSG00000196507 | 224.70 | -1.60 | 0.00 |
| ENSG00000205089 | 580.85 | 1.64 | 0.00 | ENSG00000153823 | 880.27 | -1.60 | 0.00 |
| ENSG00000182118 | 422.12 | 1.63 | 0.00 | ENSG00000110852 | 132.33 | -1.60 | 0.00 |
| ENSG00000214455 | 172.13 | 1.63 | 0.00 | ENSG00000145088 | 94.94 | -1.60 | 0.00 |
| ENSG00000114113 | 196.61 | 1.63 | 0.02 | ENSG00000123643 | 959.44 | -1.60 | 0.00 |
| ENSG00000107815 | 1140.08 | 1.63 | 0.00 | ENSG00000176387 | 5621.84 | -1.61 | 0.00 |
| ENSG00000105519 | 795.18 | 1.63 | 0.00 | ENSG00000123095 | 744.14 | -1.61 | 0.00 |
| ENSG00000167523 | 454.84 | 1.63 | 0.00 | ENSG00000254838 | 76.37 | -1.61 | 0.00 |
| ENSG00000225968 | 105.69 | 1.63 | 0.00 | ENSG00000081237 | 887.57 | -1.61 | 0.00 |
| ENSG00000181544 | 77.85 | 1.63 | 0.00 | ENSG00000164825 | 141.23 | -1.61 | 0.02 |
| ENSG00000185842 | 227.42 | 1.62 | 0.00 | ENSG00000213203 | 108.98 | -1.61 | 0.00 |
| ENSG00000137309 | 19801.98 | 1.62 | 0.00 | ENSG00000259207 | 88.85 | -1.61 | 0.00 |
| ENSG00000274460 | 50.07 | 1.62 | 0.00 | ENSG00000211945 | 977.70 | -1.61 | 0.03 |
| ENSG00000175920 | 111.51 | 1.62 | 0.00 | ENSG00000100154 | 328.33 | -1.61 | 0.00 |
| ENSG00000227939 | 94.55 | 1.62 | 0.00 | ENSG00000125648 | 2732.17 | -1.61 | 0.00 |
| ENSG00000116883 | 136.50 | 1.62 | 0.00 | ENSG00000183580 | 213.85 | -1.61 | 0.00 |
| ENSG00000225556 | 87.66 | 1.62 | 0.00 | ENSG00000259120 | 109.62 | -1.61 | 0.00 |
| ENSG00000145194 | 891.33 | 1.62 | 0.00 | ENSG00000137672 | 60.57 | -1.61 | 0.00 |
| ENSG00000115009 | 2377.97 | 1.62 | 0.00 | ENSG00000182985 | 293.96 | -1.62 | 0.00 |
| ENSG00000197989 | 539.43 | 1.62 | 0.00 | ENSG00000128815 | 218.17 | -1.62 | 0.00 |
| ENSG00000100036 | 888.14 | 1.61 | 0.00 | ENSG00000137440 | 374.21 | -1.62 | 0.00 |
| ENSG00000231503 | 73.57 | 1.61 | 0.00 | ENSG00000164237 | 2358.14 | -1.62 | 0.00 |
| ENSG00000006327 | 2027.73 | 1.61 | 0.00 | ENSG00000149090 | 238.03 | -1.62 | 0.00 |
| ENSG00000167600 | 7924.85 | 1.61 | 0.00 | ENSG00000143297 | 111.56 | -1.62 | 0.01 |
| ENSG00000159216 | 2730.56 | 1.61 | 0.00 | ENSG00000179909 | 55.77 | -1.62 | 0.00 |
| ENSG00000205336 | 10001.52 | 1.61 | 0.00 | ENSG00000118257 | 1365.06 | -1.62 | 0.00 |
| ENSG00000104341 | 7235.68 | 1.61 | 0.00 | ENSG00000101782 | 2382.13 | -1.63 | 0.00 |
| ENSG00000262468 | 123.60 | 1.61 | 0.00 | ENSG00000065882 | 1895.34 | -1.63 | 0.00 |
| ENSG00000239672 | 2969.67 | 1.61 | 0.00 | ENSG00000042445 | 3952.47 | -1.63 | 0.00 |
| ENSG00000185761 | 258.08 | 1.61 | 0.00 | ENSG00000248771 | 491.62 | -1.63 | 0.00 |
| ENSG00000104783 | 3715.72 | 1.61 | 0.00 | ENSG00000172831 | 9097.43 | -1.63 | 0.00 |
| ENSG00000179406 | 248.42 | 1.60 | 0.00 | ENSG00000104894 | 336.69 | -1.63 | 0.00 |
| ENSG00000133119 | 1373.04 | 1.60 | 0.00 | ENSG00000160539 | 56.79 | -1.63 | 0.00 |
| ENSG00000235043 | 70.80 | 1.60 | 0.00 | ENSG00000047346 | 561.44 | -1.63 | 0.00 |
| ENSG00000106397 | 8302.76 | 1.60 | 0.00 | ENSG00000100532 | 264.68 | -1.63 | 0.00 |
| ENSG00000095739 | 984.33 | 1.60 | 0.00 | ENSG00000131401 | 141.44 | -1.63 | 0.00 |
| ENSG00000116649 | 3680.34 | 1.60 | 0.00 | ENSG00000183801 | 212.13 | -1.63 | 0.00 |
| ENSG00000205940 | 52.73 | 1.60 | 0.00 | ENSG00000182022 | 570.96 | -1.63 | 0.00 |
| ENSG00000106089 | 408.49 | 1.60 | 0.00 | ENSG00000163590 | 466.96 | -1.64 | 0.00 |
| ENSG00000160345 | 77.14 | 1.60 | 0.00 | ENSG00000117016 | 219.18 | -1.64 | 0.00 |
| ENSG00000132635 | 887.56 | 1.60 | 0.00 | ENSG00000102385 | 69.01 | -1.64 | 0.01 |
| ENSG00000279716 | 51.22 | 1.60 | 0.00 | ENSG00000144426 | 311.62 | -1.64 | 0.00 |
| ENSG00000073464 | 310.34 | 1.60 | 0.00 | ENSG00000167912 | 57.53 | -1.64 | 0.00 |
| ENSG00000243547 | 51.54 | 1.60 | 0.00 | ENSG00000163249 | 805.82 | -1.64 | 0.00 |
| ENSG00000234648 | 68.23 | 1.59 | 0.01 | ENSG00000187068 | 400.27 | -1.64 | 0.00 |
| ENSG00000235280 | 207.00 | 1.59 | 0.00 | ENSG00000005102 | 77.83 | -1.64 | 0.00 |
| ENSG00000142632 | 709.09 | 1.59 | 0.00 | ENSG00000054356 | 69.54 | -1.64 | 0.01 |
| ENSG00000222041 | 289.55 | 1.59 | 0.00 | ENSG00000184785 | 96.04 | -1.64 | 0.00 |
| ENSG00000111665 | 971.54 | 1.59 | 0.00 | ENSG00000167800 | 200.40 | -1.64 | 0.01 |
| ENSG00000228929 | 88.52 | 1.59 | 0.00 | ENSG00000185630 | 1698.55 | -1.65 | 0.00 |
| ENSG00000131187 | 760.65 | 1.59 | 0.00 | ENSG00000185565 | 369.48 | -1.65 | 0.00 |
| ENSG00000175318 | 262.30 | 1.59 | 0.00 | ENSG00000166311 | 1099.59 | -1.65 | 0.00 |
| ENSG00000173432 | 319.97 | 1.59 | 0.04 | ENSG00000174500 | 70.46 | -1.65 | 0.00 |
| ENSG00000188185 | 225.60 | 1.59 | 0.00 | ENSG00000176907 | 1258.79 | -1.65 | 0.00 |
| ENSG00000164764 | 467.61 | 1.58 | 0.02 | ENSG00000211660 | 1335.39 | -1.65 | 0.04 |
| ENSG00000278709 | 133.26 | 1.58 | 0.00 | ENSG00000168016 | 1149.23 | -1.65 | 0.00 |
| ENSG00000179611 | 68.15 | 1.58 | 0.00 | ENSG00000166927 | 330.12 | -1.65 | 0.00 |
| ENSG00000141933 | 291.32 | 1.58 | 0.01 | ENSG00000151240 | 608.82 | -1.65 | 0.00 |
| ENSG00000144354 | 5585.10 | 1.58 | 0.00 | ENSG00000148357 | 674.42 | -1.65 | 0.00 |
| ENSG00000123485 | 922.40 | 1.58 | 0.00 | ENSG00000114698 | 583.85 | -1.65 | 0.00 |
| ENSG00000084774 | 3906.64 | 1.58 | 0.00 | ENSG00000128596 | 89.70 | -1.65 | 0.00 |
| ENSG00000188277 | 86.60 | 1.58 | 0.00 | ENSG00000117114 | 450.36 | -1.66 | 0.00 |
| ENSG00000196976 | 1095.74 | 1.58 | 0.00 | ENSG00000183134 | 70.25 | -1.66 | 0.00 |
| ENSG00000111641 | 2503.21 | 1.58 | 0.00 | ENSG00000152284 | 249.80 | -1.66 | 0.00 |
| ENSG00000214826 | 122.90 | 1.57 | 0.00 | ENSG00000111860 | 72.00 | -1.66 | 0.00 |
| ENSG00000212123 | 108.27 | 1.57 | 0.00 | ENSG00000196159 | 397.23 | -1.66 | 0.00 |
| ENSG00000245970 | 186.52 | 1.57 | 0.00 | ENSG00000109790 | 517.80 | -1.66 | 0.00 |
| ENSG00000146904 | 1392.78 | 1.57 | 0.00 | ENSG00000165996 | 119.14 | -1.66 | 0.00 |
| ENSG00000115657 | 191.80 | 1.57 | 0.00 | ENSG00000172379 | 202.59 | -1.67 | 0.00 |
| ENSG00000065600 | 390.24 | 1.57 | 0.00 | ENSG00000179277 | 53.96 | -1.67 | 0.00 |
| ENSG00000091127 | 1222.46 | 1.57 | 0.00 | ENSG00000137757 | 222.10 | -1.67 | 0.00 |
| ENSG00000228288 | 89.86 | 1.57 | 0.00 | ENSG00000129675 | 349.86 | -1.67 | 0.00 |
| ENSG00000090447 | 1092.84 | 1.57 | 0.00 | ENSG00000151632 | 117.31 | -1.67 | 0.00 |
| ENSG00000149380 | 90.67 | 1.57 | 0.00 | ENSG00000131171 | 2642.33 | -1.67 | 0.00 |
| ENSG00000189007 | 549.02 | 1.57 | 0.00 | ENSG00000175287 | 93.13 | -1.67 | 0.00 |
| ENSG00000147799 | 971.14 | 1.57 | 0.00 | ENSG00000185070 | 200.48 | -1.67 | 0.00 |
| ENSG00000087586 | 1938.80 | 1.56 | 0.00 | ENSG00000119950 | 1506.42 | -1.67 | 0.00 |
| ENSG00000229891 | 215.55 | 1.56 | 0.00 | ENSG00000159433 | 222.98 | -1.67 | 0.00 |
| ENSG00000236255 | 68.87 | 1.56 | 0.00 | ENSG00000187134 | 143.34 | -1.67 | 0.00 |
| ENSG00000258947 | 104.13 | 1.56 | 0.00 | ENSG00000198125 | 103.69 | -1.67 | 0.01 |
| ENSG00000269352 | 98.10 | 1.56 | 0.00 | ENSG00000169116 | 7173.79 | -1.68 | 0.00 |
| ENSG00000179588 | 621.75 | 1.56 | 0.00 | ENSG00000135709 | 538.78 | -1.68 | 0.00 |
| ENSG00000144485 | 2275.98 | 1.56 | 0.00 | ENSG00000169418 | 161.21 | -1.68 | 0.00 |
| ENSG00000127415 | 610.96 | 1.56 | 0.00 | ENSG00000183578 | 145.94 | -1.68 | 0.00 |
| ENSG00000143333 | 662.16 | 1.56 | 0.00 | ENSG00000109099 | 1674.65 | -1.68 | 0.00 |
| ENSG00000093009 | 790.72 | 1.56 | 0.00 | ENSG00000049130 | 830.33 | -1.69 | 0.00 |
| ENSG00000185332 | 73.93 | 1.56 | 0.00 | ENSG00000211976 | 218.03 | -1.69 | 0.04 |
| ENSG00000280063 | 70.11 | 1.56 | 0.00 | ENSG00000145861 | 52.25 | -1.69 | 0.00 |
| ENSG00000126709 | 3414.90 | 1.56 | 0.00 | ENSG00000121297 | 231.60 | -1.69 | 0.00 |
| ENSG00000180385 | 138.79 | 1.56 | 0.00 | ENSG00000103241 | 737.18 | -1.69 | 0.00 |
| ENSG00000101199 | 5308.89 | 1.55 | 0.00 | ENSG00000065809 | 1422.66 | -1.69 | 0.00 |
| ENSG00000101294 | 10538.62 | 1.55 | 0.00 | ENSG00000115896 | 96.77 | -1.69 | 0.00 |
| ENSG00000120262 | 259.39 | 1.55 | 0.00 | ENSG00000060140 | 356.37 | -1.69 | 0.00 |
| ENSG00000065328 | 463.03 | 1.55 | 0.00 | ENSG00000187391 | 105.37 | -1.69 | 0.00 |
| ENSG00000085465 | 237.39 | 1.55 | 0.00 | ENSG00000211668 | 1025.64 | -1.70 | 0.04 |
| ENSG00000215440 | 660.39 | 1.55 | 0.00 | ENSG00000182013 | 62.21 | -1.70 | 0.00 |
| ENSG00000168993 | 282.41 | 1.55 | 0.00 | ENSG00000104756 | 782.47 | -1.70 | 0.00 |
| ENSG00000226380 | 91.54 | 1.55 | 0.00 | ENSG00000083814 | 61.55 | -1.70 | 0.00 |
| ENSG00000134070 | 576.81 | 1.55 | 0.00 | ENSG00000158473 | 102.70 | -1.70 | 0.00 |
| ENSG00000006015 | 1697.79 | 1.54 | 0.00 | ENSG00000179954 | 565.05 | -1.70 | 0.00 |
| ENSG00000240972 | 2299.75 | 1.54 | 0.00 | ENSG00000010319 | 207.73 | -1.70 | 0.00 |
| ENSG00000139438 | 502.64 | 1.54 | 0.00 | ENSG00000122971 | 1863.59 | -1.70 | 0.00 |
| ENSG00000114251 | 928.08 | 1.54 | 0.00 | ENSG00000091622 | 529.01 | -1.71 | 0.00 |
| ENSG00000197182 | 78.32 | 1.54 | 0.00 | ENSG00000138744 | 1231.55 | -1.71 | 0.00 |
| ENSG00000124466 | 154.19 | 1.54 | 0.01 | ENSG00000127995 | 735.71 | -1.71 | 0.00 |
| ENSG00000101230 | 119.64 | 1.54 | 0.05 | ENSG00000166313 | 376.06 | -1.71 | 0.00 |
| ENSG00000249992 | 500.60 | 1.54 | 0.00 | ENSG00000134352 | 2177.63 | -1.71 | 0.00 |
| ENSG00000122691 | 118.40 | 1.54 | 0.00 | ENSG00000112210 | 514.56 | -1.71 | 0.00 |
| ENSG00000203688 | 107.55 | 1.54 | 0.00 | ENSG00000090104 | 1034.41 | -1.71 | 0.00 |
| ENSG00000112984 | 921.04 | 1.54 | 0.00 | ENSG00000170476 | 472.41 | -1.71 | 0.00 |
| ENSG00000185513 | 299.25 | 1.54 | 0.00 | ENSG00000154678 | 67.57 | -1.71 | 0.00 |
| ENSG00000186704 | 94.51 | 1.53 | 0.00 | ENSG00000102781 | 233.30 | -1.71 | 0.00 |
| ENSG00000133106 | 1853.40 | 1.53 | 0.00 | ENSG00000072657 | 73.95 | -1.71 | 0.00 |
| ENSG00000257497 | 50.60 | 1.53 | 0.00 | ENSG00000129682 | 127.22 | -1.72 | 0.00 |
| ENSG00000226415 | 115.94 | 1.53 | 0.00 | ENSG00000117569 | 330.95 | -1.72 | 0.00 |
| ENSG00000104889 | 1688.82 | 1.53 | 0.00 | ENSG00000164691 | 166.33 | -1.72 | 0.00 |
| ENSG00000125864 | 110.87 | 1.53 | 0.00 | ENSG00000112773 | 907.16 | -1.72 | 0.00 |
| ENSG00000185201 | 4429.29 | 1.53 | 0.00 | ENSG00000115380 | 1277.15 | -1.72 | 0.00 |
| ENSG00000197457 | 1703.88 | 1.53 | 0.01 | ENSG00000116473 | 2169.16 | -1.72 | 0.00 |
| ENSG00000102030 | 2325.56 | 1.53 | 0.00 | ENSG00000116106 | 252.95 | -1.72 | 0.00 |
| ENSG00000202198 | 209.82 | 1.53 | 0.01 | ENSG00000110900 | 447.67 | -1.72 | 0.00 |
| ENSG00000133315 | 1746.23 | 1.53 | 0.00 | ENSG00000157570 | 577.95 | -1.72 | 0.00 |
| ENSG00000120685 | 4796.11 | 1.52 | 0.00 | ENSG00000169071 | 382.87 | -1.72 | 0.00 |
| ENSG00000180574 | 62.41 | 1.52 | 0.01 | ENSG00000211973 | 167.23 | -1.72 | 0.04 |
| ENSG00000104356 | 557.35 | 1.52 | 0.00 | ENSG00000134061 | 101.75 | -1.72 | 0.00 |
| ENSG00000162782 | 75.25 | 1.52 | 0.01 | ENSG00000205413 | 670.83 | -1.73 | 0.00 |
| ENSG00000149948 | 599.18 | 1.52 | 0.01 | ENSG00000145703 | 1309.00 | -1.73 | 0.00 |
| ENSG00000204262 | 6072.04 | 1.52 | 0.00 | ENSG00000185811 | 275.42 | -1.73 | 0.00 |
| ENSG00000108479 | 1179.31 | 1.52 | 0.00 | ENSG00000177469 | 4818.92 | -1.73 | 0.00 |
| ENSG00000134297 | 114.17 | 1.52 | 0.00 | ENSG00000117020 | 463.87 | -1.73 | 0.00 |
| ENSG00000197472 | 52.47 | 1.52 | 0.00 | ENSG00000111727 | 270.81 | -1.73 | 0.00 |
| ENSG00000212864 | 374.59 | 1.52 | 0.00 | ENSG00000110013 | 3153.70 | -1.73 | 0.00 |
| ENSG00000261884 | 91.12 | 1.52 | 0.00 | ENSG00000119865 | 178.33 | -1.73 | 0.00 |
| ENSG00000159674 | 2828.92 | 1.52 | 0.00 | ENSG00000171503 | 663.39 | -1.73 | 0.00 |
| ENSG00000165171 | 316.97 | 1.52 | 0.01 | ENSG00000112782 | 1929.88 | -1.73 | 0.00 |
| ENSG00000114554 | 3339.52 | 1.52 | 0.00 | ENSG00000069702 | 777.87 | -1.73 | 0.00 |
| ENSG00000197961 | 1355.54 | 1.52 | 0.00 | ENSG00000118596 | 112.03 | -1.73 | 0.00 |
| ENSG00000124207 | 7192.73 | 1.52 | 0.00 | ENSG00000126500 | 51.07 | -1.74 | 0.00 |
| ENSG00000186827 | 154.13 | 1.52 | 0.00 | ENSG00000062524 | 192.21 | -1.74 | 0.00 |
| ENSG00000177464 | 221.53 | 1.52 | 0.00 | ENSG00000164442 | 1004.42 | -1.74 | 0.00 |
| ENSG00000134824 | 2542.46 | 1.52 | 0.01 | ENSG00000176903 | 798.63 | -1.74 | 0.00 |
| ENSG00000243829 | 61.61 | 1.51 | 0.01 | ENSG00000198961 | 2533.18 | -1.74 | 0.00 |
| ENSG00000197275 | 134.76 | 1.51 | 0.00 | ENSG00000127920 | 460.99 | -1.74 | 0.00 |
| ENSG00000215006 | 91.05 | 1.51 | 0.00 | ENSG00000127955 | 452.20 | -1.74 | 0.00 |
| ENSG00000203747 | 854.68 | 1.51 | 0.00 | ENSG00000157404 | 359.00 | -1.74 | 0.00 |
| ENSG00000234912 | 235.60 | 1.51 | 0.00 | ENSG00000109814 | 2299.70 | -1.75 | 0.00 |
| ENSG00000270012 | 74.24 | 1.51 | 0.00 | ENSG00000185339 | 752.04 | -1.75 | 0.00 |
| ENSG00000182841 | 256.83 | 1.51 | 0.00 | ENSG00000211669 | 388.73 | -1.75 | 0.05 |
| ENSG00000255717 | 1776.85 | 1.51 | 0.00 | ENSG00000110080 | 1867.65 | -1.75 | 0.00 |
| ENSG00000173110 | 127.13 | 1.51 | 0.00 | ENSG00000177432 | 86.97 | -1.75 | 0.00 |
| ENSG00000101311 | 10231.63 | 1.51 | 0.00 | ENSG00000092096 | 236.43 | -1.75 | 0.00 |
| ENSG00000172167 | 278.83 | 1.51 | 0.00 | ENSG00000143248 | 3270.96 | -1.75 | 0.00 |
| ENSG00000114767 | 1902.56 | 1.51 | 0.00 | ENSG00000026751 | 475.95 | -1.76 | 0.00 |
| ENSG00000029153 | 788.42 | 1.51 | 0.00 | ENSG00000171016 | 82.32 | -1.76 | 0.00 |
| ENSG00000276672 | 80.03 | 1.51 | 0.00 | ENSG00000154589 | 77.69 | -1.76 | 0.00 |
| ENSG00000106009 | 3267.53 | 1.51 | 0.00 | ENSG00000218336 | 136.50 | -1.76 | 0.01 |
| ENSG00000167700 | 1581.66 | 1.50 | 0.00 | ENSG00000253304 | 104.74 | -1.76 | 0.00 |
| ENSG00000198807 | 52.11 | 1.50 | 0.02 | ENSG00000144057 | 197.47 | -1.76 | 0.02 |
| ENSG00000215012 | 1239.12 | 1.50 | 0.00 | ENSG00000197943 | 356.41 | -1.76 | 0.00 |
| ENSG00000162063 | 1266.69 | 1.50 | 0.00 | ENSG00000081923 | 5441.57 | -1.76 | 0.00 |
| ENSG00000007376 | 1859.60 | 1.50 | 0.00 | ENSG00000165338 | 126.14 | -1.77 | 0.00 |
| ENSG00000100228 | 472.76 | 1.50 | 0.00 | ENSG00000155545 | 1240.61 | -1.77 | 0.00 |
| ENSG00000177542 | 1488.22 | 1.50 | 0.00 | ENSG00000126803 | 1168.06 | -1.77 | 0.00 |
| ENSG00000261505 | 148.30 | 1.50 | 0.00 | ENSG00000104059 | 171.85 | -1.77 | 0.01 |
| ENSG00000169962 | 180.32 | 1.49 | 0.02 | ENSG00000085265 | 111.61 | -1.78 | 0.00 |
| ENSG00000253716 | 230.76 | 1.49 | 0.00 | ENSG00000078018 | 71.91 | -1.78 | 0.00 |
| ENSG00000135476 | 1046.60 | 1.49 | 0.00 | ENSG00000110077 | 687.02 | -1.78 | 0.00 |
| ENSG00000178896 | 1452.11 | 1.49 | 0.00 | ENSG00000167037 | 111.96 | -1.78 | 0.00 |
| ENSG00000136982 | 535.95 | 1.49 | 0.00 | ENSG00000000971 | 855.19 | -1.78 | 0.00 |
| ENSG00000196793 | 245.99 | 1.49 | 0.00 | ENSG00000172578 | 179.82 | -1.78 | 0.00 |
| ENSG00000177989 | 309.84 | 1.49 | 0.00 | ENSG00000080031 | 1558.66 | -1.78 | 0.00 |
| ENSG00000091879 | 310.83 | 1.49 | 0.00 | ENSG00000276855 | 51.14 | -1.79 | 0.00 |
| ENSG00000128165 | 428.30 | 1.49 | 0.00 | ENSG00000225335 | 65.07 | -1.79 | 0.00 |
| ENSG00000101898 | 67.22 | 1.49 | 0.00 | ENSG00000069431 | 127.11 | -1.79 | 0.00 |
| ENSG00000016402 | 1103.17 | 1.49 | 0.00 | ENSG00000043039 | 215.41 | -1.79 | 0.00 |
| ENSG00000180921 | 7949.75 | 1.49 | 0.00 | ENSG00000146374 | 141.24 | -1.79 | 0.01 |
| ENSG00000166851 | 2777.64 | 1.49 | 0.00 | ENSG00000134755 | 3525.67 | -1.79 | 0.00 |
| ENSG00000177410 | 4748.76 | 1.49 | 0.00 | ENSG00000169122 | 73.18 | -1.79 | 0.00 |
| ENSG00000155367 | 60.57 | 1.49 | 0.00 | ENSG00000243466 | 2114.65 | -1.79 | 0.01 |
| ENSG00000146521 | 147.82 | 1.49 | 0.00 | ENSG00000107130 | 1342.51 | -1.79 | 0.00 |
| ENSG00000121904 | 60.69 | 1.48 | 0.00 | ENSG00000050628 | 146.83 | -1.79 | 0.00 |
| ENSG00000088356 | 1539.17 | 1.48 | 0.00 | ENSG00000076641 | 1010.65 | -1.79 | 0.00 |
| ENSG00000225177 | 60.78 | 1.48 | 0.00 | ENSG00000126353 | 107.24 | -1.80 | 0.00 |
| ENSG00000242294 | 187.65 | 1.48 | 0.00 | ENSG00000162391 | 56.69 | -1.80 | 0.00 |
| ENSG00000198088 | 181.68 | 1.48 | 0.00 | ENSG00000152784 | 81.77 | -1.80 | 0.00 |
| ENSG00000242299 | 616.81 | 1.48 | 0.00 | ENSG00000185532 | 352.51 | -1.80 | 0.00 |
| ENSG00000182685 | 108.66 | 1.48 | 0.00 | ENSG00000182636 | 400.07 | -1.80 | 0.00 |
| ENSG00000118193 | 388.86 | 1.48 | 0.00 | ENSG00000211648 | 580.38 | -1.80 | 0.02 |
| ENSG00000221944 | 205.01 | 1.48 | 0.00 | ENSG00000102409 | 380.38 | -1.80 | 0.00 |
| ENSG00000151651 | 626.00 | 1.48 | 0.00 | ENSG00000198682 | 2931.23 | -1.80 | 0.00 |
| ENSG00000223803 | 65.57 | 1.48 | 0.00 | ENSG00000171227 | 696.08 | -1.80 | 0.00 |
| ENSG00000181588 | 1440.10 | 1.48 | 0.00 | ENSG00000130558 | 224.65 | -1.81 | 0.00 |
| ENSG00000187486 | 140.34 | 1.48 | 0.00 | ENSG00000138356 | 133.68 | -1.81 | 0.00 |
| ENSG00000246898 | 160.79 | 1.47 | 0.00 | ENSG00000158201 | 1102.94 | -1.81 | 0.00 |
| ENSG00000130827 | 2476.61 | 1.47 | 0.00 | ENSG00000163297 | 2070.46 | -1.81 | 0.00 |
| ENSG00000147955 | 6245.77 | 1.47 | 0.00 | ENSG00000196502 | 775.45 | -1.81 | 0.00 |
| ENSG00000075618 | 2029.37 | 1.47 | 0.00 | ENSG00000211592 | 34025.21 | -1.81 | 0.01 |
| ENSG00000086548 | 55794.16 | 1.47 | 0.00 | ENSG00000151229 | 475.10 | -1.81 | 0.00 |
| ENSG00000188706 | 5950.83 | 1.47 | 0.00 | ENSG00000005108 | 65.51 | -1.81 | 0.00 |
| ENSG00000100429 | 376.31 | 1.47 | 0.00 | ENSG00000131711 | 605.83 | -1.81 | 0.00 |
| ENSG00000142552 | 1149.82 | 1.46 | 0.00 | ENSG00000197253 | 273.52 | -1.81 | 0.00 |
| ENSG00000013573 | 1172.42 | 1.46 | 0.00 | ENSG00000263429 | 830.14 | -1.81 | 0.00 |
| ENSG00000243753 | 858.42 | 1.46 | 0.05 | ENSG00000163681 | 2309.64 | -1.82 | 0.00 |
| ENSG00000226696 | 100.34 | 1.46 | 0.00 | ENSG00000139193 | 118.47 | -1.82 | 0.00 |
| ENSG00000105976 | 5432.92 | 1.46 | 0.00 | ENSG00000213366 | 138.47 | -1.82 | 0.00 |
| ENSG00000101146 | 2505.39 | 1.46 | 0.00 | ENSG00000108405 | 96.03 | -1.82 | 0.00 |
| ENSG00000087495 | 112.67 | 1.46 | 0.03 | ENSG00000076555 | 799.69 | -1.82 | 0.00 |
| ENSG00000050438 | 155.10 | 1.46 | 0.00 | ENSG00000224373 | 713.03 | -1.82 | 0.01 |
| ENSG00000138772 | 3675.65 | 1.46 | 0.00 | ENSG00000186594 | 403.14 | -1.82 | 0.00 |
| ENSG00000245149 | 75.81 | 1.46 | 0.00 | ENSG00000171867 | 2354.57 | -1.82 | 0.00 |
| ENSG00000213889 | 91.15 | 1.45 | 0.00 | ENSG00000241351 | 2505.29 | -1.82 | 0.01 |
| ENSG00000180758 | 1095.82 | 1.45 | 0.00 | ENSG00000171916 | 150.83 | -1.82 | 0.02 |
| ENSG00000136244 | 158.80 | 1.45 | 0.04 | ENSG00000240225 | 83.79 | -1.82 | 0.00 |
| ENSG00000116852 | 1252.68 | 1.45 | 0.00 | ENSG00000100302 | 393.85 | -1.82 | 0.00 |
| ENSG00000165724 | 1061.93 | 1.45 | 0.00 | ENSG00000124212 | 413.06 | -1.82 | 0.01 |
| ENSG00000130635 | 9178.95 | 1.45 | 0.00 | ENSG00000185862 | 239.47 | -1.82 | 0.00 |
| ENSG00000100068 | 251.53 | 1.45 | 0.00 | ENSG00000211949 | 1561.93 | -1.82 | 0.01 |
| ENSG00000137571 | 119.02 | 1.45 | 0.00 | ENSG00000170962 | 222.35 | -1.82 | 0.00 |
| ENSG00000106404 | 1668.91 | 1.45 | 0.01 | ENSG00000280237 | 129.79 | -1.83 | 0.00 |
| ENSG00000130332 | 3699.80 | 1.44 | 0.00 | ENSG00000258441 | 162.47 | -1.83 | 0.00 |
| ENSG00000054598 | 246.19 | 1.44 | 0.01 | ENSG00000110079 | 222.37 | -1.83 | 0.00 |
| ENSG00000243554 | 129.27 | 1.44 | 0.00 | ENSG00000134202 | 638.55 | -1.83 | 0.00 |
| ENSG00000177839 | 69.97 | 1.44 | 0.01 | ENSG00000140682 | 1229.42 | -1.83 | 0.00 |
| ENSG00000165828 | 4245.47 | 1.44 | 0.01 | ENSG00000067113 | 906.49 | -1.83 | 0.00 |
| ENSG00000185252 | 757.53 | 1.44 | 0.00 | ENSG00000090659 | 226.35 | -1.83 | 0.00 |
| ENSG00000182199 | 5858.40 | 1.44 | 0.00 | ENSG00000235098 | 69.56 | -1.83 | 0.00 |
| ENSG00000261556 | 84.10 | 1.44 | 0.00 | ENSG00000075884 | 128.26 | -1.84 | 0.00 |
| ENSG00000224186 | 64.93 | 1.44 | 0.00 | ENSG00000162817 | 1018.51 | -1.84 | 0.00 |
| ENSG00000237669 | 51.63 | 1.44 | 0.01 | ENSG00000179144 | 174.91 | -1.84 | 0.00 |
| ENSG00000141934 | 5275.57 | 1.44 | 0.00 | ENSG00000184349 | 131.31 | -1.84 | 0.00 |
| ENSG00000144810 | 1538.92 | 1.44 | 0.01 | ENSG00000170298 | 133.40 | -1.84 | 0.01 |
| ENSG00000160813 | 915.92 | 1.44 | 0.00 | ENSG00000072694 | 118.94 | -1.84 | 0.00 |
| ENSG00000082512 | 1978.41 | 1.44 | 0.00 | ENSG00000111913 | 152.50 | -1.84 | 0.00 |
| ENSG00000250318 | 131.64 | 1.44 | 0.00 | ENSG00000103710 | 293.60 | -1.84 | 0.00 |
| ENSG00000187840 | 2403.16 | 1.44 | 0.00 | ENSG00000168903 | 529.25 | -1.84 | 0.00 |
| ENSG00000234432 | 105.06 | 1.44 | 0.00 | ENSG00000156299 | 119.32 | -1.84 | 0.00 |
| ENSG00000163995 | 1030.88 | 1.43 | 0.00 | ENSG00000211655 | 78.29 | -1.84 | 0.04 |
| ENSG00000224739 | 58.04 | 1.43 | 0.00 | ENSG00000232216 | 68.33 | -1.85 | 0.01 |
| ENSG00000073536 | 1378.47 | 1.43 | 0.00 | ENSG00000254709 | 1507.12 | -1.85 | 0.01 |
| ENSG00000101407 | 1808.95 | 1.43 | 0.00 | ENSG00000211899 | 7722.51 | -1.85 | 0.01 |
| ENSG00000127399 | 1074.16 | 1.43 | 0.00 | ENSG00000182162 | 150.14 | -1.85 | 0.00 |
| ENSG00000272752 | 83.42 | 1.43 | 0.00 | ENSG00000059728 | 2231.55 | -1.85 | 0.00 |
| ENSG00000186871 | 316.24 | 1.43 | 0.00 | ENSG00000028277 | 154.43 | -1.85 | 0.00 |
| ENSG00000273151 | 308.44 | 1.43 | 0.00 | ENSG00000242732 | 91.67 | -1.86 | 0.00 |
| ENSG00000233493 | 979.50 | 1.43 | 0.02 | ENSG00000133116 | 66.72 | -1.86 | 0.00 |
| ENSG00000126602 | 4763.09 | 1.43 | 0.00 | ENSG00000064300 | 115.03 | -1.86 | 0.00 |
| ENSG00000114346 | 3078.22 | 1.43 | 0.00 | ENSG00000168060 | 212.88 | -1.86 | 0.00 |
| ENSG00000178623 | 4041.55 | 1.43 | 0.00 | ENSG00000177363 | 113.02 | -1.86 | 0.00 |
| ENSG00000139734 | 680.81 | 1.43 | 0.00 | ENSG00000164330 | 113.40 | -1.86 | 0.00 |
| ENSG00000184216 | 9725.35 | 1.43 | 0.00 | ENSG00000020577 | 501.63 | -1.86 | 0.00 |
| ENSG00000100003 | 239.67 | 1.42 | 0.00 | ENSG00000161281 | 191.63 | -1.86 | 0.00 |
| ENSG00000135127 | 590.42 | 1.42 | 0.00 | ENSG00000180447 | 253.47 | -1.87 | 0.01 |
| ENSG00000151136 | 129.69 | 1.42 | 0.02 | ENSG00000166532 | 193.97 | -1.87 | 0.00 |
| ENSG00000161249 | 698.88 | 1.42 | 0.02 | ENSG00000164114 | 110.84 | -1.87 | 0.00 |
| ENSG00000132680 | 1768.63 | 1.42 | 0.00 | ENSG00000241294 | 262.34 | -1.87 | 0.03 |
| ENSG00000049768 | 144.68 | 1.42 | 0.00 | ENSG00000187098 | 227.26 | -1.87 | 0.00 |
| ENSG00000148935 | 90.01 | 1.42 | 0.00 | ENSG00000241399 | 164.41 | -1.87 | 0.00 |
| ENSG00000149257 | 8096.34 | 1.42 | 0.00 | ENSG00000168077 | 376.60 | -1.87 | 0.00 |
| ENSG00000110092 | 8006.48 | 1.42 | 0.00 | ENSG00000184005 | 78.43 | -1.87 | 0.00 |
| ENSG00000146670 | 1743.43 | 1.42 | 0.00 | ENSG00000172828 | 1379.40 | -1.87 | 0.00 |
| ENSG00000136108 | 2061.09 | 1.42 | 0.00 | ENSG00000122707 | 222.46 | -1.87 | 0.00 |
| ENSG00000170293 | 637.90 | 1.42 | 0.00 | ENSG00000146122 | 324.88 | -1.87 | 0.00 |
| ENSG00000213621 | 50.83 | 1.42 | 0.00 | ENSG00000136457 | 62.60 | -1.88 | 0.00 |
| ENSG00000162650 | 243.36 | 1.42 | 0.00 | ENSG00000164342 | 322.78 | -1.88 | 0.00 |
| ENSG00000184465 | 507.71 | 1.42 | 0.00 | ENSG00000166780 | 288.39 | -1.88 | 0.00 |
| ENSG00000233762 | 339.42 | 1.41 | 0.00 | ENSG00000206538 | 199.30 | -1.88 | 0.00 |
| ENSG00000105327 | 776.59 | 1.41 | 0.00 | ENSG00000164161 | 163.72 | -1.88 | 0.00 |
| ENSG00000244486 | 455.16 | 1.41 | 0.01 | ENSG00000198771 | 286.49 | -1.88 | 0.00 |
| ENSG00000272720 | 64.91 | 1.41 | 0.00 | ENSG00000211965 | 360.91 | -1.88 | 0.01 |
| ENSG00000084207 | 25576.51 | 1.41 | 0.00 | ENSG00000006747 | 598.54 | -1.88 | 0.00 |
| ENSG00000171346 | 329.51 | 1.41 | 0.00 | ENSG00000109265 | 433.74 | -1.88 | 0.00 |
| ENSG00000160072 | 1204.09 | 1.41 | 0.00 | ENSG00000150593 | 3622.99 | -1.88 | 0.00 |
| ENSG00000197774 | 486.09 | 1.41 | 0.00 | ENSG00000169213 | 172.78 | -1.88 | 0.00 |
| ENSG00000109065 | 1373.97 | 1.41 | 0.00 | ENSG00000006740 | 754.30 | -1.88 | 0.00 |
| ENSG00000181019 | 7540.53 | 1.41 | 0.00 | ENSG00000196263 | 68.25 | -1.89 | 0.00 |
| ENSG00000274925 | 97.51 | 1.41 | 0.00 | ENSG00000152763 | 99.02 | -1.89 | 0.00 |
| ENSG00000224511 | 52.18 | 1.41 | 0.00 | ENSG00000144857 | 322.53 | -1.89 | 0.00 |
| ENSG00000137135 | 617.28 | 1.41 | 0.00 | ENSG00000069974 | 939.14 | -1.89 | 0.00 |
| ENSG00000149485 | 1091.08 | 1.41 | 0.00 | ENSG00000164128 | 62.26 | -1.89 | 0.01 |
| ENSG00000094804 | 1266.85 | 1.41 | 0.00 | ENSG00000081189 | 414.20 | -1.89 | 0.00 |
| ENSG00000179041 | 1821.73 | 1.41 | 0.00 | ENSG00000239855 | 325.07 | -1.89 | 0.01 |
| ENSG00000186416 | 672.95 | 1.41 | 0.00 | ENSG00000138193 | 947.83 | -1.89 | 0.00 |
| ENSG00000245648 | 125.89 | 1.41 | 0.03 | ENSG00000010671 | 123.36 | -1.89 | 0.00 |
| ENSG00000114993 | 2990.81 | 1.40 | 0.00 | ENSG00000227372 | 144.94 | -1.89 | 0.00 |
| ENSG00000279233 | 69.26 | 1.40 | 0.00 | ENSG00000170390 | 99.74 | -1.89 | 0.00 |
| ENSG00000168003 | 7384.23 | 1.40 | 0.00 | ENSG00000082196 | 187.45 | -1.89 | 0.00 |
| ENSG00000073060 | 4861.27 | 1.40 | 0.00 | ENSG00000101445 | 263.29 | -1.89 | 0.00 |
| ENSG00000124228 | 4521.77 | 1.40 | 0.00 | ENSG00000168405 | 161.86 | -1.90 | 0.00 |
| ENSG00000174586 | 69.96 | 1.40 | 0.00 | ENSG00000140323 | 190.21 | -1.90 | 0.00 |
| ENSG00000047634 | 1064.01 | 1.40 | 0.00 | ENSG00000046889 | 100.84 | -1.90 | 0.00 |
| ENSG00000169246 | 59.49 | 1.40 | 0.00 | ENSG00000152495 | 78.07 | -1.90 | 0.00 |
| ENSG00000130826 | 5729.26 | 1.40 | 0.00 | ENSG00000115841 | 195.51 | -1.90 | 0.00 |
| ENSG00000169710 | 14180.16 | 1.40 | 0.00 | ENSG00000147036 | 74.71 | -1.91 | 0.00 |
| ENSG00000163220 | 1550.26 | 1.40 | 0.02 | ENSG00000121440 | 707.37 | -1.91 | 0.00 |
| ENSG00000244649 | 1700.14 | 1.40 | 0.00 | ENSG00000162894 | 164.07 | -1.91 | 0.00 |
| ENSG00000157303 | 338.49 | 1.40 | 0.01 | ENSG00000174562 | 64.74 | -1.91 | 0.01 |
| ENSG00000168005 | 904.28 | 1.40 | 0.00 | ENSG00000134531 | 4077.19 | -1.91 | 0.00 |
| ENSG00000239467 | 71.39 | 1.39 | 0.00 | ENSG00000068831 | 130.01 | -1.91 | 0.00 |
| ENSG00000085999 | 446.69 | 1.39 | 0.00 | ENSG00000103942 | 56.95 | -1.92 | 0.00 |
| ENSG00000187741 | 916.77 | 1.39 | 0.00 | ENSG00000153898 | 368.37 | -1.92 | 0.00 |
| ENSG00000060656 | 620.64 | 1.39 | 0.02 | ENSG00000198846 | 371.09 | -1.92 | 0.00 |
| ENSG00000184967 | 1372.88 | 1.39 | 0.00 | ENSG00000136732 | 349.78 | -1.92 | 0.00 |
| ENSG00000164692 | 56879.76 | 1.39 | 0.00 | ENSG00000204186 | 99.40 | -1.92 | 0.00 |
| ENSG00000059588 | 1892.35 | 1.39 | 0.00 | ENSG00000007237 | 717.94 | -1.92 | 0.00 |
| ENSG00000214160 | 3391.04 | 1.39 | 0.00 | ENSG00000140450 | 1071.43 | -1.92 | 0.00 |
| ENSG00000185008 | 191.72 | 1.39 | 0.01 | ENSG00000138131 | 84.39 | -1.92 | 0.00 |
| ENSG00000149922 | 65.92 | 1.39 | 0.00 | ENSG00000158270 | 287.51 | -1.92 | 0.00 |
| ENSG00000135100 | 845.65 | 1.38 | 0.00 | ENSG00000116678 | 252.49 | -1.92 | 0.00 |
| ENSG00000100985 | 1673.90 | 1.38 | 0.01 | ENSG00000211941 | 509.94 | -1.93 | 0.01 |
| ENSG00000116455 | 2131.66 | 1.38 | 0.00 | ENSG00000162520 | 72.48 | -1.93 | 0.00 |
| ENSG00000109321 | 4041.02 | 1.38 | 0.00 | ENSG00000166086 | 567.89 | -1.93 | 0.00 |
| ENSG00000270804 | 154.05 | 1.38 | 0.00 | ENSG00000145012 | 4368.66 | -1.93 | 0.00 |
| ENSG00000213638 | 352.64 | 1.38 | 0.01 | ENSG00000111796 | 90.97 | -1.93 | 0.00 |
| ENSG00000156127 | 180.66 | 1.38 | 0.01 | ENSG00000211966 | 1203.50 | -1.93 | 0.01 |
| ENSG00000215252 | 263.99 | 1.38 | 0.00 | ENSG00000174808 | 292.34 | -1.93 | 0.00 |
| ENSG00000135912 | 1268.13 | 1.38 | 0.00 | ENSG00000111802 | 3604.55 | -1.93 | 0.00 |
| ENSG00000213420 | 60.71 | 1.38 | 0.00 | ENSG00000131781 | 702.51 | -1.94 | 0.00 |
| ENSG00000183779 | 9597.34 | 1.38 | 0.00 | ENSG00000211666 | 1531.97 | -1.94 | 0.01 |
| ENSG00000148200 | 455.03 | 1.38 | 0.00 | ENSG00000179094 | 864.69 | -1.94 | 0.00 |
| ENSG00000133627 | 402.53 | 1.38 | 0.00 | ENSG00000185909 | 364.18 | -1.94 | 0.00 |
| ENSG00000162366 | 4045.20 | 1.38 | 0.00 | ENSG00000182963 | 222.42 | -1.94 | 0.00 |
| ENSG00000240376 | 277.41 | 1.38 | 0.02 | ENSG00000196664 | 66.06 | -1.94 | 0.00 |
| ENSG00000230897 | 90.47 | 1.38 | 0.02 | ENSG00000155962 | 192.76 | -1.95 | 0.00 |
| ENSG00000233016 | 1629.43 | 1.38 | 0.00 | ENSG00000215018 | 163.54 | -1.95 | 0.00 |
| ENSG00000167962 | 2868.14 | 1.38 | 0.00 | ENSG00000183963 | 4998.55 | -1.95 | 0.00 |
| ENSG00000156802 | 1860.90 | 1.38 | 0.00 | ENSG00000144935 | 74.14 | -1.95 | 0.00 |
| ENSG00000080573 | 697.09 | 1.37 | 0.00 | ENSG00000134874 | 168.42 | -1.96 | 0.00 |
| ENSG00000171222 | 3542.90 | 1.37 | 0.00 | ENSG00000211895 | 50746.32 | -1.96 | 0.01 |
| ENSG00000111344 | 527.88 | 1.37 | 0.02 | ENSG00000169291 | 119.27 | -1.96 | 0.00 |
| ENSG00000122515 | 6696.41 | 1.37 | 0.00 | ENSG00000153902 | 200.51 | -1.96 | 0.00 |
| ENSG00000178718 | 1572.37 | 1.37 | 0.00 | ENSG00000165124 | 420.34 | -1.96 | 0.00 |
| ENSG00000204950 | 123.77 | 1.37 | 0.01 | ENSG00000064655 | 119.03 | -1.96 | 0.00 |
| ENSG00000174371 | 485.46 | 1.37 | 0.00 | ENSG00000101695 | 288.82 | -1.96 | 0.00 |
| ENSG00000198203 | 356.92 | 1.37 | 0.01 | ENSG00000186188 | 592.57 | -1.96 | 0.00 |
| ENSG00000241741 | 62.44 | 1.37 | 0.00 | ENSG00000211637 | 501.16 | -1.96 | 0.02 |
| ENSG00000136378 | 362.09 | 1.37 | 0.00 | ENSG00000244575 | 313.77 | -1.96 | 0.01 |
| ENSG00000130299 | 860.17 | 1.37 | 0.00 | ENSG00000166183 | 101.93 | -1.97 | 0.00 |
| ENSG00000198744 | 173.99 | 1.37 | 0.02 | ENSG00000189221 | 4449.39 | -1.97 | 0.00 |
| ENSG00000279789 | 76.32 | 1.37 | 0.00 | ENSG00000118507 | 408.83 | -1.97 | 0.00 |
| ENSG00000154743 | 700.74 | 1.37 | 0.00 | ENSG00000131386 | 96.42 | -1.97 | 0.00 |
| ENSG00000197785 | 1839.12 | 1.37 | 0.00 | ENSG00000137558 | 149.21 | -1.97 | 0.00 |
| ENSG00000228300 | 2308.63 | 1.37 | 0.00 | ENSG00000172349 | 286.65 | -1.97 | 0.00 |
| ENSG00000141682 | 415.89 | 1.37 | 0.00 | ENSG00000163586 | 18856.86 | -1.97 | 0.00 |
| ENSG00000165568 | 82.64 | 1.36 | 0.00 | ENSG00000116176 | 296.36 | -1.98 | 0.01 |
| ENSG00000156697 | 1755.90 | 1.36 | 0.00 | ENSG00000115556 | 113.93 | -1.98 | 0.00 |
| ENSG00000124571 | 3027.98 | 1.36 | 0.00 | ENSG00000079335 | 239.02 | -1.98 | 0.00 |
| ENSG00000125319 | 267.81 | 1.36 | 0.00 | ENSG00000168229 | 116.12 | -1.98 | 0.03 |
| ENSG00000279495 | 99.77 | 1.36 | 0.00 | ENSG00000149557 | 193.88 | -1.98 | 0.00 |
| ENSG00000102384 | 288.38 | 1.36 | 0.00 | ENSG00000211598 | 2938.94 | -1.98 | 0.00 |
| ENSG00000237214 | 108.53 | 1.36 | 0.00 | ENSG00000187955 | 1437.42 | -1.99 | 0.00 |
| ENSG00000143067 | 353.55 | 1.36 | 0.00 | ENSG00000275395 | 27725.40 | -1.99 | 0.03 |
| ENSG00000219159 | 58.49 | 1.36 | 0.05 | ENSG00000172348 | 333.46 | -1.99 | 0.00 |
| ENSG00000163931 | 17378.78 | 1.36 | 0.00 | ENSG00000211649 | 330.87 | -1.99 | 0.02 |
| ENSG00000240065 | 2781.59 | 1.36 | 0.00 | ENSG00000071205 | 299.05 | -1.99 | 0.00 |
| ENSG00000170689 | 4768.75 | 1.36 | 0.00 | ENSG00000120658 | 53.84 | -1.99 | 0.00 |
| ENSG00000105281 | 10572.86 | 1.35 | 0.00 | ENSG00000128872 | 320.88 | -1.99 | 0.00 |
| ENSG00000185730 | 376.85 | 1.35 | 0.00 | ENSG00000109705 | 52.94 | -1.99 | 0.00 |
| ENSG00000027869 | 303.41 | 1.35 | 0.00 | ENSG00000149218 | 2017.49 | -1.99 | 0.00 |
| ENSG00000101182 | 12936.68 | 1.35 | 0.00 | ENSG00000150995 | 492.94 | -2.00 | 0.00 |
| ENSG00000101945 | 822.08 | 1.35 | 0.00 | ENSG00000211942 | 92.87 | -2.00 | 0.02 |
| ENSG00000161692 | 388.79 | 1.35 | 0.00 | ENSG00000157111 | 556.30 | -2.00 | 0.00 |
| ENSG00000158806 | 69.92 | 1.35 | 0.00 | ENSG00000178695 | 2052.78 | -2.00 | 0.00 |
| ENSG00000267709 | 59.33 | 1.35 | 0.01 | ENSG00000197629 | 892.44 | -2.00 | 0.00 |
| ENSG00000197859 | 370.97 | 1.35 | 0.00 | ENSG00000147027 | 572.07 | -2.00 | 0.00 |
| ENSG00000132661 | 1191.38 | 1.35 | 0.00 | ENSG00000211934 | 399.92 | -2.00 | 0.01 |
| ENSG00000111799 | 7628.80 | 1.35 | 0.00 | ENSG00000256643 | 84.53 | -2.00 | 0.00 |
| ENSG00000214279 | 50.92 | 1.35 | 0.00 | ENSG00000079102 | 116.39 | -2.00 | 0.00 |
| ENSG00000103148 | 2055.63 | 1.35 | 0.00 | ENSG00000129595 | 101.96 | -2.00 | 0.00 |
| ENSG00000105948 | 336.58 | 1.35 | 0.00 | ENSG00000100307 | 775.60 | -2.00 | 0.00 |
| ENSG00000174791 | 959.98 | 1.34 | 0.00 | ENSG00000188921 | 215.42 | -2.01 | 0.00 |
| ENSG00000135111 | 1236.46 | 1.34 | 0.00 | ENSG00000154153 | 1194.61 | -2.01 | 0.00 |
| ENSG00000105982 | 174.41 | 1.34 | 0.00 | ENSG00000100321 | 172.72 | -2.01 | 0.00 |
| ENSG00000179862 | 465.70 | 1.34 | 0.01 | ENSG00000141447 | 631.08 | -2.01 | 0.00 |
| ENSG00000178093 | 85.54 | 1.34 | 0.00 | ENSG00000106772 | 2006.27 | -2.01 | 0.00 |
| ENSG00000175264 | 112.84 | 1.34 | 0.00 | ENSG00000254827 | 284.79 | -2.01 | 0.00 |
| ENSG00000171045 | 786.70 | 1.34 | 0.00 | ENSG00000176928 | 72.75 | -2.02 | 0.00 |
| ENSG00000101003 | 1166.38 | 1.34 | 0.00 | ENSG00000143816 | 89.41 | -2.02 | 0.00 |
| ENSG00000142945 | 1377.33 | 1.34 | 0.00 | ENSG00000178462 | 250.38 | -2.02 | 0.00 |
| ENSG00000165661 | 1210.77 | 1.34 | 0.00 | ENSG00000057704 | 919.52 | -2.02 | 0.00 |
| ENSG00000175792 | 2722.33 | 1.34 | 0.00 | ENSG00000184828 | 702.35 | -2.02 | 0.00 |
| ENSG00000186603 | 899.67 | 1.34 | 0.00 | ENSG00000153064 | 100.90 | -2.02 | 0.00 |
| ENSG00000073111 | 3681.78 | 1.34 | 0.00 | ENSG00000163683 | 2452.56 | -2.02 | 0.00 |
| ENSG00000101361 | 5881.86 | 1.34 | 0.00 | ENSG00000158315 | 375.97 | -2.02 | 0.00 |
| ENSG00000106628 | 6093.96 | 1.34 | 0.00 | ENSG00000125845 | 679.59 | -2.02 | 0.00 |
| ENSG00000167771 | 181.16 | 1.34 | 0.00 | ENSG00000044524 | 205.41 | -2.02 | 0.00 |
| ENSG00000163485 | 72.52 | 1.34 | 0.00 | ENSG00000116833 | 479.44 | -2.02 | 0.00 |
| ENSG00000261326 | 143.52 | 1.33 | 0.00 | ENSG00000170703 | 97.22 | -2.02 | 0.00 |
| ENSG00000060491 | 2721.23 | 1.33 | 0.00 | ENSG00000007216 | 546.75 | -2.02 | 0.01 |
| ENSG00000132182 | 3681.98 | 1.33 | 0.00 | ENSG00000171791 | 289.50 | -2.02 | 0.00 |
| ENSG00000226332 | 67.86 | 1.33 | 0.00 | ENSG00000100490 | 65.97 | -2.02 | 0.00 |
| ENSG00000111206 | 2305.48 | 1.33 | 0.00 | ENSG00000211947 | 479.64 | -2.03 | 0.01 |
| ENSG00000109084 | 2561.17 | 1.33 | 0.00 | ENSG00000169554 | 711.88 | -2.03 | 0.00 |
| ENSG00000140988 | 65211.82 | 1.33 | 0.00 | ENSG00000234456 | 166.36 | -2.03 | 0.00 |
| ENSG00000205903 | 1802.69 | 1.33 | 0.00 | ENSG00000185567 | 1139.46 | -2.03 | 0.00 |
| ENSG00000070669 | 2151.01 | 1.33 | 0.00 | ENSG00000091656 | 79.20 | -2.03 | 0.00 |
| ENSG00000040608 | 353.92 | 1.33 | 0.00 | ENSG00000162949 | 389.09 | -2.03 | 0.00 |
| ENSG00000269131 | 332.44 | 1.32 | 0.00 | ENSG00000026103 | 538.43 | -2.03 | 0.00 |
| ENSG00000261685 | 564.68 | 1.32 | 0.03 | ENSG00000130518 | 66.52 | -2.03 | 0.00 |
| ENSG00000117834 | 157.31 | 1.32 | 0.02 | ENSG00000131018 | 635.92 | -2.04 | 0.00 |
| ENSG00000083635 | 614.78 | 1.32 | 0.00 | ENSG00000243264 | 91.92 | -2.04 | 0.01 |
| ENSG00000081181 | 148.34 | 1.32 | 0.00 | ENSG00000013297 | 63.07 | -2.04 | 0.00 |
| ENSG00000163516 | 1627.69 | 1.32 | 0.00 | ENSG00000112208 | 446.78 | -2.04 | 0.00 |
| ENSG00000126368 | 964.69 | 1.32 | 0.00 | ENSG00000180044 | 87.91 | -2.04 | 0.00 |
| ENSG00000068438 | 1949.70 | 1.32 | 0.00 | ENSG00000142875 | 2017.05 | -2.05 | 0.00 |
| ENSG00000037897 | 838.05 | 1.32 | 0.00 | ENSG00000121807 | 66.69 | -2.05 | 0.00 |
| ENSG00000118690 | 145.16 | 1.32 | 0.00 | ENSG00000170271 | 599.22 | -2.05 | 0.00 |
| ENSG00000142623 | 91.06 | 1.32 | 0.03 | ENSG00000141574 | 825.49 | -2.05 | 0.00 |
| ENSG00000126249 | 411.88 | 1.32 | 0.00 | ENSG00000132561 | 1490.06 | -2.06 | 0.00 |
| ENSG00000101084 | 2400.34 | 1.31 | 0.00 | ENSG00000140297 | 3412.96 | -2.06 | 0.00 |
| ENSG00000137343 | 589.38 | 1.31 | 0.00 | ENSG00000224650 | 706.41 | -2.06 | 0.01 |
| ENSG00000204946 | 732.36 | 1.31 | 0.00 | ENSG00000169946 | 74.45 | -2.06 | 0.00 |
| ENSG00000171680 | 1180.59 | 1.31 | 0.00 | ENSG00000040199 | 694.00 | -2.06 | 0.00 |
| ENSG00000157227 | 9843.54 | 1.31 | 0.00 | ENSG00000012223 | 184.75 | -2.06 | 0.00 |
| ENSG00000110104 | 1441.80 | 1.31 | 0.00 | ENSG00000023902 | 932.46 | -2.07 | 0.00 |
| ENSG00000187796 | 170.09 | 1.31 | 0.02 | ENSG00000175356 | 171.34 | -2.07 | 0.00 |
| ENSG00000103254 | 780.01 | 1.31 | 0.00 | ENSG00000197565 | 53.13 | -2.08 | 0.00 |
| ENSG00000176809 | 236.48 | 1.31 | 0.00 | ENSG00000148180 | 13513.21 | -2.08 | 0.00 |
| ENSG00000204149 | 213.38 | 1.31 | 0.00 | ENSG00000162460 | 98.67 | -2.08 | 0.00 |
| ENSG00000187775 | 95.22 | 1.31 | 0.00 | ENSG00000144824 | 377.56 | -2.08 | 0.00 |
| ENSG00000061656 | 260.28 | 1.31 | 0.00 | ENSG00000278196 | 543.77 | -2.08 | 0.01 |
| ENSG00000205560 | 53.73 | 1.31 | 0.00 | ENSG00000135437 | 68.35 | -2.08 | 0.00 |
| ENSG00000133316 | 1373.29 | 1.31 | 0.00 | ENSG00000188641 | 297.29 | -2.08 | 0.00 |
| ENSG00000140263 | 1764.87 | 1.31 | 0.00 | ENSG00000109819 | 420.50 | -2.08 | 0.00 |
| ENSG00000162591 | 719.13 | 1.31 | 0.01 | ENSG00000262655 | 1947.54 | -2.08 | 0.00 |
| ENSG00000225131 | 83.35 | 1.31 | 0.00 | ENSG00000164850 | 143.25 | -2.09 | 0.00 |
| ENSG00000176153 | 27361.88 | 1.31 | 0.00 | ENSG00000099139 | 496.73 | -2.09 | 0.00 |
| ENSG00000130706 | 6840.27 | 1.30 | 0.00 | ENSG00000211650 | 132.58 | -2.09 | 0.02 |
| ENSG00000075218 | 722.90 | 1.30 | 0.00 | ENSG00000099864 | 301.65 | -2.09 | 0.00 |
| ENSG00000092445 | 515.01 | 1.30 | 0.00 | ENSG00000137265 | 153.04 | -2.09 | 0.00 |
| ENSG00000125898 | 743.77 | 1.30 | 0.00 | ENSG00000144063 | 431.85 | -2.10 | 0.00 |
| ENSG00000101181 | 1950.61 | 1.30 | 0.00 | ENSG00000171451 | 57.40 | -2.10 | 0.00 |
| ENSG00000168061 | 750.46 | 1.30 | 0.00 | ENSG00000118526 | 185.34 | -2.11 | 0.00 |
| ENSG00000162769 | 1007.98 | 1.30 | 0.00 | ENSG00000241755 | 482.30 | -2.11 | 0.01 |
| ENSG00000171552 | 5956.48 | 1.30 | 0.00 | ENSG00000114166 | 377.69 | -2.11 | 0.00 |
| ENSG00000131591 | 662.20 | 1.30 | 0.00 | ENSG00000149451 | 168.80 | -2.11 | 0.00 |
| ENSG00000106484 | 2762.67 | 1.30 | 0.00 | ENSG00000135916 | 21726.62 | -2.11 | 0.00 |
| ENSG00000213918 | 1056.03 | 1.30 | 0.00 | ENSG00000197872 | 187.31 | -2.11 | 0.00 |
| ENSG00000051341 | 424.85 | 1.30 | 0.00 | ENSG00000104723 | 274.79 | -2.12 | 0.00 |
| ENSG00000168542 | 62686.57 | 1.30 | 0.01 | ENSG00000166963 | 499.81 | -2.12 | 0.00 |
| ENSG00000173137 | 1074.58 | 1.29 | 0.00 | ENSG00000172159 | 306.12 | -2.12 | 0.00 |
| ENSG00000158062 | 693.37 | 1.29 | 0.00 | ENSG00000172137 | 99.59 | -2.12 | 0.00 |
| ENSG00000279407 | 88.05 | 1.29 | 0.00 | ENSG00000182575 | 148.80 | -2.12 | 0.00 |
| ENSG00000152413 | 288.40 | 1.29 | 0.00 | ENSG00000072952 | 954.00 | -2.12 | 0.00 |
| ENSG00000174177 | 871.58 | 1.29 | 0.00 | ENSG00000169764 | 3983.37 | -2.13 | 0.00 |
| ENSG00000124217 | 966.27 | 1.29 | 0.00 | ENSG00000105851 | 100.47 | -2.13 | 0.00 |
| ENSG00000102221 | 888.96 | 1.29 | 0.00 | ENSG00000187288 | 85.80 | -2.13 | 0.00 |
| ENSG00000004777 | 363.84 | 1.29 | 0.00 | ENSG00000018408 | 965.00 | -2.13 | 0.00 |
| ENSG00000106683 | 1827.79 | 1.29 | 0.00 | ENSG00000163751 | 282.03 | -2.13 | 0.00 |
| ENSG00000204387 | 3497.89 | 1.29 | 0.00 | ENSG00000135424 | 622.49 | -2.13 | 0.00 |
| ENSG00000135919 | 3523.78 | 1.29 | 0.00 | ENSG00000112320 | 146.32 | -2.14 | 0.00 |
| ENSG00000091073 | 2473.02 | 1.29 | 0.00 | ENSG00000260244 | 223.91 | -2.14 | 0.00 |
| ENSG00000173621 | 1553.45 | 1.29 | 0.00 | ENSG00000259330 | 196.61 | -2.14 | 0.00 |
| ENSG00000186792 | 218.07 | 1.29 | 0.00 | ENSG00000166482 | 2153.26 | -2.14 | 0.00 |
| ENSG00000140937 | 1826.61 | 1.28 | 0.00 | ENSG00000099866 | 62.70 | -2.14 | 0.00 |
| ENSG00000184220 | 1009.16 | 1.28 | 0.00 | ENSG00000111341 | 2451.78 | -2.14 | 0.00 |
| ENSG00000165304 | 1148.87 | 1.28 | 0.00 | ENSG00000240864 | 361.16 | -2.14 | 0.00 |
| ENSG00000062822 | 1790.91 | 1.28 | 0.00 | ENSG00000123094 | 247.45 | -2.15 | 0.00 |
| ENSG00000104312 | 950.19 | 1.28 | 0.00 | ENSG00000137273 | 321.67 | -2.15 | 0.00 |
| ENSG00000093010 | 5602.42 | 1.28 | 0.00 | ENSG00000068615 | 338.65 | -2.15 | 0.00 |
| ENSG00000116691 | 1137.94 | 1.28 | 0.00 | ENSG00000150510 | 84.30 | -2.15 | 0.00 |
| ENSG00000259933 | 414.70 | 1.28 | 0.00 | ENSG00000211663 | 1019.48 | -2.15 | 0.00 |
| ENSG00000187653 | 300.05 | 1.28 | 0.01 | ENSG00000090530 | 535.58 | -2.15 | 0.00 |
| ENSG00000007171 | 1851.81 | 1.28 | 0.05 | ENSG00000211651 | 765.41 | -2.15 | 0.00 |
| ENSG00000160214 | 1718.35 | 1.28 | 0.00 | ENSG00000025423 | 121.59 | -2.15 | 0.00 |
| ENSG00000126001 | 2181.36 | 1.28 | 0.00 | ENSG00000071967 | 1615.73 | -2.15 | 0.00 |
| ENSG00000052749 | 2298.19 | 1.28 | 0.00 | ENSG00000187239 | 1426.68 | -2.15 | 0.00 |
| ENSG00000204394 | 5920.93 | 1.28 | 0.00 | ENSG00000176438 | 208.94 | -2.15 | 0.00 |
| ENSG00000077063 | 493.72 | 1.28 | 0.01 | ENSG00000223648 | 52.51 | -2.16 | 0.00 |
| ENSG00000137166 | 6135.72 | 1.28 | 0.00 | ENSG00000158125 | 885.37 | -2.16 | 0.00 |
| ENSG00000205403 | 624.30 | 1.28 | 0.01 | ENSG00000125148 | 2031.34 | -2.16 | 0.00 |
| ENSG00000116337 | 2149.19 | 1.28 | 0.00 | ENSG00000167701 | 475.04 | -2.16 | 0.00 |
| ENSG00000158286 | 301.12 | 1.28 | 0.00 | ENSG00000196557 | 954.41 | -2.16 | 0.00 |
| ENSG00000204388 | 4873.25 | 1.28 | 0.00 | ENSG00000181061 | 2391.43 | -2.17 | 0.00 |
| ENSG00000117385 | 1016.39 | 1.28 | 0.00 | ENSG00000139117 | 246.54 | -2.17 | 0.00 |
| ENSG00000170779 | 1052.70 | 1.27 | 0.00 | ENSG00000239951 | 2403.05 | -2.17 | 0.00 |
| ENSG00000171453 | 1600.10 | 1.27 | 0.00 | ENSG00000225698 | 256.79 | -2.17 | 0.01 |
| ENSG00000163121 | 77.97 | 1.27 | 0.02 | ENSG00000211974 | 60.91 | -2.17 | 0.03 |
| ENSG00000159259 | 559.86 | 1.27 | 0.00 | ENSG00000137860 | 344.97 | -2.17 | 0.02 |
| ENSG00000163584 | 1611.75 | 1.27 | 0.00 | ENSG00000140285 | 240.01 | -2.17 | 0.00 |
| ENSG00000130222 | 405.45 | 1.27 | 0.01 | ENSG00000171724 | 96.59 | -2.18 | 0.00 |
| ENSG00000135763 | 960.00 | 1.27 | 0.00 | ENSG00000183508 | 834.29 | -2.18 | 0.00 |
| ENSG00000165689 | 2032.83 | 1.27 | 0.00 | ENSG00000211673 | 411.76 | -2.18 | 0.00 |
| ENSG00000105472 | 505.03 | 1.27 | 0.00 | ENSG00000177675 | 257.19 | -2.18 | 0.00 |
| ENSG00000101670 | 1188.32 | 1.27 | 0.00 | ENSG00000182168 | 128.36 | -2.18 | 0.00 |
| ENSG00000141293 | 398.26 | 1.27 | 0.00 | ENSG00000095110 | 679.48 | -2.19 | 0.00 |
| ENSG00000128059 | 866.26 | 1.27 | 0.00 | ENSG00000163412 | 724.80 | -2.19 | 0.00 |
| ENSG00000184207 | 1346.62 | 1.27 | 0.00 | ENSG00000173198 | 54.06 | -2.19 | 0.00 |
| ENSG00000168237 | 1056.07 | 1.27 | 0.00 | ENSG00000122756 | 64.70 | -2.19 | 0.01 |
| ENSG00000137573 | 6095.42 | 1.27 | 0.02 | ENSG00000169508 | 249.42 | -2.19 | 0.00 |
| ENSG00000136270 | 5683.19 | 1.27 | 0.00 | ENSG00000073711 | 277.90 | -2.20 | 0.00 |
| ENSG00000166508 | 9493.84 | 1.27 | 0.00 | ENSG00000122224 | 51.89 | -2.20 | 0.00 |
| ENSG00000185324 | 2665.98 | 1.27 | 0.00 | ENSG00000139970 | 92.86 | -2.21 | 0.00 |
| ENSG00000139880 | 445.05 | 1.27 | 0.00 | ENSG00000114790 | 208.86 | -2.21 | 0.00 |
| ENSG00000109736 | 3778.93 | 1.27 | 0.00 | ENSG00000167779 | 396.54 | -2.21 | 0.00 |
| ENSG00000071894 | 5988.21 | 1.26 | 0.00 | ENSG00000227051 | 222.74 | -2.22 | 0.00 |
| ENSG00000160284 | 395.84 | 1.26 | 0.01 | ENSG00000185303 | 78.75 | -2.22 | 0.00 |
| ENSG00000135749 | 529.41 | 1.26 | 0.00 | ENSG00000048052 | 157.99 | -2.22 | 0.00 |
| ENSG00000230207 | 62.79 | 1.26 | 0.00 | ENSG00000172572 | 986.47 | -2.22 | 0.00 |
| ENSG00000165730 | 204.39 | 1.26 | 0.00 | ENSG00000171659 | 140.87 | -2.22 | 0.00 |
| ENSG00000273373 | 129.95 | 1.26 | 0.00 | ENSG00000187097 | 2473.99 | -2.22 | 0.00 |
| ENSG00000141994 | 1624.38 | 1.26 | 0.00 | ENSG00000164236 | 61.93 | -2.22 | 0.00 |
| ENSG00000197070 | 3891.48 | 1.26 | 0.00 | ENSG00000231292 | 57.15 | -2.23 | 0.00 |
| ENSG00000012211 | 665.93 | 1.26 | 0.00 | ENSG00000124440 | 96.40 | -2.23 | 0.00 |
| ENSG00000137474 | 391.76 | 1.26 | 0.00 | ENSG00000101188 | 97.69 | -2.23 | 0.01 |
| ENSG00000239415 | 81.59 | 1.26 | 0.00 | ENSG00000136274 | 63.57 | -2.23 | 0.00 |
| ENSG00000148335 | 1144.83 | 1.26 | 0.00 | ENSG00000197928 | 62.74 | -2.24 | 0.00 |
| ENSG00000116670 | 919.54 | 1.26 | 0.00 | ENSG00000133104 | 354.10 | -2.24 | 0.00 |
| ENSG00000259994 | 68.06 | 1.26 | 0.00 | ENSG00000178031 | 156.96 | -2.24 | 0.00 |
| ENSG00000242193 | 1905.78 | 1.26 | 0.00 | ENSG00000110777 | 290.05 | -2.24 | 0.00 |
| ENSG00000272405 | 1214.31 | 1.26 | 0.00 | ENSG00000169851 | 380.00 | -2.24 | 0.00 |
| ENSG00000105202 | 9237.30 | 1.26 | 0.00 | ENSG00000240382 | 360.96 | -2.24 | 0.00 |
| ENSG00000146733 | 968.03 | 1.26 | 0.00 | ENSG00000173467 | 1301.34 | -2.25 | 0.00 |
| ENSG00000229167 | 101.08 | 1.25 | 0.00 | ENSG00000137941 | 92.18 | -2.25 | 0.00 |
| ENSG00000278192 | 180.54 | 1.25 | 0.00 | ENSG00000211640 | 503.93 | -2.25 | 0.00 |
| ENSG00000101442 | 701.07 | 1.25 | 0.00 | ENSG00000117472 | 12751.96 | -2.25 | 0.00 |
| ENSG00000188878 | 137.95 | 1.25 | 0.00 | ENSG00000203710 | 62.10 | -2.25 | 0.00 |
| ENSG00000049449 | 5548.29 | 1.25 | 0.00 | ENSG00000164116 | 479.24 | -2.26 | 0.00 |
| ENSG00000180822 | 824.48 | 1.25 | 0.00 | ENSG00000151882 | 1147.81 | -2.26 | 0.00 |
| ENSG00000122678 | 944.58 | 1.25 | 0.00 | ENSG00000213088 | 280.54 | -2.26 | 0.00 |
| ENSG00000107833 | 1495.07 | 1.25 | 0.00 | ENSG00000117091 | 299.87 | -2.26 | 0.00 |
| ENSG00000137834 | 810.73 | 1.25 | 0.00 | ENSG00000113448 | 551.52 | -2.26 | 0.00 |
| ENSG00000064651 | 16677.44 | 1.25 | 0.00 | ENSG00000148541 | 53.94 | -2.26 | 0.00 |
| ENSG00000204618 | 211.03 | 1.25 | 0.00 | ENSG00000140416 | 12895.79 | -2.27 | 0.00 |
| ENSG00000071564 | 5101.64 | 1.25 | 0.00 | ENSG00000074410 | 3910.58 | -2.27 | 0.00 |
| ENSG00000261123 | 343.85 | 1.25 | 0.00 | ENSG00000144642 | 136.77 | -2.27 | 0.00 |
| ENSG00000162639 | 686.29 | 1.25 | 0.00 | ENSG00000183036 | 170.69 | -2.27 | 0.00 |
| ENSG00000163874 | 1957.16 | 1.25 | 0.00 | ENSG00000205683 | 59.17 | -2.28 | 0.00 |
| ENSG00000143228 | 588.19 | 1.25 | 0.00 | ENSG00000211445 | 1345.14 | -2.28 | 0.00 |
| ENSG00000106263 | 12703.96 | 1.25 | 0.00 | ENSG00000092421 | 951.94 | -2.28 | 0.00 |
| ENSG00000102125 | 1241.01 | 1.25 | 0.00 | ENSG00000133328 | 50.55 | -2.29 | 0.00 |
| ENSG00000103064 | 847.45 | 1.24 | 0.00 | ENSG00000119138 | 876.72 | -2.29 | 0.00 |
| ENSG00000231345 | 82.27 | 1.24 | 0.04 | ENSG00000011465 | 8601.64 | -2.29 | 0.00 |
| ENSG00000141101 | 2925.19 | 1.24 | 0.00 | ENSG00000134198 | 469.19 | -2.29 | 0.00 |
| ENSG00000144381 | 23417.93 | 1.24 | 0.00 | ENSG00000126860 | 140.27 | -2.29 | 0.00 |
| ENSG00000162129 | 729.97 | 1.24 | 0.00 | ENSG00000133687 | 265.36 | -2.30 | 0.00 |
| ENSG00000134222 | 497.39 | 1.24 | 0.00 | ENSG00000105609 | 118.51 | -2.30 | 0.00 |
| ENSG00000130305 | 1583.80 | 1.24 | 0.00 | ENSG00000117215 | 69.56 | -2.31 | 0.00 |
| ENSG00000244560 | 57.00 | 1.24 | 0.00 | ENSG00000078804 | 1819.22 | -2.31 | 0.00 |
| ENSG00000244398 | 542.68 | 1.24 | 0.00 | ENSG00000180543 | 146.34 | -2.32 | 0.00 |
| ENSG00000125726 | 52.70 | 1.24 | 0.03 | ENSG00000091986 | 1978.75 | -2.32 | 0.00 |
| ENSG00000196497 | 235.24 | 1.24 | 0.00 | ENSG00000109339 | 81.41 | -2.32 | 0.00 |
| ENSG00000139514 | 6167.94 | 1.24 | 0.00 | ENSG00000127990 | 293.19 | -2.32 | 0.00 |
| ENSG00000104522 | 7179.24 | 1.24 | 0.00 | ENSG00000211968 | 80.00 | -2.32 | 0.00 |
| ENSG00000224578 | 207.98 | 1.24 | 0.00 | ENSG00000168280 | 111.70 | -2.32 | 0.00 |
| ENSG00000119969 | 807.86 | 1.24 | 0.00 | ENSG00000166391 | 816.15 | -2.32 | 0.00 |
| ENSG00000279738 | 92.91 | 1.24 | 0.00 | ENSG00000159958 | 74.24 | -2.32 | 0.00 |
| ENSG00000203876 | 104.31 | 1.23 | 0.00 | ENSG00000198865 | 58.65 | -2.33 | 0.00 |
| ENSG00000100726 | 2273.45 | 1.23 | 0.00 | ENSG00000173267 | 78.78 | -2.33 | 0.00 |
| ENSG00000198221 | 73.23 | 1.23 | 0.00 | ENSG00000166402 | 77.49 | -2.33 | 0.00 |
| ENSG00000142694 | 415.91 | 1.23 | 0.01 | ENSG00000108823 | 86.80 | -2.33 | 0.00 |
| ENSG00000183763 | 381.21 | 1.23 | 0.00 | ENSG00000197256 | 2485.97 | -2.33 | 0.00 |
| ENSG00000117399 | 2903.45 | 1.23 | 0.00 | ENSG00000268388 | 542.64 | -2.34 | 0.00 |
| ENSG00000221978 | 3230.96 | 1.23 | 0.00 | ENSG00000049246 | 302.01 | -2.34 | 0.00 |
| ENSG00000108107 | 43276.63 | 1.23 | 0.00 | ENSG00000167080 | 220.55 | -2.34 | 0.02 |
| ENSG00000197119 | 971.36 | 1.23 | 0.00 | ENSG00000215218 | 56.19 | -2.35 | 0.00 |
| ENSG00000168918 | 2199.71 | 1.23 | 0.00 | ENSG00000255248 | 188.17 | -2.35 | 0.00 |
| ENSG00000169972 | 661.22 | 1.23 | 0.00 | ENSG00000144619 | 93.19 | -2.36 | 0.00 |
| ENSG00000165271 | 3109.25 | 1.23 | 0.00 | ENSG00000154277 | 206.36 | -2.36 | 0.00 |
| ENSG00000149636 | 1240.31 | 1.23 | 0.00 | ENSG00000183023 | 353.29 | -2.36 | 0.00 |
| ENSG00000135372 | 3607.71 | 1.23 | 0.00 | ENSG00000177098 | 85.10 | -2.36 | 0.00 |
| ENSG00000187678 | 1957.58 | 1.23 | 0.00 | ENSG00000105270 | 460.19 | -2.37 | 0.00 |
| ENSG00000176022 | 1040.07 | 1.23 | 0.00 | ENSG00000185432 | 1775.03 | -2.37 | 0.00 |
| ENSG00000100170 | 3597.31 | 1.23 | 0.00 | ENSG00000150764 | 563.98 | -2.37 | 0.00 |
| ENSG00000188486 | 3276.86 | 1.23 | 0.00 | ENSG00000124942 | 17593.63 | -2.37 | 0.00 |
| ENSG00000231806 | 70.88 | 1.22 | 0.00 | ENSG00000197766 | 1050.78 | -2.37 | 0.00 |
| ENSG00000134569 | 3194.50 | 1.22 | 0.02 | ENSG00000171431 | 12375.99 | -2.38 | 0.00 |
| ENSG00000080839 | 768.52 | 1.22 | 0.00 | ENSG00000178473 | 53.05 | -2.38 | 0.00 |
| ENSG00000214706 | 3481.74 | 1.22 | 0.00 | ENSG00000111863 | 493.44 | -2.38 | 0.00 |
| ENSG00000111331 | 3386.50 | 1.22 | 0.00 | ENSG00000107186 | 257.44 | -2.38 | 0.00 |
| ENSG00000127580 | 693.50 | 1.22 | 0.00 | ENSG00000240671 | 64.42 | -2.39 | 0.00 |
| ENSG00000187801 | 84.04 | 1.22 | 0.00 | ENSG00000122694 | 698.82 | -2.39 | 0.00 |
| ENSG00000123191 | 1436.60 | 1.22 | 0.00 | ENSG00000134769 | 189.81 | -2.39 | 0.00 |
| ENSG00000235065 | 81.94 | 1.22 | 0.01 | ENSG00000123096 | 445.27 | -2.39 | 0.00 |
| ENSG00000171316 | 2240.92 | 1.22 | 0.00 | ENSG00000125775 | 1885.03 | -2.39 | 0.00 |
| ENSG00000142330 | 981.98 | 1.22 | 0.00 | ENSG00000188175 | 1070.13 | -2.40 | 0.01 |
| ENSG00000233937 | 89.09 | 1.22 | 0.00 | ENSG00000188738 | 80.53 | -2.40 | 0.00 |
| ENSG00000228544 | 106.96 | 1.22 | 0.00 | ENSG00000154188 | 112.68 | -2.40 | 0.00 |
| ENSG00000136877 | 2866.00 | 1.22 | 0.00 | ENSG00000115252 | 140.41 | -2.40 | 0.00 |
| ENSG00000181638 | 515.80 | 1.22 | 0.00 | ENSG00000129116 | 4494.38 | -2.40 | 0.00 |
| ENSG00000133612 | 3243.05 | 1.22 | 0.00 | ENSG00000104435 | 253.35 | -2.41 | 0.00 |
| ENSG00000133740 | 668.80 | 1.22 | 0.00 | ENSG00000080644 | 86.37 | -2.41 | 0.00 |
| ENSG00000182240 | 6070.02 | 1.22 | 0.00 | ENSG00000137872 | 299.38 | -2.41 | 0.00 |
| ENSG00000249115 | 809.62 | 1.22 | 0.00 | ENSG00000277586 | 63.10 | -2.41 | 0.02 |
| ENSG00000196839 | 421.21 | 1.22 | 0.00 | ENSG00000163637 | 240.90 | -2.41 | 0.00 |
| ENSG00000196123 | 605.25 | 1.22 | 0.00 | ENSG00000269936 | 313.63 | -2.41 | 0.00 |
| ENSG00000160999 | 230.53 | 1.22 | 0.00 | ENSG00000162409 | 148.90 | -2.41 | 0.00 |
| ENSG00000123136 | 4439.18 | 1.21 | 0.00 | ENSG00000165449 | 418.59 | -2.41 | 0.00 |
| ENSG00000183682 | 58.56 | 1.21 | 0.01 | ENSG00000244734 | 771.51 | -2.42 | 0.00 |
| ENSG00000124181 | 3528.29 | 1.21 | 0.00 | ENSG00000064309 | 203.76 | -2.42 | 0.00 |
| ENSG00000125835 | 11707.43 | 1.21 | 0.00 | ENSG00000112182 | 54.71 | -2.42 | 0.00 |
| ENSG00000205078 | 73.75 | 1.21 | 0.04 | ENSG00000188906 | 122.69 | -2.42 | 0.00 |
| ENSG00000254290 | 892.67 | 1.21 | 0.00 | ENSG00000166816 | 417.59 | -2.43 | 0.00 |
| ENSG00000175592 | 521.27 | 1.21 | 0.01 | ENSG00000166770 | 62.56 | -2.43 | 0.00 |
| ENSG00000104369 | 1112.10 | 1.21 | 0.00 | ENSG00000154822 | 229.79 | -2.44 | 0.00 |
| ENSG00000130511 | 2400.32 | 1.21 | 0.00 | ENSG00000069535 | 317.71 | -2.44 | 0.00 |
| ENSG00000099904 | 1548.46 | 1.21 | 0.00 | ENSG00000064787 | 1761.91 | -2.44 | 0.00 |
| ENSG00000125046 | 156.59 | 1.21 | 0.04 | ENSG00000108852 | 76.85 | -2.44 | 0.00 |
| ENSG00000143476 | 800.39 | 1.21 | 0.00 | ENSG00000166510 | 366.54 | -2.44 | 0.00 |
| ENSG00000143847 | 57.37 | 1.21 | 0.02 | ENSG00000174236 | 68.35 | -2.44 | 0.00 |
| ENSG00000126453 | 1020.01 | 1.21 | 0.00 | ENSG00000113580 | 540.54 | -2.44 | 0.00 |
| ENSG00000169750 | 176.09 | 1.21 | 0.01 | ENSG00000136826 | 3255.20 | -2.44 | 0.00 |
| ENSG00000101391 | 1503.64 | 1.21 | 0.00 | ENSG00000211890 | 28382.37 | -2.45 | 0.00 |
| ENSG00000264577 | 61.61 | 1.21 | 0.00 | ENSG00000145335 | 74.67 | -2.46 | 0.00 |
| ENSG00000139266 | 832.04 | 1.21 | 0.00 | ENSG00000154734 | 963.58 | -2.46 | 0.00 |
| ENSG00000185522 | 129.08 | 1.21 | 0.00 | ENSG00000113303 | 405.99 | -2.46 | 0.00 |
| ENSG00000115163 | 421.45 | 1.21 | 0.00 | ENSG00000036672 | 143.07 | -2.46 | 0.00 |
| ENSG00000270189 | 190.25 | 1.21 | 0.00 | ENSG00000123243 | 827.56 | -2.46 | 0.00 |
| ENSG00000188157 | 7752.99 | 1.21 | 0.00 | ENSG00000113805 | 61.41 | -2.46 | 0.00 |
| ENSG00000273045 | 170.75 | 1.21 | 0.00 | ENSG00000165821 | 89.92 | -2.47 | 0.00 |
| ENSG00000125485 | 800.31 | 1.21 | 0.00 | ENSG00000198932 | 146.21 | -2.47 | 0.00 |
| ENSG00000132749 | 444.86 | 1.21 | 0.00 | ENSG00000106571 | 184.50 | -2.48 | 0.00 |
| ENSG00000117724 | 2395.82 | 1.20 | 0.00 | ENSG00000115616 | 748.47 | -2.48 | 0.00 |
| ENSG00000106686 | 115.88 | 1.20 | 0.00 | ENSG00000144712 | 56.83 | -2.48 | 0.00 |
| ENSG00000126457 | 7658.71 | 1.20 | 0.00 | ENSG00000165457 | 261.40 | -2.48 | 0.00 |
| ENSG00000261061 | 181.97 | 1.20 | 0.00 | ENSG00000082497 | 145.88 | -2.48 | 0.00 |
| ENSG00000101190 | 823.62 | 1.20 | 0.00 | ENSG00000205795 | 113.17 | -2.48 | 0.00 |
| ENSG00000099326 | 904.47 | 1.20 | 0.00 | ENSG00000143995 | 320.47 | -2.49 | 0.00 |
| ENSG00000242125 | 1391.05 | 1.20 | 0.00 | ENSG00000266200 | 159.89 | -2.49 | 0.00 |
| ENSG00000169696 | 1801.79 | 1.20 | 0.00 | ENSG00000196092 | 123.21 | -2.49 | 0.00 |
| ENSG00000176485 | 1314.83 | 1.20 | 0.01 | ENSG00000122786 | 8261.45 | -2.49 | 0.00 |
| ENSG00000204271 | 163.42 | 1.20 | 0.00 | ENSG00000145147 | 220.39 | -2.49 | 0.00 |
| ENSG00000168970 | 117.04 | 1.20 | 0.00 | ENSG00000146267 | 79.30 | -2.49 | 0.00 |
| ENSG00000168612 | 916.53 | 1.20 | 0.00 | ENSG00000105784 | 62.05 | -2.49 | 0.00 |
| ENSG00000223959 | 563.01 | 1.20 | 0.00 | ENSG00000198121 | 356.93 | -2.49 | 0.00 |
| ENSG00000261659 | 63.61 | 1.20 | 0.00 | ENSG00000073712 | 925.42 | -2.50 | 0.00 |
| ENSG00000065057 | 1473.25 | 1.20 | 0.00 | ENSG00000138678 | 296.08 | -2.50 | 0.00 |
| ENSG00000187608 | 1707.24 | 1.20 | 0.01 | ENSG00000185274 | 69.04 | -2.51 | 0.00 |
| ENSG00000218426 | 514.94 | 1.20 | 0.01 | ENSG00000181804 | 122.04 | -2.51 | 0.00 |
| ENSG00000125630 | 1538.02 | 1.20 | 0.00 | ENSG00000126947 | 173.20 | -2.51 | 0.00 |
| ENSG00000183397 | 61.27 | 1.20 | 0.00 | ENSG00000145287 | 1586.10 | -2.52 | 0.00 |
| ENSG00000136271 | 3590.55 | 1.20 | 0.00 | ENSG00000105369 | 344.64 | -2.52 | 0.00 |
| ENSG00000125247 | 1605.19 | 1.20 | 0.00 | ENSG00000154553 | 1088.38 | -2.52 | 0.00 |
| ENSG00000070756 | 76317.70 | 1.20 | 0.00 | ENSG00000186469 | 347.42 | -2.53 | 0.00 |
| ENSG00000162814 | 52.02 | 1.20 | 0.01 | ENSG00000090402 | 705.51 | -2.53 | 0.01 |
| ENSG00000101346 | 7269.46 | 1.19 | 0.00 | ENSG00000133710 | 304.56 | -2.53 | 0.00 |
| ENSG00000130158 | 3277.28 | 1.19 | 0.00 | ENSG00000153814 | 238.50 | -2.54 | 0.00 |
| ENSG00000260920 | 90.79 | 1.19 | 0.00 | ENSG00000124491 | 602.01 | -2.54 | 0.00 |
| ENSG00000121089 | 98.23 | 1.19 | 0.00 | ENSG00000164176 | 963.70 | -2.54 | 0.00 |
| ENSG00000276023 | 548.89 | 1.19 | 0.00 | ENSG00000112425 | 151.93 | -2.54 | 0.00 |
| ENSG00000125826 | 6282.91 | 1.19 | 0.00 | ENSG00000072163 | 1069.15 | -2.54 | 0.00 |
| ENSG00000120256 | 2567.35 | 1.19 | 0.00 | ENSG00000088854 | 209.21 | -2.55 | 0.00 |
| ENSG00000170638 | 2767.82 | 1.19 | 0.00 | ENSG00000179314 | 261.54 | -2.55 | 0.00 |
| ENSG00000171877 | 213.91 | 1.19 | 0.02 | ENSG00000160712 | 512.17 | -2.55 | 0.00 |
| ENSG00000204764 | 183.33 | 1.19 | 0.00 | ENSG00000170382 | 212.87 | -2.55 | 0.00 |
| ENSG00000184162 | 1035.39 | 1.19 | 0.00 | ENSG00000114455 | 1524.92 | -2.56 | 0.00 |
| ENSG00000186638 | 341.70 | 1.19 | 0.00 | ENSG00000243955 | 56.37 | -2.56 | 0.00 |
| ENSG00000258727 | 58.04 | 1.19 | 0.00 | ENSG00000173068 | 189.63 | -2.56 | 0.00 |
| ENSG00000165684 | 935.93 | 1.19 | 0.00 | ENSG00000143867 | 56.52 | -2.56 | 0.00 |
| ENSG00000138101 | 404.18 | 1.19 | 0.00 | ENSG00000147883 | 676.02 | -2.56 | 0.00 |
| ENSG00000164934 | 2531.55 | 1.19 | 0.00 | ENSG00000112175 | 85.52 | -2.56 | 0.00 |
| ENSG00000213553 | 752.32 | 1.19 | 0.02 | ENSG00000214814 | 229.80 | -2.57 | 0.00 |
| ENSG00000197406 | 229.33 | 1.19 | 0.05 | ENSG00000152580 | 68.35 | -2.57 | 0.00 |
| ENSG00000265148 | 98.68 | 1.19 | 0.00 | ENSG00000211638 | 388.10 | -2.57 | 0.00 |
| ENSG00000135632 | 1629.30 | 1.19 | 0.00 | ENSG00000160191 | 1010.88 | -2.58 | 0.00 |
| ENSG00000146411 | 315.58 | 1.19 | 0.02 | ENSG00000166250 | 499.57 | -2.58 | 0.00 |
| ENSG00000109089 | 666.22 | 1.19 | 0.00 | ENSG00000111728 | 62.97 | -2.59 | 0.00 |
| ENSG00000189343 | 539.57 | 1.18 | 0.01 | ENSG00000196924 | 32679.52 | -2.59 | 0.00 |
| ENSG00000186567 | 218.69 | 1.18 | 0.00 | ENSG00000177455 | 52.85 | -2.59 | 0.00 |
| ENSG00000106462 | 1250.15 | 1.18 | 0.00 | ENSG00000120885 | 2973.37 | -2.59 | 0.00 |
| ENSG00000112378 | 18027.48 | 1.18 | 0.00 | ENSG00000047457 | 154.49 | -2.59 | 0.00 |
| ENSG00000164818 | 2754.36 | 1.18 | 0.00 | ENSG00000243244 | 233.54 | -2.60 | 0.00 |
| ENSG00000163900 | 1465.40 | 1.18 | 0.00 | ENSG00000146938 | 51.78 | -2.60 | 0.00 |
| ENSG00000196411 | 5756.98 | 1.18 | 0.00 | ENSG00000109846 | 514.91 | -2.60 | 0.00 |
| ENSG00000167291 | 3098.89 | 1.18 | 0.00 | ENSG00000167723 | 59.71 | -2.60 | 0.00 |
| ENSG00000131747 | 5887.58 | 1.18 | 0.00 | ENSG00000151090 | 391.46 | -2.60 | 0.00 |
| ENSG00000147394 | 1054.32 | 1.18 | 0.00 | ENSG00000016490 | 7015.39 | -2.60 | 0.02 |
| ENSG00000148344 | 760.71 | 1.18 | 0.01 | ENSG00000182175 | 341.91 | -2.60 | 0.00 |
| ENSG00000213397 | 118.45 | 1.18 | 0.00 | ENSG00000134917 | 78.72 | -2.60 | 0.00 |
| ENSG00000111490 | 543.56 | 1.18 | 0.00 | ENSG00000173641 | 322.13 | -2.61 | 0.00 |
| ENSG00000203772 | 130.82 | 1.18 | 0.00 | ENSG00000156298 | 673.92 | -2.61 | 0.00 |
| ENSG00000154096 | 3723.64 | 1.18 | 0.00 | ENSG00000166025 | 698.19 | -2.61 | 0.00 |
| ENSG00000070404 | 868.02 | 1.18 | 0.01 | ENSG00000183778 | 1501.40 | -2.62 | 0.00 |
| ENSG00000169682 | 138.24 | 1.18 | 0.00 | ENSG00000150471 | 90.19 | -2.62 | 0.00 |
| ENSG00000169991 | 941.01 | 1.18 | 0.00 | ENSG00000188783 | 883.75 | -2.62 | 0.00 |
| ENSG00000136856 | 2054.03 | 1.18 | 0.00 | ENSG00000224189 | 303.01 | -2.63 | 0.00 |
| ENSG00000167157 | 99.24 | 1.18 | 0.03 | ENSG00000053438 | 120.11 | -2.63 | 0.00 |
| ENSG00000177225 | 2886.18 | 1.18 | 0.00 | ENSG00000066468 | 531.61 | -2.63 | 0.00 |
| ENSG00000068489 | 885.56 | 1.17 | 0.00 | ENSG00000189129 | 85.94 | -2.63 | 0.00 |
| ENSG00000124574 | 1582.21 | 1.17 | 0.00 | ENSG00000148516 | 700.44 | -2.64 | 0.00 |
| ENSG00000148362 | 1522.35 | 1.17 | 0.00 | ENSG00000159176 | 10479.70 | -2.64 | 0.00 |
| ENSG00000151490 | 2192.95 | 1.17 | 0.05 | ENSG00000119508 | 220.92 | -2.65 | 0.00 |
| ENSG00000172315 | 1677.14 | 1.17 | 0.00 | ENSG00000138685 | 140.17 | -2.65 | 0.00 |
| ENSG00000170909 | 107.13 | 1.17 | 0.00 | ENSG00000158467 | 3588.87 | -2.65 | 0.00 |
| ENSG00000178999 | 1203.94 | 1.17 | 0.00 | ENSG00000171408 | 57.25 | -2.65 | 0.00 |
| ENSG00000131697 | 604.94 | 1.17 | 0.00 | ENSG00000155980 | 67.53 | -2.65 | 0.00 |
| ENSG00000149658 | 3759.42 | 1.17 | 0.00 | ENSG00000131016 | 1002.75 | -2.65 | 0.00 |
| ENSG00000119333 | 4314.32 | 1.17 | 0.00 | ENSG00000166920 | 5071.25 | -2.66 | 0.00 |
| ENSG00000140104 | 394.16 | 1.17 | 0.00 | ENSG00000089199 | 141.36 | -2.66 | 0.00 |
| ENSG00000003147 | 2442.06 | 1.17 | 0.00 | ENSG00000005249 | 259.22 | -2.66 | 0.00 |
| ENSG00000145220 | 1116.96 | 1.17 | 0.00 | ENSG00000162614 | 414.75 | -2.66 | 0.00 |
| ENSG00000107338 | 964.75 | 1.17 | 0.00 | ENSG00000197321 | 3130.99 | -2.67 | 0.00 |
| ENSG00000183828 | 1337.01 | 1.17 | 0.00 | ENSG00000124205 | 384.04 | -2.67 | 0.00 |
| ENSG00000184575 | 3903.48 | 1.17 | 0.00 | ENSG00000128714 | 209.97 | -2.67 | 0.00 |
| ENSG00000145217 | 160.44 | 1.17 | 0.00 | ENSG00000172594 | 798.71 | -2.68 | 0.00 |
| ENSG00000188807 | 1100.67 | 1.17 | 0.00 | ENSG00000171533 | 70.72 | -2.68 | 0.00 |
| ENSG00000247373 | 96.31 | 1.17 | 0.00 | ENSG00000130055 | 113.50 | -2.68 | 0.00 |
| ENSG00000139998 | 3415.74 | 1.17 | 0.00 | ENSG00000152217 | 207.69 | -2.69 | 0.00 |
| ENSG00000173209 | 1013.47 | 1.16 | 0.00 | ENSG00000115590 | 432.20 | -2.69 | 0.00 |
| ENSG00000121621 | 333.37 | 1.16 | 0.00 | ENSG00000173597 | 2131.95 | -2.69 | 0.00 |
| ENSG00000092068 | 2718.94 | 1.16 | 0.00 | ENSG00000132563 | 66.89 | -2.69 | 0.00 |
| ENSG00000177674 | 1439.49 | 1.16 | 0.00 | ENSG00000171951 | 148.95 | -2.69 | 0.00 |
| ENSG00000225828 | 85.49 | 1.16 | 0.00 | ENSG00000167641 | 327.91 | -2.69 | 0.00 |
| ENSG00000169607 | 542.07 | 1.16 | 0.00 | ENSG00000162733 | 988.83 | -2.70 | 0.00 |
| ENSG00000172244 | 147.60 | 1.16 | 0.00 | ENSG00000169860 | 263.20 | -2.70 | 0.00 |
| ENSG00000167525 | 74.13 | 1.16 | 0.00 | ENSG00000135702 | 227.46 | -2.71 | 0.00 |
| ENSG00000064932 | 3767.78 | 1.16 | 0.00 | ENSG00000211652 | 208.13 | -2.71 | 0.00 |
| ENSG00000101457 | 3072.91 | 1.16 | 0.00 | ENSG00000007312 | 117.89 | -2.71 | 0.00 |
| ENSG00000105677 | 3715.10 | 1.16 | 0.00 | ENSG00000111962 | 93.01 | -2.71 | 0.00 |
| ENSG00000258366 | 55.42 | 1.16 | 0.00 | ENSG00000204136 | 95.03 | -2.72 | 0.00 |
| ENSG00000131797 | 562.27 | 1.16 | 0.00 | ENSG00000116741 | 797.91 | -2.72 | 0.00 |
| ENSG00000025708 | 1711.57 | 1.16 | 0.00 | ENSG00000234690 | 53.96 | -2.72 | 0.00 |
| ENSG00000066279 | 943.56 | 1.16 | 0.00 | ENSG00000169347 | 65.24 | -2.72 | 0.01 |
| ENSG00000221955 | 852.29 | 1.16 | 0.00 | ENSG00000082397 | 529.86 | -2.73 | 0.00 |
| ENSG00000281026 | 77.78 | 1.16 | 0.00 | ENSG00000184434 | 911.48 | -2.73 | 0.00 |
| ENSG00000056736 | 1086.14 | 1.16 | 0.00 | ENSG00000124253 | 2911.07 | -2.73 | 0.00 |
| ENSG00000138180 | 1306.06 | 1.16 | 0.00 | ENSG00000137634 | 1396.28 | -2.74 | 0.00 |
| ENSG00000234072 | 250.76 | 1.16 | 0.00 | ENSG00000204614 | 85.70 | -2.74 | 0.00 |
| ENSG00000274627 | 154.25 | 1.16 | 0.00 | ENSG00000253958 | 802.37 | -2.74 | 0.00 |
| ENSG00000136908 | 2140.24 | 1.16 | 0.00 | ENSG00000100626 | 58.52 | -2.75 | 0.00 |
| ENSG00000130726 | 16530.20 | 1.16 | 0.00 | ENSG00000166819 | 74.16 | -2.75 | 0.00 |
| ENSG00000250899 | 196.04 | 1.16 | 0.00 | ENSG00000158560 | 70.96 | -2.75 | 0.00 |
| ENSG00000174276 | 596.98 | 1.16 | 0.00 | ENSG00000172724 | 159.63 | -2.76 | 0.00 |
| ENSG00000271870 | 59.20 | 1.15 | 0.00 | ENSG00000121898 | 480.15 | -2.76 | 0.00 |
| ENSG00000082684 | 61.08 | 1.15 | 0.00 | ENSG00000118308 | 118.78 | -2.76 | 0.00 |
| ENSG00000138031 | 1841.67 | 1.15 | 0.00 | ENSG00000169083 | 67.00 | -2.76 | 0.00 |
| ENSG00000001497 | 2410.71 | 1.15 | 0.00 | ENSG00000137077 | 726.62 | -2.76 | 0.00 |
| ENSG00000276550 | 454.63 | 1.15 | 0.00 | ENSG00000211937 | 77.07 | -2.77 | 0.00 |
| ENSG00000263013 | 50.24 | 1.15 | 0.00 | ENSG00000145687 | 208.63 | -2.77 | 0.00 |
| ENSG00000158402 | 352.48 | 1.15 | 0.00 | ENSG00000232070 | 55.39 | -2.77 | 0.00 |
| ENSG00000105926 | 353.75 | 1.15 | 0.00 | ENSG00000175906 | 118.44 | -2.77 | 0.00 |
| ENSG00000056277 | 220.23 | 1.15 | 0.00 | ENSG00000174514 | 692.48 | -2.77 | 0.00 |
| ENSG00000260196 | 492.29 | 1.15 | 0.00 | ENSG00000184347 | 604.31 | -2.77 | 0.00 |
| ENSG00000152117 | 633.17 | 1.15 | 0.00 | ENSG00000105974 | 1921.24 | -2.78 | 0.00 |
| ENSG00000161888 | 594.20 | 1.15 | 0.00 | ENSG00000165757 | 769.36 | -2.79 | 0.00 |
| ENSG00000146828 | 2881.28 | 1.15 | 0.00 | ENSG00000211611 | 65.64 | -2.79 | 0.00 |
| ENSG00000255224 | 64.11 | 1.15 | 0.00 | ENSG00000095637 | 2605.41 | -2.79 | 0.00 |
| ENSG00000204371 | 3544.97 | 1.15 | 0.00 | ENSG00000152583 | 3356.90 | -2.81 | 0.00 |
| ENSG00000172009 | 2966.41 | 1.15 | 0.00 | ENSG00000151320 | 122.33 | -2.81 | 0.00 |
| ENSG00000101474 | 5574.35 | 1.15 | 0.00 | ENSG00000138735 | 859.54 | -2.82 | 0.00 |
| ENSG00000237649 | 1870.94 | 1.15 | 0.00 | ENSG00000187193 | 689.28 | -2.83 | 0.00 |
| ENSG00000172932 | 1047.29 | 1.15 | 0.00 | ENSG00000160408 | 2531.12 | -2.83 | 0.00 |
| ENSG00000229953 | 216.23 | 1.15 | 0.00 | ENSG00000211639 | 98.88 | -2.83 | 0.00 |
| ENSG00000270605 | 60.52 | 1.15 | 0.00 | ENSG00000197361 | 82.85 | -2.83 | 0.00 |
| ENSG00000065268 | 2222.96 | 1.15 | 0.00 | ENSG00000124749 | 52.31 | -2.83 | 0.00 |
| ENSG00000026508 | 11526.72 | 1.15 | 0.00 | ENSG00000196620 | 170.07 | -2.83 | 0.00 |
| ENSG00000001617 | 1645.46 | 1.15 | 0.00 | ENSG00000242515 | 169.30 | -2.84 | 0.00 |
| ENSG00000148840 | 2468.18 | 1.15 | 0.00 | ENSG00000134533 | 183.75 | -2.84 | 0.00 |
| ENSG00000106125 | 84.24 | 1.14 | 0.00 | ENSG00000125740 | 1325.56 | -2.85 | 0.00 |
| ENSG00000176619 | 6637.28 | 1.14 | 0.00 | ENSG00000134138 | 180.28 | -2.85 | 0.00 |
| ENSG00000181773 | 55.21 | 1.14 | 0.00 | ENSG00000172935 | 501.89 | -2.85 | 0.00 |
| ENSG00000240342 | 2823.90 | 1.14 | 0.00 | ENSG00000185437 | 90.29 | -2.86 | 0.00 |
| ENSG00000132382 | 2996.10 | 1.14 | 0.00 | ENSG00000078295 | 57.74 | -2.86 | 0.00 |
| ENSG00000204172 | 113.10 | 1.14 | 0.00 | ENSG00000164120 | 1271.01 | -2.86 | 0.00 |
| ENSG00000198169 | 679.09 | 1.14 | 0.00 | ENSG00000058668 | 2597.23 | -2.86 | 0.00 |
| ENSG00000102977 | 960.86 | 1.14 | 0.00 | ENSG00000250722 | 4205.08 | -2.86 | 0.00 |
| ENSG00000148399 | 991.06 | 1.14 | 0.00 | ENSG00000198417 | 407.85 | -2.86 | 0.00 |
| ENSG00000132793 | 1454.36 | 1.14 | 0.00 | ENSG00000151623 | 830.99 | -2.86 | 0.00 |
| ENSG00000130881 | 1822.17 | 1.14 | 0.02 | ENSG00000153993 | 61.65 | -2.86 | 0.00 |
| ENSG00000171604 | 3303.60 | 1.14 | 0.00 | ENSG00000156804 | 1838.53 | -2.87 | 0.00 |
| ENSG00000172893 | 4278.20 | 1.14 | 0.00 | ENSG00000187824 | 115.30 | -2.87 | 0.00 |
| ENSG00000280195 | 58.99 | 1.14 | 0.00 | ENSG00000145681 | 121.37 | -2.87 | 0.00 |
| ENSG00000161912 | 125.98 | 1.14 | 0.00 | ENSG00000129167 | 102.09 | -2.87 | 0.00 |
| ENSG00000219438 | 177.29 | 1.14 | 0.01 | ENSG00000115295 | 162.23 | -2.88 | 0.00 |
| ENSG00000117650 | 964.84 | 1.14 | 0.00 | ENSG00000118515 | 1682.06 | -2.88 | 0.00 |
| ENSG00000121957 | 2485.60 | 1.14 | 0.00 | ENSG00000135678 | 684.57 | -2.88 | 0.00 |
| ENSG00000174669 | 1430.95 | 1.14 | 0.00 | ENSG00000211642 | 204.21 | -2.88 | 0.00 |
| ENSG00000183207 | 5494.90 | 1.14 | 0.00 | ENSG00000153956 | 194.01 | -2.88 | 0.00 |
| ENSG00000133216 | 10550.70 | 1.14 | 0.00 | ENSG00000066629 | 435.79 | -2.89 | 0.00 |
| ENSG00000150990 | 1655.30 | 1.13 | 0.00 | ENSG00000132915 | 58.69 | -2.89 | 0.00 |
| ENSG00000106105 | 6121.55 | 1.13 | 0.00 | ENSG00000154556 | 1049.00 | -2.89 | 0.00 |
| ENSG00000130699 | 1602.50 | 1.13 | 0.00 | ENSG00000198947 | 594.43 | -2.90 | 0.00 |
| ENSG00000171984 | 360.16 | 1.13 | 0.00 | ENSG00000154721 | 209.15 | -2.90 | 0.00 |
| ENSG00000134057 | 3012.35 | 1.13 | 0.00 | ENSG00000185483 | 104.72 | -2.90 | 0.00 |
| ENSG00000123179 | 1776.01 | 1.13 | 0.00 | ENSG00000182916 | 63.73 | -2.91 | 0.00 |
| ENSG00000176978 | 3992.45 | 1.13 | 0.00 | ENSG00000198074 | 568.53 | -2.91 | 0.00 |
| ENSG00000103485 | 3626.78 | 1.13 | 0.01 | ENSG00000176533 | 108.39 | -2.92 | 0.00 |
| ENSG00000133247 | 576.40 | 1.13 | 0.00 | ENSG00000086696 | 530.13 | -2.92 | 0.00 |
| ENSG00000138834 | 2012.62 | 1.13 | 0.00 | ENSG00000119919 | 179.69 | -2.92 | 0.00 |
| ENSG00000070814 | 3649.53 | 1.13 | 0.00 | ENSG00000128573 | 233.99 | -2.93 | 0.00 |
| ENSG00000213186 | 361.82 | 1.13 | 0.00 | ENSG00000137094 | 241.16 | -2.94 | 0.00 |
| ENSG00000099899 | 1225.76 | 1.13 | 0.00 | ENSG00000039987 | 165.11 | -2.94 | 0.00 |
| ENSG00000151849 | 633.99 | 1.13 | 0.00 | ENSG00000169715 | 1290.02 | -2.95 | 0.00 |
| ENSG00000162910 | 1431.12 | 1.13 | 0.00 | ENSG00000173175 | 249.93 | -2.95 | 0.00 |
| ENSG00000154358 | 699.38 | 1.13 | 0.02 | ENSG00000119938 | 105.96 | -2.95 | 0.00 |
| ENSG00000132017 | 1575.49 | 1.13 | 0.00 | ENSG00000181856 | 197.92 | -2.95 | 0.00 |
| ENSG00000132004 | 589.56 | 1.13 | 0.00 | ENSG00000146039 | 238.21 | -2.96 | 0.00 |
| ENSG00000274471 | 57.03 | 1.13 | 0.03 | ENSG00000131471 | 1114.40 | -2.96 | 0.00 |
| ENSG00000171858 | 32906.81 | 1.13 | 0.00 | ENSG00000197380 | 219.00 | -2.96 | 0.00 |
| ENSG00000128050 | 7110.48 | 1.13 | 0.00 | ENSG00000127472 | 50.60 | -2.97 | 0.00 |
| ENSG00000244405 | 519.62 | 1.13 | 0.00 | ENSG00000135218 | 329.96 | -2.98 | 0.00 |
| ENSG00000011478 | 729.94 | 1.12 | 0.00 | ENSG00000186642 | 162.09 | -2.98 | 0.00 |
| ENSG00000115268 | 20319.72 | 1.12 | 0.00 | ENSG00000078596 | 287.86 | -2.98 | 0.00 |
| ENSG00000029993 | 2589.81 | 1.12 | 0.00 | ENSG00000178343 | 76.37 | -2.99 | 0.00 |
| ENSG00000236824 | 139.49 | 1.12 | 0.00 | ENSG00000240771 | 306.18 | -3.00 | 0.00 |
| ENSG00000236552 | 1036.12 | 1.12 | 0.01 | ENSG00000166501 | 227.14 | -3.00 | 0.00 |
| ENSG00000164050 | 5288.60 | 1.12 | 0.00 | ENSG00000259823 | 1414.00 | -3.00 | 0.00 |
| ENSG00000125977 | 5529.49 | 1.12 | 0.00 | ENSG00000198467 | 7056.32 | -3.01 | 0.00 |
| ENSG00000153291 | 200.83 | 1.12 | 0.00 | ENSG00000180155 | 136.96 | -3.01 | 0.00 |
| ENSG00000120889 | 2813.46 | 1.12 | 0.00 | ENSG00000145384 | 369.34 | -3.01 | 0.00 |
| ENSG00000126768 | 2802.71 | 1.12 | 0.00 | ENSG00000146469 | 369.63 | -3.01 | 0.00 |
| ENSG00000162065 | 570.50 | 1.12 | 0.00 | ENSG00000156234 | 184.84 | -3.02 | 0.00 |
| ENSG00000242802 | 2178.18 | 1.12 | 0.00 | ENSG00000271447 | 408.04 | -3.02 | 0.00 |
| ENSG00000167526 | 71897.11 | 1.12 | 0.00 | ENSG00000280143 | 213.66 | -3.02 | 0.00 |
| ENSG00000181135 | 489.09 | 1.12 | 0.00 | ENSG00000179674 | 373.90 | -3.02 | 0.00 |
| ENSG00000130675 | 485.55 | 1.12 | 0.00 | ENSG00000101680 | 185.67 | -3.03 | 0.00 |
| ENSG00000089685 | 2677.39 | 1.12 | 0.00 | ENSG00000019102 | 1303.07 | -3.03 | 0.00 |
| ENSG00000065923 | 408.01 | 1.12 | 0.01 | ENSG00000174944 | 98.40 | -3.04 | 0.00 |
| ENSG00000106344 | 1403.76 | 1.12 | 0.00 | ENSG00000132514 | 127.24 | -3.05 | 0.00 |
| ENSG00000164220 | 261.86 | 1.12 | 0.01 | ENSG00000007306 | 9620.57 | -3.06 | 0.00 |
| ENSG00000054179 | 1333.67 | 1.12 | 0.00 | ENSG00000162461 | 62.31 | -3.06 | 0.00 |
| ENSG00000138030 | 698.57 | 1.12 | 0.00 | ENSG00000065320 | 242.50 | -3.06 | 0.00 |
| ENSG00000108262 | 4031.47 | 1.12 | 0.00 | ENSG00000135220 | 692.28 | -3.07 | 0.00 |
| ENSG00000138363 | 5357.45 | 1.11 | 0.00 | ENSG00000117115 | 2657.17 | -3.08 | 0.00 |
| ENSG00000143498 | 217.74 | 1.11 | 0.00 | ENSG00000186868 | 78.03 | -3.08 | 0.00 |
| ENSG00000177732 | 959.71 | 1.11 | 0.00 | ENSG00000112964 | 159.01 | -3.08 | 0.00 |
| ENSG00000242861 | 213.19 | 1.11 | 0.00 | ENSG00000170323 | 125.98 | -3.08 | 0.00 |
| ENSG00000156521 | 1570.80 | 1.11 | 0.00 | ENSG00000101955 | 234.66 | -3.10 | 0.00 |
| ENSG00000165644 | 1534.33 | 1.11 | 0.03 | ENSG00000160307 | 121.50 | -3.10 | 0.00 |
| ENSG00000053918 | 5791.47 | 1.11 | 0.00 | ENSG00000137726 | 529.47 | -3.10 | 0.00 |
| ENSG00000164611 | 1506.52 | 1.11 | 0.00 | ENSG00000148483 | 486.27 | -3.11 | 0.00 |
| ENSG00000147536 | 351.80 | 1.11 | 0.00 | ENSG00000166825 | 2169.57 | -3.11 | 0.00 |
| ENSG00000061938 | 2152.63 | 1.11 | 0.00 | ENSG00000130224 | 60.68 | -3.12 | 0.00 |
| ENSG00000242372 | 10676.40 | 1.11 | 0.00 | ENSG00000127083 | 54.10 | -3.13 | 0.00 |
| ENSG00000172824 | 106.05 | 1.11 | 0.01 | ENSG00000138615 | 230.24 | -3.13 | 0.00 |
| ENSG00000177380 | 962.65 | 1.11 | 0.00 | ENSG00000095303 | 931.20 | -3.14 | 0.00 |
| ENSG00000101161 | 8183.38 | 1.11 | 0.00 | ENSG00000174099 | 931.92 | -3.15 | 0.00 |
| ENSG00000123473 | 819.31 | 1.11 | 0.00 | ENSG00000061455 | 81.93 | -3.17 | 0.00 |
| ENSG00000173272 | 1813.70 | 1.11 | 0.00 | ENSG00000163687 | 189.26 | -3.17 | 0.00 |
| ENSG00000181026 | 1656.88 | 1.11 | 0.00 | ENSG00000154165 | 50.02 | -3.17 | 0.00 |
| ENSG00000197927 | 84.74 | 1.11 | 0.00 | ENSG00000106034 | 441.85 | -3.18 | 0.00 |
| ENSG00000165886 | 337.69 | 1.11 | 0.00 | ENSG00000197165 | 79.57 | -3.19 | 0.00 |
| ENSG00000170485 | 2242.07 | 1.11 | 0.00 | ENSG00000172915 | 125.89 | -3.19 | 0.00 |
| ENSG00000165138 | 1706.54 | 1.11 | 0.00 | ENSG00000100079 | 441.74 | -3.19 | 0.00 |
| ENSG00000124222 | 3004.89 | 1.11 | 0.00 | ENSG00000100628 | 236.77 | -3.20 | 0.00 |
| ENSG00000007384 | 2031.09 | 1.10 | 0.00 | ENSG00000112183 | 71.85 | -3.20 | 0.00 |
| ENSG00000140859 | 685.22 | 1.10 | 0.00 | ENSG00000143603 | 157.84 | -3.21 | 0.00 |
| ENSG00000222009 | 107.80 | 1.10 | 0.00 | ENSG00000105894 | 145.81 | -3.23 | 0.00 |
| ENSG00000144837 | 169.08 | 1.10 | 0.02 | ENSG00000156966 | 3385.95 | -3.23 | 0.00 |
| ENSG00000123975 | 2056.47 | 1.10 | 0.00 | ENSG00000176641 | 264.36 | -3.23 | 0.00 |
| ENSG00000101464 | 2268.42 | 1.10 | 0.00 | ENSG00000189056 | 66.32 | -3.24 | 0.00 |
| ENSG00000119681 | 1878.38 | 1.10 | 0.01 | ENSG00000149591 | 11915.73 | -3.25 | 0.00 |
| ENSG00000168140 | 529.15 | 1.10 | 0.01 | ENSG00000112186 | 220.59 | -3.26 | 0.00 |
| ENSG00000164054 | 8907.36 | 1.10 | 0.00 | ENSG00000107562 | 1072.34 | -3.26 | 0.00 |
| ENSG00000144115 | 777.06 | 1.10 | 0.04 | ENSG00000186198 | 272.96 | -3.27 | 0.00 |
| ENSG00000165655 | 1112.99 | 1.10 | 0.00 | ENSG00000108924 | 105.75 | -3.27 | 0.00 |
| ENSG00000152229 | 473.07 | 1.10 | 0.00 | ENSG00000113296 | 627.23 | -3.29 | 0.00 |
| ENSG00000247077 | 3542.51 | 1.10 | 0.00 | ENSG00000079308 | 4771.46 | -3.29 | 0.00 |
| ENSG00000118420 | 222.70 | 1.10 | 0.00 | ENSG00000012124 | 111.91 | -3.29 | 0.00 |
| ENSG00000160959 | 1446.25 | 1.10 | 0.00 | ENSG00000073737 | 636.77 | -3.30 | 0.00 |
| ENSG00000159685 | 631.82 | 1.10 | 0.00 | ENSG00000229619 | 153.65 | -3.30 | 0.00 |
| ENSG00000146918 | 1473.32 | 1.10 | 0.00 | ENSG00000197614 | 451.04 | -3.33 | 0.00 |
| ENSG00000170801 | 1761.45 | 1.10 | 0.02 | ENSG00000133083 | 83.70 | -3.34 | 0.00 |
| ENSG00000090889 | 1266.13 | 1.10 | 0.00 | ENSG00000127951 | 849.14 | -3.35 | 0.00 |
| ENSG00000102312 | 394.94 | 1.10 | 0.00 | ENSG00000172318 | 85.94 | -3.35 | 0.00 |
| ENSG00000141696 | 1236.79 | 1.10 | 0.00 | ENSG00000118407 | 123.13 | -3.35 | 0.00 |
| ENSG00000135446 | 3940.87 | 1.10 | 0.00 | ENSG00000048462 | 67.29 | -3.35 | 0.00 |
| ENSG00000167775 | 3884.13 | 1.10 | 0.00 | ENSG00000171714 | 103.12 | -3.36 | 0.00 |
| ENSG00000126215 | 759.28 | 1.10 | 0.00 | ENSG00000166869 | 1432.17 | -3.36 | 0.00 |
| ENSG00000160602 | 275.81 | 1.09 | 0.00 | ENSG00000134201 | 63.19 | -3.36 | 0.00 |
| ENSG00000127957 | 95.77 | 1.09 | 0.00 | ENSG00000149294 | 217.12 | -3.36 | 0.00 |
| ENSG00000102393 | 1261.41 | 1.09 | 0.00 | ENSG00000134121 | 125.01 | -3.37 | 0.00 |
| ENSG00000146410 | 300.93 | 1.09 | 0.00 | ENSG00000072195 | 360.60 | -3.38 | 0.00 |
| ENSG00000196236 | 774.42 | 1.09 | 0.00 | ENSG00000105737 | 55.70 | -3.39 | 0.00 |
| ENSG00000103811 | 5498.03 | 1.09 | 0.00 | ENSG00000163145 | 59.49 | -3.39 | 0.00 |
| ENSG00000138346 | 408.59 | 1.09 | 0.00 | ENSG00000109182 | 196.88 | -3.40 | 0.00 |
| ENSG00000127191 | 1307.96 | 1.09 | 0.00 | ENSG00000158246 | 66.04 | -3.40 | 0.00 |
| ENSG00000064547 | 923.99 | 1.09 | 0.00 | ENSG00000203685 | 120.80 | -3.41 | 0.00 |
| ENSG00000080189 | 3578.46 | 1.09 | 0.00 | ENSG00000154262 | 52.02 | -3.42 | 0.00 |
| ENSG00000122547 | 1309.57 | 1.09 | 0.00 | ENSG00000018236 | 82.56 | -3.42 | 0.00 |
| ENSG00000120075 | 680.16 | 1.09 | 0.01 | ENSG00000149596 | 311.74 | -3.43 | 0.00 |
| ENSG00000103222 | 3251.96 | 1.09 | 0.00 | ENSG00000248144 | 1200.37 | -3.44 | 0.00 |
| ENSG00000099821 | 2761.14 | 1.09 | 0.00 | ENSG00000111846 | 80.28 | -3.44 | 0.00 |
| ENSG00000132768 | 1839.84 | 1.09 | 0.00 | ENSG00000125144 | 1520.55 | -3.45 | 0.00 |
| ENSG00000138411 | 195.09 | 1.09 | 0.00 | ENSG00000112276 | 103.48 | -3.45 | 0.00 |
| ENSG00000226328 | 239.61 | 1.09 | 0.00 | ENSG00000168447 | 775.97 | -3.46 | 0.00 |
| ENSG00000140525 | 1836.48 | 1.09 | 0.00 | ENSG00000155850 | 7038.86 | -3.47 | 0.00 |
| ENSG00000118705 | 21120.46 | 1.09 | 0.00 | ENSG00000158887 | 65.82 | -3.47 | 0.00 |
| ENSG00000183751 | 2561.71 | 1.09 | 0.00 | ENSG00000077157 | 3021.37 | -3.47 | 0.00 |
| ENSG00000169718 | 5971.30 | 1.09 | 0.00 | ENSG00000067840 | 75.74 | -3.49 | 0.00 |
| ENSG00000204237 | 909.15 | 1.09 | 0.00 | ENSG00000004799 | 927.49 | -3.51 | 0.00 |
| ENSG00000125798 | 2036.29 | 1.09 | 0.00 | ENSG00000165410 | 601.21 | -3.52 | 0.00 |
| ENSG00000172478 | 555.39 | 1.09 | 0.03 | ENSG00000130294 | 58.66 | -3.53 | 0.00 |
| ENSG00000178460 | 87.10 | 1.09 | 0.01 | ENSG00000186417 | 147.22 | -3.53 | 0.00 |
| ENSG00000180900 | 6238.66 | 1.09 | 0.00 | ENSG00000168309 | 139.48 | -3.54 | 0.00 |
| ENSG00000166451 | 644.05 | 1.09 | 0.00 | ENSG00000004776 | 576.28 | -3.54 | 0.00 |
| ENSG00000182173 | 1986.37 | 1.09 | 0.00 | ENSG00000167434 | 1433.76 | -3.54 | 0.00 |
| ENSG00000147162 | 6126.12 | 1.09 | 0.00 | ENSG00000198300 | 61.87 | -3.56 | 0.00 |
| ENSG00000106003 | 4646.37 | 1.08 | 0.00 | ENSG00000198624 | 944.00 | -3.58 | 0.00 |
| ENSG00000184402 | 944.31 | 1.08 | 0.00 | ENSG00000163295 | 160.29 | -3.58 | 0.00 |
| ENSG00000260428 | 99.81 | 1.08 | 0.02 | ENSG00000249669 | 71.11 | -3.60 | 0.00 |
| ENSG00000178026 | 444.38 | 1.08 | 0.00 | ENSG00000141052 | 269.10 | -3.62 | 0.00 |
| ENSG00000060558 | 388.44 | 1.08 | 0.00 | ENSG00000128591 | 2709.71 | -3.62 | 0.00 |
| ENSG00000148384 | 665.96 | 1.08 | 0.00 | ENSG00000101335 | 11794.02 | -3.62 | 0.00 |
| ENSG00000213442 | 768.84 | 1.08 | 0.02 | ENSG00000145936 | 361.68 | -3.62 | 0.00 |
| ENSG00000132801 | 382.19 | 1.08 | 0.00 | ENSG00000119121 | 501.35 | -3.62 | 0.00 |
| ENSG00000260114 | 119.84 | 1.08 | 0.00 | ENSG00000185774 | 63.75 | -3.64 | 0.00 |
| ENSG00000049089 | 509.45 | 1.08 | 0.05 | ENSG00000166831 | 161.26 | -3.64 | 0.00 |
| ENSG00000166166 | 1708.70 | 1.08 | 0.00 | ENSG00000173237 | 160.82 | -3.65 | 0.00 |
| ENSG00000077152 | 944.64 | 1.08 | 0.00 | ENSG00000204936 | 1685.31 | -3.67 | 0.00 |
| ENSG00000171097 | 189.24 | 1.08 | 0.00 | ENSG00000130822 | 114.65 | -3.67 | 0.00 |
| ENSG00000149016 | 508.67 | 1.08 | 0.00 | ENSG00000136842 | 83.00 | -3.67 | 0.00 |
| ENSG00000080608 | 1856.36 | 1.08 | 0.00 | ENSG00000174358 | 150.42 | -3.69 | 0.00 |
| ENSG00000180340 | 149.78 | 1.08 | 0.01 | ENSG00000187699 | 465.04 | -3.70 | 0.00 |
| ENSG00000087077 | 2485.45 | 1.08 | 0.01 | ENSG00000046653 | 177.39 | -3.71 | 0.00 |
| ENSG00000133265 | 2907.26 | 1.08 | 0.00 | ENSG00000156738 | 113.56 | -3.71 | 0.00 |
| ENSG00000134905 | 3052.89 | 1.08 | 0.00 | ENSG00000151892 | 160.31 | -3.74 | 0.00 |
| ENSG00000136122 | 492.01 | 1.08 | 0.00 | ENSG00000180875 | 271.51 | -3.75 | 0.00 |
| ENSG00000124787 | 329.66 | 1.08 | 0.00 | ENSG00000113594 | 164.58 | -3.75 | 0.00 |
| ENSG00000164867 | 578.89 | 1.08 | 0.00 | ENSG00000172260 | 237.95 | -3.76 | 0.00 |
| ENSG00000224877 | 943.23 | 1.08 | 0.00 | ENSG00000197888 | 1639.31 | -3.77 | 0.00 |
| ENSG00000115687 | 650.70 | 1.08 | 0.00 | ENSG00000205358 | 130.48 | -3.77 | 0.00 |
| ENSG00000197157 | 13116.49 | 1.08 | 0.00 | ENSG00000134028 | 1348.26 | -3.78 | 0.00 |
| ENSG00000105821 | 1527.65 | 1.08 | 0.00 | ENSG00000101938 | 489.65 | -3.78 | 0.00 |
| ENSG00000096384 | 56060.01 | 1.08 | 0.00 | ENSG00000187783 | 101.47 | -3.79 | 0.00 |
| ENSG00000148426 | 454.41 | 1.08 | 0.00 | ENSG00000101605 | 117.31 | -3.82 | 0.00 |
| ENSG00000111788 | 130.11 | 1.08 | 0.00 | ENSG00000168497 | 401.45 | -3.83 | 0.00 |
| ENSG00000148334 | 4214.80 | 1.08 | 0.00 | ENSG00000144218 | 61.88 | -3.84 | 0.00 |
| ENSG00000137692 | 981.28 | 1.08 | 0.00 | ENSG00000138944 | 132.86 | -3.85 | 0.00 |
| ENSG00000239857 | 270.62 | 1.08 | 0.00 | ENSG00000156113 | 786.05 | -3.85 | 0.00 |
| ENSG00000179271 | 2736.23 | 1.07 | 0.00 | ENSG00000175084 | 12926.93 | -3.85 | 0.00 |
| ENSG00000141569 | 1299.46 | 1.07 | 0.00 | ENSG00000022267 | 1760.06 | -3.89 | 0.00 |
| ENSG00000275004 | 108.82 | 1.07 | 0.00 | ENSG00000154175 | 350.11 | -3.89 | 0.00 |
| ENSG00000197140 | 97.88 | 1.07 | 0.03 | ENSG00000145362 | 248.50 | -3.92 | 0.00 |
| ENSG00000204386 | 4771.53 | 1.07 | 0.00 | ENSG00000163710 | 77.67 | -3.93 | 0.00 |
| ENSG00000204348 | 581.33 | 1.07 | 0.00 | ENSG00000174992 | 6151.88 | -3.94 | 0.00 |
| ENSG00000168096 | 553.43 | 1.07 | 0.00 | ENSG00000135447 | 59.21 | -3.94 | 0.00 |
| ENSG00000147383 | 1823.09 | 1.07 | 0.00 | ENSG00000169432 | 91.19 | -3.94 | 0.00 |
| ENSG00000149091 | 3907.32 | 1.07 | 0.00 | ENSG00000181617 | 69.18 | -3.94 | 0.00 |
| ENSG00000245910 | 2557.02 | 1.07 | 0.00 | ENSG00000065534 | 6533.43 | -3.95 | 0.00 |
| ENSG00000147144 | 1052.19 | 1.07 | 0.00 | ENSG00000156218 | 149.55 | -3.96 | 0.00 |
| ENSG00000105855 | 800.66 | 1.07 | 0.01 | ENSG00000117322 | 181.78 | -3.96 | 0.00 |
| ENSG00000165591 | 379.28 | 1.07 | 0.00 | ENSG00000166828 | 68.87 | -3.98 | 0.00 |
| ENSG00000121152 | 915.11 | 1.07 | 0.00 | ENSG00000118777 | 221.36 | -3.98 | 0.00 |
| ENSG00000279978 | 294.82 | 1.07 | 0.00 | ENSG00000138722 | 175.41 | -4.01 | 0.00 |
| ENSG00000113140 | 33881.09 | 1.07 | 0.01 | ENSG00000132465 | 21759.21 | -4.02 | 0.00 |
| ENSG00000168672 | 2648.27 | 1.07 | 0.00 | ENSG00000135549 | 457.95 | -4.02 | 0.00 |
| ENSG00000184445 | 1015.97 | 1.07 | 0.00 | ENSG00000091138 | 11804.74 | -4.03 | 0.00 |
| ENSG00000264112 | 401.27 | 1.07 | 0.00 | ENSG00000059915 | 378.02 | -4.03 | 0.00 |
| ENSG00000186810 | 253.19 | 1.07 | 0.01 | ENSG00000144891 | 51.46 | -4.03 | 0.00 |
| ENSG00000103037 | 565.47 | 1.07 | 0.00 | ENSG00000184809 | 103.50 | -4.04 | 0.00 |
| ENSG00000164045 | 795.26 | 1.07 | 0.00 | ENSG00000127241 | 250.51 | -4.04 | 0.00 |
| ENSG00000280987 | 79.46 | 1.07 | 0.00 | ENSG00000135333 | 138.31 | -4.05 | 0.00 |
| ENSG00000261578 | 58.90 | 1.07 | 0.00 | ENSG00000130176 | 3511.33 | -4.07 | 0.00 |
| ENSG00000167615 | 5837.36 | 1.07 | 0.00 | ENSG00000163431 | 1795.61 | -4.11 | 0.00 |
| ENSG00000246575 | 89.70 | 1.07 | 0.00 | ENSG00000163017 | 9293.00 | -4.11 | 0.00 |
| ENSG00000146263 | 559.34 | 1.07 | 0.00 | ENSG00000164530 | 111.14 | -4.15 | 0.00 |
| ENSG00000110911 | 3440.88 | 1.07 | 0.00 | ENSG00000065325 | 66.54 | -4.16 | 0.00 |
| ENSG00000157399 | 2994.57 | 1.07 | 0.02 | ENSG00000087258 | 221.83 | -4.16 | 0.00 |
| ENSG00000185090 | 725.47 | 1.07 | 0.00 | ENSG00000135842 | 902.90 | -4.18 | 0.00 |
| ENSG00000185238 | 978.82 | 1.07 | 0.00 | ENSG00000167676 | 388.28 | -4.19 | 0.00 |
| ENSG00000133027 | 812.55 | 1.06 | 0.00 | ENSG00000181541 | 505.44 | -4.19 | 0.00 |
| ENSG00000279382 | 88.36 | 1.06 | 0.00 | ENSG00000121577 | 162.95 | -4.23 | 0.00 |
| ENSG00000239382 | 101.24 | 1.06 | 0.00 | ENSG00000104332 | 275.35 | -4.24 | 0.00 |
| ENSG00000158050 | 565.00 | 1.06 | 0.01 | ENSG00000170153 | 190.61 | -4.27 | 0.00 |
| ENSG00000183048 | 1421.73 | 1.06 | 0.00 | ENSG00000100604 | 1155.01 | -4.28 | 0.00 |
| ENSG00000276710 | 108.26 | 1.06 | 0.00 | ENSG00000168477 | 472.74 | -4.32 | 0.00 |
| ENSG00000142528 | 505.57 | 1.06 | 0.00 | ENSG00000152137 | 655.91 | -4.35 | 0.00 |
| ENSG00000144559 | 468.59 | 1.06 | 0.00 | ENSG00000118729 | 178.91 | -4.37 | 0.00 |
| ENSG00000215154 | 82.81 | 1.06 | 0.00 | ENSG00000163815 | 205.44 | -4.38 | 0.00 |
| ENSG00000132952 | 1132.74 | 1.06 | 0.00 | ENSG00000154330 | 810.26 | -4.40 | 0.00 |
| ENSG00000184786 | 87.56 | 1.06 | 0.00 | ENSG00000205364 | 205.70 | -4.44 | 0.00 |
| ENSG00000273117 | 65.29 | 1.06 | 0.00 | ENSG00000068976 | 75.64 | -4.45 | 0.00 |
| ENSG00000136111 | 2290.65 | 1.06 | 0.00 | ENSG00000197273 | 1729.01 | -4.46 | 0.00 |
| ENSG00000265415 | 86.93 | 1.06 | 0.00 | ENSG00000198523 | 489.55 | -4.47 | 0.00 |
| ENSG00000221829 | 954.01 | 1.06 | 0.00 | ENSG00000133800 | 223.10 | -4.47 | 0.00 |
| ENSG00000198026 | 1283.26 | 1.06 | 0.00 | ENSG00000168079 | 342.30 | -4.49 | 0.00 |
| ENSG00000177943 | 425.37 | 1.06 | 0.03 | ENSG00000075073 | 248.41 | -4.50 | 0.00 |
| ENSG00000137807 | 954.90 | 1.06 | 0.00 | ENSG00000122367 | 118.14 | -4.54 | 0.00 |
| ENSG00000103168 | 1321.08 | 1.06 | 0.00 | ENSG00000163873 | 51.18 | -4.55 | 0.00 |
| ENSG00000064102 | 1150.74 | 1.06 | 0.00 | ENSG00000172403 | 3246.63 | -4.59 | 0.00 |
| ENSG00000120526 | 1210.00 | 1.06 | 0.00 | ENSG00000126950 | 116.22 | -4.61 | 0.00 |
| ENSG00000111364 | 885.29 | 1.06 | 0.00 | ENSG00000181072 | 96.39 | -4.62 | 0.00 |
| ENSG00000142675 | 1384.03 | 1.06 | 0.00 | ENSG00000164107 | 147.28 | -4.64 | 0.00 |
| ENSG00000128626 | 2533.94 | 1.06 | 0.00 | ENSG00000183287 | 72.72 | -4.71 | 0.00 |
| ENSG00000240230 | 897.83 | 1.06 | 0.00 | ENSG00000156284 | 425.35 | -4.72 | 0.00 |
| ENSG00000261804 | 53.07 | 1.06 | 0.00 | ENSG00000113196 | 75.50 | -4.74 | 0.00 |
| ENSG00000116771 | 1565.16 | 1.06 | 0.00 | ENSG00000112936 | 602.04 | -4.76 | 0.00 |
| ENSG00000120158 | 602.45 | 1.05 | 0.00 | ENSG00000080493 | 1024.45 | -4.78 | 0.00 |
| ENSG00000121211 | 225.99 | 1.05 | 0.00 | ENSG00000240498 | 209.40 | -4.79 | 0.00 |
| ENSG00000185122 | 5194.00 | 1.05 | 0.00 | ENSG00000104267 | 3811.94 | -4.82 | 0.00 |
| ENSG00000231607 | 152.81 | 1.05 | 0.01 | ENSG00000141338 | 176.32 | -4.83 | 0.00 |
| ENSG00000255737 | 229.47 | 1.05 | 0.00 | ENSG00000048540 | 204.46 | -4.85 | 0.00 |
| ENSG00000198959 | 7012.37 | 1.05 | 0.00 | ENSG00000170500 | 53.00 | -4.87 | 0.00 |
| ENSG00000214021 | 572.49 | 1.05 | 0.00 | ENSG00000106809 | 249.37 | -4.88 | 0.00 |
| ENSG00000197362 | 317.10 | 1.05 | 0.00 | ENSG00000044012 | 197.72 | -4.88 | 0.00 |
| ENSG00000167967 | 1304.02 | 1.05 | 0.00 | ENSG00000182253 | 2308.87 | -4.88 | 0.00 |
| ENSG00000171224 | 262.34 | 1.05 | 0.00 | ENSG00000143196 | 635.19 | -4.88 | 0.00 |
| ENSG00000146731 | 10073.59 | 1.05 | 0.00 | ENSG00000016602 | 2315.79 | -4.90 | 0.00 |
| ENSG00000183077 | 1068.95 | 1.05 | 0.00 | ENSG00000079689 | 63.37 | -4.93 | 0.00 |
| ENSG00000256525 | 322.24 | 1.05 | 0.00 | ENSG00000152785 | 177.15 | -4.94 | 0.00 |
| ENSG00000168411 | 2063.27 | 1.05 | 0.00 | ENSG00000269404 | 158.54 | -4.97 | 0.00 |
| ENSG00000170191 | 455.19 | 1.05 | 0.00 | ENSG00000133392 | 26342.47 | -4.97 | 0.00 |
| ENSG00000048162 | 1332.79 | 1.05 | 0.00 | ENSG00000167281 | 60.20 | -4.98 | 0.00 |
| ENSG00000157778 | 1544.70 | 1.05 | 0.00 | ENSG00000071203 | 1310.80 | -5.05 | 0.00 |
| ENSG00000153044 | 524.53 | 1.05 | 0.00 | ENSG00000165966 | 83.78 | -5.16 | 0.00 |
| ENSG00000013306 | 9487.45 | 1.05 | 0.00 | ENSG00000162706 | 92.90 | -5.17 | 0.00 |
| ENSG00000143793 | 766.92 | 1.05 | 0.00 | ENSG00000165072 | 120.42 | -5.24 | 0.00 |
| ENSG00000171425 | 986.06 | 1.05 | 0.00 | ENSG00000196616 | 599.21 | -5.34 | 0.00 |
| ENSG00000279413 | 52.99 | 1.05 | 0.00 | ENSG00000114200 | 98.90 | -5.35 | 0.00 |
| ENSG00000106268 | 904.60 | 1.05 | 0.00 | ENSG00000142959 | 193.07 | -5.43 | 0.00 |
| ENSG00000160917 | 1291.43 | 1.05 | 0.00 | ENSG00000131096 | 394.96 | -5.48 | 0.00 |
| ENSG00000215045 | 56.10 | 1.04 | 0.00 | ENSG00000168748 | 275.21 | -5.48 | 0.00 |
| ENSG00000182648 | 479.96 | 1.04 | 0.00 | ENSG00000116194 | 127.57 | -5.50 | 0.00 |
| ENSG00000153574 | 2313.87 | 1.04 | 0.00 | ENSG00000175785 | 77.49 | -5.51 | 0.00 |
| ENSG00000000460 | 404.68 | 1.04 | 0.00 | ENSG00000103375 | 1065.23 | -5.51 | 0.00 |
| ENSG00000149679 | 794.55 | 1.04 | 0.00 | ENSG00000182271 | 326.28 | -5.57 | 0.00 |
| ENSG00000101000 | 1504.71 | 1.04 | 0.01 | ENSG00000147655 | 74.32 | -5.61 | 0.00 |
| ENSG00000171456 | 3400.68 | 1.04 | 0.00 | ENSG00000150625 | 92.93 | -5.62 | 0.00 |
| ENSG00000253729 | 9822.15 | 1.04 | 0.00 | ENSG00000018625 | 194.99 | -5.81 | 0.00 |
| ENSG00000141349 | 2791.28 | 1.04 | 0.00 | ENSG00000172410 | 386.78 | -5.90 | 0.00 |
| ENSG00000112742 | 769.36 | 1.04 | 0.00 | ENSG00000171246 | 155.09 | -6.02 | 0.00 |
| ENSG00000177595 | 811.55 | 1.04 | 0.00 | ENSG00000130226 | 60.10 | -6.05 | 0.00 |
| ENSG00000188747 | 765.00 | 1.04 | 0.01 | ENSG00000237125 | 128.04 | -6.06 | 0.00 |
| ENSG00000134461 | 366.00 | 1.04 | 0.00 | ENSG00000133742 | 3940.60 | -6.13 | 0.00 |
| ENSG00000021300 | 1821.11 | 1.04 | 0.01 | ENSG00000115263 | 315.20 | -6.21 | 0.00 |
| ENSG00000170919 | 434.05 | 1.04 | 0.00 | ENSG00000157005 | 95.82 | -6.32 | 0.00 |
| ENSG00000174943 | 712.70 | 1.04 | 0.00 | ENSG00000136546 | 172.37 | -6.34 | 0.00 |
| ENSG00000169679 | 1329.16 | 1.04 | 0.00 | ENSG00000123560 | 132.45 | -6.52 | 0.00 |
| ENSG00000100401 | 5417.88 | 1.04 | 0.00 | ENSG00000183034 | 141.85 | -7.52 | 0.00 |

**Supplementary table 5 GO analysis of overlap DEGs for COAD**

| Term | | Count | PValue |
| --- | --- | --- | --- |
| CC | integral component of plasma membrane | 26 | 0.015 |
|  | cell surface | 12 | 0.041 |
|  | dendrite | 10 | 0.013 |
|  | synapse | 7 | 0.016 |
|  | axon | 7 | 0.038 |
|  | cell-cell junction | 6 | 0.044 |
|  | Z disc | 5 | 0.043 |
|  | extrinsic component of cytoplasmic side of plasma membrane | 4 | 0.040 |
|  | connexon complex | 3 | 0.023 |
|  | filamentous actin | 3 | 0.046 |
|  | main axon | 2 | 0.033 |
| BP | negative regulation of transcription from RNA polymerase II promoter | 17 | 0.012 |
|  | protein phosphorylation | 11 | 0.045 |
|  | cell adhesion | 11 | 0.047 |
|  | axon guidance | 7 | 0.012 |
|  | skeletal system development | 6 | 0.024 |
|  | positive regulation of MAPK cascade | 5 | 0.016 |
|  | response to toxic substance | 5 | 0.018 |
|  | cell-matrix adhesion | 5 | 0.022 |
|  | osteoblast differentiation | 5 | 0.035 |
|  | neural crest cell migration | 4 | 0.016 |
|  | organ regeneration | 4 | 0.018 |
|  | positive regulation of fibroblast proliferation | 4 | 0.026 |
|  | cartilage development | 4 | 0.033 |
|  | SMAD protein signal transduction | 4 | 0.037 |
|  | positive regulation of phosphatidylinositol 3-kinase signaling | 4 | 0.042 |
|  | endocardial cushion morphogenesis | 3 | 0.015 |
|  | negative regulation of axon extension involved in axon guidance | 3 | 0.038 |
|  | positive regulation of filopodium assembly | 3 | 0.038 |
|  | endocrine pancreas development | 3 | 0.038 |
|  | growth | 3 | 0.043 |
|  | long-term memory | 3 | 0.043 |
|  | dopaminergic neuron axon guidance | 2 | 0.035 |
|  | serotonergic neuron axon guidance | 2 | 0.035 |
| MF | protein kinase activity | 10 | 0.021 |
|  | growth factor activity | 6 | 0.037 |
|  | semaphorin receptor binding | 3 | 0.028 |
|  | chemorepellent activity | 3 | 0.037 |

**Supplementary table 6 GO analysis of overlap DEGs for READ**

| Term | | Count | PValue |
| --- | --- | --- | --- |
| CC | nucleus | 81 | 0.011 |
|  | nucleoplasm | 44 | 0.036 |
|  | cytoskeleton | 11 | 0.013 |
|  | microtubule | 10 | 0.012 |
|  | focal adhesion | 10 | 0.043 |
|  | actin cytoskeleton | 8 | 0.015 |
|  | growth cone | 6 | 0.012 |
|  | lamellipodium | 6 | 0.041 |
| BP | signal transduction | 25 | 0.013 |
|  | apoptotic process | 15 | 0.014 |
|  | positive regulation of transcription, DNA-templated | 14 | 0.015 |
|  | peptidyl-tyrosine phosphorylation | 7 | 0.014 |
|  | axon guidance | 7 | 0.016 |
|  | transcription initiation from RNA polymerase II promoter | 6 | 0.045 |
|  | ephrin receptor signaling pathway | 5 | 0.024 |
|  | neuron migration | 5 | 0.045 |
|  | intracellular receptor signaling pathway | 4 | 0.012 |
|  | cell development | 4 | 0.014 |
|  | negative regulation of fat cell differentiation | 4 | 0.016 |
|  | circadian regulation of gene expression | 4 | 0.036 |
|  | retinal ganglion cell axon guidance | 3 | 0.024 |
|  | positive regulation of cardiac muscle cell proliferation | 3 | 0.032 |
|  | protein localization to cell surface | 3 | 0.032 |
|  | regulation of ERK1 and ERK2 cascade | 3 | 0.040 |
|  | growth | 3 | 0.049 |
|  | negative regulation of cytoplasmic translational elongation | 2 | 0.025 |
|  | negative regulation of myofibroblast differentiation | 2 | 0.025 |
| MF | metal ion binding | 37 | 0.028 |
|  | zinc ion binding | 24 | 0.023 |
|  | protein kinase activity | 11 | 0.016 |
|  | protein serine/threonine kinase activity | 11 | 0.021 |
|  | protein kinase binding | 11 | 0.021 |
|  | microtubule binding | 7 | 0.049 |
|  | beta-catenin binding | 5 | 0.020 |
|  | core promoter sequence-specific DNA binding | 4 | 0.017 |
|  | mRNA 3'-UTR binding | 4 | 0.024 |
|  | GDP binding | 4 | 0.031 |
|  | transcriptional repressor activity, RNA polymerase II transcription regulatory region sequence-specific binding | 4 | 0.038 |
|  | transmembrane receptor protein tyrosine phosphatase activity | 3 | 0.019 |
|  | chemorepellent activity | 3 | 0.045 |
|  | activin receptor activity, type I | 2 | 0.050 |
|  | retinoic acid 4-hydroxylase activity | 2 | 0.050 |
|  | ciliary neurotrophic factor receptor activity | 2 | 0.050 |

**Supplementary table 7 KEGG analysis of overlap DEGs for COAD**

| Term | Count | PValue |
| --- | --- | --- |
| Hippo signaling pathway | 7 | 0.010 |
| Alzheimer's disease | 7 | 0.016 |
| Rap1 signaling pathway | 7 | 0.043 |
| Melanoma | 5 | 0.011 |
| Gap junction | 5 | 0.022 |
| Prostate cancer | 5 | 0.022 |
| Circadian entrainment | 5 | 0.028 |
| Choline metabolism in cancer | 5 | 0.035 |
| Chagas disease (American trypanosomiasis) | 5 | 0.038 |
| Cholinergic synapse | 5 | 0.046 |
| Acute myeloid leukemia | 4 | 0.031 |
| Glioma | 4 | 0.045 |
| p53 signaling pathway | 4 | 0.048 |

**Supplementary table 8 KEGG analysis of overlap DEGs for READ**

| Term | Count | PValue |
| --- | --- | --- |
| Proteoglycans in cancer | 8 | 0.029 |
| cGMP-PKG signaling pathway | 7 | 0.030 |
| Dopaminergic synapse | 6 | 0.041 |
| Platelet activation | 6 | 0.043 |
| Renin secretion | 5 | 0.015 |
| Aldosterone synthesis and secretion | 5 | 0.032 |
